# Programmatic access to ICTV virus taxonomy through a public ontology API

**DOI:** 10.1093/gigascience/giag089

**Published:** 2026-09-04

**Authors:** Philippe Lieutaud, James McLaughlin, R Curtis Hendrickson, Romain David, Helen Parkinson, Elliot J Lefkowitz, Donald M Dempsey, Bruno Coutard

**Affiliations:** Unité Des Virus Emergents (UVE: Aix-Marseille University, Universita Di Corsica, IRD190, Inserm 1207 IRBA), 25 boulevard Jean Moulin, 13005, Marseille, France; European Bioinformatics Institute (EMBL-EBI), European Molecular Biology Laboratory, Wellcome Trust Genome Campus, Hinxton, Cambridge CB10 1SD, United Kingdom; International Committee on the Taxonomy of Viruses (ICTV); University of Alabama at Birmingham, Department of Microbiology, Birmingham, Alabama 35294, United States; European Research Infrastructure on Highly Pathogenic Agents (ERINHA AISBL), 98 rue du Trône, B-1050 Bruxelles, Belgium; European Bioinformatics Institute (EMBL-EBI), European Molecular Biology Laboratory, Wellcome Trust Genome Campus, Hinxton, Cambridge CB10 1SD, United Kingdom; International Committee on the Taxonomy of Viruses (ICTV); University of Alabama at Birmingham, Department of Microbiology, Birmingham, Alabama 35294, United States; International Committee on the Taxonomy of Viruses (ICTV); University of Alabama at Birmingham, Department of Microbiology, Birmingham, Alabama 35294, United States; Unité Des Virus Emergents (UVE: Aix-Marseille University, Universita Di Corsica, IRD190, Inserm 1207 IRBA), 25 boulevard Jean Moulin, 13005, Marseille, France

**Keywords:** ICTV, virus taxonomy, ontology, API, FAIR principles, interoperability

## Abstract

**Background:**

The International Committee on Taxonomy of Viruses (ICTV) is responsible for developing and maintaining a universal virus taxonomy. As the reference framework for organizing the viral world, it is essential for virology and related fields. Despite its widespread use in research and public health, programmatic access to ICTV taxonomy has remained limited, posing challenges for integration, versioning, and interoperability across databases and bioinformatics resources requiring up-to-date virus taxonomy.

**Findings:**

To address this, we developed a public and sustainable solution leveraging ontology-based APIs. All available ICTV Master Species List (MSL) releases, from MSL1 to MSL41, were transformed into a unified, semantically structured ontology comprising more than 195,000 current and historical entities and deployed through the Ontology Lookup Service. The ontology is automatically rebuilt and republished whenever a new MSL release becomes available. Complementary ICTV-NCBI mappings and helper libraries support integration into downstream systems.

**Conclusions:**

Together, these resources enable, for the first time, public programmatic retrieval of current and historical ICTV taxon names, taxonomic relationships, metadata, and persistent identifiers through stable endpoints, including resolution of former taxonomic terms to their current accepted taxon or taxa and retrieval of taxon histories across releases. More broadly, this work illustrates a general strategy for transforming structured biological datasets into semantically enriched graph resources exposed through scalable public APIs. These developments enhance interoperability, reduce manual curation, and support Findable, Accessible, Interoperable, and Reusable (FAIR)-aligned taxonomic data management in virology and pandemic preparedness.

## Graphical Abstract

**Graphical Abstract:**
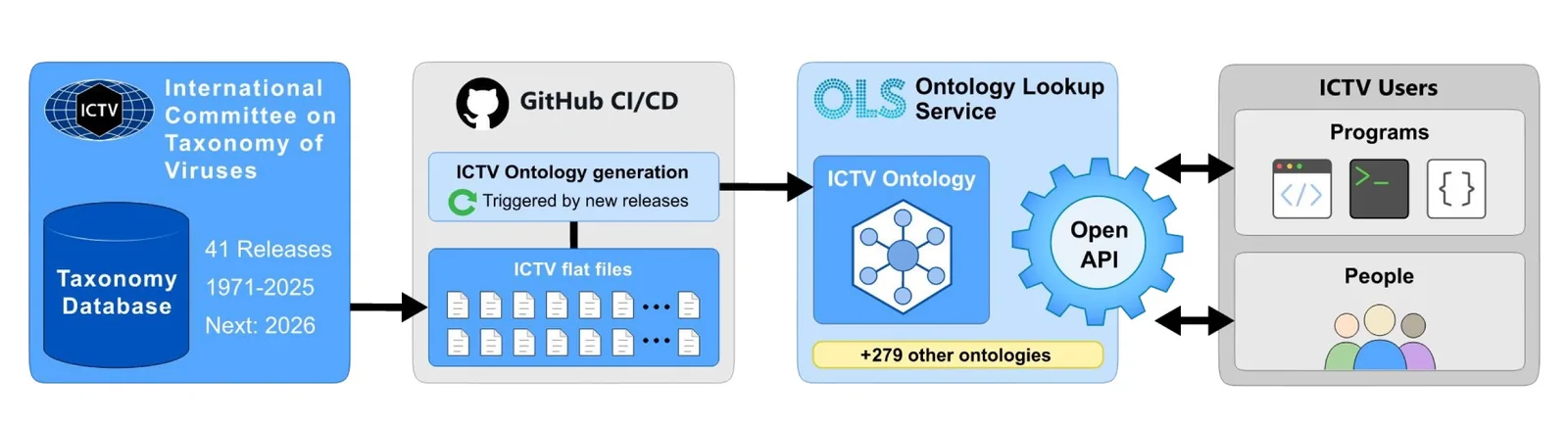
Unified ICTV ontology generation and public API access. Diagram showing 41 International Committee on Taxonomy of Viruses (ICTV) taxonomy releases transformed through GitHub continuous integration and continuous delivery (CI/CD) into a unified ICTV ontology hosted by the Ontology Lookup Service. An open application programming interface provides programmatic access for software and browsing access for users.

## Background

The International Committee on Taxonomy of Viruses (ICTV) [1], established under the Virology Division of the International Union of Microbiological Societies (IUMS) [2], is the authoritative body responsible for the classification of viruses and naming of virus taxa, ensuring a standardized and commonly accepted nomenclature. Virus taxonomy provides a structured framework for classifying viruses based on evolutionary relationships, shared biological properties, and genomic features, such as genetic material, structural features, replication strategies, and host range. While everyday virus names function as vernacular labels that can vary across studies and contexts, the ICTV taxon names provide formal, standardized designations that ensure unambiguous communication across the scientific community [3].

The regularly updated ICTV Master Species Lists (MSLs) [4] reflect ongoing advances in virology, including classification of newly discovered viruses and refined taxonomic insights, with one major new release each year, sometimes complemented by minor releases [5]. As virus discovery accelerates, particularly through metagenomics, maintaining consistent and up-to-date taxonomic references in databases and bioinformatics tools relying on ICTV taxa becomes increasingly important.

While the ICTV releases provide authoritative reference data, their distribution format is primarily designed for human consultation and does not readily support programmatic access [6]. As a result, integrating ICTV taxonomy updates into third-party computational systems remains challenging. Broader taxonomic platforms such as NCBI Taxonomy (National Center for Biotechnology Information) [7], GBIF (Global Biodiversity Information Facility) [8], COL (Catalogue of Life) [9], and Wikidata [10] embed ICTV releases as the authoritative source for virus taxonomy. However, these platforms often incorporate ICTV updates with substantial delays and may lack systematic support for resolving historical taxonomic changes.

Downstream resources can lag by one or more ICTV taxonomic updates, resulting in discrepancies in the classification they expose. Such inconsistencies create significant challenges for users who require the most up-to-date virus classifications, potentially affecting research accuracy and data consistency. For example, outdated species names may persist for some time in major sequence repositories such as GenBank [11] and the European Nucleotide Archive (ENA) [12], leading to further propagation of downstream inconsistencies. Since these repositories rely on the NCBI Taxonomy as their primary taxonomic reference, such delays can be observed by comparing historical taxonomic archives of NCBI and ICTV. A concrete example is ICTV renaming “*Severe acute respiratory syndrome-related coronavirus*” to “*Betacoronavirus pandemicum*” in MSL39. This change did not appear in NCBI Taxonomy for more than a year, and, when it did, was represented by the creation of a new NCBI taxon, rather than a direct renaming of the existing taxa. Obsolete taxon names that continue to be treated as current references complicate reproducibility and hinder interoperability across resources. These problems are inevitable, given that NCBI is not simply embedding ICTV taxonomy, but integrating it within a broader, sequence-linked system whose update process must preserve continuity for existing records and dependent workflows. As such, ICTV remains the authoritative source for virus taxonomy, while NCBI serves as a widely used integration layer. Consequently, taxon abolitions, splits, merges, moves, and renames may require specific handling rather than simple replacement. Such cases illustrate why programmatic resolution mechanisms are needed [13], along with traceable ICTV identifiers.

The European Viral Outbreak Response Alliance (EVORA) project [14] is a European initiative (2024–2026; grant no. 101131959) [15] that aims to enhance pandemic preparedness and response by strengthening research coordination, regulatory alignment, and data sharing among EU Research Infrastructures. Improving universal adoption of accurate and up-to-date virus taxonomy represents an important component of this mission. Members of EVORA and ICTV collaborated to provide a structured, machine-actionable version of the official ICTV taxonomy using ontology-based application programming interfaces (APIs), with support for historical name resolution, revision tracking, and cross-system identifiers.

Many biological data resources are distributed as relational databases, spreadsheets, or tabular datasets that are well suited for storage, but less adapted to programmatic integration across heterogeneous systems. In addition to addressing the immediate need for improved access to ICTV taxonomy, this work illustrates a broader methodological approach for transforming structured database content into semantically enriched knowledge representations through the use of ontologies. Ontologies play a crucial role in the management, integration, and analysis of biological data by providing explicit, human- and machine-readable semantics that support the consistent annotation, sharing, and interpretation of complex datasets [16]. Converting ICTV releases into an ontology enables graph-based querying and integration. As graph-based representations naturally capture hierarchical relationships, synonyms, and historical changes, representing taxonomy as an ontology therefore enables more expressive queries, automated reasoning, and easier integration with other semantic resources.

Across the life sciences, the Ontology Lookup Service (OLS) provides widely used infrastructure for searching, browsing, and accessing biological and biomedical ontologies through standard APIs, supporting the reuse of ontology terms and relations in data curation and computational workflows [17, 18].

## Methods

In this paper, “OLS API” refers to the generic programmatic interface provided by the OLS, including its Representational State Transfer (REST) endpoints and controllers. “ICTV ontology API” refers to access to the ICTV ontology through the OLS API, either directly via OLS endpoints or indirectly through ICTV-specific helper libraries built on top of them.

### Design requirements and use cases

To support practical integration of ICTV taxonomy into data systems and analytical workflows, several key use cases were defined to address common end-user challenges for automated processing. First, it must provide historical resolution by mapping obsolete names, synonyms, and ICTV identifiers from previous ICTV releases to their corresponding taxa in the current ICTV classification. Second, taxonomic changes must be tracked across releases, including taxa that have been abolished, split, merged, renamed, or reassigned (promoted) within the hierarchy. Third, cross-resource mapping must be supported, and, specifically, a mapping, with provenance, between NCBI Taxonomy identifiers and ICTV identifiers (historical and current) must be provided. Finally, there must be a stable, public, well-documented API for performing these functions.

### Persistent identifiers

Persistent identifiers are key for data integration [19]. The ICTV defines persistent identifiers for all versions of all taxa, as well as links connecting the taxa into hierarchies and documenting changes between releases. These unique identifiers facilitate resolution of outdated taxonomic terms to current ones by allowing taxa to be efficiently tracked across renames, rank changes, merges, splits, and abolitions between releases. To support programmatic resolution and cross-release linkage, and to further support Findable, Accessible, Interoperable, and Reusable (FAIR) principles, ICTV publicly documented these existing identifiers and defined Compact Uniform Resource Identifiers for them. ICTV provides 3 principal identifiers. In the shorthand used below, the letter prefix indicates the identifier type, and # represents the numeric identifier value:

- Taxnode identifier (Taxnode ID; TN#): uniquely identifies a taxon node within the ICTV source database in a specific release.
- ICTV identifier (ICTV ID; ICTV#): identifies the same taxon across releases, including through renames, changes in location within the taxonomic tree (moves), and changes in rank (promotion/demotion). Splits and merges create new ICTV IDs, which are linked back to their antecedent taxa.
- Isolate identifier (Isolate ID; VMR#): defined by the Virus Metadata Resource (VMR) [20], links GenBank sequence accessions for one or more segments to the vernacular name and the corresponding viral species [6].

During ontology generation, TN identifiers are used as ICTV source-table keys to reconstruct parent–child relationships within each release, associate VMR records with taxon nodes, and interpret changes between releases. They are not included in the ontology because their role is limited to processing the ICTV source data. Instead, each release-specific taxon state is represented as an OWL class and is assigned a resolvable Internationalized Resource Identifier (IRI) combining the MSL release and the persistent ICTV ID (e.g., the MSL41 representation of ICTV20040588 [21]). This design uniquely identifies each release-specific taxon state, preserves direct resolution across simple renames, and uses explicit revision and replacement relations when taxonomic events create new ICTV IDs.

**Figure 1:**
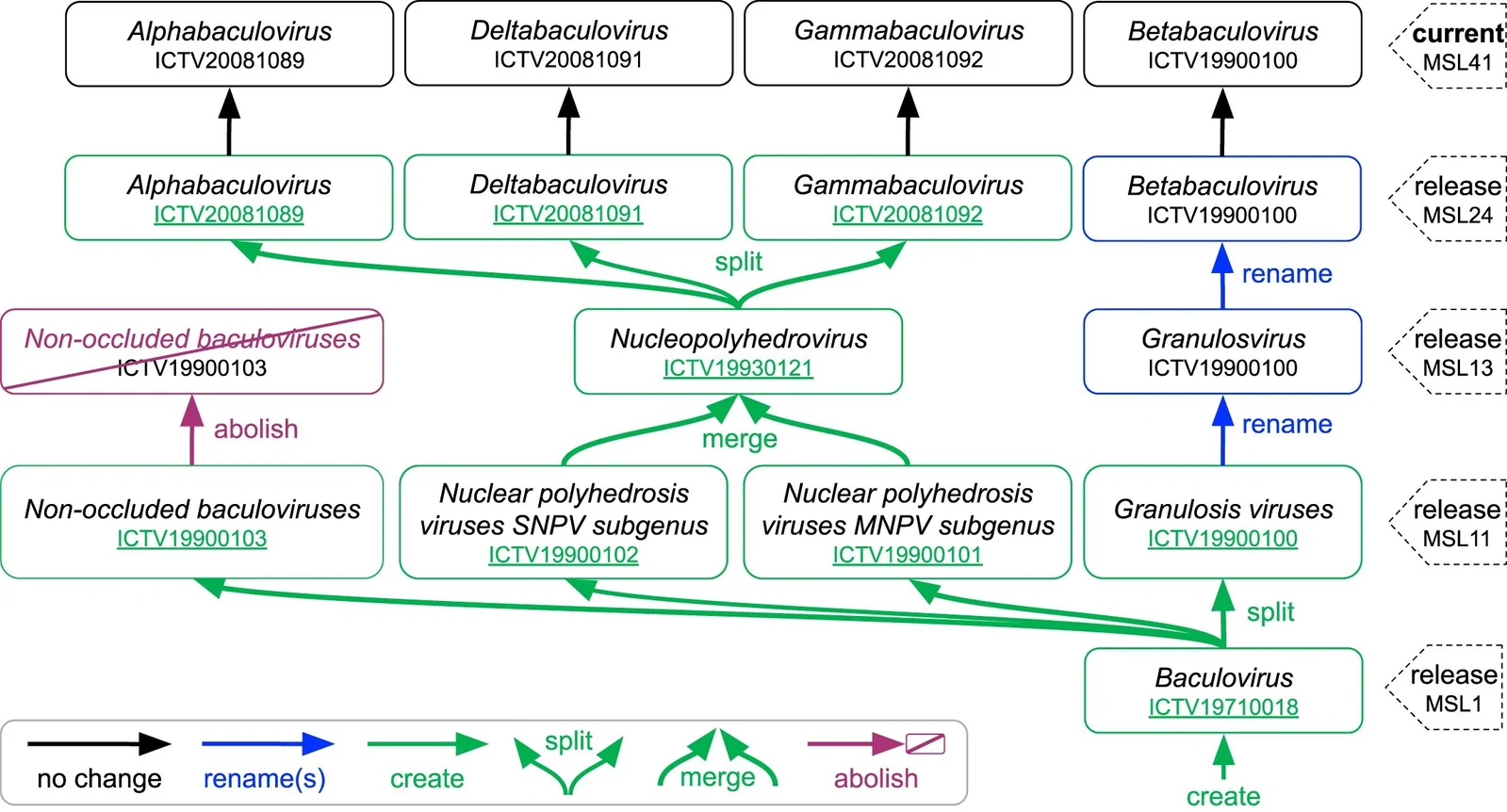
Illustrative taxonomic changes across ICTV releases for a genus-level taxon first recorded in MSL1. A diagram of historical and current genus names originating with the genus *Baculovirus* (ICTV19710018), first recorded in the first ICTV release, and ending with the 4 currently recognized genera. This example illustrates how the grouping of species into genera, although species are not shown here, can be redefined over time as viruses are better understood and characterized. In this historical trajectory, the original genus was first split into 4 genera; 2 of these were then merged; and the resulting lineage was later split into 3 genera. This illustrates some of the complexity that can occur in the history of a single taxon, and more broadly how taxonomic changes across different ranks and releases can make the overall taxonomy difficult to trace. It also shows how ICTV IDs are assigned to link historical versions of the same taxon. When a taxon is created, split, or merged (green single, diverging, or converging arrows, respectively), a new ICTV ID is assigned (underlined and in green). When taxa are renamed, promoted, demoted, or moved, the ICTV ID remains the same, linking the different versions.

This identifier design underpins the modelling of ICTV releases as semantically linked ontology resources. An extremely abridged example of the history of a taxon related to the genus *Baculovirus* in Fig. 1 illustrates some of the complexity, and demonstrates how ICTV IDs link historic versions of a taxon, from the first ICTV release to the current MSL41 release [22].

### Initial modelling of ICTV releases as ontologies

To meet the design requirements and use cases described above, including historical resolution, cross-release tracking and programmatic querying, each of the 41 (as of 2026) official ICTV taxonomy updates since 1971 was modelled as an independent OWL2 ontology, representing hierarchies, ranks, synonyms and revision metadata. Taxa were modelled as OWL classes, and virus isolates as OWL named individuals. This approach captures taxonomic relationships and their evolution over time while preserving the structural and semantic fidelity of the original ICTV data in a machine-readable format.

The ontologies were constructed using established semantic standards and interoperable vocabularies commonly used in biomedical ontology ecosystems (e.g., [23]), built upon Semantic Web standards including OWL, RDFS, PROV-O [24], and SKOS semantic relations [25], together with complementary vocabularies such as Dublin Core Terms, Friend Of A Friend, the Taxonomic Rank Vocabulary, and oboInOwl ontology annotations. This supports compatibility with the broader life-science ontology ecosystem [26] and reuses selected terms and annotations from OBO-associated resources [27]. Where a replacement exists, deprecated historical OWL classes are linked to their replacements using IAO:0100001 (term replaced by), enabling rapid resolution of former terms to their current representations.

The OLS enables direct exposure of ontology data through stable REST API controllers [28], a web-based user interface, and a Model Context Protocol server for access by AI agents. OLS provides a ready-to-use infrastructure to host ontologies and deliver programmatic access through standardized endpoints [17]. This approach eliminates the need to develop and maintain custom API infrastructures while ensuring compatibility with the broader ecosystem of life science ontologies. Thus, transforming existing data into a semantically structured graph representation leverages the OLS infrastructure to efficiently provide standard, public APIs and interoperability across data infrastructures.

The set of release-specific ontologies was published via OLS. The OLS API enables dynamic querying, retrieval of data, and linking across ontologies, allowing applications to access up-to-date information directly from ICTV data via the ontology stored in OLS.

### Redesign as a unified ontology

However, querying multiple ontologies proved complex for the primary use case of reconciling historical references with the current taxonomy. The system was therefore redesigned to merge all releases into a single, unified ontology (over 195,000 terms in 2026). In this model, the most recent ICTV release represents the authoritative classification, while entities from previous releases are preserved as deprecated historical OWL classes and, where applicable, linked through explicit replacement relations. This design reduces query complexity while preserving full history, provenance, and backward compatibility across ICTV releases. This enables any taxonomic reference appearing in historical datasets or publications to be programmatically reconciled with the current ICTV classification.

The ontology is primarily functional: it was engineered to expose current and historical ICTV taxonomy through OLS and the helper libraries so that taxon identity, lineage, synonyms, and revision history can be queried programmatically. It does not import BFO, COB, or RO, has not been submitted to the OBO Foundry, and no submission is currently planned.

**Figure 2:**
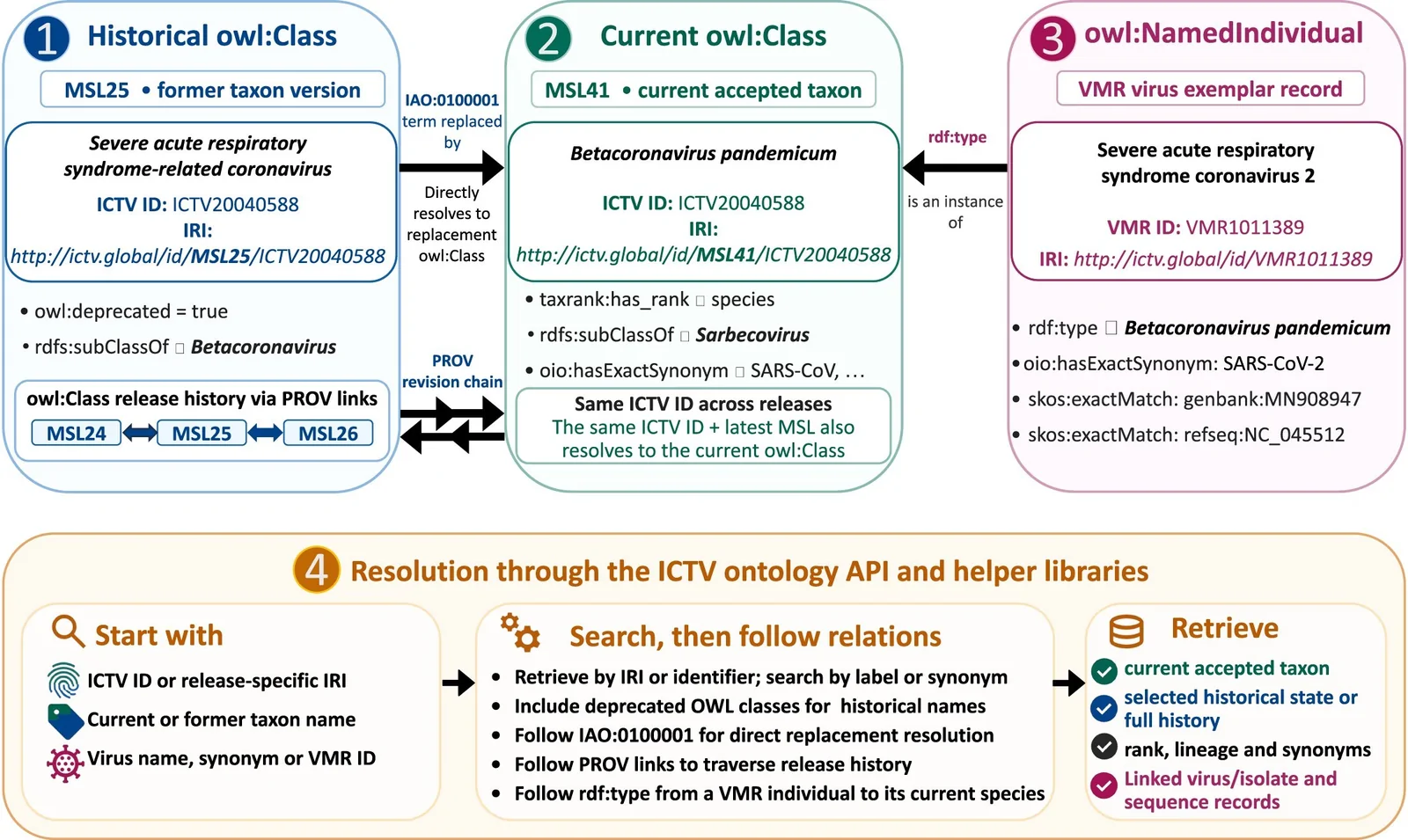
Historical and current taxon resolution using ICTV taxon and VMR virus record information. A former MSL25 taxon version is represented as a release-specific OWL class (i.e., owl:Class) (1), marked as deprecated with “owl:deprecated = true,” and linked through IAO:0100001 to its current MSL41 representation, while PROV relations connect successive historical release states. The current accepted taxon is represented by a distinct OWL class carrying the same stable ICTV identifier (2). In simple taxon rename paths, this identifier can also be combined with the latest MSL release to retrieve the corresponding current OWL class. A VMR virus exemplar record is represented as an owl:NamedIndividual and asserted as an instance of its corresponding species OWL class using rdf:type (3). The resolution workflow (4) shows how ICTV identifiers and release-specific IRIs, current or former taxon names, and virus names, synonyms, or VMR identifiers can be used through the ICTV ontology API and helper libraries to retrieve current taxa, historical states, lineage, synonyms, and linked sequence records. In the context of this ontology, an OWL class represents an ICTV taxon and should not be confused with the ICTV rank class; rdfs:subClassOf expresses hierarchical relations between these ontology entities. The statement owl:deprecated = true applies to all historical, release-specific OWL classes representing taxa. Deprecated OWL classes linked by either an IAO:0100001 (replaced by) relation or prov:hadRevision link have been updated in a subsequent release. Only OWL classes with owl:deprecated = true and neither an IAO:0100001 relation nor a prov:hadRevision link represent truly abolished taxa.

Figure 2 illustrates how the entities and relations in the unified ontology support taxon resolution across ICTV releases. Release-specific taxon representations, modelled as OWL classes, are connected through PROV revision relations and, when a replacement is defined, through IAO:0100001 to the corresponding OWL class in the latest MSL, which represents the currently accepted ICTV taxon. In simple taxon rename paths, reusing the stable ICTV ID with the latest MSL release also retrieves the current OWL class, whereas IAO:0100001 provides a more direct route to the replacement OWL class and can resolve replacement or merge events without traversing the full release history. VMR virus exemplar records modelled as OWL named individuals are linked by rdf:type to their corresponding species OWL classes in the ontology. Together, these relations allow identifiers and current or former taxon or virus names to be resolved to current taxa, to defined historical states, or to complete taxon histories through the OLS API and helper libraries.

### Availability and sustainability

Continuous Integration and Deployment (CI/CD) workflows implemented through public GitHub repositories automatically trigger the ontology generation workflow for each release published in the ICTV’s official GitHub repository [29]. This automation removes manual intervention and contributes to long-term sustainability of the infrastructure, in line with recommendations for sustainable FAIR implementation in life sciences [30]. The unified ontology is immediately available as a release from the EVORA GitHub repository [31], and is automatically deployed to the OLS, typically within 24 h of an ICTV release, which provides public web browsing and API access. Dissemination and long-term discoverability are further supported through versioned GitHub and Zenodo releases, as well as registration of the software resources in bio.tools and SciCrunch (see the Availability of supporting source code and requirements section).

### ICTV-NCBI mapping

To improve interoperability at the broader scale [32], ICTV-NCBI mappings are generated using the OLS API, and stored in SSSOM (Simple Standard for Sharing Ontology Mapping) format, aligning ICTV identifiers with NCBI Taxon identifiers [33]. The code to generate this mapping is maintained in a dedicated GitHub repository [34], where the resulting SSSOM files are then published as releases (see Additional file 1, Supplementary Material S4). While this analysis is also automated using a GitHub CI/CD workflow, it runs on a schedule, decoupled from the release cycles of the 2 ontologies, allowing independent updates while remaining consistent with the continuously updated ontology infrastructure.

These mappings not only facilitate direct and bidirectional linkage between ICTV and NCBI taxonomies, but also enable alignment across downstream resources that rely on NCBI as a taxonomic backbone, including sequence repositories and bioinformatics platforms. This feature enables broader platforms that rely on the NCBI taxonomy for viral references (e.g., ENA) to stay current with the ICTV taxonomy while continuing to leverage the broader coverage of NCBI taxonomy.

The NCBI integrates the ICTV Taxonomy into its own taxonomy system, which is used to designate species related to the entries in its sequence databases (e.g., GenBank). This integration is supported by an established exchange process between the ICTV database team and the NCBI team responsible for virus taxonomy updates. This process predates the present work and accommodates NCBI’s specific sequence-linked update requirements. The public ICTV ontology API and ICTV-NCBI mappings complement this process by providing a general, community-facing, and machine-actionable layer for accessing and reconciling current and historical ICTV taxonomy. Historically, ICTV updates have been incorporated with roughly a one-year delay, resulting in NCBI being one or 2 releases behind the latest ICTV version (as described above). Observations following MSL41 release suggest a recent effort by NCBI to shorten this delay, with progressive integration starting in May 2026, about 2 months after the MSL41 release. This included the integration of renamed taxa, while newly created taxa remained absent and abolished taxa had not yet been removed (see Additional file 1, Supplementary Material S3). As part of this work, ICTV has publicly documented several persistent identifiers (described above) that link taxa in successive ICTV Taxonomy versions, which should facilitate rapid integration of new releases. These identifiers are exposed in the ICTV ontology metadata and support the generation of SSSOM ICTV-NCBI mappings. In the released mapping file, lexical ICTV-to-NCBI Taxon correspondences are expressed with skos:exactMatch, enabling alignment between ICTV taxonomy and NCBI Taxonomy identifiers used by downstream sequence resources such as GenBank.

## Results

### Unified ontology for ICTV taxonomy and OLS deployment

We have produced a unified ontology for the ICTV taxonomy, aggregating all releases into a single resource. The ontology is available for download or via OLS and enables programmatic access to current and historical classifications. Ontology updates are automatically triggered by new ICTV releases and reflected in OLS, typically within 24 h.

**Figure 3:**
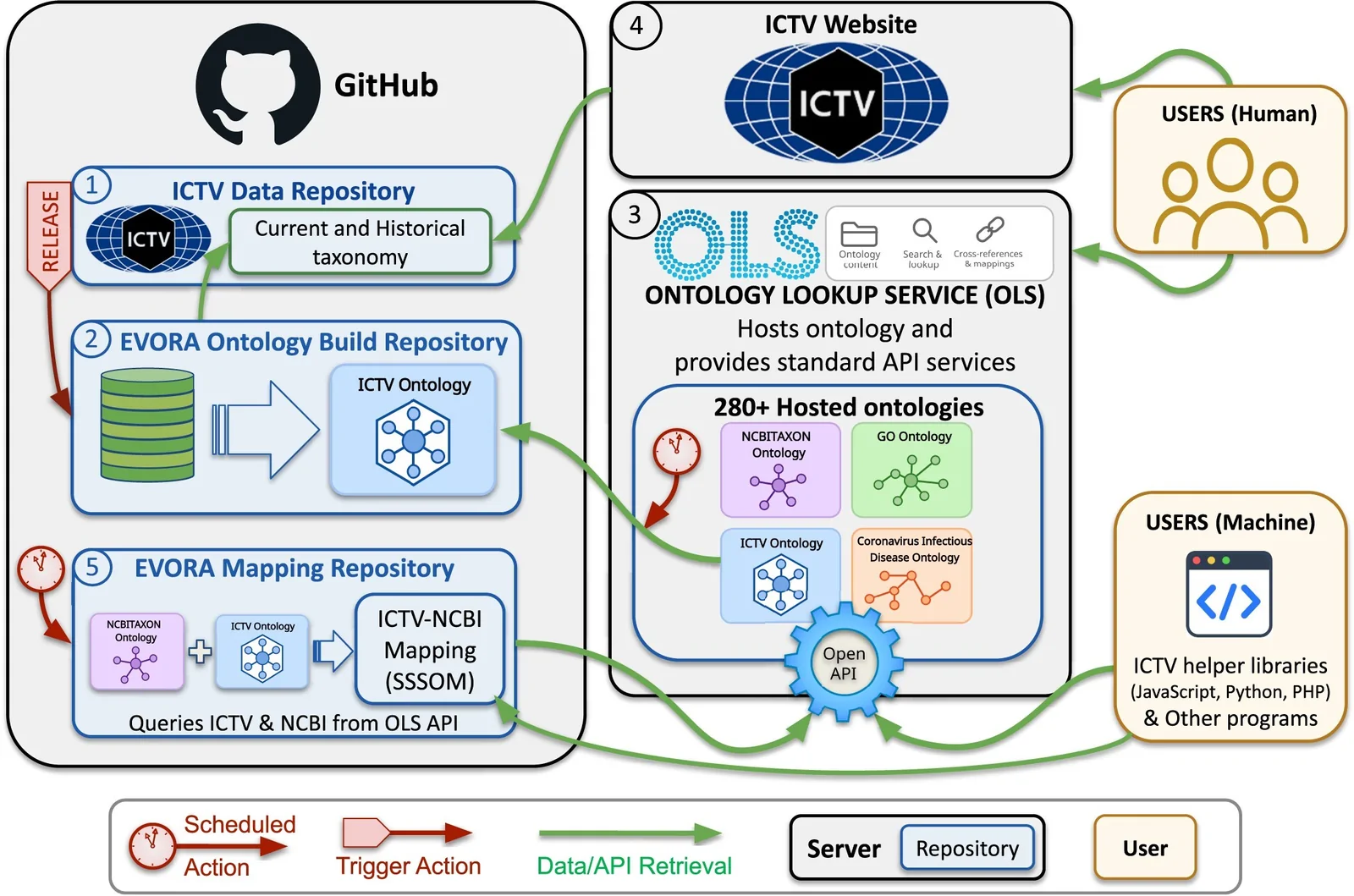
Automated data flows for ICTV ontology generation, publication, ICTV-NCBI mapping, and API access. Official ICTV taxonomy data are released through the public ICTV Data repository (1), hosted on GitHub. A new release triggers the EVORA CI/CD workflow hosted in the EVORA Ontology Build repository (2), which retrieves the ICTV release files, transforms them into OWL ontology artefacts and merges them into a unified ICTV ontology. In this unified ontology, the latest ICTV release represents the current authoritative taxonomy, while entities from previous releases are retained as deprecated historical OWL classes linked through explicit semantic relations. Through scheduled actions, the unified ontology is loaded into the OLS (3), which provides both a browsable human interface and standard API services. The same ICTV source data also feed the ICTV website (4), which provides the official human-facing access point to ICTV taxonomy. In parallel, a separate EVORA Mapping repository (5) runs scheduled workflows that query OLS to use the latest ICTV and NCBITaxon ontologies to generate ICTV–NCBI mappings in SSSOM format; these mappings are stored in the corresponding public repository. Downstream access is supported through direct API queries and language-specific OLS ICTV API helper libraries, enabling external applications to perform historical taxon resolution, cross-resource mapping, and to reduce manual curation. Green arrows indicate data/API retrieval actions; dark red arrows indicate workflow trigger actions; dark red clocks indicate scheduled actions. Black boxes represent servers, blue boxes repositories, and brown boxes users.

Asynchronously, ICTV-NCBI mappings are generated in SSSOM format, enabling bidirectional resolution between the taxonomies. Mappings are maintained in a dedicated EVORA virus-taxonomy-mappings repository and updated independently [34]. The mapping supports bidirectional many-to-many resolution between ICTV and NCBI taxonomies and facilitates integration with downstream resources. The overall architecture of the generation and publication workflow is shown in Fig. 3.

To simplify integration into downstream applications, ICTV-specific helper libraries were developed on top of the standard OLS API. These libraries are provided in JavaScript, Python, and PHP and encapsulate resolution logic (synonyms, obsolescence, lineage, mappings) while returning normalized data about taxon objects. These objects contain identifiers, labels, synonyms, ranks, lineage, obsolescence metadata and cross-references, enabling application developers to integrate ICTV-aware logic without direct manipulation of raw API calls. The helper libraries prioritize exact identifier and name-based routes before free-text fallback. A bare ICTV ID returns the corresponding OWL class from the latest matching MSL release by default, while an explicit MSL release retrieves the corresponding historical state. For VMR records represented in the ontology, a VMR identifier resolves through the corresponding OLS individual and its rdf:type relation to the current species OWL class.

The OLS API documentation, ICTV-specific guidance and helper libraries are publicly available [35] (see Additional file 1, Supplementary Material S1). Together, the unified ontology, its OLS deployment, the helper libraries and the complementary SSSOM mappings constitute an integrated ontology-based access resource for automated resolution, cross-taxonomy linkage, and reproducible versioning.

### Reference application and early adopters

**Figure 4:**
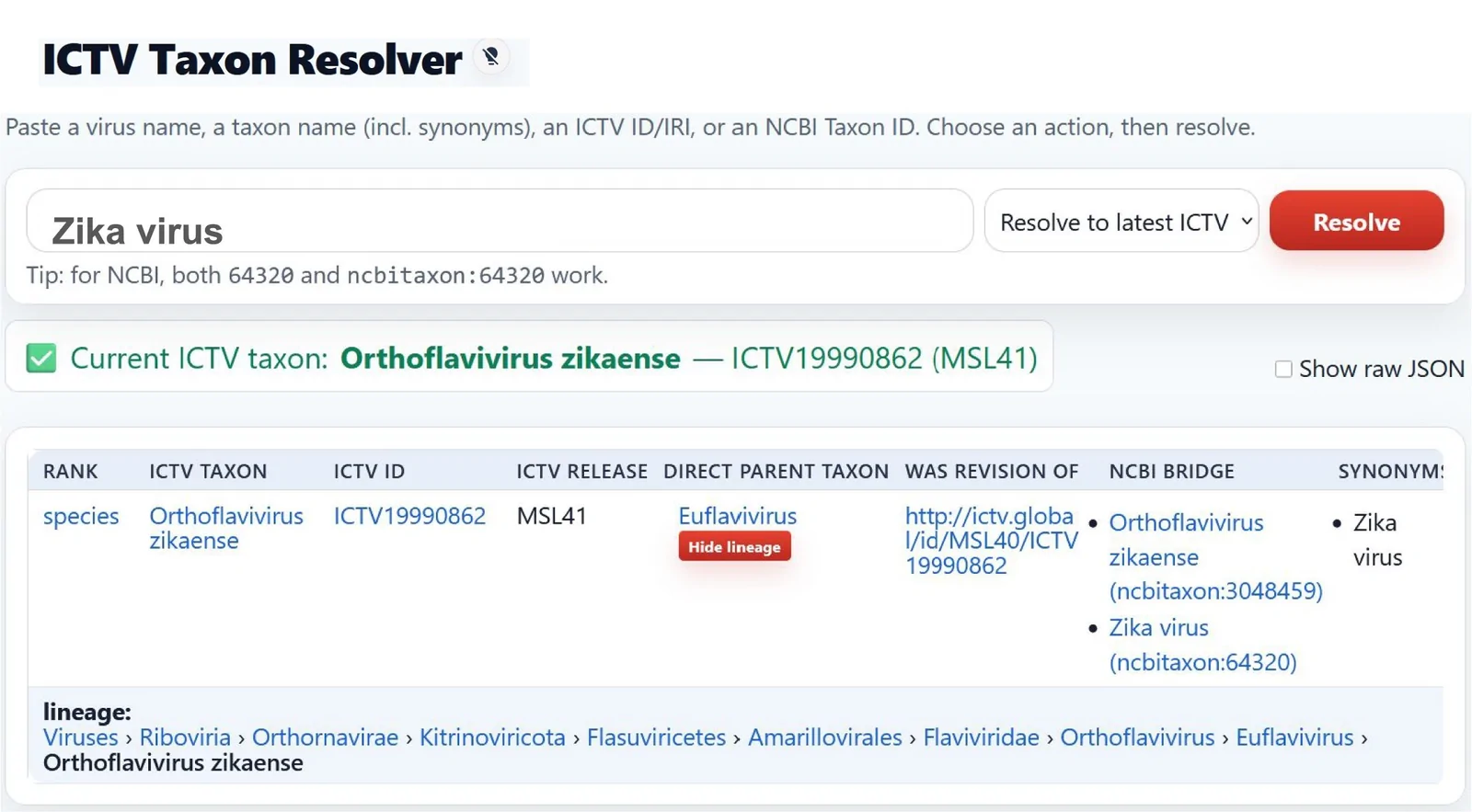
ICTV Taxon Resolver and programmatic taxonomic resolution. The ICTV Taxon Resolver interface illustrates automated resolution of historical or synonymous virus names to the currently accepted ICTV taxon, including lineage reconstruction and cross-references to external identifiers. The resolver is a reference implementation built on the JavaScript helper library that encapsulates OLS API calls. It accepts heterogeneous user inputs, including historical virus names, ICTV identifiers, IRIs, synonyms, and NCBI Taxon identifiers, and applies a resolution strategy combining label matching, synonym detection, obsolete-term replacement chains, and cross-taxonomy mappings. The output panel displays the currently accepted ICTV taxon together with its hierarchical lineage, rank, revision history, and available cross-references to external resources.

A public demonstration interface, the ICTV Taxon Resolver [36] web application, was developed as a reference implementation using the JavaScript helper library. The interface provides 2 selectable actions. The default action, “Resolve to latest ICTV,” returns the current accepted ICTV taxon when one can be resolved. “Show history across MSLs” starts from the OWL class representing the taxon identified from the query and displays the release-specific OWL classes linked to it across the MSL releases included in the ontology. Consequently, the displayed history may begin after MSL1 or end before MSL41, for example, when a taxon was introduced later or subsequently abolished. The source code is available from the EVORA ICTV Resolver GitHub repository [37]. The resolver allows users to submit current and historical ICTV taxon names and virus names, ICTV identifiers, IRIs, or NCBI Taxon IDs and receive the corresponding currently accepted ICTV taxon or taxa, together with their full lineages and revision history. Concise usage guidance is displayed directly beneath the interface title. This tool illustrates practical applicability while serving as a functional community resource. The interface and an example resolution of the former species name *Zika virus* to its post-2022 name and lineage, *Orthoflavivirus zikaense*, are shown in Fig. 4. For example, in history mode, the relationship between *Alphabaculovirus* and *Nucleopolyhedrovirus* illustrated in Fig. 1 can be traced from MSL23 back to MSL13. The resolver is a demonstration and reference implementation built on the JavaScript helper library and does not expose every capability of the OLS-backed ICTV ontology API, for which complete developer documentation is available separately.

Among the first operational deployments using the ICTV ontology API is the EVORA Portal [38], a consolidated catalogue of research infrastructures’ resources and services in virology designed to support outbreak response and pandemic preparedness. The utilization of the ICTV ontology API is integrated through one of the helper libraries directly into the catalogue generation workflow, enabling retrieval of up-to-date taxonomic terms along with associated lineage and synonym information. This ensures consistent use of the latest ICTV taxonomy, even when metadata provided by contributing research infrastructures correspond to earlier ICTV releases. As an added value for users of the research infrastructures, lineage information and historical synonyms retrieved through the ontology API enable enhanced search capabilities. This allows users to find resources using outdated taxa or alternative names, an option that was not available in the former search module.

Early adoption of the ICTV ontology API has also been observed in an external initiative concerned with pandemic preparedness and data integration: the Pandemic PACT programme [39]. This programme is now using ICTV taxonomy via the OLS API to align viral pathogen families and scientific names within its scope with the latest ICTV taxonomy (see the Acknowledgements section). The retrieved ICTV metadata will be incorporated into Pandemic PACT dashboard visualizations [40], where they will be integrated with metadata from other ontologies accessed through the OLS, such as the Systematized Nomenclature of Medicine, aligning up-to-date viral pathogen taxonomy with related information, including associated diseases.

Integration of ICTV taxonomy through an API enables timely and automated updates across resources. The European Virus Archive (EVA) [41], a network of distributed virus collections using a single web catalogue for virus and virus-related material ordering and distribution [42], plans to integrate the ICTV ontology into its workflows using OLS API queries to maintain local references to virus taxonomy aligned with the most recent ICTV releases. This will allow EVA datasets to remain synchronized with official taxonomy updates while preserving historical annotations associated with archived materials, which will enhance the interconnectivity of research and data management processes, improving resource discovery and data consistency.

These deployments demonstrate practical applicability across research infrastructures and public health platforms. The system enables automated resolution, cross-taxonomy linkage, reduced manual curation, and reproducible versioning.

## Discussion

Virus data resources include numerous databases with different scopes, data models, functionalities, and degrees of FAIR maturity, as reflected in recent reviews of virus databases [43]. This heterogeneity creates practical challenges for interoperability and synchronization with authoritative taxonomic references such as ICTV taxonomy. This work demonstrates how relational datasets can be easily transformed and exposed through scalable, public, ontology-based APIs. Standards-based ontology-enabled APIs drastically reduce the work needed to make datasets machine-actionable. Here, we developed an ontology for the widely used and regularly updated ICTV virus taxonomy and exposed it through a public API, providing downstream systems with programmatic access to current and historical taxonomic information and the ability to resolve former terms to the current classification, thereby supporting uses ranging from direct queries to recurring automated updates. The ontology infrastructure acts as a semantic resolution layer that continuously bridges evolving authoritative ICTV releases with downstream computational ecosystems and legacy taxonomic references. Timely updates, in turn, reduce discrepancies that arise when downstream systems rely on outdated or partially synchronized taxonomic references and enable reproducible taxonomic resolution across computational workflows. Research infrastructures, sequence repositories, biodiversity catalogues, and knowledge bases can use automated alignment mechanisms to maintain consistency with official ICTV classifications, reducing the need for extensive manual curation.

Persistent ICTV identifiers and replacement relations provide a structured mechanism to track taxonomic evolution, historically prone to ambiguity and data fragmentation, in a computationally accessible way. Complementary ICTV-NCBI mappings, together with ontology cross-references to associated sequence resources, provide a pragmatic bridge between the authoritative, annually updated ICTV classification and NCBI taxonomy. This improves reproducibility and supports FAIR principles, and their progressive implementation [44–46]. While this approach substantially improves interoperability and historical resolution, synchronization across downstream resources still depends on heterogeneous update cycles and integration strategies. In addition, some complex taxonomic transitions, such as splits, merges, or taxon abolitions, may still require complementary expert interpretation in specific downstream analytical contexts.

These capabilities help connect distributed data systems in FAIR-aligned federated environments, where independently maintained resources must remain discoverable, accessible, and interoperable, including initiatives such as the European Open Science Cloud (EOSC) and the European Health Data Space (EHDS).

Consistent and machine-actionable taxonomy references support large-scale computational analyses, automated data integration workflows and reproducible comparative studies by reducing ambiguity in taxonomic interpretation. This contributes to improved retrieval and harmonization of virological data across interoperable research infrastructures and data-sharing environments [47], supporting more reliable data integration, comparative analyses, evidence-based decision-making, and more informed experimental design and data interpretation. The infrastructure also establishes a foundation for future enhancements, including expanded mappings with sequence repositories and the possible development of additional interfaces relying on the public OLS API.

Our approach aligns with the article “Four principles to establish a universal virus taxonomy” [48] that outlines key recommendations to achieve a coherent and comprehensive virus taxonomy. Our work helps make ICTV taxonomy more useful by ensuring that references to the official, evolution-based ICTV taxonomy can be easily maintained and kept up-to-date, irrespective of the intended use or alternate classification scheme. For example, classifying viruses to support the surveillance and public health response to disease outbreaks (pathogens prioritization: a scientific framework for epidemic and pandemic research preparedness) [49] results in a classification that is significantly different than that of the ICTV taxonomy. It is however still important to reference the current evolutionary-based classification so that our current knowledge on viruses can be quickly and easily adapted to newly identified or newly emerging ones. The ICTV taxonomy serves as the universal reference for virus classification, while the services we report here enable the critical links needed to connect that taxonomy to alternate schemes.

Adoption of the ICTV ontology API by resources relying on virus taxonomy (EVORA and Pandemic PACT) demonstrates its practical applicability. Ontology-based APIs can facilitate alignment between biological taxonomy resources and clinical terminologies used in public health systems, enabling consistent pathogen identification across research infrastructures, data catalogues, and policy-oriented dashboards. By supporting automated retrieval of up-to-date taxonomic terms, lineage information, and synonym relationships, the API-based approach supports consistent taxonomy usage across distributed data infrastructures and reduces the need for manual synchronization of taxonomic references similarly to interoperability approaches explored in tools such as Treemendous for reconciling taxonomic backbones in tree species naming [50].

Taxonomic resources and biodiversity platforms such as NCBI Taxonomy and GBIF could use the OLS API to implement more automated synchronization with ICTV releases. Databases such as ENA and GenBank, which depend on the NCBI taxonomy and its integration of ICTV classifications [51], would in turn benefit from improved data integration and accuracy. In cases where updates of virus taxonomy within NCBI do not immediately reflect the latest ICTV release, these resources could additionally leverage the ICTV ontology API, coupled with the provided SSSOM mappings, to maintain up-to-date virus taxonomy.

Our work builds on established FAIR and semantic standards [26, 52]. The inclusion of detailed metadata and the reuse of established semantic standards are critical for ensuring that the ICTV taxonomy serves as a reliable reference across different platforms and applications. This approach provides a lightweight and sustainable mechanism for publishing structured datasets as interoperable APIs, by leveraging an existing ontology service (OLS) and GitHub’s publicly available workflow automation. This approach is applicable to other domain-specific datasets. In parallel with the public OLS-based service, ICTV is using a dedicated access mechanism for data exchange with NCBI and plans a broader API providing access to a more comprehensive ICTV dataset. These complementary services support specialized or higher-volume exchanges, while the OLS API remains the public, standards-based access layer.

More broadly, this work illustrates how ontology technologies can expose structured datasets as semantically enriched graph representations through interoperable APIs. Traditional database schemas are optimized for storage and transactional operations but often require custom APIs to expose their content programmatically. In contrast, ontology-based representations allow relationships between entities to be explicitly encoded and navigated as a graph, enabling more flexible data integration and discovery. Such representations align with knowledge graph and Linked Open Data approaches [53, 54]. More generally, this work demonstrates a reusable pattern for exposing structured, domain-specific datasets as interoperable ontologies and APIs by leveraging established ontology infrastructures, such as the OLS for biomedical ontologies.

## Conclusion

This work shows that representing an evolving classification system, such as the ICTV taxonomy, as a unified ontology hosted through a public, scalable, standards-based API, increases its accessibility and addresses key challenges in access, versioning, and interoperability. The resulting infrastructure supports backward-compatible resolution of virus taxonomy across historical ICTV releases, enabling historical traceability and alignment with current classifications through semantic modelling, persistent identifiers and interoperable mappings.

By building on open standards, automated workflows and existing ontology infrastructure services, this approach supports long-term sustainability and scalability while making ICTV taxonomy more readily integrable into computational workflows. It also facilitates synchronization between authoritative taxonomic references and downstream biological, biomedical and public-health data infrastructures, supporting broader adoption of the latest official ICTV taxonomy as the reference standard and strengthening data consistency, reproducibility and preparedness for emerging viral threats, including through more timely dissemination of high-impact updates such as the pandemic-based designation of the species *Betacoronavirus pandemicum*.

Future developments may support broader interoperability with additional taxonomy providers, sequence repositories and interconnected semantic resources. More broadly, this work provides a reusable model for maintaining, exposing and reusing evolving taxonomic references through sustainable, machine-actionable semantic infrastructure.

## Availability of supporting source code and requirements

- Project name: ICTV Ontology API and helper libraries
  - Project home page: https://github.com/EVORA-project/ictv-ontology.
  - Documentation: https://evora-project.github.io/ictv-ontology/helpers/.
  - Operating system(s): Platform independent.
  - Programming language: language-agnostic for direct API access; helper libraries are provided in Python 3.8 or later, JavaScript as an ES module, and PHP 7.4 or later.
  - Other requirements: Internet access to the OLS API; depending on the helper library used, the Python requests library, the JavaScript Fetch API or the PHP cURL extension is required. For PHP, allow_url_fopen must also be enabled when accessing the remote ICTV-NCBI SSSOM mapping.
  - Licences:
    - ontology-generation code, Apache License 2.0;
    - ICTV-derived ontology data, Creative Commons Attribution 4.0 International (CC BY 4.0), with attribution to ICTV;
    - JavaScript, Python and PHP helper libraries, MIT License.
  - Version used for this manuscript: GitHub release 2026-Jul-28–74d7801; Zenodo version DOI 10.5281/zenodo.21506852.
  - All versions: 10.5281/zenodo.20610328.
  - RRID:SCR_028724
  - bio.tools ID: ictv_ontology_api.
- Project name: ICTV-to-NCBITaxon SSSOM mapping
  - Project home page: https://github.com/EVORA-project/virus-taxonomy-mappings.
  - Operating system(s): Platform independent.
  - Programming language: language-agnostic for SSSOM file utilization; SSSOM file generation code in Python.
  - Release archived at https://doi.org/10.5281/zenodo.21890424 [55].
  - License: CC0 1.0 Universal.
  - RRID:SCR_028725
  - bio.tools ID: ictv_to_ncbitaxon_sssom_mapping.
- Project name: ICTV Taxon Resolver
  - Project home page: https://github.com/EVORA-project/ictv-resolver.
  - Live instance: https://evora-project.github.io/ictv-resolver.
  - Programming languages: HTML, JavaScript, and CSS.
  - Other requirements: A modern web browser with JavaScript and internet access to OLS.
  - License: MIT.
  - Version used: v1.0.5.
  - RRID:SCR_028722.
  - bio.tools ID: ictv_taxon_resolver.
- External service dependency: Ontology Lookup Service (OLS):
  - Project home page: https://www.ebi.ac.uk/ols4/.
  - Documentation: https://github.com/EBISPOT/ols4/blob/dev/README.md [56].
  - Source code: https://github.com/EBISPOT/ols4.
  - Software licence: Apache License 2.0.

## Additional files

**Additional file 1:** Additional file 1_Supplementary material.pdf.

Supplementary Materials S1–S4 provide API usage examples, the helper-library architecture, a comparison of virus taxonomy providers and the ICTV-NCBI mapping specification.

## List of abbreviations

API(s): Application Programming Interface(s); CI/CD: Continuous Integration and Deployment; COL: Catalogue of Life; CURIE: Compact Uniform Resource Identifier; DCTERMS: Dublin Core Terms namespace; ENA: European Nucleotide Archive; ETL process: Extract, Transform, and Load; EVA: European Virus Archive; EVORA: European Viral Outbreak Response Alliance; FAIR: Findable, Accessible, Interoperable and Reusable; FOAF: Friend of a Friend ontology; GBIF: Global Biodiversity Information Facility; IAO: Information Artifact Ontology; ICTV: International Committee on Taxonomy of Viruses; IRI: Internationalized Resource Identifier; LOD: Linked Open Data; MCP: Model Context Protocol; MSL: Master Species List; NCBI: National Center for Biotechnology Information; OLS: Ontology Lookup Service; OWL: Web Ontology Language; PROV-O: Provenance Ontology; RDFS: Resource Description Framework Schema; REST: Representational State Transfer; SKOS: Simple Knowledge Organization System; SNOMED: Systematized Nomenclature of Medicine; SSSOM: Simple Standard for Sharing Ontology Mapping; TAXRANK: Taxonomic Rank Vocabulary; VMR: Virus Metadata Resource.

## Supplementary Material

giag089_Supplemental_Files

giag089_Authors_Response_To_Reviewer_Comments_original_submission

giag089_GIGA-D-26-00217_Original_Submission

giag089_GIGA-D-26-00217_Revision_1

giag089_Reviewer_1_Report_original_submission Reviewer 1 -- 7/9/2026

giag089_Reviewer_1_Report_Revision_1 Reviewer 1 -- 8/31/2026

giag089_Reviewer_2_Report_original_submission Reviewer 2 -- 7/20/2026

## Data Availability

All ontology artefacts, helper libraries, mapping resources, example notebooks, and continuous-integration workflows are publicly available under open licences. The ICTV MSL41 source data used in this work are available as the ICTVdatabase MSL41.v1 release [22]. The exact EVORA ICTV ontology release used for this manuscript is archived in Zenodo as EVORA-project/ictv-ontology: 2026-Jul-28-74d7801 [60], and its complete version history is available through the corresponding Zenodo concept record [61]. The exact ICTV-NCBI mapping release is archived in Zenodo as EVORA-project/virus-taxonomy-mappings: Virus Taxonomy Mappings Update, release-3 [55], and its complete version history is available through the corresponding Zenodo concept record [62]. Current development versions and latest releases are available from the ICTV database, ICTV ontology, ICTV-NCBI mapping, and unified OLS resources [ 29, 31, 34, 63]. All resources are reusable, openly licensed, and versioned, enabling independent verification and downstream integration. NCBI Taxonomy taxdump archives used to verify the integration status of ICTV releases are available from the NCBI Taxonomy archive [64].
