# Supplementary material for: Programmatic access to ICTV virus taxonomy through a public ontology API
Source: Gigascience. 2026 Sep 4;15:giag089. doi: 10.1093/gigascience/giag089 (PMC13615544; doi:10.1093/gigascience/giag089)
Supplement: Supplementary file 1 [file giag089_authors_response_to_reviewer_comments_original_submission.pdf]

Author's Response To Reviewer Comments

Dear Editor and Reviewers,

We thank the Editor and Reviewers for their careful and constructive assessment. We revised the manuscript and supporting resources accordingly. The comments concerned clarification, documentation and presentation; no new scientific data or analyses were required. The responses below address the reviewer comments point by point, and all substantive revisions are described below and incorporated in the revised manuscript.

Summary of principal revisions

- Clarified complete MSL1–MSL41 coverage, the public release-specific IRI design and the source-only role of TN identifiers.

- Added Figure 2 and accompanying text explaining historical and current taxon resolution, PROV and replacement relations, and VMR isolates as OWL NamedIndividuals linked to species OWL classes.

- Clarified the complementary roles of ICTV, NCBI Taxonomy, the public ontology API and the ICTV–NCBI mappings; named and archived the mapping resource.

- Expanded direct API, helper-library and Taxon Resolver documentation, including worked examples in Supplementary Material S1 and the Figure 4 result screenshot.

- Applied terminology, accessibility and presentation revisions, including context-sensitive use of 'virus taxonomy', qualified FAIR language and the colour-blind-safe Figure 1 palette.

Location note. Section locations are provided below and correspond to the clean, line-numbered revised manuscript.

Response to Reviewer 1

We thank the reviewer for the detailed comments and respond point by point below.

R1.1

Comment

Have NCBI been included in conversations about these developments? It seems strange that representatives from EMBL-EBI are authors but there is no inclusion of partners from NCBI when NCBITaxon is a key resource for many platforms, application ontologies, and tools. Could the authors mention engagement efforts with NCBI so we understand whether the ICTV IDs and synonymy will be adopted? I think this is important as well as the authors pointedly describe the challenges of updating taxonomy at NCBI, but don't note that major consumers/providers of data are public health agencies. Every change in NCBITaxon breaks critical workflows in systems used in routine public health operations. NCBI is caught between a rock and a hard place every time ICTV makes changes - wanting to respect expert recommendations while managing pushback from the community e.g. there were/are many challenges of adopting the change of 'Severe acute respiratory syndrome-related coronavirus' to 'Betacoronavirus pandemicum' including adapting communication strategies with the public and Ministries of Health. Understanding communication/collaborations with NCBI would be helpful.

Response. We thank the reviewer for this important point. We agree that the respective roles of ICTV and NCBI Taxonomy, as well as the existing interactions between them, required clearer explanation.

ICTV is responsible for establishing the internationally agreed taxonomy and nomenclature of viruses. NCBI

Taxonomy has a different operational role, linking taxonomic identifiers to a very large number of sequence records and downstream resources. Consequently, the incorporation of ICTV changes into NCBI cannot always be implemented as a simple replacement of a name or taxon. Abolitions, splits, merges, moves and other changes must be handled in a way that preserves continuity for existing records and dependent workflows.

NCBI staff were not directly involved in developing the public ICTV ontology API described in this manuscript, and we do not claim that NCBI has committed to adopting it. The API is publicly available and could be used by NCBI or other downstream systems, but its main purpose is to provide a general, community-facing and machine-actionable access layer for current and historical ICTV taxonomy.

The ICTV database team, represented among the authors of this manuscript, has an established working relationship with the NCBI team responsible for incorporating virus taxonomy updates. This relationship and its associated processes predate the present work. The ontology API and ICTV-NCBI mappings are intended to complement these existing interactions, not to replace or disrupt them.

In parallel, ICTV is using a dedicated access mechanism for its data exchange with NCBI and plans a broader API providing access to a more comprehensive ICTV dataset. These services complement the public OLS-based API and support more specialised or higher-volume exchanges.

We made targeted revisions to the Introduction, the ICTV-NCBI mapping section and the Discussion to clarify these points.

Location in revised manuscript. Introduction; Results: ICTV–NCBI mapping; Discussion

R1.2

Comment

The taxonomy change modelling in Figure 1 was very helpful. It helped me understand the ICTV hierarchy and use of ICTV identifiers. Would it be possible to provide figures demonstrating the use of isolate and TN identifiers? It is not clear to me how those will be used.

Response. We thank the reviewer for this helpful suggestion. We clarified that TN identifiers are used as ICTV source-table keys during ontology generation. They are used to reconstruct parent-child relationships, associate VMR records with taxon nodes and interpret changes between releases, but are not embedded in the generated ontology. Public release-specific taxon states are instead represented as OWL classes with resolvable IRIs combining the MSL release and the persistent ICTV ID. We also added Figure 2 and accompanying text to show how historical and current taxon representations, replacement and revision relations, and VMR virus exemplar records are modelled as OWL named individuals and support taxon resolution.

Location in revised manuscript. Methods: Persistent identifiers and unified ontology; Figure 2; Additional file 1, Supplementary Material S1

R1.3

Comment

Figure 1 was very clear in how changes occur in the hierarchy in different ICTV releases. However, when I went to look in the ICTV Taxon Resolver, I wouldn't have understood the changes as clearly without that figure. For example, when I looked up Alphabaculovirus, it doesn't show ever having any relation to Nucleopolyhedrovirus like it does in the figure. How far back does the Taxon Resolver go? EBI-OLS suggests that the ontology was built on MSL41, so previous changes may not be modelled. Could the authors provide a little more information about this? And is there a user guide available that could provide some guidance on using the interface? I couldn't find one on the website or in the GitHub documentation, and I think it could be very helpful for those familiarizing themselves with the ICTV hierarchy and the tool.

Response. We thank the reviewer for highlighting this point. We clarified that the unified ontology contains every available release from MSL1 to MSL41. The Resolver description now states that "Show history across MSLs" starts from the OWL class representing the taxon identified from the query and displays the release-specific OWL classes linked to it across the MSL releases included in the ontology. A history may therefore begin after MSL1 or end before MSL41 if a taxon was introduced later or subsequently abolished. We also state that the relationship between Alphabaculovirus and Nucleopolyhedrovirus illustrated in Figure 1 can be traced in history mode from MSL23 back to MSL13. The goal of the default Resolver mode, "Resolve to latest ICTV", is to efficiently link to the latest version of a term. The alternative "Show history across MSLs" mode displays the linked release-specific OWL classes described above, while full histories are also available on the ICTV website.

Location in revised manuscript. Abstract–-Findings; Results: ICTV Taxon Resolver

R1.4

Comment

Is there a user guide available that could provide some guidance on using the interface? I couldn't find one on the website or in the GitHub documentation, and I think it could be very helpful for those familiarizing themselves with the ICTV hierarchy and the tool.

Response. We thank the reviewer for this useful suggestion. Concise usage guidance is displayed directly beneath the ICTV Taxon Resolver interface title, and the revised Results section draws attention to it. We also added a direct citation to the ICTV-specific API and helper-library documentation and expanded Supplementary Material S1 with direct OLS requests and a working JavaScript example.

Location in revised manuscript. Results: ICTV Taxon Resolver and developer documentation; Additional file 1, Supplementary Material S1

R1.5

Comment

The authors reference the Disease Ontology and using semantic practices prescribed by the OBO Foundry. Does the ICTV ontology use BFO/COB and RO as building blocks? It was unclear as I looked in the documentation. It also does not appear that the ICTV ontology has been submitted to the OBO Foundry, can the authors describe whether this is planned?

Response. We thank the reviewer for raising this point. We clarified the ontology's scope: it reuses selected ontology terms and annotations but does not import BFO, COB or RO. It has not been submitted to the OBO Foundry, and no submission is currently planned.

Location in revised manuscript. Methods: Ontology modelling and unified-ontology redesign

Response to Reviewer 2

We thank the reviewer for the detailed comments and respond point by point below.

R2.1

Comment

Title: consider including OLS.

Response. We considered this suggestion carefully. OLS was included in earlier versions of the title. We ultimately replaced "OLS API" with "public ontology API" to make the title immediately understandable to

readers who may not know OLS and to avoid adding another acronym to the title. ICTV and API were retained because they are central to the paper and are commonly understood across the intended audience. To make the role of OLS clearer, we added a sentence at the end of the Introduction describing its broader role in making biological and biomedical ontologies searchable, browsable and programmatically accessible through standard APIs. OLS is also identified explicitly in the Abstract and described throughout the manuscript. We therefore retained the current title.

Location in revised manuscript. Title; Abstract; Introduction

R2.2

Comment

Abstract, line 27: Replace 'framework' with 'ontology' or similar.

Response. We thank the reviewer for prompting this clarification. We replaced 'framework' in the Abstract with a precise description of the relevant components: 'Complementary ICTV–NCBI mappings and helper libraries support integration into downstream systems.' In other project-specific passages, we likewise used the component names or pointed to the previously described approach. We retained 'framework' only where it has a generic meaning, such as 'taxonomic framework', or occurs in the title of a cited source.

Location in revised manuscript. Abstract–-Findings; Key Points; Results; Figure 3; Discussion; Conclusions

R2.3

Comment

Introduction, line 105: clarify 'to make meaning human and machine readable', e.g. as 'through explicit, human- and machine-readable semantics'.

Response. We agree and revised the sentence to state that ontologies provide explicit, human- and machine-readable semantics that support the consistent annotation, sharing and interpretation of complex datasets.

Location in revised manuscript. Introduction

R2.4

Comment

Throughout the manuscript: consider replacing 'viral taxonomy' with 'virus taxonomy' and other instances.

Response. We thank the reviewer for this terminology suggestion. We reviewed the occurrences contextually and now use 'virus taxonomy' when referring to the ICTV taxonomic system, which includes taxa at all ranks. We retained scientifically appropriate uses of 'viral' where it modifies another noun, for example 'viral taxa'.

Location in revised manuscript. Abstract; main manuscript; Additional file 1

R2.5

Comment

Results, line 244: 'Mappings are maintained in a dedicated repository and updated independently.' Which GitHub repository?

Response. We thank the reviewer for identifying this omission. The revised Results section now names and

cites the dedicated EVORA virus-taxonomy-mappings repository, and the Data availability statement cites the exact mapping release archived in Zenodo together with its concept DOI for all versions.

Location in revised manuscript. Results: ICTV–NCBI mapping; Data availability; References

R2.6

Comment

Results, line 284: remove second 'pathogens'.

Response. Thank you for noting this duplication. The second occurrence of 'pathogens' was removed.

Location in revised manuscript. Results: Reference application and early adopters

R2.7

Comment

Please include links to documentation and/or tutorials for app developers apart from the supplementary material, and a plan for the dissemination of the resources.

Response. We thank the reviewer for this helpful suggestion. The revised Results section now cites the public ICTV-specific developer documentation and helper libraries, and Supplementary Material S1 provides detailed API and helper-library examples. The Availability and sustainability section now describes dissemination and discoverability through OLS, versioned GitHub and Zenodo releases, and registration of the software resources in bio.tools and SciCrunch.

Location in revised manuscript. Results: API/helper-library documentation; Methods: Availability and sustainability; Availability of supporting source code and requirements; Additional file 1, Supplementary Material S1

R2.8

Comment

Discussion, line 310: Temper 'much easier to access' and focus on the improved technical capability.

Response. We agree and replaced the subjective phrase 'much easier to access' with a specific description of the improved technical capability: downstream systems now have programmatic access to current and historical taxonomic information and can resolve former terms to the current classification, supporting uses from direct queries to recurring automated updates.

Location in revised manuscript. Discussion

R2.9

Comment

Throughout the manuscript: the wording 'alignment with FAIR principles' or 'FAIR-aligned' is sufficient; FAIR-compliant is not necessary as there is not a defined compliance standard.

Response. We agree. We revised the manuscript to use 'FAIR-aligned' or similarly qualified wording rather than claiming full FAIR compliance.

Location in revised manuscript. Discussion; Conclusions; Additional file 1

R2.10

Comment

Figure 1: consider changing the red color to color-blind-friendly magenta.

Response. We thank the reviewer for this accessibility suggestion. The red encoding in Figure 1 was replaced with the colour-blind-safe dark magenta #AA3377 (RGB 170, 51, 119) from Paul Tol's bright qualitative colour scheme.

Location in revised manuscript. Figure 1

R2.11

Comment

Supplementary Material S1: please add a concrete example and screenshot of result in tutorial style.

Response. Supplementary Material S1 now provides a worked example for resolving a former name using direct OLS API requests, identifies the replacement relation followed to the current OWL class, and gives the equivalent JavaScript helper-library example. The corresponding completed Resolver example is shown in the new Figure 4, providing a visual result without duplicating the same material in the supplement.

Location in revised manuscript. Results: ICTV Taxon Resolver; Figure 4; Additional file 1, Supplementary Material S1

Closing statement

We thank the Editor and Reviewers again for their constructive assessment. We believe that the revisions improve the clarity, reproducibility and usability of the manuscript and its associated resources.

Editorial Manager®

Cookie Preference Center

We use cookies which are necessary to make our site work. We may also use additional cookies to analyze, improve and personalize our content and your digital experience. For more information, see our Cookie Policy.

You may choose not to allow some types of cookies. However, blocking some types may impact your experience of our site and the services we are able to offer. See the different category headings below to find out more or change your settings.

Allow all

Manage Consent Preferences

Strictly Necessary Cookies

Always active

These cookies are necessary for the website to function and cannot be switched off in our systems. They are usually only set in response to actions made by you which amount to a request for services, such as setting your privacy preferences, logging in or filling in forms. You can set your browser to block or alert you about these cookies, but some parts of the site will not then work.

Functional Cookies

These cookies enable the website to provide enhanced functionality and personalisation. They may be set by us or by third party providers whose services we have added to our pages. If you do not allow these cookies then some or all of these services may not function properly.

Performance Cookies

These cookies allow us to count visits and traffic sources so we can measure and improve the performance of our site. They help us to know which pages are the most and least popular and see how visitors move around the site.

Targeting Cookies

These cookies may be set through our site by our advertising partners. They may be used by those companies to build a profile of your interests and show you relevant adverts on other sites. If you do not allow these cookies, you will experience less targeted advertising.

Cookie List

Clear

Apply Cancel

Consent Leg.Interest

Confirm my choices

Power
