# Supplementary material for: Programmatic access to ICTV virus taxonomy through a public ontology API
Source: Gigascience. 2026 Sep 4;15:giag089. doi: 10.1093/gigascience/giag089 (PMC13615544; doi:10.1093/gigascience/giag089)
Supplement: Supplementary file 3 [file giag089_giga-d-26-00217_revision_1.pdf]

GigaScience

Programmatic access to ICTV virus taxonomy through a public ontology API

--Manuscript Draft--

**Manuscript Number:**	GIGA-D-26-00217R1
**Full Title:**	Programmatic access to ICTV virus taxonomy through a public ontology API
**Article Type:**	Technical Note
**Funding Information:**	HORIZON EUROPE Framework Programme (101131959) — Mr. Philippe Lieutaud, Dr. James McLaughlin, Dr. Romain David, Dr. Helen Parkinson, Dr. Bruno Coutard<br>Division of Microbiology and Infectious Diseases, National Institute of Allergy and Infectious Diseases (U24AI162625) — Dr. R. Curtis Hendrickson, Dr. Elliot J Lefkowitz, Mr. Donald M Dempsey
**Abstract:**	BackgroundThe International Committee on Taxonomy of Viruses (ICTV) is responsible for developing and maintaining a universal virus taxonomy. As the reference framework for organising the viral world, it is essential for virology and related fields. Despite its widespread use in research and public health, programmatic access to ICTV taxonomy has remained limited, posing challenges for integration, versioning, and interoperability across databases and bioinformatics resources requiring up-to-date virus taxonomy. FindingsTo address this, we developed a public and sustainable solution leveraging ontology-based APIs. All available ICTV Master Species List (MSL) releases, from MSL1 to MSL41, were transformed into a unified, semantically structured ontology comprising more than 195,000 current and historical entities and deployed through the Ontology Lookup Service (OLS). The ontology is automatically rebuilt and republished whenever a new MSL release becomes available. Complementary ICTV-NCBI mappings and helper libraries support integration into downstream systems.ConclusionsTogether, these resources enable, for the first time, public programmatic retrieval of current and historical ICTV taxon names, taxonomic relationships, metadata, and persistent identifiers through stable endpoints, including resolution of former taxonomic terms to their current accepted taxon or taxa and retrieval of taxon histories across releases. More broadly, this work illustrates a general strategy for transforming structured biological datasets into semantically enriched graph resources exposed through scalable public APIs. These developments enhance interoperability, reduce manual curation, and support FAIR-aligned taxonomic data management in virology and pandemic preparedness.
**Corresponding Author:**	Philippe Lieutaud<br>Aix-Marseille-University: Aix-Marseille Universite<br>Marseille, FRANCE
**Corresponding Author Secondary Information:**	
**Corresponding Author's Institution:**	Aix-Marseille-University: Aix-Marseille Universite
**Corresponding Author's Secondary Institution:**	
**First Author:**	Philippe Lieutaud
**First Author Secondary Information:**	
**Order of Authors:**	Philippe Lieutaud<br>James McLaughlin<br>R. Curtis Hendrickson<br>Romain David<br>Helen Parkinson

Powered by Editorial Manager® and ProduXion Manager® from Aries Systems Corporation

	Elliot J Lefkowitz
	Donald M Dempsey
	Bruno Coutard
**Order of Authors Secondary Information:**	

Response to Reviewers:

Dear Editor and Reviewers,

We thank the Editor and Reviewers for their careful and constructive assessment. We revised the manuscript and supporting resources accordingly. The comments concerned clarification, documentation and presentation; no new scientific data or analyses were required. The responses below address the reviewer comments point by point, and all substantive revisions are described below and incorporated in the revised manuscript.

Summary of principal revisions

- Clarified complete MSL1–MSL41 coverage, the public release-specific IRI design and the source-only role of TN identifiers.

- Added Figure 2 and accompanying text explaining historical and current taxon resolution, PROV and replacement relations, and VMR isolates as OWL NamedIndividuals linked to species OWL classes.

- Clarified the complementary roles of ICTV, NCBI Taxonomy, the public ontology API and the ICTV–NCBI mappings; named and archived the mapping resource.

- Expanded direct API, helper-library and Taxon Resolver documentation, including worked examples in Supplementary Material S1 and the Figure 4 result screenshot.

- Applied terminology, accessibility and presentation revisions, including context-sensitive use of 'virus taxonomy', qualified FAIR language and the colour-blind-safe Figure 1 palette.

Location note. Section locations are provided below and correspond to the clean, line-numbered revised manuscript.

Response to Reviewer 1

We thank the reviewer for the detailed comments and respond point by point below.

R1.1

Comment

Have NCBI been included in conversations about these developments? It seems strange that representatives from EMBL-EBI are authors but there is no inclusion of partners from NCBI when NCBITaxon is a key resource for many platforms, application ontologies, and tools. Could the authors mention engagement efforts with NCBI so we understand whether the ICTV IDs and synonymy will be adopted? I think this is important as well as the authors pointedly describe the challenges of updating taxonomy at NCBI, but don't note that major consumers/providers of data are public health agencies. Every change in NCBITaxon breaks critical workflows in systems used in routine public health operations. NCBI is caught between a rock and a hard place every time ICTV makes changes - wanting to respect expert recommendations while managing pushback from the community e.g. there were/are many challenges of adopting the change of 'Severe acute respiratory syndrome-related coronavirus' to 'Betacoronavirus pandemicum' including adapting communication strategies with the public and Ministries of Health. Understanding communication/collaborations with NCBI would be helpful.

Response. We thank the reviewer for this important point. We agree that the respective roles of ICTV and NCBI Taxonomy, as well as the existing interactions between them, required clearer explanation.

ICTV is responsible for establishing the internationally agreed taxonomy and nomenclature of viruses. NCBI Taxonomy has a different operational role, linking taxonomic identifiers to a very large number of sequence records and downstream resources. Consequently, the incorporation of ICTV changes into NCBI cannot always be implemented as a simple replacement of a name or taxon. Abolitions, splits, merges, moves and other changes must be handled in a way that preserves continuity for existing records and dependent workflows.

NCBI staff were not directly involved in developing the public ICTV ontology API described in this manuscript, and we do not claim that NCBI has committed to adopting it. The API is publicly available and could be used by NCBI or other downstream systems, but its main purpose is to provide a general, community-facing and machine-actionable access layer for current and historical ICTV taxonomy.

The ICTV database team, represented among the authors of this manuscript, has an established working relationship with the NCBI team responsible for incorporating virus taxonomy updates. This relationship and its associated processes predate the present work. The ontology API and ICTV-NCBI mappings are intended to complement these existing interactions, not to replace or disrupt them.

In parallel, ICTV is using a dedicated access mechanism for its data exchange with NCBI and plans a broader API providing access to a more comprehensive ICTV dataset. These services complement the public OLS-based API and support more specialised or higher-volume exchanges.

We made targeted revisions to the Introduction, the ICTV-NCBI mapping section and the Discussion to clarify these points.

Location in revised manuscript. Introduction; Results: ICTV–NCBI mapping; Discussion

R1.2

Comment

The taxonomy change modelling in Figure 1 was very helpful. It helped me understand the ICTV hierarchy and use of ICTV identifiers. Would it be possible to provide figures demonstrating the use of isolate and TN identifiers? It is not clear to me how those will be used.

Response. We thank the reviewer for this helpful suggestion. We clarified that TN identifiers are used as ICTV source-table keys during ontology generation. They are used to reconstruct parent-child relationships, associate VMR records with taxon nodes and interpret changes between releases, but are not embedded in the generated ontology. Public release-specific taxon states are instead represented as OWL classes with resolvable IRIs combining the MSL release and the persistent ICTV ID. We also added Figure 2 and accompanying text to show how historical and current taxon representations, replacement and revision relations, and VMR virus exemplar records are modelled as OWL named individuals and support taxon resolution.

Location in revised manuscript. Methods: Persistent identifiers and unified ontology; Figure 2; Additional file 1, Supplementary Material S1

R1.3

Comment

Figure 1 was very clear in how changes occur in the hierarchy in different ICTV releases. However, when I went to look in the ICTV Taxon Resolver, I wouldn't have understood the changes as clearly without that figure. For example, when I looked up Alphabaculovirus, it doesn't show ever having any relation to Nucleopolyhedrovirus like it does in the figure. How far back does the Taxon Resolver go? EBI-OLS suggests that the ontology was built on MSL41, so previous changes may not be modelled. Could the authors provide a little more information about this? And is there a user

guide available that could provide some guidance on using the interface? I couldn't find one on the website or in the GitHub documentation, and I think it could be very helpful for those familiarizing themselves with the ICTV hierarchy and the tool.

Response. We thank the reviewer for highlighting this point. We clarified that the unified ontology contains every available release from MSL1 to MSL41. The Resolver description now states that "Show history across MSLs" starts from the OWL class representing the taxon identified from the query and displays the release-specific OWL classes linked to it across the MSL releases included in the ontology. A history may therefore begin after MSL1 or end before MSL41 if a taxon was introduced later or subsequently abolished. We also state that the relationship between Alphabaculovirus and Nucleopolyhedrovirus illustrated in Figure 1 can be traced in history mode from MSL23 back to MSL13. The goal of the default Resolver mode, "Resolve to latest ICTV", is to efficiently link to the latest version of a term. The alternative "Show history across MSLs" mode displays the linked release-specific OWL classes described above, while full histories are also available on the ICTV website.

Location in revised manuscript. Abstract–-Findings; Results: ICTV Taxon Resolver

R1.4

Comment

Is there a user guide available that could provide some guidance on using the interface? I couldn't find one on the website or in the GitHub documentation, and I think it could be very helpful for those familiarizing themselves with the ICTV hierarchy and the tool.

Response. We thank the reviewer for this useful suggestion. Concise usage guidance is displayed directly beneath the ICTV Taxon Resolver interface title, and the revised Results section draws attention to it. We also added a direct citation to the ICTV-specific API and helper-library documentation and expanded Supplementary Material S1 with direct OLS requests and a working JavaScript example.

Location in revised manuscript. Results: ICTV Taxon Resolver and developer documentation; Additional file 1, Supplementary Material S1

R1.5

Comment

The authors reference the Disease Ontology and using semantic practices prescribed by the OBO Foundry. Does the ICTV ontology use BFO/COB and RO as building blocks? It was unclear as I looked in the documentation. It also does not appear that the ICTV ontology has been submitted to the OBO Foundry, can the authors describe whether this is planned?

Response. We thank the reviewer for raising this point. We clarified the ontology's scope: it reuses selected ontology terms and annotations but does not import BFO, COB or RO. It has not been submitted to the OBO Foundry, and no submission is currently planned.

Location in revised manuscript. Methods: Ontology modelling and unified-ontology redesign

Response to Reviewer 2

We thank the reviewer for the detailed comments and respond point by point below.

R2.1

Comment

Title: consider including OLS.

Powered by Editorial Manager® and ProduXion Manager® from Aries Systems Corporation

Response. We considered this suggestion carefully. OLS was included in earlier versions of the title. We ultimately replaced "OLS API" with "public ontology API" to make the title immediately understandable to readers who may not know OLS and to avoid adding another acronym to the title. ICTV and API were retained because they are central to the paper and are commonly understood across the intended audience. To make the role of OLS clearer, we added a sentence at the end of the Introduction describing its broader role in making biological and biomedical ontologies searchable, browsable and programmatically accessible through standard APIs. OLS is also identified explicitly in the Abstract and described throughout the manuscript. We therefore retained the current title.

Location in revised manuscript. Title; Abstract; Introduction

R2.2

Comment

Abstract, line 27: Replace 'framework' with 'ontology' or similar.

Response. We thank the reviewer for prompting this clarification. We replaced 'framework' in the Abstract with a precise description of the relevant components: 'Complementary ICTV–NCBI mappings and helper libraries support integration into downstream systems.' In other project-specific passages, we likewise used the component names or pointed to the previously described approach. We retained 'framework' only where it has a generic meaning, such as 'taxonomic framework', or occurs in the title of a cited source.

Location in revised manuscript. Abstract–-Findings; Key Points; Results; Figure 3; Discussion; Conclusions

R2.3

Comment

Introduction, line 105: clarify 'to make meaning human and machine readable', e.g. as 'through explicit, human- and machine-readable semantics'.

Response. We agree and revised the sentence to state that ontologies provide explicit, human- and machine-readable semantics that support the consistent annotation, sharing and interpretation of complex datasets.

Location in revised manuscript. Introduction

R2.4

Comment

Throughout the manuscript: consider replacing 'viral taxonomy' with 'virus taxonomy' and other instances.

Response. We thank the reviewer for this terminology suggestion. We reviewed the occurrences contextually and now use 'virus taxonomy' when referring to the ICTV taxonomic system, which includes taxa at all ranks. We retained scientifically appropriate uses of 'viral' where it modifies another noun, for example 'viral taxa'.

Location in revised manuscript. Abstract; main manuscript; Additional file 1

R2.5

Comment

Results, line 244: 'Mappings are maintained in a dedicated repository and updated independently.' Which GitHub repository?

Response. We thank the reviewer for identifying this omission. The revised Results section now names and cites the dedicated EVORA virus-taxonomy-mappings repository, and the Data availability statement cites the exact mapping release archived in Zenodo together with its concept DOI for all versions.

Location in revised manuscript. Results: ICTV–NCBI mapping; Data availability; References

R2.6

Comment

Results, line 284: remove second 'pathogens'.

Response. Thank you for noting this duplication. The second occurrence of 'pathogens' was removed.

Location in revised manuscript. Results: Reference application and early adopters

R2.7

Comment

Please include links to documentation and/or tutorials for app developers apart from the supplementary material, and a plan for the dissemination of the resources.

Response. We thank the reviewer for this helpful suggestion. The revised Results section now cites the public ICTV-specific developer documentation and helper libraries, and Supplementary Material S1 provides detailed API and helper-library examples. The Availability and sustainability section now describes dissemination and discoverability through OLS, versioned GitHub and Zenodo releases, and registration of the software resources in bio.tools and SciCrunch.

Location in revised manuscript. Results: API/helper-library documentation; Methods: Availability and sustainability; Availability of supporting source code and requirements; Additional file 1, Supplementary Material S1

R2.8

Comment

Discussion, line 310: Temper 'much easier to access' and focus on the improved technical capability.

Response. We agree and replaced the subjective phrase 'much easier to access' with a specific description of the improved technical capability: downstream systems now have programmatic access to current and historical taxonomic information and can resolve former terms to the current classification, supporting uses from direct queries to recurring automated updates.

Location in revised manuscript. Discussion

R2.9

Comment

Throughout the manuscript: the wording 'alignment with FAIR principles' or 'FAIR-aligned' is sufficient; FAIR-compliant is not necessary as there is not a defined compliance standard.

Response. We agree. We revised the manuscript to use 'FAIR-aligned' or similarly qualified wording rather than claiming full FAIR compliance.

Powered by Editorial Manager® and ProduXion Manager® from Aries Systems Corporation

	Location in revised manuscript. Discussion; Conclusions; Additional file 1<br><br>R2.10<br><br>Comment<br><br>Figure 1: consider changing the red color to color-blind-friendly magenta.<br><br>Response. We thank the reviewer for this accessibility suggestion. The red encoding in Figure 1 was replaced with the colour-blind-safe dark magenta #AA3377 (RGB 170, 51, 119) from Paul Tol's bright qualitative colour scheme.<br><br>Location in revised manuscript. Figure 1<br><br>R2.11<br><br>Comment<br><br>Supplementary Material S1: please add a concrete example and screenshot of result in tutorial style.<br><br>Response. Supplementary Material S1 now provides a worked example for resolving a former name using direct OLS API requests, identifies the replacement relation followed to the current OWL class, and gives the equivalent JavaScript helper-library example. The corresponding completed Resolver example is shown in the new Figure 4, providing a visual result without duplicating the same material in the supplement.<br><br>Location in revised manuscript. Results: ICTV Taxon Resolver; Figure 4; Additional file 1, Supplementary Material S1<br><br>Closing statement<br><br>We thank the Editor and Reviewers again for their constructive assessment. We believe that the revisions improve the clarity, reproducibility and usability of the manuscript and its associated resources.
**Additional Information:**	
**Question**	**Response**
Are you submitting this manuscript to a special series or article collection?	No
**Experimental design and statistics**<br><br>Full details of the experimental design and statistical methods used should be given in the Methods section, as detailed in our Minimum Standards Reporting Checklist. Information essential to interpreting the data presented should be made available in the figure legends.<br><br>Have you included all the information requested in your manuscript?	Yes
**Resources**	Yes

A description of all resources used, including antibodies, cell lines, animals and software tools, with enough information to allow them to be uniquely identified, should be included in the Methods section. Authors are strongly encouraged to cite Research Resource Identifiers (RRIDs) for antibodies, model organisms and tools, where possible.<br><br>Have you included the information requested as detailed in our Minimum Standards Reporting Checklist?	
**Availability of data and materials**<br><br>All datasets and code on which the conclusions of the paper rely must be either included in your submission or deposited in publicly available repositories (where available and ethically appropriate), referencing such data using a unique identifier in the references and in the “Availability of Data and Materials” section of your manuscript.<br><br>Have you have met the above requirement as detailed in our Minimum Standards Reporting Checklist?	Yes
GigaScience has policies and guidelines in place for the use of generative AI-writing tools such as ChatGPT. If you have used such writing tools to assist with writing the manuscript this must be declared and cited in the text. Authors should not list AI-writing tools and other AI-assisted technologies as an author or co-author and should acknowledge that they are fully responsible for text generated or refined by AI-writing tools.<p><br><br>A summary of use (particularly in the introduction or among methods) needs to be included at the end of the paper, and the outputs should also be included as a supplementary file hosted in GigaDB or other open repositories. Please read our guidelines for more information. <p>	Yes

Powered by Editorial Manager® and ProduXion Manager® from Aries Systems Corporation

By submitting to GigaScience, you are aware of the journal's AI-writing tools policy, and if you have declared use of such tools below, you have acknowledged this where appropriate in your manuscript and have made a summary of use and outputs available. <p> <b>AI-assisted writing tools have been used in the preparation of this manuscript?	

Main manuscript TeX

Click here to access/download;LaTeX - Main Document (TeX file);main.tex

GigaScience, 2026, 1–14

doi: xx.xxxx/xxxx
Manuscript in Preparation
Technical Note

TECHNICAL NOTE

Programmatic access to ICTV virus taxonomy through a public ontology API

Philippe Lieutaud[1,*], James McLaughlin[2,*], R. Curtis Hendrickson[3,*], Romain David[4,*], Helen Parkinson[2,*], Elliot J. Lefkowitz[3,*], Donald M. Dempsey[3,*] and Bruno Coutard[1,*]

[1]European Viral Outbreak Response Alliance (EVORA); unité des virus émergents - UVE - UMR 190 - Aix Marseille Université, IRD 190, INSERM U1207, France and [2]European Bioinformatics Institute (EMBL-EBI), European Molecular Biology Laboratory, Wellcome Trust Genome Campus, Hinxton, Cambridge CB10 1SD, United Kingdom and [3]International Committee on the Taxonomy of Viruses (ICTV); University of Alabama at Birmingham, Department of Microbiology, Birmingham, Alabama 35294, United States and [4]European Research Infrastructure on Highly Pathogenic Agents (ERINHA AISBL), 98 rue du Trône, B-1050 Bruxelles, Belgium

[*]Corresponding author: Philippe.Lieutaud@univ-amu.fr. Author emails: Philippe.Lieutaud@univ-amu.fr; jmcl@ebi.ac.uk; curtish@uab.edu; romain.david@erinha.eu; parkinso@ebi.ac.uk; elliotl@uab.edu; ddempsey@uab.edu; bruno.coutard@univ-amu.fr.

Abstract

Background
The International Committee on Taxonomy of Viruses (ICTV) is responsible for developing and maintaining a universal virus taxonomy. As the reference framework for organising the viral world, it is essential for virology and related fields. Despite its widespread use in research and public health, programmatic access to ICTV taxonomy has remained limited, posing challenges for integration, versioning, and interoperability across databases and bioinformatics resources requiring up-to-date virus taxonomy.

Findings
To address this, we developed a public and sustainable solution leveraging ontology-based APIs. All available ICTV Master Species List (MSL) releases, from MSL1 to MSL41, were transformed into a unified, semantically structured ontology comprising more than 195,000 current and historical entities and deployed through the Ontology Lookup Service (OLS). The ontology is automatically rebuilt and republished whenever a new MSL release becomes available. Complementary ICTV-NCBI mappings and helper libraries support integration into downstream systems.

Conclusions
Together, these resources enable, for the first time, public programmatic retrieval of current and historical ICTV taxon names, taxonomic relationships, metadata, and persistent identifiers through stable endpoints, including resolution of former taxonomic terms to their current accepted taxon or taxa and retrieval of taxon histories across releases. More broadly, this work illustrates a general strategy for transforming structured biological datasets into semantically enriched graph resources exposed through scalable public APIs. These developments enhance interoperability, reduce manual curation, and support FAIR-aligned taxonomic data management in virology and pandemic preparedness.

Key words: ICTV; Virus taxonomy; Ontology; API; FAIR principles; Interoperability

Introduction and landscape

The International Committee on Taxonomy of Viruses (ICTV) [1], established under the Virology Division of the International Union

Compiled on: August 25, 2026.
Draft manuscript prepared by the author.

Key Points

- ICTV provides the official taxonomy for classifying viruses and naming virus taxa, but lacks standardised programmatic access.
- Transforming ICTV data into an ontology enables semantic, machine-actionable access across releases via ontology-based APIs.
- ICTV-NCBI mappings support interoperability across bioinformatics resources.
- The integrated ontology-based resource enables programmatic resolution of current and historical viral taxa.
- This approach provides a reusable model for exposing biological datasets through public APIs.

of Microbiological Societies (IUMS) [2], is the authoritative body responsible for the classification of viruses and naming of virus taxa, ensuring a standardised and commonly accepted nomenclature. Virus taxonomy provides a structured framework for classifying viruses based on evolutionary relationships, shared biological properties, and genomic features, such as genetic material, structural features, replication strategies and host range. While everyday virus names function as vernacular labels that can vary across studies and contexts, the ICTV taxon names provide formal, standardised designations that ensure unambiguous communication across the scientific community [3].

The regularly updated ICTV Master Species Lists (MSLs) [4] reflect ongoing advances in virology, including classification of newly discovered viruses and refined taxonomic insights, with one major new release each year, sometimes complemented by minor releases [5]. As virus discovery accelerates, particularly through metagenomics, maintaining consistent and up-to-date taxonomic references in databases and bioinformatics tools relying on ICTV taxa becomes increasingly important.

While the ICTV releases provide authoritative reference data, their distribution format is primarily designed for human consultation and does not readily support programmatic access [6]. As a result, integrating ICTV taxonomy updates into third-party computational systems remains challenging. Broader taxonomic platforms such as NCBI Taxonomy (National Center for Biotechnology Information) [7], GBIF (Global Biodiversity Information Facility) [8], COL (Catalogue of Life) [9] and Wikidata [10] embed ICTV releases as the authoritative source for virus taxonomy. However, these platforms often incorporate ICTV updates with substantial delays and may lack systematic support for resolving historical taxonomic changes.

Downstream resources can lag by one or more ICTV taxonomic updates, resulting in discrepancies in the classification they expose. Such inconsistencies create significant challenges for users who require the most up-to-date virus classifications, potentially affecting research accuracy and data consistency. For example, outdated species names may persist for some time in major sequence repositories such as GenBank [11] and the European Nucleotide Archive (ENA) [12], leading to further propagation of downstream inconsistencies. Since these repositories rely on the NCBI Taxonomy as their primary taxonomic reference, such delays can be observed by comparing historical taxonomic archives of NCBI and ICTV. A concrete example is ICTV renaming "*Severe acute respiratory syndrome-related coronavirus*" to "*Betacoronavirus pandemicum*" in MSL39. This change did not appear in NCBI Taxonomy for more than a year, and, when it did, was represented by the creation of a new NCBI taxon, rather than a direct renaming of the existing taxa. Obsolete taxon names that continue to be treated as current references complicate reproducibility and hinder interoperability across resources. These problems are inevitable, given that NCBI is not simply embedding ICTV taxonomy, but integrating it within a broader, sequence-linked system whose update process must preserve continuity for existing records and dependent workflows. As such, ICTV remains the authoritative source for virus taxonomy, while NCBI serves as a widely used integration layer. Consequently, taxon abolitions, splits, merges, moves, and renames may require specific handling rather than simple replacement. Such cases illustrate why programmatic resolution mechanisms are needed [13], along with traceable ICTV identifiers.

The European Viral Outbreak Response Alliance (EVORA) project [14] is a European initiative (2024-2026; grant No. 101131959) [15] that aims to enhance pandemic preparedness and response by strengthening research coordination, regulatory alignment, and data sharing among EU Research Infrastructures. Improving universal adoption of accurate and up-to-date virus taxonomy represents an important component of this mission. Members of EVORA and ICTV collaborated to provide a structured, machine-actionable version of the official ICTV taxonomy using ontology-based APIs, with support for historical name resolution, revision tracking and cross-system identifiers.

Many biological data resources are distributed as relational databases, spreadsheets or tabular datasets that are well suited for storage, but less adapted to programmatic integration across heterogeneous systems. In addition to addressing the immediate need for improved access to ICTV taxonomy, this work illustrates a broader methodological approach for transforming structured database content into semantically enriched knowledge representations through the use of ontologies. Ontologies play a crucial role in the management, integration, and analysis of biological data by providing explicit, human- and machine-readable semantics that support the consistent annotation, sharing and interpretation of complex datasets [16]. Converting ICTV releases into an ontology enables graph-based querying and integration. As graph-based representations naturally capture hierarchical relationships, synonyms, and historical changes, representing taxonomy as an ontology therefore enables more expressive queries, automated reasoning, and easier integration with other semantic resources.

Across the life sciences, the Ontology Lookup Service (OLS) provides widely used infrastructure for searching, browsing and accessing biological and biomedical ontologies through standard APIs, supporting the reuse of ontology terms and relations in data curation and computational workflows [17, 18].

Methods and strategy

In this paper, "OLS API" refers to the generic programmatic interface provided by the OLS, including its Representational State Transfer (REST) endpoints and controllers. "ICTV ontology API" refers to access to the ICTV ontology through the OLS API, either directly via OLS endpoints or indirectly through ICTV-specific helper libraries built on top of them.

Design requirements and use cases

To support practical integration of ICTV taxonomy into data systems and analytical workflows, several key use cases were defined to address common end-user challenges for automated processing. First, it must provide historical resolution by mapping obsolete names, synonyms, and ICTV identifiers from previous ICTV releases to their corresponding taxa in the current ICTV classification. Second, taxonomic changes must be tracked across releases, including taxa

that have been abolished, split, merged, renamed, or reassigned (promoted) within the hierarchy. Third, cross-resource mapping must be supported, and, specifically, a mapping, with provenance, between NCBI Taxonomy identifiers and ICTV identifiers (historical and current) must be provided. Finally, there must be a stable, public, well-documented API for performing these functions.

Persistent identifiers

Persistent identifiers are key for data integration [19]. The ICTV defines persistent identifiers for all versions of all taxa, as well as links connecting the taxa into hierarchies and documenting changes between releases. These unique identifiers facilitate resolution of outdated taxonomic terms to current ones by allowing taxa to be efficiently tracked across renames, rank changes, merges, splits, and abolitions between releases. To support programmatic resolution and cross-release linkage, and to further support FAIR principles, ICTV publicly documented these existing identifiers and defined Compact Uniform Resource Identifiers (CURIEs) for them. ICTV provides three principal identifiers. In the shorthand used below, the letter prefix indicates the identifier type and # represents the numeric identifier value:

- Taxnode identifier (Taxnode ID; TN#): uniquely identifies a taxon node within the ICTV source database in a specific release.
- ICTV identifier (ICTV ID; ICTV#): identifies the same taxon across releases, including through renames, changes in location within the taxonomic tree (moves), and changes in rank (promotion/demotion). Splits and merges create new ICTV IDs, which are linked back to their antecedent taxa.
- Isolate identifier (Isolate ID; VMR#): defined by the Virus Metadata Resource (VMR) [20], links GenBank sequence accessions for one or more segments to the vernacular name and the corresponding viral species [6].

During ontology generation, TN identifiers are used as ICTV source-table keys to reconstruct parent–child relationships within each release, associate VMR records with taxon nodes and interpret changes between releases. They are not included in the ontology because their role is limited to processing the ICTV source data. Instead, each release-specific taxon state is represented as an OWL class and is assigned a resolvable IRI combining the MSL release and the persistent ICTV ID (for example, `http://ictv.global/id/MSL41/ICTV20040588`). This design uniquely identifies each release-specific taxon state, preserves direct resolution across simple renames and uses explicit revision and replacement relations when taxonomic events create new ICTV IDs.

This identifier design underpins the modelling of ICTV releases as semantically linked ontology resources. An extremely abridged example of the history of a taxon related to the genus *Baculovirus* in Figure 1 illustrates some of the complexity, and demonstrates how ICTV IDs link historic versions of a taxon, from the first ICTV release to the current MSL41 release [21].

Initial modelling of ICTV releases as ontologies

To meet the design requirements and use cases described above, including historical resolution, cross-release tracking and programmatic querying, each of the 41 (as of 2026) official ICTV taxonomy updates since 1971 was modelled as an independent OWL2 ontology, representing hierarchies, ranks, synonyms and revision metadata. Taxa were modelled as OWL classes and virus isolates as OWL named individuals. This approach captures taxonomic relationships and their evolution over time, while preserving the structural and semantic fidelity of the original ICTV data in a machine-readable format.

The ontologies were constructed using established semantic standards and interoperable vocabularies commonly used in biomedical ontology ecosystems (e.g. [22], built upon semantic web standards including OWL, RDFS, PROV-O [23] and SKOS semantic relations [24], together with complementary vocabularies such as Dublin Core Terms (DCTERMS), Friend Of A Friend (FOAF), the Taxonomic Rank Vocabulary (TAXRANK) and oboInOwl ontology annotations. This supports compatibility with the broader life-science ontology ecosystem [25] and reuses selected terms and annotations from OBO-associated resources [26]. Where a replacement exists, deprecated historical OWL classes are linked to their replacements using IAO:0100001 (term replaced by), enabling rapid resolution of former terms to their current representations.

The OLS enables direct exposure of ontology data through stable REST API controllers, a web-based user interface [27], and a Model Context Protocol (MCP) server for access by AI agents. OLS provides a ready-to-use infrastructure to host ontologies and deliver programmatic access through standardised endpoints [17]. This approach eliminates the need to develop and maintain custom API infrastructures while ensuring compatibility with the broader ecosystem of life science ontologies. Thus, transforming existing data into a semantically structured graph representation leverages the OLS infrastructure to efficiently provide standard, public APIs and interoperability across data infrastructures.

The set of release-specific ontologies was published via OLS. The OLS API enables dynamic querying, retrieval of data and linking across ontologies, allowing applications to access up-to-date information directly from ICTV data via the ontology stored in OLS.

Redesign as a unified ontology

However, querying multiple ontologies proved complex for the primary use case of reconciling historical references with the current taxonomy. The system was therefore redesigned to merge all releases into a single, unified ontology (over 195,000 terms in 2026). In this model, the most recent ICTV release represents the authoritative classification, while entities from previous releases are preserved as deprecated historical OWL classes and, where applicable, linked through explicit replacement relations. This design reduces query complexity, while preserving full history, provenance and backward compatibility across ICTV releases. This enables any taxonomic reference appearing in historical datasets or publications to be programmatically reconciled with the current ICTV classification.

The ontology is primarily functional: it was engineered to expose current and historical ICTV taxonomy through OLS and the helper libraries so that taxon identity, lineage, synonyms and revision history can be queried programmatically. It does not import BFO, COB or RO, has not been submitted to the OBO Foundry, and no submission is currently planned.

Figure 2 illustrates how the entities and relations in the unified ontology support taxon resolution across ICTV releases. Release-specific taxon representations, modelled as OWL classes, are connected through PROV revision relations and, when a replacement is defined, through IAO:0100001 to the corresponding OWL class in the latest MSL, which represents the currently accepted ICTV taxon. In simple taxon rename paths, reusing the stable ICTV ID with the latest MSL release also retrieves the current OWL class, whereas IAO:0100001 provides a more direct route to the replacement OWL class and can resolve replacement or merge events without traversing the full release history. VMR virus exemplar records modelled as OWL named individuals are linked by rdf:type to their corresponding species OWL classes in the ontology. Together, these relations allow identifiers and current or former taxon or virus names to be resolved to current taxa, to defined historical states or to complete taxon histories through the OLS API and helper libraries.

Availability and sustainability

Continuous Integration and Deployment (CI/CD) workflows implemented through public GitHub repositories automatically trigger the ontology generation workflow for each release published in the ICTV's official GitHub repository [28]. This automation removes manual intervention and contributes to long-term sustainability of the infrastructure, in line with recommendations for sustainable FAIR implementation in Life Sciences [29]. The unified ontology is immediately available as a release from the EVORA GitHub repository [30], and is automatically deployed to the OLS, typically within 24 hours of an ICTV release, which provides public web browsing and API access. Dissemination and long-term discoverability are further supported through versioned GitHub and Zenodo releases, as well as registration of the software resources in bio.tools and SciCrunch (see Availability of supporting source code and requirements section).

ICTV-NCBI mapping

To improve interoperability at the broader scale [31], ICTV-NCBI mappings are generated using the OLS API, and stored in SSSOM (Simple Standard for Sharing Ontology Mapping) format, aligning ICTV identifiers with NCBI Taxon identifiers [32]. The code to generate this mapping is maintained in a dedicated GitHub repository [33], where the resulting SSSOM files are then published as releases (see Additional file 1, Supplementary Material S4). While this analysis is also automated using a GitHub CI/CD workflow, it runs on a schedule, decoupled from the release cycles of the two ontologies, allowing independent updates while remaining consistent with the continuously updated ontology infrastructure.

These mappings not only facilitate direct and bidirectional linkage between ICTV and NCBI taxonomies, but also enable alignment across downstream resources that rely on NCBI as a taxonomic backbone, including sequence repositories and bioinformatics platforms. This feature enables broader platforms that rely on the NCBI taxonomy for viral references (e.g., ENA) to stay current with the ICTV taxonomy while continuing to leverage the broader coverage of NCBI taxonomy.

The NCBI integrates the ICTV Taxonomy into its own taxonomy system, which is used to designate species related to the entries in its sequence databases (e.g. GenBank). This integration is supported by an established exchange process between the ICTV database team and the NCBI team responsible for virus taxonomy updates. This process predates the present work and accommodates NCBI's specific sequence-linked update requirements. The public ICTV ontology API and ICTV-NCBI mappings complement this process by providing a general, community-facing and machine-actionable layer for accessing and reconciling current and historical ICTV taxonomy. Historically, ICTV updates have been incorporated with roughly a one-year delay, resulting in NCBI being one or two releases behind the latest ICTV version (as described above). Observations following MSL41 release suggest a recent effort by NCBI to shorten this delay, with progressive integration starting in May 2026, about two months after the MSL41 release. This included the integration of renamed taxa, while newly created taxa remained absent and abolished taxa had not yet been removed (see Additional file 1, Supplementary Material S3). As part of this work, ICTV has publicly documented several persistent identifiers (described above) that link taxa in successive ICTV Taxonomy versions, which should facilitate rapid integration of new releases. These identifiers are exposed in the ICTV ontology metadata and support the generation of SSSOM ICTV-NCBI mappings. In the released mapping file, lexical ICTV-to-NCBI Taxon correspondences are expressed with skos:exactMatch, enabling alignment between ICTV taxonomy and NCBI Taxonomy identifiers used by downstream sequence resources such as GenBank.

Results

Unified ontology for ICTV taxonomy and OLS deployment

We have produced a unified ontology for the ICTV taxonomy, aggregating all releases into a single resource. The ontology is available for download or via OLS and enables programmatic access to current and historical classifications. Ontology updates are automatically triggered by new ICTV releases and reflected in OLS, typically within 24 hours.

Asynchronously, ICTV-NCBI mappings are generated in SSSOM format, enabling bidirectional resolution between the taxonomies. Mappings are maintained in a dedicated EVORA virus-taxonomy-mappings repository and updated independently [33]. The mapping supports bidirectional many-to-many resolution between ICTV and NCBI taxonomies and facilitates integration with downstream resources. The overall architecture of the generation and publication workflow is shown in **Figure 3**.

To simplify integration into downstream applications, ICTV-specific helper libraries were developed on top of the standard OLS API. These libraries are provided in JavaScript, Python and PHP and encapsulate resolution logic (synonyms, obsolescence, lineage, mappings) while returning normalised data about taxon objects. These objects contain identifiers, labels, synonyms, ranks, lineage, obsolescence metadata and cross-references, enabling application developers to integrate ICTV-aware logic without direct manipulation of raw API calls. The helper libraries prioritise exact identifier and name-based routes before free-text fallback. A bare ICTV ID returns the corresponding OWL class from the latest matching MSL release by default, while an explicit MSL release retrieves the corresponding historical state. For VMR records represented in the ontology, a VMR identifier resolves through the corresponding OLS individual and its rdf:type relation to the current species OWL class.

The OLS API documentation, ICTV-specific guidance and helper libraries are publicly available [34] (see Additional file 1, Supplementary Material S1). Together, the unified ontology, its OLS deployment, the helper libraries and the complementary SSSOM mappings constitute an integrated ontology-based access resource for automated resolution, cross-taxonomy linkage and reproducible versioning.

Reference application and early adopters

A public demonstration interface, the ICTV Taxon Resolver [35] web application, was developed as a reference implementation using the JavaScript helper library. The interface provides two selectable actions. The default action, 'Resolve to latest ICTV', returns the current accepted ICTV taxon when one can be resolved. 'Show history across MSLs' starts from the OWL class representing the taxon identified from the query and displays the release-specific OWL classes linked to it across the MSL releases included in the ontology. Consequently, the displayed history may begin after MSL1 or end before MSL41, for example when a taxon was introduced later or subsequently abolished. The source code is available from the EVORA ICTV Resolver GitHub repository [36]. The resolver allows users to submit current and historical ICTV taxon names and virus names, ICTV identifiers, Internationalized Resource Identifiers (IRIs) or NCBI Taxon IDs and receive the corresponding currently accepted ICTV taxon or taxa, together with their full lineages and revision history. Concise usage guidance is displayed directly beneath the interface title. This tool illustrates practical applicability while serving as a functional community resource. The interface and an example resolution of the former species name *Zika virus* to its post-2022 name and lineage, *Orthoflavivirus zikaense*, are shown in **Figure 4**. For example, in history mode, the relationship between *Alphabaculovirus* and *Nucleopolyhedrovirus* illustrated in Figure 1 can be traced from MSL23 back to MSL13. The resolver is a demon-

stration and reference implementation built on the JavaScript helper library and does not expose every capability of the OLS-backed ICTV ontology API, for which complete developer documentation is available separately.

Among the first operational deployments using the ICTV ontology API is the EVORA Portal [37], a consolidated catalogue of research infrastructures' resources and services in virology designed to support outbreak response and pandemic preparedness. The utilisation of the ICTV ontology API is integrated through one of the helper libraries directly into the catalogue generation workflow, enabling retrieval of up-to-date taxonomic terms along with associated lineage and synonym information. This ensures consistent use of the latest ICTV taxonomy, even when metadata provided by contributing research infrastructures correspond to earlier ICTV releases. As an added value for users of the research infrastructures, lineage information and historical synonyms retrieved through the ontology API enable enhanced search capabilities. This allows users to find resources using outdated taxa or alternative names, an option that was not available in the former search module.

Early adoption of the ICTV ontology API has also been observed in an external initiative concerned with pandemic preparedness and data integration: the Pandemic PACT programme [38]. This programme is now using ICTV taxonomy via the OLS API to align viral pathogen families and scientific names within its scope with the latest ICTV taxonomy (see Acknowledgements section). The retrieved ICTV metadata will be incorporated into Pandemic PACT dashboard visualisations [39], where they will be integrated with metadata from other ontologies accessed through the OLS, such as the Systematized Nomenclature of Medicine (SNOMED), aligning up-to-date viral pathogen taxonomy with related information, including associated diseases.

Integration of ICTV taxonomy through an API enables timely and automated updates across resources. The European Virus Archive (EVA) [40], a network of distributed virus collections using a single web catalogue for virus and virus-related material ordering and distribution [41], plans to integrate the ICTV ontology into its workflows using OLS API queries to maintain local references to virus taxonomy aligned with the most recent ICTV releases. This will allow EVA datasets to remain synchronised with official taxonomy updates while preserving historical annotations associated with archived materials, which will enhance the interconnectivity of research and data management processes, improving resource discovery and data consistency.

These deployments demonstrate practical applicability across research infrastructures and public health platforms. The system enables automated resolution, cross-taxonomy linkage, reduced manual curation and reproducible versioning.

Discussion

Virus data resources include numerous databases with different scopes, data models, functionalities and degrees of FAIR maturity, as reflected in recent reviews of virus databases [42]. This heterogeneity creates practical challenges for interoperability and synchronisation with authoritative taxonomic references such as ICTV taxonomy. This work demonstrates how relational datasets can be easily transformed and exposed through scalable, public, ontology-based APIs. Standards-based ontology-enabled APIs drastically reduce the work needed to make datasets machine-actionable. Here, we developed an ontology for the widely used and regularly updated ICTV virus taxonomy and exposed it through a public API, providing downstream systems with programmatic access to current and historical taxonomic information and the ability to resolve former terms to the current classification, thereby supporting uses ranging from direct queries to recurring automated updates. The ontology infrastructure acts as a semantic resolution layer that continuously bridges evolving authoritative ICTV releases with downstream computational ecosystems and legacy taxonomic references. Timely updates, in turn, reduce discrepancies that arise when downstream systems rely on outdated or partially synchronised taxonomic references and enable reproducible taxonomic resolution across computational workflows. Research infrastructures, sequence repositories, biodiversity catalogues, and knowledge bases can use automated alignment mechanisms to maintain consistency with official ICTV classifications, reducing the need for extensive manual curation.

Persistent ICTV identifiers and replacement relations provide a structured mechanism to track taxonomic evolution, historically prone to ambiguity and data fragmentation, in a computationally accessible way. Complementary ICTV-NCBI mappings, together with ontology cross-references to associated sequence resources, provide a pragmatic bridge between the authoritative, annually updated ICTV classification and NCBI taxonomy. This improves reproducibility and supports FAIR principles, and their progressive implementation [43, 44, 45]. While this approach substantially improves interoperability and historical resolution, synchronisation across downstream resources still depends on heterogeneous update cycles and integration strategies. In addition, some complex taxonomic transitions, such as splits, merges, or taxon abolitions, may still require complementary expert interpretation in specific downstream analytical contexts.

These capabilities help connect distributed data systems in FAIR-aligned federated environments, where independently maintained resources must remain discoverable, accessible and interoperable, including initiatives such as the European Open Science Cloud (EOSC) and the European Health Data Space (EHDS).

Consistent and machine-actionable taxonomy references support large-scale computational analyses, automated data integration workflows and reproducible comparative studies by reducing ambiguity in taxonomic interpretation. This contributes to improved retrieval and harmonisation of virological data across interoperable research infrastructures and data-sharing environments [46], supporting more reliable data integration, comparative analyses, evidence-based decision making, and more informed experimental design and data interpretation. The infrastructure also establishes a foundation for future enhancements, including expanded mappings with sequence repositories and the possible development of additional interfaces relying on the public OLS API.

Our approach aligns with the article "Four principles to establish a universal virus taxonomy" [47] that outlines key recommendations to achieve a coherent and comprehensive virus taxonomy. Our work helps make ICTV taxonomy more useful by ensuring that references to the official, evolution-based ICTV taxonomy, can be easily maintained and kept up-to-date, irrespective of the intended use or alternate classification scheme. For example, classifying viruses to support the surveillance and public health response to disease outbreaks (Pathogens prioritization: a scientific framework for epidemic and pandemic research preparedness) [48] results in a classification that is significantly different than that of the ICTV taxonomy. It is however still important to reference the current evolutionary-based classification so that our current knowledge on viruses can be quickly and easily adapted to newly identified or newly emerging ones. The ICTV taxonomy serves as the universal reference for virus classification, while the services we report here enable the critical links needed to connect that taxonomy to alternate schemes.

Adoption of the ICTV ontology API by resources relying on virus taxonomy (EVORA and Pandemic PACT) demonstrates its practical applicability. Ontology-based APIs can facilitate alignment between biological taxonomy resources and clinical terminologies used in public health systems, enabling consistent pathogen identification across research infrastructures, data catalogues, and policy-oriented dashboards. By supporting automated retrieval of up-to-date taxonomic terms, lineage information and synonym relationships, the API-based approach supports consistent taxonomy us-

age across distributed data infrastructures and reduces the need for manual synchronisation of taxonomic references similarly to interoperability approaches explored in tools such as Treemendous for reconciling taxonomic backbones in tree species naming [49].

Taxonomic resources and biodiversity platforms such as NCBI Taxonomy and GBIF could use the OLS API to implement more automated synchronisation with ICTV releases. Databases such as ENA and GenBank, which depend on the NCBI taxonomy and its integration of ICTV classifications [50], would in turn benefit from improved data integration and accuracy. In cases where updates of virus taxonomy within NCBI do not immediately reflect the latest ICTV release, these resources could additionally leverage the ICTV ontology API, coupled with the provided SSSOM mappings, to maintain up-to-date virus taxonomy.

Our work builds on established FAIR and semantic standards [25, 51]. The inclusion of detailed metadata and the reuse of established semantic standards are critical for ensuring that the ICTV taxonomy serves as a reliable reference across different platforms and applications. This approach provides a lightweight and sustainable mechanism for publishing structured datasets as interoperable APIs, by leveraging an existing ontology service (OLS) and GitHub's publicly available workflow automation. This approach is applicable to other domain-specific datasets. In parallel with the public OLS-based service, ICTV is using a dedicated access mechanism for data exchange with NCBI and plans a broader API providing access to a more comprehensive ICTV dataset. These complementary services support specialised or higher-volume exchanges, while the OLS API remains the public, standards-based access layer.

More broadly, this work illustrates how ontology technologies can expose structured datasets as semantically enriched graph representations through interoperable APIs. Traditional database schemas are optimised for storage and transactional operations but often require custom APIs to expose their content programmatically. In contrast, ontology-based representations allow relationships between entities to be explicitly encoded and navigated as a graph, enabling more flexible data integration and discovery. Such representations align with knowledge graph and Linked Open Data (LOD) approaches [52, 53]. More generally, this work demonstrates a reusable pattern for exposing structured, domain-specific datasets as interoperable ontologies and APIs by leveraging established ontology infrastructures, such as the OLS for biomedical ontologies.

Conclusion

This work shows that representing an evolving classification system, such as the ICTV taxonomy, as a unified ontology hosted through a public, scalable, standards-based API, increases its accessibility and addresses key challenges in access, versioning and interoperability. The resulting infrastructure supports backward-compatible resolution of virus taxonomy across historical ICTV releases, enabling historical traceability and alignment with current classifications through semantic modelling, persistent identifiers and interoperable mappings.

By building on open standards, automated workflows and existing ontology infrastructure services, this approach supports long-term sustainability and scalability while making ICTV taxonomy more readily integrable into computational workflows. It also facilitates synchronisation between authoritative taxonomic references and downstream biological, biomedical and public-health data infrastructures, supporting broader adoption of the latest official ICTV taxonomy as the reference standard and strengthening data consistency, reproducibility and preparedness for emerging viral threats, including through more timely dissemination of high-impact updates such as the pandemic-based designation of the species *Betacoronavirus pandemicum*.

Future developments may support broader interoperability with additional taxonomy providers, sequence repositories and interconnected semantic resources. More broadly, this work provides a reusable model for maintaining, exposing and reusing evolving taxonomic references through sustainable, machine-actionable semantic infrastructure.

Declarations

Ethics approval and consent to participate

Not applicable.

Consent for publication

Not applicable.

Funding

This work was supported by the EVORA project (European Union's HORIZON programme grant agreement N° 101131959). The contribution of the University of Alabama at Birmingham (UAB) team to the research reported in this publication was supported by the National Institute of Allergy and Infectious Diseases of the National Institutes of Health under Award Number U24AI162625. The content is solely the responsibility of the authors and does not necessarily represent the official views of the National Institutes of Health. The UAB team assisted with accessing all viral taxonomic data and associated metadata. The UAB team also served as consultants to assist in the understanding of the underlying ICTV data structures and data organization that were used to construct the ontology. In total, the UAB effort comprised approximately 10% of the overall effort.

Acknowledgements

This work is a product of the EVORA project (European Viral Outbreak Response Alliance) and the ICTV's UAB team.

We gratefully acknowledge Emilia Antonio and Alice Norton from the Pandemic PACT programme for their feedback on the adoption of the ICTV OLS API within Pandemic PACT to align viral pathogen families and scientific names with the latest ICTV taxonomy identifiers.

Authors' contributions

Based on Contributor Roles Taxonomy (CRediT) [54]

- Conceptualization: PL
- Project administration: PL, BC
- Resources: CH, EL, PL, BC, JM, HP
- Software: JM, PL
- Methodology: PL, JM, HP, RD, CH
- Writing – original draft: PL, RD
- Writing – review & editing: PL, RD, HP, JM, BC, CH, EL
- Visualization: PL, RD, CH, DMD

Disclosure of use of AI-assisted tools including generative AI

- During manuscript preparation and revision in 2024-2026, Philippe Lieutaud, Romain David and R. Curtis Hendrickson used OpenAI ChatGPT, initially with GPT-4o and subsequently with GPT-5-series models, including GPT-5.4, GPT-5.5 and GPT-5.6, at different stages as a writing and editorial aid [55].

The tool was used to suggest rewording and stylistic improvements, identify redundancy, improve the organisation of arguments, check conformity with journal requirements during final review, including reference formatting and the availability of cited links, and suggest improvements to figure layout and presentation. The AI-assisted textual and figure-design suggestions were treated as conceptual proposals and were not incorporated verbatim; no AI-generated images were used. The authors determined and validated all final scientific content, and produced the final figures.
- Philippe Lieutaud also used OpenAI ChatGPT with GPT-5.4 and GPT-5.5 to assist in drafting user and developer documentation published in the ICTV ontology and ICTV Resolver GitHub repositories. He used the same models to assist in porting the ICTV API helper library from Python to JavaScript and PHP, and to check consistency across the three implementations. GPT-5.5 was also used to suggest fixes for Zenodo release synchronisation involving .zenodo.json metadata and new GitHub release events in the ICTV ontology and ICTV-NCBI mapping repositories, and to help design a sharded, failure-resilient strategy for long-running ICTV-NCBI mapping updates. GPT-5.5 also assisted in developing a local script to convert the Word manuscript to LaTeX; Philippe Lieutaud produced and checked the final LaTeX files. The resulting documentation, code and workflow changes were reviewed and validated by the authors against the intended functionality of the libraries, release processes, mapping workflows and OLS API.
- Separately, on 7 October 2025, James McLaughlin used the GitHub Copilot coding agent (underlying model not recorded) to generate initial GitHub Actions implementations for building and releasing the ICTV ontology and for scheduling updates of the ICTV-NCBI mappings [56]. In the mapping repository, Copilot also drafted associated documentation and an initial modification supporting the incremental reuse of existing mappings. These contributions are preserved in the public development history of the corresponding repositories. The generated code was reviewed and merged by James McLaughlin and subsequently revised and extended by the authors.
- The AI-assisted tools were not used to generate scientific data or independently interpret the results. All generated suggestions and code contributions were critically reviewed and validated by the authors, who take full responsibility for the content of the manuscript and the associated software.

Competing interests

The authors declare that they have no competing interests.

Data availability

All ontology artefacts, helper libraries, mapping resources, example notebooks, and continuous-integration workflows are publicly available under open licences. The ICTV MSL41 source data used in this work are available as the ICTVdatabase MSL41.v1 release [21]. The exact EVORA ICTV ontology release used for this manuscript is archived in Zenodo as EVORA-project/ictv-ontology: 2026-Jul-28-74d7801 doi:10.5281/zenodo.21506852 [57]. The concept DOI 10.5281/zenodo.20610328 resolves to all versions. The exact ICTV-NCBI mapping release is archived in Zenodo as EVORA-project/virus-taxonomy-mappings: Virus Taxonomy Mappings Update, release-3 doi:10.5281/zenodo.21890424 [58]. The complete version history is accessible through the Zenodo concept DOI doi:10.5281/zenodo.20610592. Current development versions and latest releases are available from the public repositories listed below:

- ICTV database: https://github.com/ICTV-Virus-Knowledgebase/ICTVdatabase/
- ICTV ontology generation code, helper libraries, and generated ontologies (OWL/Turtle files): https://github.com/EVORA-project/ictv-ontology
- Latest ICTV ontology release: https://github.com/EVORA-project/ictv-ontology/releases/latest
- ICTV-NCBI SSSOM mappings: https://github.com/EVORA-project/virus-taxonomy-mappings
- Latest ICTV-NCBI mapping release: https://github.com/EVORA-project/virus-taxonomy-mappings/releases/latest
- Unified ICTV ontology in OLS: https://www.ebi.ac.uk/ols4/ontologies/ictv [59]

All resources are reusable, openly licensed and versioned, enabling independent verification and downstream integration.

NCBI Taxonomy taxdump archives used to verify the integration status of ICTV releases are available from the NCBI taxonomy archive: https://ftp.ncbi.nih.gov/pub/taxonomy/taxdump_archive/ [60]

Availability of supporting source code and requirements

- Project name: ICTV Ontology API and helper libraries
  - Project home page: https://github.com/EVORA-project/ictv-ontology
  - Documentation: https://evora-project.github.io/ictv-ontology/helpers/
  - Operating system(s): Platform independent
  - Programming language: language-agnostic for direct API access; helper libraries are provided in Python 3.8 or later, JavaScript as an ES module, and PHP 7.4 or later.
  - Other requirements: Internet access to the OLS API; depending on the helper library used, the Python requests library, the JavaScript Fetch API or the PHP cURL extension is required. For PHP, allow_url_fopen must also be enabled when accessing the remote ICTV-NCBI SSSOM mapping.
  - Licences:
    * ontology-generation code, Apache License 2.0;
    * ICTV-derived ontology data, Creative Commons Attribution 4.0 International (CC BY 4.0), with attribution to ICTV;
    * JavaScript, Python and PHP helper libraries, MIT License.
  - Version used for this manuscript: GitHub release 2026-Jul-28-74d7801; Zenodo version DOI 10.5281/zenodo.21506852
  - All versions: 10.5281/zenodo.20610328
  - RRID: SCR_028724
  - bio.tools ID: ictv_ontology_api
- Project name: ICTV-to-NCBITaxon SSSOM mapping
  - Project home page: https://github.com/EVORA-project/virus-taxonomy-mappings
  - Operating system(s): Platform independent
  - Programming language: language-agnostic for SSSOM file utilisation; SSSOM file generation code in Python.
  - Release archived at https://doi.org/10.5281/zenodo.21890424 [58]
  - License: CC0 1.0 Universal
  - RRID: SCR_028725
  - bio.tools ID: ictv_to_ncbitaxon_sssom_mapping
- Project name: ICTV Taxon Resolver

  - Project home page: https://github.com/EVORA-project/ictv-resolver
  - Live instance: https://evora-project.github.io/ictv-resolver
  - Programming languages: HTML, JavaScript and CSS.
  - Other requirements: A modern web browser with JavaScript and internet access to OLS.
  - License: MIT.
  - Version used: v1.0.5.
  - RRID: SCR_028722
  - bio.tools ID: ictv_taxon_resolver

- External service dependency: Ontology Lookup Service (OLS):
  - Project home page: https://www.ebi.ac.uk/ols4/
  - Documentation: https://github.com/EBISPOT/ols4/blob/dev/README.md [61]
  - Source code: https://github.com/EBISPOT/ols4
  - Software licence: Apache License 2.0

List of abbreviations

- **API(s):** Application Programming Interface(s)
- **CI/CD:** Continuous Integration and Deployment
- **COL:** Catalogue of Life
- **CURIE:** Compact Uniform Resource Identifier
- **DCTERMS:** Dublin Core Terms namespace
- **ENA:** European Nucleotide Archive
- **ETL process:** Extract, Transform, and Load
- **EVA:** European Virus Archive
- **EVORA:** European Viral Outbreak Response Alliance.
- **FAIR:** Findable, Accessible, Interoperable and Reusable
- **FOAF**: Friend of a Friend ontology
- **GBIF:** Global Biodiversity Information Facility
- **IAO:** Information Artifact Ontology
- **ICTV:** International Committee on Taxonomy of Viruses
- **IRI**: Internationalized Resource Identifier
- **LOD:** Linked Open Data
- **MCP:** Model Context Protocol
- **MSL:** Master Species List
- **NCBI:** National Center for Biotechnology Information
- **OLS:** Ontology Lookup Service
- **OWL:** Web Ontology Language
- **PROV-O**: Provenance Ontology
- **RDFS:** Resource Description Framework Schema
- **REST:** Representational State Transfer
- **SKOS:** Simple Knowledge Organization System
- **SNOMED:** Systematized Nomenclature of Medicine
- **SSSOM:** Simple Standard for Sharing Ontology Mapping
- **TAXRANK:** Taxonomic Rank Vocabulary
- **VMR:** Virus Metadata Resource

References

1. International Committee on Taxonomy of Viruses (ICTV). ICTV website. 2026. https://ictv.global/. Accessed 09 June 2026.
2. International Union of Microbiological Societies (IUMS). IUMS website. 2026. https://www.iums.org/. Accessed 09 June 2026.
3. Zerbini FM, Siddell SG, Mushegian AR, Walker PJ, Lefkowitz EJ, et al. Differentiating between viruses and virus species by writing their names correctly. Arch Virol 2022;167:1231–1234. doi:10.1007/s00705-021-05323-4
4. International Committee on Taxonomy of Viruses (ICTV). ICTV Master Species List (MSL). 2026. https://ictv.global/msl. Accessed 09 June 2026.
5. Simmonds P, Adriaenssens EM, Lefkowitz EJ et al. Changes to virus taxonomy, the international code of virus classification and nomenclature, and the ICTV statutes ratified by the International Committee on Taxonomy of Viruses (2025). *Arch Virol* 2026;171:23. doi:10.1007/s00705-025-06485-1
6. Black EJ, et al. Virus taxonomy: the database of the International Committee on Taxonomy of Viruses. Nucleic Acids Res 2026;54(D1):D776–D789. doi:10.1093/nar/gkaf1159
7. National Center for Biotechnology Information (NCBI). NCBI website. 2026. https://www.ncbi.nlm.nih.gov/. Accessed 09 June 2026.
8. GBIF. Global Biodiversity Information Facility website. 2026. https://www.gbif.org/. Accessed 09 June 2026.
9. Catalogue of Life. Catalogue of Life (COL). 2026. https://www.catalogueoflife.org/. Accessed 09 June 2026.
10. Wikidata. Wikidata. 2026. https://www.wikidata.org/. Accessed 09 June 2026.
11. National Center for Biotechnology Information (NCBI). GenBank. 2026. https://www.ncbi.nlm.nih.gov/genbank/. Accessed 09 June 2026.
12. European Nucleotide Archive (ENA). ENA Browser. 2026. https://www.ebi.ac.uk/ena/browser/home. Accessed 09 June 2026.
13. Boyle B, Hopkins N, Lu Z, et al. The taxonomic name resolution service: an online tool for automated standardization of plant names. BMC Bioinformatics 2013;14:16. doi:10.1186/1471-2105-14-16
14. EVORA. European Viral Outbreak Response Alliance project website. 2026. https://evora-project.eu/. Accessed 09 June 2026.
15. European Commission. European Viral Outbreak Response Alliance (EVORA), grant agreement ID 101131959. CORDIS. 2026. https://doi.org/10.3030/101131959. Accessed 09 June 2026.
16. Malone J, Stevens R, et al. Ten simple rules for selecting a bio-ontology. PLoS Comput Biol 2016;12(2):e1004743. doi:10.1371/journal.pcbi.1004743
17. McLaughlin J, Lagrimas J, Iqbal H, et al. OLS4: a new Ontology Lookup Service for a growing interdisciplinary knowledge ecosystem. Bioinformatics 2025;41(5):btaf279. doi:10.1093/bioinformatics/btaf279
18. EMBL-EBI. Ontology Lookup Service (OLS4). 2026. https://www.ebi.ac.uk/ols4/. Accessed 09 June 2026.
19. McMurry JA, Juty N, Blomberg N, et al. Identifiers for the 21st century: how to design, provision, and reuse persistent identifiers. PLoS Biol 2017;15(6):e2001414. doi:10.1371/journal.pbio.2001414
20. International Committee on Taxonomy of Viruses (ICTV). ICTV Virus Metadata Resource (VMR). 2026. https://ictv.global/vmr. Accessed 09 June 2026.
21. Hendrickson RC, Mims L, Lefkowitz EJ. ICTV-Virus-Knowledgebase/ICTVdatabase: Taxonomy Release: MSL41.v1 20260320 [Dataset]. Zenodo 2026. doi:10.5281/zenodo.19339543
22. Kibbe WA, Arze C, Felix V, et al. Disease Ontology 2015 update: an expanded and updated database of human diseases for linking biomedical knowledge through disease data. Nucleic Acids Res 2015;43(D1):D1071–D1078. doi:10.1093/nar/gku1011
23. Lebo T, Sahoo S, McGuinness D, et al. PROV-O: the PROV ontology. W3C Recommendation 2013. https://www.w3.org/TR/prov-o/. Accessed 12 May 2026.
24. Miles A, Bechhofer S. SKOS simple knowledge organization system reference. W3C Recommendation 2009. https://www.w3.org/TR/skos-reference/. Accessed 12 May 2026.
25. Xu F, Juty N, Goble C, Jupp S, Parkinson H, Courtot M. Features of a FAIR vocabulary. J Biomed Semantics 2023;14(1):6. doi:10.1186/s13326-023-00286-8
26. Smith B, Ashburner M, Rosse C, et al. The OBO Foundry: coordinated evolution of ontologies to support biomedical data inte-

gration. Nat Biotechnol 2007;25(11):1251–1255. doi:10.1038/nbt1346

27. Fielding RT, Taylor RN. Principled design of the modern Web architecture. ACM Trans Internet Technol 2002;2(2):115–150. doi:10.1145/514183.514185
28. International Committee on Taxonomy of Viruses (ICTV). ICTV database GitHub repository. 2026. https://github.com/ICTV-Virus-Knowledgebase/ICTVdatabase/. Accessed 09 June 2026.
29. David R, Rybina A, Burel J, et al. "Be sustainable": EOSC-Life recommendations for implementation of FAIR principles in life science data handling. EMBO J 2023;42:e115008. doi:10.15252/embj.2023115008
30. EVORA Project. EVORA ICTV ontology GitHub repository. 2026. https://github.com/EVORA-project/ictv-ontology. Accessed 09 June 2026.
31. David R, Baumann K, LeFranc Y, et al. Converging on a semantic interoperability framework for the European Data Space for Science, Research and Innovation (EOSC). In: Proceedings of the 2nd Workshop on Ontologies for FAIR and FAIR Ontologies (Onto4FAIR). 2023. doi:10.5281/zenodo.8102786
32. Matentzoglu N, Balhoff J, Bello S, et al. A simple standard for sharing ontological mappings (SSSOM). Database 2022;2022:baac035. doi:10.1093/database/baac035
33. EVORA Project. EVORA virus-taxonomy-mappings GitHub repository. 2026. https://github.com/EVORA-project/virus-taxonomy-mappings. Accessed 09 June 2026.
34. EVORA Project. EVORA ICTV ontology helper and API documentation. 2026. https://evora-project.github.io/ictv-ontology/helpers/. Accessed 09 June 2026.
35. EVORA Project. EVORA ICTV Taxon Resolver live interface. 2026. https://evora-project.github.io/ictv-resolver. Accessed 09 June 2026.
36. EVORA Project. EVORA ICTV Taxon Resolver GitHub repository. 2026. https://github.com/EVORA-project/ictv-resolver. Accessed 09 June 2026.
37. EVORA Project. EVORA Portal catalogue. 2026. https://portal.evora-project.eu/. Accessed 09 June 2026.
38. Pandemic PACT. Programme website. 2026. https://www.pandemicpact.org/. Accessed 09 June 2026.
39. Pandemic PACT. Dashboard visualisations. 2026. https://www.pandemicpact.org/grants/visualise. Accessed 09 June 2026.
40. European Virus Archive (EVA). EVA website. 2026. https://www.european-virus-archive.com/. Accessed 09 June 2026.
41. Romette JL, Prat CM, Gould EA, et al. The European Virus Archive goes global: a growing resource for research. Antiviral Res 2018;158:127–134. doi:10.1016/j.antiviral.2018.07.017
42. Ritsch M, Cassman NA, Saghaei S, Marz M. Navigating the Landscape: A Comprehensive Review of Current Virus Databases. *Viruses* 2023;15(9):1834. doi:10.3390/v15091834
43. Wilkinson MD, Dumontier M, Aalbersberg IJ, et al. The FAIR guiding principles for scientific data management and stewardship. Sci Data 2016;3:160018. doi:10.1038/sdata.2016.18
44. Sansone SA, McQuilton P, Rocca-Serra P, et al. FAIRsharing as a community approach to standards, repositories and policies. Nat Biotechnol 2019;37(4):358–367. doi:10.1038/s41587-019-0080-8
45. David R, Mabile L, Specht A, et al. FAIRness literacy: the Achilles' heel of applying FAIR principles. Data Sci J 2020;19(1):32. doi:10.5334/dsj-2020-032
46. Research Data Alliance COVID-19 Working Group. Recommendations and guidelines on data sharing. 2020. doi:10.15497/rda00052
47. Simmonds P, Adriaenssens EM, Zerbini FM, et al. Four principles to establish a universal virus taxonomy. PLoS Biol 2023;21(2):e3001922. doi:10.1371/journal.pbio.3001922
48. World Health Organization (WHO). Pathogens prioritization: a scientific framework for epidemic and pandemic research preparedness. Meeting report. 2024. https://www.who.int/publications/m/item/pathogens-prioritization-a-scientific-framework-for-epidemic-and-pandemic-research-preparedness. Accessed 09 June 2026.
49. Specker F, Paz A, Crowther TW, Maynard DS. Treemendous: an R package for integrating taxonomic information across backbones. PeerJ 2024;12:e16896. doi:10.7717/peerj.16896
50. Schoch CL, Ciufo S, Domrachev M, et al. NCBI taxonomy: a comprehensive update on curation, resources and tools. Database 2020;2020:baaa062. doi:10.1093/database/baaa062
51. Berners-Lee T. Linked Data. 2006. https://www.w3.org/DesignIssues/LinkedData.html. Accessed 09 June 2026.
52. Hogan A, Blomqvist E, Cochez M, et al. Knowledge graphs. ACM Comput Surv 2021;54(4):1–37. doi:10.1145/3447772
53. Kamdar MR, Musen MA. An empirical meta-analysis of the life sciences linked open data on the web. Sci Data 2021;8:24. doi:10.1038/s41597-021-00797-y
54. National Information Standards Organization (NISO). Contributor Roles Taxonomy (CRediT). 2026. https://credit.niso.org/. Accessed 09 June 2026.
55. OpenAI (2026). ChatGPT (GPT-4o and GPT-5-series models, including GPT-5.4, GPT-5.5 and GPT-5.6) [large language models]. Responses to queries made by Philippe Lieutaud, Romain David and R. Curtis Hendrickson during manuscript preparation and revision, June 2024–August 2026. https://chatgpt.com/. Accessed 24 August 2026.
56. GitHub (2025). GitHub Copilot coding agent (underlying model not recorded) [AI-assisted coding agent]. Responses to tasks initiated by James McLaughlin, 7 October 2025. https://github.com/EVORA-project/ictv-ontology/pull/45. Accessed 09 June 2026; https://github.com/EVORA-project/virus-taxonomy-mappings/pull/2. Accessed 09 June 2026.
57. McLaughlin J, Lieutaud P, Hendrickson C. EVORA-project/ictv-ontology: 2026-Jul-28-74d7801 [Software]. Zenodo 2026. doi:10.5281/zenodo.21506852
58. McLaughlin J, Lieutaud P. EVORA-project/virus-taxonomy-mappings: Virus Taxonomy Mappings Update. Version release-3 [Software]. Zenodo 2026. doi:10.5281/zenodo.21890424
59. EMBL-EBI. Unified ICTV ontology in OLS. 2026. https://www.ebi.ac.uk/ols4/ontologies/ictv. Accessed 09 June 2026.
60. National Center for Biotechnology Information (NCBI). NCBI Taxonomy archive (taxdump archive). 2026. https://ftp.ncbi.nih.gov/pub/taxonomy/taxdump_archive/. Accessed 09 June 2026.
61. EMBL-EBI. OLS4 server documentation. 2026. https://github.com/EBISPOT/ols4/blob/dev/README.md. Accessed 09 June 2026.

Additional files

Additional file 1

File name: Additional_file_1_Supplementary_material.pdf

File format: .pdf

Title: Supplementary materials—API use, helper libraries, provider landscape and mapping specification

Description: Supplementary Materials S1–S4 provide API usage examples, the helper-library architecture, a comparison of virus taxonomy providers and the ICTV-NCBI mapping specification.

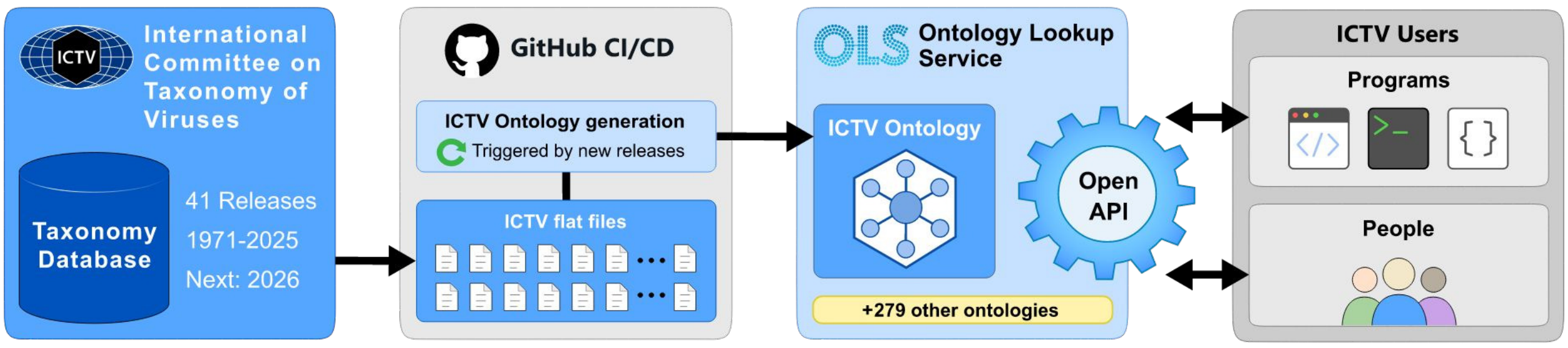

Graphical abstract. Unified ICTV ontology generation and public API access. Diagram showing 41 International Committee on Taxonomy of Viruses (ICTV) taxonomy releases transformed through GitHub continuous integration and continuous delivery (CI/CD) into a unified ICTV ontology hosted by the Ontology Lookup Service (OLS). An open application programming interface (API) provides programmatic access for software and browsing access for users.

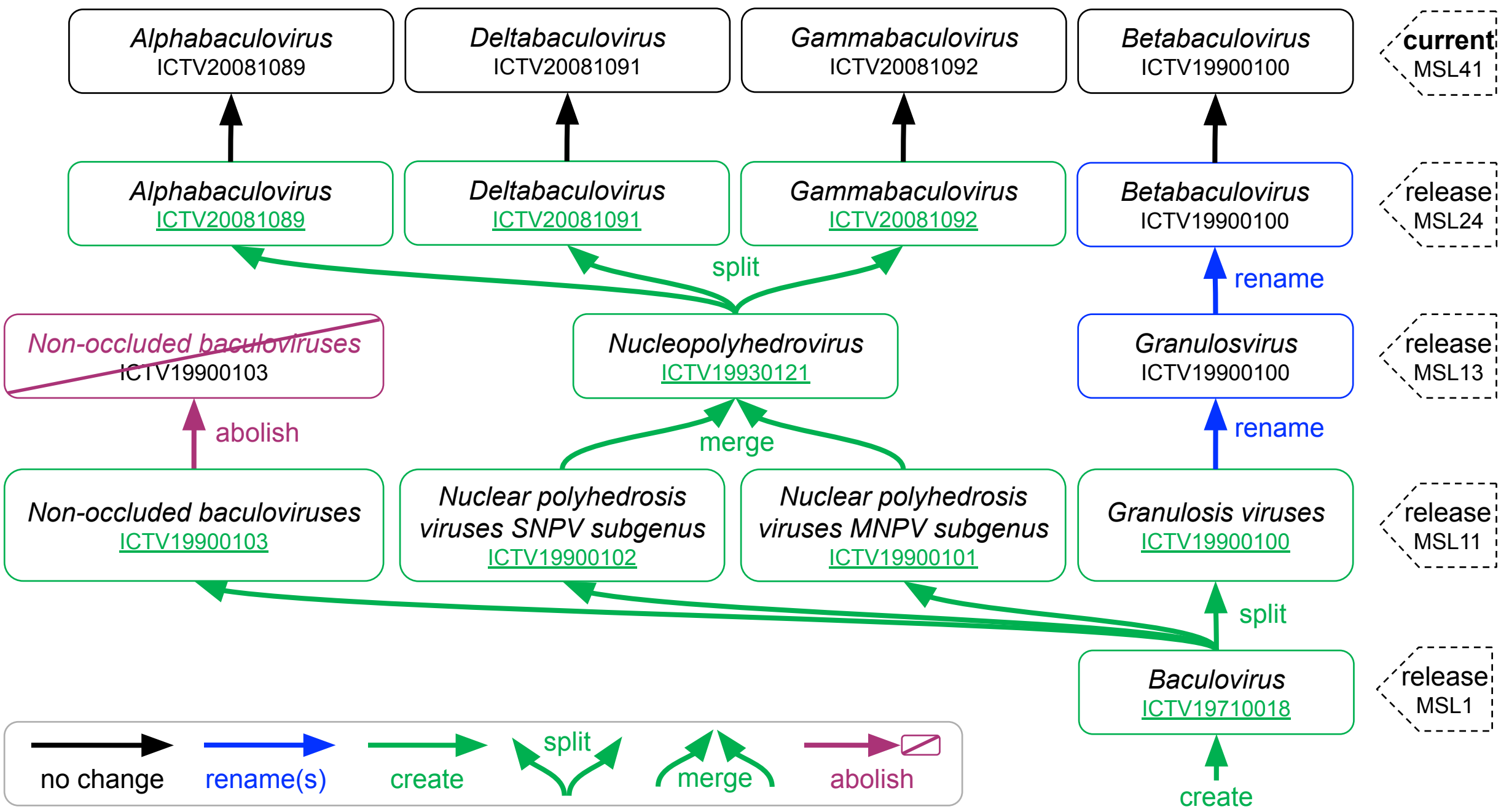

Figure 1. Illustrative taxonomic changes across ICTV releases for a genus-level taxon first recorded in MSL1. A diagram of historical and current genus names originating with the genus *Baculovirus* (ICTV19710018), first recorded in the first ICTV release, and ending with the four currently recognised genera. This example illustrates how the grouping of species into genera, although species are not shown here, can be redefined over time as viruses are better understood and characterised. In this historical trajectory, the original genus was first split into four genera; two of these were then merged; and the resulting lineage was later split into three genera. This illustrates some of the complexity that can occur in the history of a single taxon, and more broadly how taxonomic changes across different ranks and releases can make the overall taxonomy difficult to trace. It also shows how ICTV IDs are assigned to link historical versions of the same taxon. When a taxon is created, split or merged (green single, diverging or converging arrows, respectively), a new ICTV ID is assigned (underlined and in green). When taxa are renamed, promoted, demoted or moved, the ICTV ID remains the same, linking the different versions.

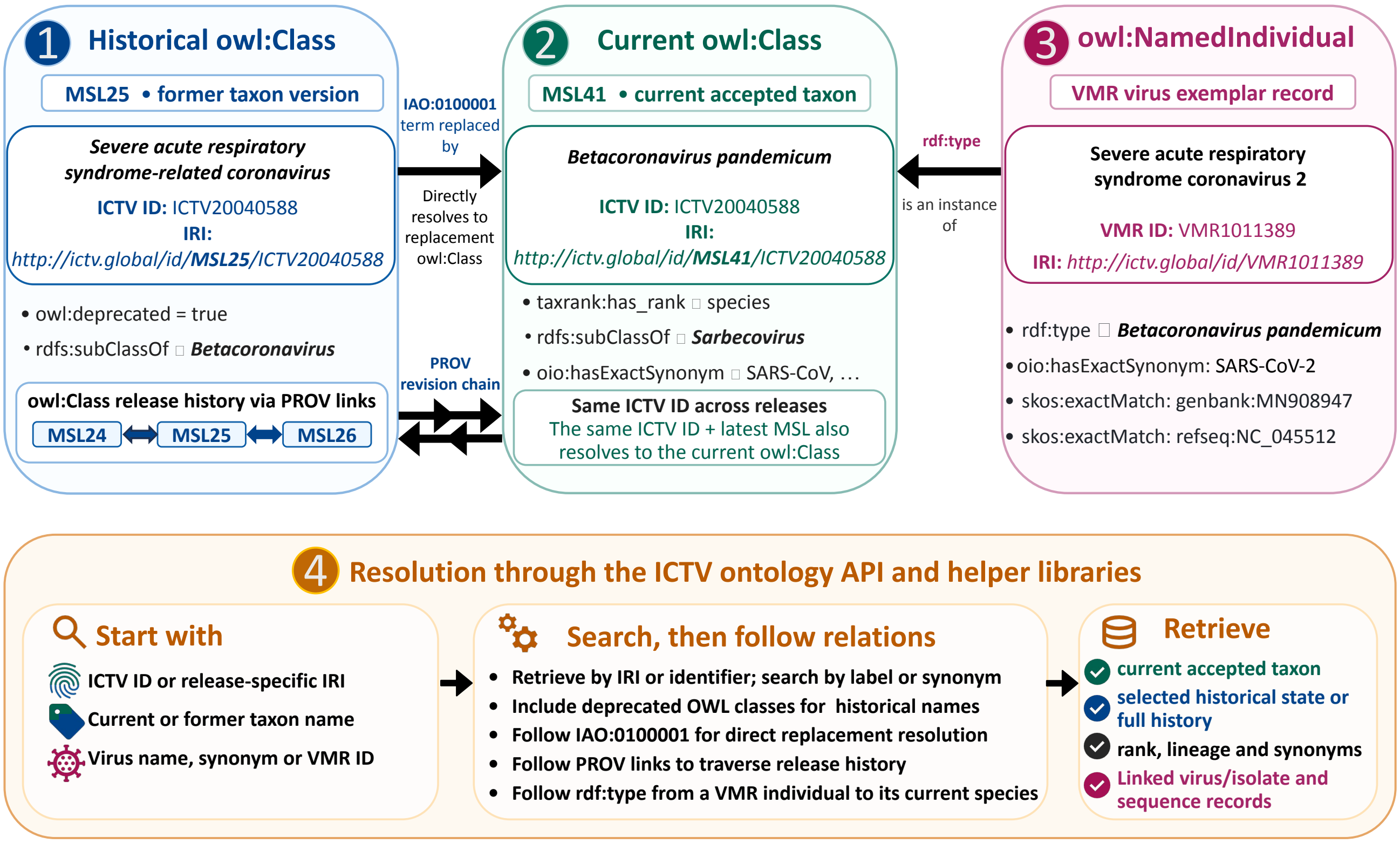

Figure 2. Historical and current taxon resolution using ICTV taxon and VMR virus record information. A former MSL25 taxon version is represented as a release-specific OWL class (i.e. owl:Class) (1), marked as deprecated with "owl:deprecated = true", and linked through IAO:0100001 to its current MSL41 representation, while PROV relations connect successive historical release states. The current accepted taxon is represented by a distinct OWL class carrying the same stable ICTV identifier (2). In simple taxon rename paths, this identifier can also be combined with the latest MSL release to retrieve the corresponding current OWL class. A VMR virus exemplar record is represented as an owl:NamedIndividual and asserted as an instance of its corresponding species OWL class using rdf:type (3). The resolution workflow (4) shows how ICTV identifiers and release-specific IRIs, current or former taxon names, and virus names, synonyms or VMR identifiers can be used through the ICTV ontology API and helper libraries to retrieve current taxa, historical states, lineage, synonyms and linked sequence records. In the context of this ontology, an OWL class represents an ICTV taxon and should not be confused with the ICTV rank class; rdfs:subClassOf expresses hierarchical relations between these ontology entities. The statement owl:deprecated = true applies to all historical, release-specific OWL classes representing taxa. Deprecated OWL classes linked by either an IAO:0100001 (replaced by) relation or prov:hadRevision link have been updated in a subsequent release. Only OWL classes with owl:deprecated = true and neither an IAO:0100001 relation nor a prov:hadRevision link represent truly abolished taxa.

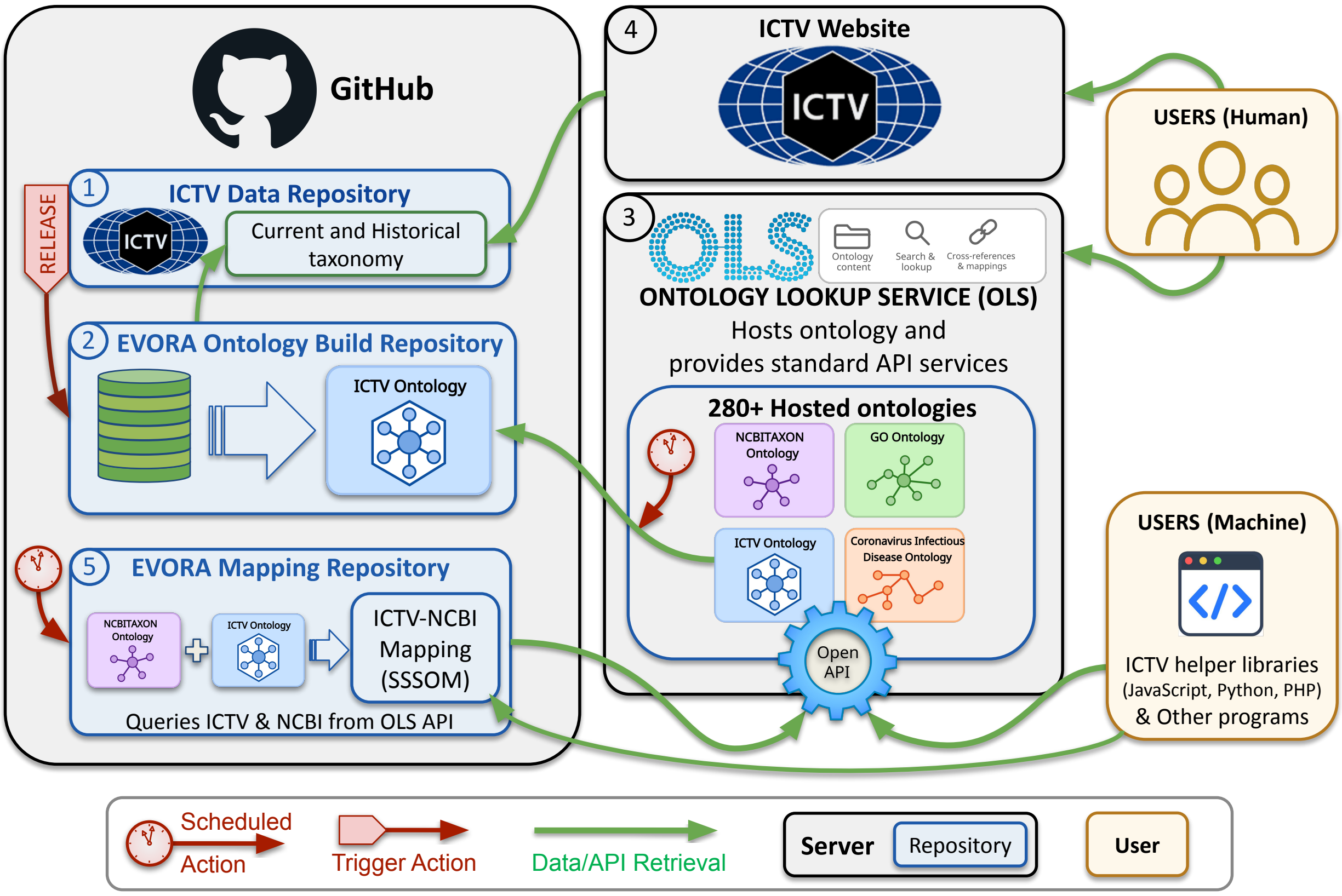

Figure 3. Automated data flows for ICTV ontology generation, publication, ICTV-NCBI mapping and API access. Official ICTV taxonomy data are released through the public ICTV Data repository (1), hosted on GitHub. A new release triggers the EVORA CI/CD workflow hosted in the EVORA Ontology Build repository (2), which retrieves the ICTV release files, transforms them into OWL ontology artefacts and merges them into a unified ICTV ontology. In this unified ontology, the latest ICTV release represents the current authoritative taxonomy, while entities from previous releases are retained as deprecated historical OWL classes linked through explicit semantic relations. Through scheduled actions, the unified ontology is loaded into the Ontology Lookup Service (OLS) (3), which provides both a browsable human interface and standard API services. The same ICTV source data also feed the ICTV website (4), which provides the official human-facing access point to ICTV taxonomy. In parallel, a separate EVORA Mapping repository (5) runs scheduled workflows that query OLS to use the latest ICTV and NCBITaxon ontologies to generate ICTV–NCBI mappings in SSSOM format; these mappings are stored in the corresponding public repository. Downstream access is supported through direct API queries and language-specific OLS ICTV API helper libraries, enabling external applications to perform historical taxon resolution, cross-resource mapping and to reduce manual curation. Green arrows indicate data/API retrieval actions; dark red arrows indicate workflow trigger actions; dark red clocks indicate scheduled actions. Black boxes represent servers, blue boxes repositories and brown boxes users.

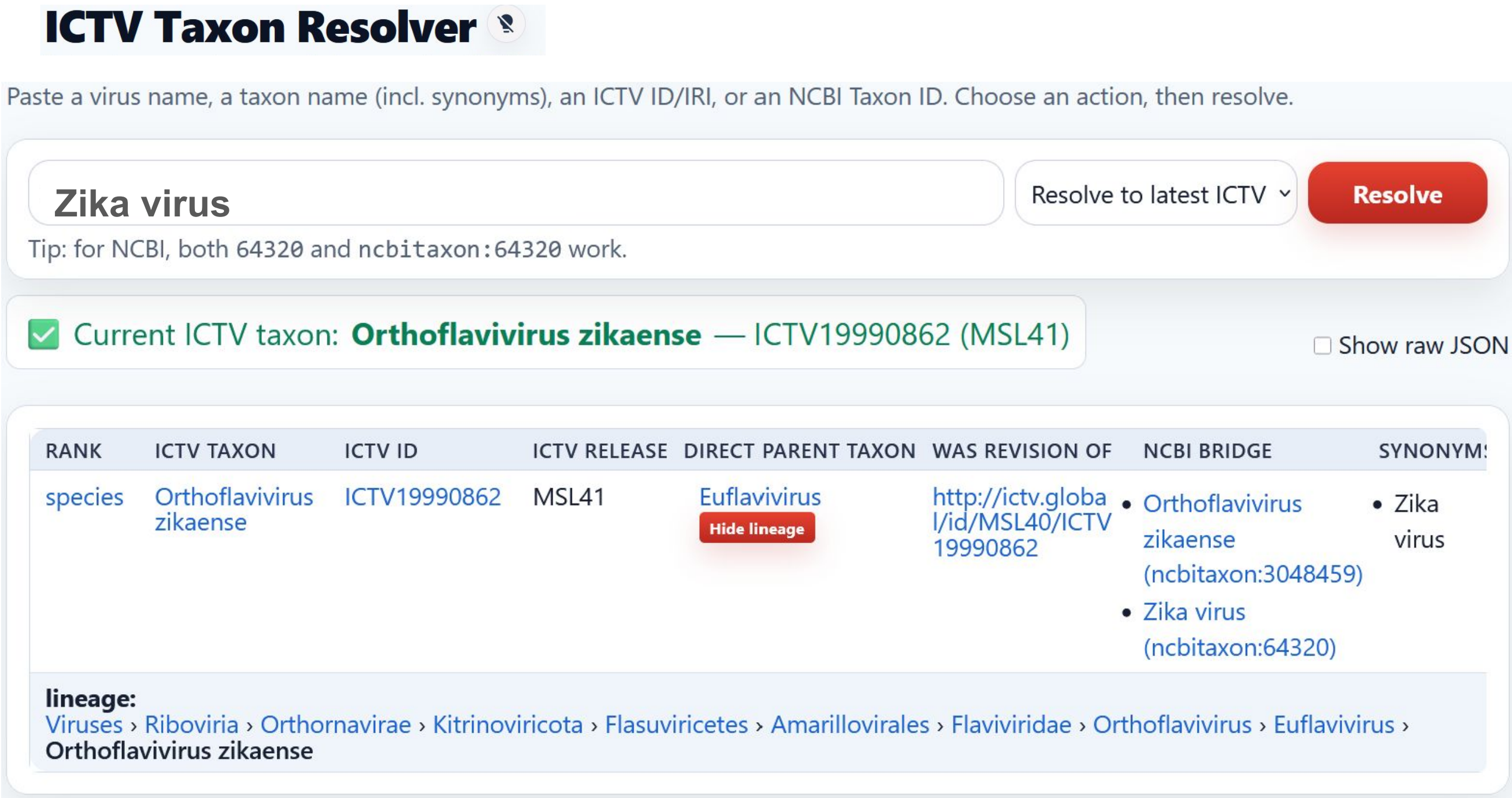

Figure 4. ICTV Taxon Resolver and programmatic taxonomic resolution. The ICTV Taxon Resolver interface illustrates automated resolution of historical or synonymous virus names to the currently accepted ICTV taxon, including lineage reconstruction and cross-references to external identifiers. The resolver is a reference implementation built on the JavaScript helper library that encapsulates Ontology Lookup Service (OLS) API calls. It accepts heterogeneous user inputs, including historical virus names, ICTV identifiers, Internationalized Resource Identifiers (IRIs), synonyms and NCBI Taxon identifiers, and applies a resolution strategy combining label matching, synonym detection, obsolete-term replacement chains and cross-taxonomy mappings. The output panel displays the currently accepted ICTV taxon together with its hierarchical lineage, rank, revision history and available cross-references to external resources.

Supplementary source
Click here to access/download;LaTeX - Other (clo, cls, sty etc.);supplementary_material.tex

Supplementary material for: Programmatic access to ICTV virus taxonomy through a public ontology API

Philippe Lieutaud et al.

1 Supplementary Material

This supplementary file accompanies the manuscript and provides implementation-oriented documentation, developer guidance, and additional context for comparative evaluation and mapping specifications.

1.1 Supplementary Material S1 – API usage examples

In addition to the generic OLS4 API documentation, a dedicated, ICTV-specific documentation page is provided on EVORA ICTV-ontology GitHub repository. This resource documents both direct use of the ICTV ontology via the generic OLS API, and use of the ICTV-specific helper libraries (see Supplementary Material S2), and enables implementation of common taxonomic resolution workflows. It complements the general OLS documentation by specifically addressing ICTV-related use cases:

- OLS4 Server documentation: `https://github.com/EBISPOT/ols4/blob/dev/README.md` [1]
- OLS4 API documentation: `https://www.ebi.ac.uk/ols4/api-docs` [2]
- EVORA ICTV helper and API documentation page: `https://evora-project.github.io/ictv-ontology/helpers/` [3]

Typical EVORA/ICTV use cases include retrieving taxonomy entities, resolving obsolete taxa, reconstructing lineage information, and retrieving synonyms and historical names.

OLS4 provides multiple REST controllers for querying classes, properties, entities, individuals, and search through v1 and v2 entry points. The v1 endpoints are retained as a legacy interface for compatibility with applications developed against OLS3 and continue to provide useful operations, notably the /api/suggest endpoint, which is not currently available in v2. The v2 endpoints return richer representations of ontology entities and their semantic links and are therefore used for the direct API examples below.

Together, these endpoints support typical ICTV use cases. For example, the paginated v1 /terms endpoint provides access to the full ontology, including historical and obsolete terms, whereas the v2 /classes and /individuals endpoints support direct retrieval by double URL-encoded IRI and text search through the documented search parameter, optionally constrained using searchFields and exactMatch. Because OWL classes from earlier MSL releases are marked as deprecated and exposed by OLS as obsolete entities, includeObsoleteEntities=true is required when they must be included in search or collection results. Metadata remain available in returned

entities and linked entities, but the presence of a field in the response does not imply that it is available as an independent server-side filter. Historical names and obsolete taxa can be resolved using metadata integrated into the ontology, notably oboInOwl:hasExactSynonym, IAO:0100001 (term_replaced_by) and PROV revision relations.

As an example, the currently accepted taxon corresponding to the species formerly known as "*Severe acute respiratory syndrome coronavirus*" can be identified by searching for this former label among obsolete classes:

GET https://www.ebi.ac.uk/ols4/api/v2/ontologies/ictv/classes?search=Severe%20acute%20respiratory%20syndrome%20coronavirus&searchFields=label&exactMatch=true&includeObsoleteEntities=true

Among the returned historical occurrences, the release-specific OWL class http://ictv.global/id/MSL22/ICTV20040588 contains an IAO:0100001 relation pointing to the current accepted OWL class:

http://ictv.global/id/MSL41/ICTV20040588

After double URL encoding, this replacement IRI can be retrieved directly:

GET https://www.ebi.ac.uk/ols4/api/v2/ontologies/ictv/classes/http%253A%252F%252Fictv.global%252Fid%252FMSL41%252FICTV20040588

The returned entity represents the current species *Betacoronavirus pandemicum*. Thus, the first request identifies the historical OWL class and provides the replacement IRI required for direct retrieval of the current OWL class.

Stable ICTV identifiers, such as ICTV20040588, serve as direct entry points. The EVORA repository further provides example notebooks and utilities illustrating typical workflows such as querying taxa by label or ICTV identifier, following obsolescence relationships, and reconstructing the history of a taxon across releases.

The equivalent resolution can be performed using the helper libraries, which simplify the required processing. The following example uses the JavaScript helper library from an HTML page:

```
<script type=”module”>

import { ICTVApi } from

”https://cdn.jsdelivr.net/gh/EVORA-project/ictv-ontology/helpers/js/ictv-api.js”;

const api = new ICTVApi();

const result = await api.resolveToLatest(

”Severe acute respiratory syndrome coronavirus”

);

console.log(result);

</script>
```

The helper encapsulates the name-matching and replacement-resolution logic and returns the corresponding current ICTV taxon; in this example, console.log(result) displays the returned object in the browser's developer console. A screenshot of a completed name-resolution result is provided in Figure 4 using the complementary Zika virus example.

1.2 Supplementary Material S2 – Helper library architecture

Helper libraries, developed in JavaScript, Python and PHP, simplify integration into computational pipelines. These libraries encapsulate the main ICTV resolution use cases logic on top of the OLS API and hide the complexity of direct ontology queries from downstream developers.

The JavaScript helper is provided as a browser-compatible JavaScript module for web applications. It exposes methods for resolving terms to the latest accepted ICTV taxon, retrieving taxon revision histories across MSL releases, resolving virus-name individuals through their parent taxa, handling direct taxon names and ICTV, VMR or NCBI Taxon identifiers, and returning lineage information.

The PHP helper provides equivalent functionality for PHP-based services, allowing resolution of identifiers and names, traversal of replacement chains, export of releases and retrieval of isolate-related information.

The Python helper provides the same high-level operations for scripts, Extract, Transform, and Load (ETL) workflows and Jupyter-based analyses. This makes it suitable for pipeline integration and reproducible computational notebooks.

Across all three implementations, the same general resolution strategy is applied:

1. recognise ICTV IRIs and ICTV IDs;
2. optionally map NCBI Taxon identifiers to ICTV taxa;
3. treat individuals, such as isolates, as entry points and resolve them to the corresponding species;
4. fall back to labels and synonyms where needed;
5. when an entity is obsolete, follow the replacement chain until final non-obsolete taxa are reached.

The libraries return a normalised ICTV taxon object containing identifiers, IRI, label, synonyms, rank, lineage, obsolescence status, revision links and, where available, NCBI mappings. This abstraction allows developers to focus on domain logic rather than the details of the OLS API.

Description examples and further details available from:

`https://github.com/EVORA-project/ictv-ontology/tree/main/helpers` [4]

A public browser-based ICTV Taxon Resolver was built as a reference implementation of this logic using the JavaScript helper library. It accepts historical or current virus names, ICTV identifiers, IRIs and NCBI Taxon identifiers, and returns the latest accepted ICTV taxon together with its lineage and mapping information.

To demonstrate how these helpers can be used in practice, we built a public, browser-based ICTV Taxon Resolver, using the JavaScript library. It is available as open source here:

`https://github.com/EVORA-project/ictv-resolver` [5]

with a live, interactive instance at:

`https://evora-project.github.io/ictv-resolver` [6]

This tool serves both as a practical service for virologists and as a didactic example of how to integrate the OLS-backed ICTV ontology into web applications such as those used by EVORA.

1.3 Supplementary Material S3 – Landscape of virus taxonomy providers and integration patterns (June 2026)

This supplementary section provides a qualitative overview of major resources involved in the dissemination, integration, and reuse of virus taxonomy. This analysis aims to characterise how ICTV taxonomy is propagated across the data resource ecosystem and to highlight practical implications for programmatic use.

The comparison focuses on key dimensions relevant to computational workflows, including: (i) recency relative to ICTV releases, (ii) availability of historical names and synonym information, (iii) persistence of identifiers across releases, (iv) availability of programmatic access (e.g. APIs or bulk downloads), (v) support for lineage reconstruction, and (vi) integration with external resources.

1.3.1 Authoritative source

International Committee on Taxonomy of Viruses (ICTV) ICTV is the official authority responsible for virus classification and nomenclature [7]. It publishes the Master Species List (MSL) [8] and associated data files, which constitute the reference taxonomy used across virology, as well as the Virus Metadata Resource (VMR) [9] which links the species in that reference taxonomy to exemplar virus isolate genomes in GenBank [10]. At the time of submission of this manuscript, the most recent ICTV release is MSL41.v1, with 22 670 taxonomic terms, released in March 2026.

ICTV resources are comprehensive and regularly updated; they provide access to historical information through structured files and web interfaces. However, access to this information is primarily designed for human use. While raw data files are publicly available, no public API has historically been provided to support programmatic access to taxonomic history, automated resolution of obsolete terms, or identifier-based queries across releases. As a result, integration into computational workflows requires additional processing and interpretation.

1.3.2 Integration platforms embedding ICTV taxonomy

NCBI Taxonomy NCBI Taxonomy [11] provides a widely used taxonomic backbone integrated with sequence databases such as GenBank and ENA [12]. It incorporates ICTV taxonomy and offers persistent identifiers, extensive cross-links to biological data, and mature programmatic access via the E-utilities APIs and bulk downloads. In addition, NCBI provides monthly historical taxdump archives [13] as dated files named new_taxdump_YYYY-MM-01.zip, which allow changes in NCBI taxonomic content to be retrospectively examined. The observations described below were checked against the April, May and June 2026 archives (new_taxdump_2026-04-01.zip, new_taxdump_2026-05-01.zip and new_taxdump_2026-06-01.zip in the taxdump archives), the first three monthly archives after the March 2026 MSL41 release, and against the live NCBI Taxonomy service on 10 June 2026.

However, integration of ICTV releases has historically lagged behind the most recent official version and sometimes occurs progressively. As of April 2026 archive, NCBI appeared to still reflect the previous (MSL40) release for the examples checked: the recently (MSL41) renamed species "*Mamastrovirus suisorientalis*" (ICTV19950506) was absent, although the corresponding NCBI taxon later shown as ncbitaxon:3430637 was represented under its previous name, and the newly created species "*Mastadenovirus himalaiense*" (ICTV202522146) was absent from NCBI Taxonomy. In May 2026, renamed taxa such as "*Mamastrovirus suisorientalis*" (ICTV19950506, ncbitaxon:3430637) had begun to appear, while in the June 2026 archive and in the live NCBI

Taxonomy service checked on 10 June 2026, the newly created taxa checked here were still absent, suggesting a progressive integration of the latest release (MSL41).

In the June 2026 archive, and in the live NCBI Taxonomy service checked on 10 June 2026, the abolished ICTV taxa could also still be found in NCBI Taxonomy with taxonomic ranks and labelled as ICTV-accepted names. Examples include the abolished order "*Petitvirales*" (ICTV201907169, ncbitaxon:2732414), still displayed as an order, and the abolished species "*Nepovirus americaense*" (ICTV19911709, ncbitaxon:3047734), still displayed as a species.

In addition, NCBI maintains historical and intermediate taxonomic states to preserve consistency with sequence records. While this approach is essential for data stability, it may introduce discrepancies with the latest ICTV classification and complicate resolution of current accepted names or taxonomy. Furthermore, extensions below ICTV-defined ranks may introduce additional heterogeneity relative to the official taxonomy. As an illustrative example, the former ICTV species taxon name "*Severe acute respiratory syndrome-related coronavirus*" still has an entry conserving this exact name at NCBI known as ncbitaxon:694009, representing an NCBI-specific entry rather than a current ICTV-accepted taxon.

Catalogue of Life and GBIF The Catalogue of Life (COL) [14] and the Global Biodiversity Information Facility (GBIF) [15] provide broad biodiversity integration and global dissemination infrastructures. Both incorporate virus taxonomy derived from ICTV releases, either directly (COL) or through intermediary providers (GBIF via COL). As of June 2026 COL still integrates ICTV MSL38 and is three full releases behind ICTV [16].

These platforms offer programmatic access and facilitate interoperability across biodiversity datasets. However, their update cycles are independent of ICTV, which may result in delays relative to the latest official taxonomy. In addition, their data models generally prioritise current classifications and may provide limited support for explicit representation of historical terms or systematic resolution of obsolete names. When intermediary providers are involved, additional latency and transformation layers may further affect consistency with the source taxonomy.

1.3.3 Downstream consumers of virus taxonomy

Resources such as ViralZone [17], EVA (European Virus Archive) [18], GenBank, ENA, and Virus-Host DB [19] incorporate taxonomic information about viruses primarily for data organisation, annotation, and discovery. These systems either integrate ICTV taxonomy directly from source files or rely on other providers (e.g. NCBI Taxonomy or other intermediaries) for classification. As a result, their taxonomic representations depend both on their own update cycles and on those of their upstream providers, along with the associated limitations. While they provide essential domain-specific functionality, they generally do not aim to expose the full structure, history, or evolution of virus taxonomy in a form suitable for programmatic reconciliation across releases.

A distinct case is Wikidata [20], which integrates and links heterogeneous data from multiple sources within a knowledge graph framework. While this enables rich cross-resource connections, taxonomic entities originating from different sources or time points may coexist without explicit, machine readable links reflecting their historical relationships. For example, Wikidata may contain both the former ICTV taxon species "Zika virus" (ICTV19990862, wikidata:Q202864) and the current corresponding ICTV taxon species "Orthoflavivirus zikaense" (ICTV19990862, wikidata:Q118455511) as separate entities representing the same species, without indicating deprecation or providing a systematic mechanism to resolve their taxonomic continuity.

1.3.4 Synthesis and implications

This analysis highlights that, although ICTV taxonomy is widely disseminated and reused across multiple platforms, significant heterogeneity remains in how it is represented, updated, harmonised and accessed in data resources [21]. In particular, differences are observed in update latency relative to ICTV releases, persistence and interpretation of identifiers, and support for historical term resolution.

No single resource currently provides a unified solution combining:
(i) up-to-date alignment with ICTV releases,
(ii) explicit and machine-actionable representation of taxonomic history, and
(iii) standardised programmatic access enabling automated resolution of obsolete or historical terms.

The ontology-based infrastructure presented in this work addresses these limitations by providing a unified, semantically explicit, and historically traceable representation of ICTV taxonomy, together with a programmatic access layer through the OLS API. This approach enables consistent resolution of taxonomic entities across releases and facilitates integration of ICTV taxonomy into computational workflows.

1.4 Supplementary Material S4 – ICTV-NCBI mapping specification

Interoperability between ICTV and NCBI taxonomy is supported through an ICTV-NCBI mapping compiled and published, in SSSOM format [22] in the EVORA virus-taxonomy-mappings repository [23]:

https://github.com/EVORA-project/virus-taxonomy-mappings

The mapping release used in this work is release-3, archived in Zenodo [24].

The mapping file (ictv_ncbitaxon_exact.sssom.tsv) contains exact lexical matches between ICTV taxa, represented as ICTV CURIEs, and corresponding NCBI Taxon identifiers. The file header declares the CURIE map, license, mapping provider, mapping set identifier, and mapping set title. At row level, the mapping table includes the subject and object identifiers and labels, the predicate, the mapping justification, the mapping tool/provider, and the mapping date.

Mappings are expressed using skos:exactMatch [25], and the justification is indicated as semapv:LexicalMatching. The repository URL is identified as the mapping provider tool that generated the mapping, and each mapping row includes a mapping date. These elements provide basic mapping metadata and document the origin and method of the generated correspondences.

A mapping layer in the helper libraries (see Supplementary Material S2) loads this SSSOM file and supports both ICTV → NCBI and NCBI → ICTV lookups. This enables downstream resources to align locally stored NCBI-based annotations with official ICTV taxonomy and *vice versa*.

Because the mappings are expressed in the SSSOM standard, they can also be reused by generic ontology-mapping tooling and broader cross-mapping services, strengthening interoperability across the life-science ontology ecosystem.

2 References

1. EMBL-EBI. OLS4 server documentation. 2026. https://github.com/EBISPOT/ols4/blob/dev/README.md. Accessed 09 June 2026.

2. EMBL-EBI. OLS4 API documentation. 2026. https://www.ebi.ac.uk/ols4/api-docs. Accessed 09 June 2026.

3. EVORA Project. EVORA ICTV ontology helper and API documentation. 2026. https://evora-project.github.io/ictv-ontology/helpers/. Accessed 09 June 2026.

4. EVORA Project. EVORA ICTV helper libraries GitHub directory. 2026. https://github.com/EVORA-project/ictv-ontology/tree/main/helpers. Accessed 09 June 2026.

5. EVORA Project. EVORA ICTV Taxon Resolver GitHub repository. 2026. https://github.com/EVORA-project/ictv-resolver. Accessed 09 June 2026.

6. EVORA Project. EVORA ICTV Taxon Resolver live interface. 2026. https://evora-project.github.io/ictv-resolver. Accessed 09 June 2026.

7. International Committee on Taxonomy of Viruses (ICTV). ICTV website. 2026. https://ictv.global/. Accessed 09 June 2026.

8. International Committee on Taxonomy of Viruses (ICTV). ICTV Master Species List (MSL). 2026. https://ictv.global/msl. Accessed 09 June 2026.

9. International Committee on Taxonomy of Viruses (ICTV). ICTV Virus Metadata Resource (VMR). 2026. https://ictv.global/vmr. Accessed 09 June 2026.

10. National Center for Biotechnology Information (NCBI). GenBank. 2026. https://www.ncbi.nlm.nih.gov/genbank/. Accessed 09 June 2026.

11. National Center for Biotechnology Information (NCBI). NCBI Taxonomy Browser. 2026. https://www.ncbi.nlm.nih.gov/datasets/taxonomy/browser/. Accessed 09 June 2026.

12. European Nucleotide Archive (ENA). ENA Browser. 2026. https://www.ebi.ac.uk/ena/browser/home. Accessed 09 June 2026.

13. National Center for Biotechnology Information (NCBI). NCBI Taxonomy archive (taxdump archive). 2026. https://ftp.ncbi.nih.gov/pub/taxonomy/taxdump_archive/. Accessed 09 June 2026.

14. Catalogue of Life. Catalogue of Life (COL). 2026. https://www.catalogueoflife.org/. Accessed 09 June 2026.

15. GBIF. Global Biodiversity Information Facility website. 2026. https://www.gbif.org/. Accessed 09 June 2026.

16. Catalogue of Life. ICTV Master Species List dataset. 2026. https://www.catalogueoflife.org/data/dataset/1014. Accessed 09 June 2026.

17. ViralZone. ViralZone database. 2026. https://viralzone.expasy.org/. Accessed 09 June 2026.

18. European Virus Archive (EVA). EVA website. 2026. https://www.european-virus-archive.com/. Accessed 09 June 2026.

19. Virus Host DB. Virus Host DB. 2026. https://www.genome.jp/virushostdb/. Accessed 09 June 2026.

20. Wikidata. Wikidata. 2026. https://www.wikidata.org/. Accessed 09 June 2026.

21. Seah BKB. Paying it forward: crowdsourcing the harmonisation and linking of taxon names and biodiversity identifiers. Biodivers Data J 2023;11:e114076. doi:10.3897/BDJ.11.e114076

22. Mapping Commons. Simple Standard for Sharing Ontological Mappings (SSSOM), version 1.0. 2026. https://mapping-commons.github.io/sssom/1.0/. Accessed 09 June 2026.

23. EVORA Project. EVORA virus-taxonomy-mappings GitHub repository. 2026. https://github.com/EVORA-project/virus-taxonomy-mappings. Accessed 09 June 2026.

24. McLaughlin J, Lieutaud P. EVORA-project/virus-taxonomy-mappings: Virus Taxonomy Mappings Update. Version release-3 [Software]. Zenodo 2026. doi:10.5281/zenodo.21890424

25. Miles A, Bechhofer S. SKOS simple knowledge organization system reference. W3C Recommendation 2009. https://www.w3.org/TR/skos-reference/. Accessed 12 May 2026.

Manuscript final version
Click here to access/download;Manuscript;GIGA-D-26-00217_updated_manuscript.docx

Programmatic access to ICTV virus taxonomy through a public ontology API

Authors: Philippe Lieutaud[1]*, James McLaughlin[2], R. Curtis Hendrickson[3], Romain David[4], Helen Parkinson[2], Elliot J. Lefkowitz[3], Donald M. Dempsey[3], Bruno Coutard[1]

[1] European Viral Outbreak Response Alliance (EVORA); unité des virus émergents - UVE - UMR 190 - Aix Marseille Université, IRD 190, INSERM U1207, France

[2] European Bioinformatics Institute (EMBL-EBI), European Molecular Biology Laboratory, Wellcome Trust Genome Campus, Hinxton, Cambridge CB10 1SD, United Kingdom

[3] International Committee on the Taxonomy of Viruses (ICTV); University of Alabama at Birmingham, Department of Microbiology, Birmingham, Alabama 35294, United States

[4] European Research Infrastructure on Highly Pathogenic Agents (ERINHA AISBL), 98 rue du Trône, B-1050 Bruxelles, Belgium

Corresponding author: Philippe Lieutaud, Philippe.Lieutaud@univ-amu.fr

Author emails: Philippe.Lieutaud@univ-amu.fr; jmcl@ebi.ac.uk; curtish@uab.edu; romain.david@erinha.eu; parkinso@ebi.ac.uk; elliotl@uab.edu; ddempsey@uab.edu; bruno.coutard@univ-amu.fr

ORCIDs: Philippe Lieutaud [0000-0002-5080-3456]; James McLaughlin [0000-0002-8361-2795]; R. Curtis Hendrickson [0000-0001-6986-4630]; Romain David [0000-0003-4073-7456]; Helen Parkinson [0000-0003-3035-4195]; Elliot J. Lefkowitz [0000-0002-

4748-4925]; Donald M. Dempsey [0000-0002-2200-5828]; Bruno Coutard [0000-0002-2859-7123].

Abstract

Background

The International Committee on Taxonomy of Viruses (ICTV) is responsible for developing and maintaining a universal virus taxonomy. As the reference framework for organising the viral world, it is essential for virology and related fields. Despite its widespread use in research and public health, programmatic access to ICTV taxonomy has remained limited, posing challenges for integration, versioning, and interoperability across databases and bioinformatics resources requiring up-to-date virus taxonomy.

Findings

To address this, we developed a public and sustainable solution leveraging ontology-based APIs. All available ICTV Master Species List (MSL) releases, from MSL1 to MSL41, were transformed into a unified, semantically structured ontology comprising more than 195,000 current and historical entities and deployed through the Ontology Lookup Service (OLS). The ontology is automatically rebuilt and republished whenever a new MSL release becomes available. Complementary ICTV-NCBI mappings and helper libraries support integration into downstream systems.

Conclusions

Together, these resources enable, for the first time, public programmatic retrieval of current and historical ICTV taxon names, taxonomic relationships, metadata, and persistent identifiers through stable endpoints, including resolution of former taxonomic terms to their current accepted taxon or taxa and retrieval of taxon histories across releases. More broadly, this work illustrates a general strategy for transforming structured biological datasets into semantically enriched graph resources exposed through scalable public APIs. These developments enhance interoperability, reduce manual curation, and support FAIR-aligned taxonomic data management in virology and pandemic preparedness.

Keywords

ICTV; Virus taxonomy; Ontology; API; FAIR principles; Interoperability

Key points

- ICTV provides the official taxonomy for classifying viruses and naming virus taxa, but lacks standardised programmatic access.
- Transforming ICTV data into an ontology enables semantic, machine-actionable access across releases via ontology-based APIs.
- ICTV-NCBI mappings support interoperability across bioinformatics resources.
- The integrated ontology-based resource enables programmatic resolution of current and historical viral taxa.

- This approach provides a reusable model for exposing biological datasets through public APIs.

Background

The International Committee on Taxonomy of Viruses (ICTV) [1], established under the Virology Division of the International Union of Microbiological Societies (IUMS) [2], is the authoritative body responsible for the classification of viruses and naming of virus taxa, ensuring a standardised and commonly accepted nomenclature. Virus taxonomy provides a structured framework for classifying viruses based on evolutionary relationships, shared biological properties, and genomic features, such as genetic material, structural features, replication strategies and host range. While everyday virus names function as vernacular labels that can vary across studies and contexts, the ICTV taxon names provide formal, standardised designations that ensure unambiguous communication across the scientific community [3].

The regularly updated ICTV Master Species Lists (MSLs) [4] reflect ongoing advances in virology, including classification of newly discovered viruses and refined taxonomic insights, with one major new release each year, sometimes complemented by minor releases [5]. As virus discovery accelerates, particularly through metagenomics, maintaining consistent and up-to-date taxonomic references in databases and bioinformatics tools relying on ICTV taxa becomes increasingly important.

While the ICTV releases provide authoritative reference data, their distribution format is primarily designed for human consultation and does not readily support programmatic

access [6]. As a result, integrating ICTV taxonomy updates into third-party computational systems remains challenging. Broader taxonomic platforms such as NCBI Taxonomy (National Center for Biotechnology Information) [7], GBIF (Global Biodiversity Information Facility) [8], COL (Catalogue of Life) [9] and Wikidata [10] embed ICTV releases as the authoritative source for virus taxonomy. However, these platforms often incorporate ICTV updates with substantial delays and may lack systematic support for resolving historical taxonomic changes.

Downstream resources can lag by one or more ICTV taxonomic updates, resulting in discrepancies in the classification they expose. Such inconsistencies create significant challenges for users who require the most up-to-date virus classifications, potentially affecting research accuracy and data consistency. For example, outdated species names may persist for some time in major sequence repositories such as GenBank [11] and the European Nucleotide Archive (ENA) [12], leading to further propagation of downstream inconsistencies. Since these repositories rely on the NCBI Taxonomy as their primary taxonomic reference, such delays can be observed by comparing historical taxonomic archives of NCBI and ICTV. A concrete example is ICTV renaming "*Severe acute respiratory syndrome-related coronavirus*" to "*Betacoronavirus pandemicum*" in MSL39. This change did not appear in NCBI Taxonomy for more than a year, and, when it did, was represented by the creation of a new NCBI taxon, rather than a direct renaming of the existing taxa. Obsolete taxon names that continue to be treated as current references complicate reproducibility and hinder interoperability across resources. These problems are inevitable, given that NCBI is not simply embedding ICTV taxonomy, but integrating it within a broader, sequence-linked system whose

update process must preserve continuity for existing records and dependent workflows. As such, ICTV remains the authoritative source for virus taxonomy, while NCBI serves as a widely used integration layer. Consequently, taxon abolitions, splits, merges, moves, and renames may require specific handling rather than simple replacement. Such cases illustrate why programmatic resolution mechanisms are needed [13], along with traceable ICTV identifiers.

The European Viral Outbreak Response Alliance (EVORA) project [14] is a European initiative (2024-2026; grant No. 101131959) [15] that aims to enhance pandemic preparedness and response by strengthening research coordination, regulatory alignment, and data sharing among EU Research Infrastructures. Improving universal adoption of accurate and up-to-date virus taxonomy represents an important component of this mission. Members of EVORA and ICTV collaborated to provide a structured, machine-actionable version of the official ICTV taxonomy using ontology-based APIs, with support for historical name resolution, revision tracking and cross-system identifiers.

Many biological data resources are distributed as relational databases, spreadsheets or tabular datasets that are well suited for storage, but less adapted to programmatic integration across heterogeneous systems. In addition to addressing the immediate need for improved access to ICTV taxonomy, this work illustrates a broader methodological approach for transforming structured database content into semantically enriched knowledge representations through the use of ontologies. Ontologies play a crucial role in the management, integration, and analysis of biological data by providing explicit, human- and machine-readable semantics that support the consistent annotation, sharing and interpretation of complex datasets [16]. Converting ICTV releases into an

ontology enables graph-based querying and integration. As graph-based representations naturally capture hierarchical relationships, synonyms, and historical changes, representing taxonomy as an ontology therefore enables more expressive queries, automated reasoning, and easier integration with other semantic resources.

Across the life sciences, the Ontology Lookup Service (OLS) provides widely used infrastructure for searching, browsing and accessing biological and biomedical ontologies through standard APIs, supporting the reuse of ontology terms and relations in data curation and computational workflows [17, 18].

Methods

In this paper, “OLS API” refers to the generic programmatic interface provided by the OLS, including its Representational State Transfer (REST) endpoints and controllers. “ICTV ontology API” refers to access to the ICTV ontology through the OLS API, either directly via OLS endpoints or indirectly through ICTV-specific helper libraries built on top of them.

Design requirements and use cases

To support practical integration of ICTV taxonomy into data systems and analytical workflows, several key use cases were defined to address common end-user challenges for automated processing. First, it must provide historical resolution by mapping obsolete names, synonyms, and ICTV identifiers from previous ICTV releases to their corresponding taxa in the current ICTV classification. Second, taxonomic

changes must be tracked across releases, including taxa that have been abolished, split, merged, renamed, or reassigned (promoted) within the hierarchy. Third, cross-resource mapping must be supported, and, specifically, a mapping, with provenance, between NCBI Taxonomy identifiers and ICTV identifiers (historical and current) must be provided. Finally, there must be a stable, public, well-documented API for performing these functions.

Persistent identifiers

Persistent identifiers are key for data integration [19]. The ICTV defines persistent identifiers for all versions of all taxa, as well as links connecting the taxa into hierarchies and documenting changes between releases. These unique identifiers facilitate resolution of outdated taxonomic terms to current ones by allowing taxa to be efficiently tracked across renames, rank changes, merges, splits, and abolitions between releases. To support programmatic resolution and cross-release linkage, and to further support FAIR principles, ICTV publicly documented these existing identifiers and defined Compact Uniform Resource Identifiers (CURIEs) for them. ICTV provides three principal identifiers. In the shorthand used below, the letter prefix indicates the identifier type and # represents the numeric identifier value:

- Taxnode identifier (Taxnode ID; TN#): uniquely identifies a taxon node within the ICTV source database in a specific release.
- ICTV identifier (ICTV ID; ICTV#): identifies the same taxon across releases, including through renames, changes in location within the taxonomic tree

(moves), and changes in rank (promotion/demotion). Splits and merges create new ICTV IDs, which are linked back to their antecedent taxa.

- Isolate identifier (Isolate ID; VMR#): defined by the Virus Metadata Resource (VMR) [20], links GenBank sequence accessions for one or more segments to the vernacular name and the corresponding viral species [6].

During ontology generation, TN identifiers are used as ICTV source-table keys to reconstruct parent–child relationships within each release, associate VMR records with taxon nodes and interpret changes between releases. They are not included in the ontology because their role is limited to processing the ICTV source data. Instead, each release-specific taxon state is represented as an OWL class and is assigned a resolvable IRI combining the MSL release and the persistent ICTV ID (for example, the MSL41 representation of ICTV20040588 [21]). This design uniquely identifies each release-specific taxon state, preserves direct resolution across simple renames and uses explicit revision and replacement relations when taxonomic events create new ICTV IDs.

This identifier design underpins the modelling of ICTV releases as semantically linked ontology resources. An extremely abridged example of the history of a taxon related to the genus *Baculovirus* in Figure 1 illustrates some of the complexity, and demonstrates how ICTV IDs link historic versions of a taxon, from the first ICTV release to the current MSL41 release [22].

Initial modelling of ICTV releases as ontologies

To meet the design requirements and use cases described above, including historical resolution, cross-release tracking and programmatic querying, each of the 41 (as of 2026) official ICTV taxonomy updates since 1971 was modelled as an independent OWL2 ontology, representing hierarchies, ranks, synonyms and revision metadata. Taxa were modelled as OWL classes and virus isolates as OWL named individuals. This approach captures taxonomic relationships and their evolution over time, while preserving the structural and semantic fidelity of the original ICTV data in a machine-readable format.

The ontologies were constructed using established semantic standards and interoperable vocabularies commonly used in biomedical ontology ecosystems (e.g. [23], built upon semantic web standards including OWL, RDFS, PROV-O [24] and SKOS semantic relations [25], together with complementary vocabularies such as Dublin Core Terms (DCTERMS), Friend Of A Friend (FOAF), the Taxonomic Rank Vocabulary (TAXRANK) and oboInOwl ontology annotations. This supports compatibility with the broader life-science ontology ecosystem [26] and reuses selected terms and annotations from OBO-associated resources [27]. Where a replacement exists, deprecated historical OWL classes are linked to their replacements using IAO:0100001 (term replaced by), enabling rapid resolution of former terms to their current representations.

The OLS enables direct exposure of ontology data through stable REST API controllers, a web-based user interface [28], and a Model Context Protocol (MCP) server for access

by AI agents. OLS provides a ready-to-use infrastructure to host ontologies and deliver programmatic access through standardised endpoints [17]. This approach eliminates the need to develop and maintain custom API infrastructures while ensuring compatibility with the broader ecosystem of life science ontologies. Thus, transforming existing data into a semantically structured graph representation leverages the OLS infrastructure to efficiently provide standard, public APIs and interoperability across data infrastructures.

The set of release-specific ontologies was published via OLS. The OLS API enables dynamic querying, retrieval of data and linking across ontologies, allowing applications to access up-to-date information directly from ICTV data via the ontology stored in OLS.

Redesign as a unified ontology

However, querying multiple ontologies proved complex for the primary use case of reconciling historical references with the current taxonomy. The system was therefore redesigned to merge all releases into a single, unified ontology (over 195,000 terms in 2026). In this model, the most recent ICTV release represents the authoritative classification, while entities from previous releases are preserved as deprecated historical OWL classes and, where applicable, linked through explicit replacement relations. This design reduces query complexity, while preserving full history, provenance and backward compatibility across ICTV releases. This enables any taxonomic reference appearing in historical datasets or publications to be programmatically reconciled with the current ICTV classification.

The ontology is primarily functional: it was engineered to expose current and historical ICTV taxonomy through OLS and the helper libraries so that taxon identity, lineage, synonyms and revision history can be queried programmatically. It does not import BFO, COB or RO, has not been submitted to the OBO Foundry, and no submission is currently planned.

Figure 2 illustrates how the entities and relations in the unified ontology support taxon resolution across ICTV releases. Release-specific taxon representations, modelled as OWL classes, are connected through PROV revision relations and, when a replacement is defined, through IAO:0100001 to the corresponding OWL class in the latest MSL, which represents the currently accepted ICTV taxon. In simple taxon rename paths, reusing the stable ICTV ID with the latest MSL release also retrieves the current OWL class, whereas IAO:0100001 provides a more direct route to the replacement OWL class and can resolve replacement or merge events without traversing the full release history. VMR virus exemplar records modelled as OWL named individuals are linked by rdf:type to their corresponding species OWL classes in the ontology. Together, these relations allow identifiers and current or former taxon or virus names to be resolved to current taxa, to defined historical states or to complete taxon histories through the OLS API and helper libraries.

Availability and sustainability

Continuous Integration and Deployment (CI/CD) workflows implemented through public GitHub repositories automatically trigger the ontology generation workflow for each release published in the ICTV's official GitHub repository [29]. This automation removes

manual intervention and contributes to long-term sustainability of the infrastructure, in line with recommendations for sustainable FAIR implementation in Life Sciences [30]. The unified ontology is immediately available as a release from the EVORA GitHub repository [31], and is automatically deployed to the OLS, typically within 24 hours of an ICTV release, which provides public web browsing and API access. Dissemination and long-term discoverability are further supported through versioned GitHub and Zenodo releases, as well as registration of the software resources in bio.tools and SciCrunch (see Availability of supporting source code and requirements section).

ICTV-NCBI mapping

To improve interoperability at the broader scale [32], ICTV-NCBI mappings are generated using the OLS API, and stored in SSSOM (Simple Standard for Sharing Ontology Mapping) format, aligning ICTV identifiers with NCBI Taxon identifiers [33]. The code to generate this mapping is maintained in a dedicated GitHub repository [34], where the resulting SSSOM files are then published as releases (see Additional file 1, Supplementary Material S4). While this analysis is also automated using a GitHub CI/CD workflow, it runs on a schedule, decoupled from the release cycles of the two ontologies, allowing independent updates while remaining consistent with the continuously updated ontology infrastructure.

These mappings not only facilitate direct and bidirectional linkage between ICTV and NCBI taxonomies, but also enable alignment across downstream resources that rely on NCBI as a taxonomic backbone, including sequence repositories and bioinformatics platforms. This feature enables broader platforms that rely on the NCBI taxonomy for

viral references (e.g., ENA) to stay current with the ICTV taxonomy while continuing to leverage the broader coverage of NCBI taxonomy.

The NCBI integrates the ICTV Taxonomy into its own taxonomy system, which is used to designate species related to the entries in its sequence databases (e.g. GenBank). This integration is supported by an established exchange process between the ICTV database team and the NCBI team responsible for virus taxonomy updates. This process predates the present work and accommodates NCBI's specific sequence-linked update requirements. The public ICTV ontology API and ICTV-NCBI mappings complement this process by providing a general, community-facing and machine-actionable layer for accessing and reconciling current and historical ICTV taxonomy. Historically, ICTV updates have been incorporated with roughly a one-year delay, resulting in NCBI being one or two releases behind the latest ICTV version (as described above). Observations following MSL41 release suggest a recent effort by NCBI to shorten this delay, with progressive integration starting in May 2026, about two months after the MSL41 release. This included the integration of renamed taxa, while newly created taxa remained absent and abolished taxa had not yet been removed (see Additional file 1, Supplementary Material S3). As part of this work, ICTV has publicly documented several persistent identifiers (described above) that link taxa in successive ICTV Taxonomy versions, which should facilitate rapid integration of new releases. These identifiers are exposed in the ICTV ontology metadata and support the generation of SSSOM ICTV-NCBI mappings. In the released mapping file, lexical ICTV-to-NCBI Taxon correspondences are expressed with skos:exactMatch, enabling

alignment between ICTV taxonomy and NCBI Taxonomy identifiers used by downstream sequence resources such as GenBank.

Results

Unified ontology for ICTV taxonomy and OLS deployment

We have produced a unified ontology for the ICTV taxonomy, aggregating all releases into a single resource. The ontology is available for download or via OLS and enables programmatic access to current and historical classifications. Ontology updates are automatically triggered by new ICTV releases and reflected in OLS, typically within 24 hours.

Asynchronously, ICTV-NCBI mappings are generated in SSSOM format, enabling bidirectional resolution between the taxonomies. Mappings are maintained in a dedicated EVORA virus-taxonomy-mappings repository and updated independently [34]. The mapping supports bidirectional many-to-many resolution between ICTV and NCBI taxonomies and facilitates integration with downstream resources. The overall architecture of the generation and publication workflow is shown in **Figure 3**.

To simplify integration into downstream applications, ICTV-specific helper libraries were developed on top of the standard OLS API. These libraries are provided in JavaScript, Python and PHP and encapsulate resolution logic (synonyms, obsolescence, lineage, mappings) while returning normalised data about taxon objects. These objects contain identifiers, labels, synonyms, ranks, lineage, obsolescence metadata and cross-references, enabling application developers to integrate ICTV-aware logic without direct

manipulation of raw API calls. The helper libraries prioritise exact identifier and name-based routes before free-text fallback. A bare ICTV ID returns the corresponding OWL class from the latest matching MSL release by default, while an explicit MSL release retrieves the corresponding historical state. For VMR records represented in the ontology, a VMR identifier resolves through the corresponding OLS individual and its rdf:type relation to the current species OWL class.

The OLS API documentation, ICTV-specific guidance and helper libraries are publicly available [35] (see Additional file 1, Supplementary Material S1). Together, the unified ontology, its OLS deployment, the helper libraries and the complementary SSSOM mappings constitute an integrated ontology-based access resource for automated resolution, cross-taxonomy linkage and reproducible versioning.

Reference application and early adopters

A public demonstration interface, the ICTV Taxon Resolver [36] web application, was developed as a reference implementation using the JavaScript helper library. The interface provides two selectable actions. The default action, ‘Resolve to latest ICTV’, returns the current accepted ICTV taxon when one can be resolved. ‘Show history across MSLs’ starts from the OWL class representing the taxon identified from the query and displays the release-specific OWL classes linked to it across the MSL releases included in the ontology. Consequently, the displayed history may begin after MSL1 or end before MSL41, for example when a taxon was introduced later or subsequently abolished. The source code is available from the EVORA ICTV Resolver GitHub repository [37]. The resolver allows users to submit current and historical ICTV

taxon names and virus names, ICTV identifiers, Internationalized Resource Identifiers (IRIs) or NCBI Taxon IDs and receive the corresponding currently accepted ICTV taxon or taxa, together with their full lineages and revision history. Concise usage guidance is displayed directly beneath the interface title. This tool illustrates practical applicability while serving as a functional community resource. The interface and an example resolution of the former species name *Zika virus* to its post-2022 name and lineage, *Orthoflavivirus zikaense*, are shown in **Figure 4**. For example, in history mode, the relationship between *Alphabaculovirus* and *Nucleopolyhedrovirus* illustrated in Figure 1 can be traced from MSL23 back to MSL13. The resolver is a demonstration and reference implementation built on the JavaScript helper library and does not expose every capability of the OLS-backed ICTV ontology API, for which complete developer documentation is available separately.

Among the first operational deployments using the ICTV ontology API is the EVORA Portal [38], a consolidated catalogue of research infrastructures' resources and services in virology designed to support outbreak response and pandemic preparedness. The utilisation of the ICTV ontology API is integrated through one of the helper libraries directly into the catalogue generation workflow, enabling retrieval of up-to-date taxonomic terms along with associated lineage and synonym information. This ensures consistent use of the latest ICTV taxonomy, even when metadata provided by contributing research infrastructures correspond to earlier ICTV releases. As an added value for users of the research infrastructures, lineage information and historical synonyms retrieved through the ontology API enable enhanced search capabilities. This

allows users to find resources using outdated taxa or alternative names, an option that was not available in the former search module.

Early adoption of the ICTV ontology API has also been observed in an external initiative concerned with pandemic preparedness and data integration: the Pandemic PACT programme [39]. This programme is now using ICTV taxonomy via the OLS API to align viral pathogen families and scientific names within its scope with the latest ICTV taxonomy (see Acknowledgements section). The retrieved ICTV metadata will be incorporated into Pandemic PACT dashboard visualisations [40], where they will be integrated with metadata from other ontologies accessed through the OLS, such as the Systematized Nomenclature of Medicine (SNOMED), aligning up-to-date viral pathogen taxonomy with related information, including associated diseases.

Integration of ICTV taxonomy through an API enables timely and automated updates across resources. The European Virus Archive (EVA) [41], a network of distributed virus collections using a single web catalogue for virus and virus-related material ordering and distribution [42], plans to integrate the ICTV ontology into its workflows using OLS API queries to maintain local references to virus taxonomy aligned with the most recent ICTV releases. This will allow EVA datasets to remain synchronised with official taxonomy updates while preserving historical annotations associated with archived materials, which will enhance the interconnectivity of research and data management processes, improving resource discovery and data consistency.

These deployments demonstrate practical applicability across research infrastructures and public health platforms. The system enables automated resolution, cross-taxonomy linkage, reduced manual curation and reproducible versioning.

Discussion

Virus data resources include numerous databases with different scopes, data models, functionalities and degrees of FAIR maturity, as reflected in recent reviews of virus databases [43]. This heterogeneity creates practical challenges for interoperability and synchronisation with authoritative taxonomic references such as ICTV taxonomy. This work demonstrates how relational datasets can be easily transformed and exposed through scalable, public, ontology-based APIs. Standards-based ontology-enabled APIs drastically reduce the work needed to make datasets machine-actionable. Here, we developed an ontology for the widely used and regularly updated ICTV virus taxonomy and exposed it through a public API, providing downstream systems with programmatic access to current and historical taxonomic information and the ability to resolve former terms to the current classification, thereby supporting uses ranging from direct queries to recurring automated updates. The ontology infrastructure acts as a semantic resolution layer that continuously bridges evolving authoritative ICTV releases with downstream computational ecosystems and legacy taxonomic references. Timely updates, in turn, reduce discrepancies that arise when downstream systems rely on outdated or partially synchronised taxonomic references and enable reproducible taxonomic resolution across computational workflows. Research infrastructures, sequence repositories, biodiversity catalogues, and knowledge bases can use

automated alignment mechanisms to maintain consistency with official ICTV classifications, reducing the need for extensive manual curation.

Persistent ICTV identifiers and replacement relations provide a structured mechanism to track taxonomic evolution, historically prone to ambiguity and data fragmentation, in a computationally accessible way. Complementary ICTV-NCBI mappings, together with ontology cross-references to associated sequence resources, provide a pragmatic bridge between the authoritative, annually updated ICTV classification and NCBI taxonomy. This improves reproducibility and supports FAIR principles, and their progressive implementation [44, 45, 46]. While this approach substantially improves interoperability and historical resolution, synchronisation across downstream resources still depends on heterogeneous update cycles and integration strategies. In addition, some complex taxonomic transitions, such as splits, merges, or taxon abolitions, may still require complementary expert interpretation in specific downstream analytical contexts.

These capabilities help connect distributed data systems in FAIR-aligned federated environments, where independently maintained resources must remain discoverable, accessible and interoperable, including initiatives such as the European Open Science Cloud (EOSC) and the European Health Data Space (EHDS).

Consistent and machine-actionable taxonomy references support large-scale computational analyses, automated data integration workflows and reproducible comparative studies by reducing ambiguity in taxonomic interpretation. This contributes to improved retrieval and harmonisation of virological data across interoperable

research infrastructures and data-sharing environments [47], supporting more reliable data integration, comparative analyses, evidence-based decision making, and more informed experimental design and data interpretation. The infrastructure also establishes a foundation for future enhancements, including expanded mappings with sequence repositories and the possible development of additional interfaces relying on the public OLS API.

Our approach aligns with the article “Four principles to establish a universal virus taxonomy” [48] that outlines key recommendations to achieve a coherent and comprehensive virus taxonomy. Our work helps make ICTV taxonomy more useful by ensuring that references to the official, evolution-based ICTV taxonomy, can be easily maintained and kept up-to-date, irrespective of the intended use or alternate classification scheme. For example, classifying viruses to support the surveillance and public health response to disease outbreaks (Pathogens prioritization: a scientific framework for epidemic and pandemic research preparedness) [49] results in a classification that is significantly different than that of the ICTV taxonomy. It is however still important to reference the current evolutionary-based classification so that our current knowledge on viruses can be quickly and easily adapted to newly identified or newly emerging ones. The ICTV taxonomy serves as the universal reference for virus classification, while the services we report here enable the critical links needed to connect that taxonomy to alternate schemes.

Adoption of the ICTV ontology API by resources relying on virus taxonomy (EVORA and Pandemic PACT) demonstrates its practical applicability. Ontology-based APIs can facilitate alignment between biological taxonomy resources and clinical terminologies

used in public health systems, enabling consistent pathogen identification across research infrastructures, data catalogues, and policy-oriented dashboards. By supporting automated retrieval of up-to-date taxonomic terms, lineage information and synonym relationships, the API-based approach supports consistent taxonomy usage across distributed data infrastructures and reduces the need for manual synchronisation of taxonomic references similarly to interoperability approaches explored in tools such as Treemendous for reconciling taxonomic backbones in tree species naming [50].

Taxonomic resources and biodiversity platforms such as NCBI Taxonomy and GBIF could use the OLS API to implement more automated synchronisation with ICTV releases. Databases such as ENA and GenBank, which depend on the NCBI taxonomy and its integration of ICTV classifications [51], would in turn benefit from improved data integration and accuracy. In cases where updates of virus taxonomy within NCBI do not immediately reflect the latest ICTV release, these resources could additionally leverage the ICTV ontology API, coupled with the provided SSSOM mappings, to maintain up-to-date virus taxonomy.

Our work builds on established FAIR and semantic standards [26, 52]. The inclusion of detailed metadata and the reuse of established semantic standards are critical for ensuring that the ICTV taxonomy serves as a reliable reference across different platforms and applications. This approach provides a lightweight and sustainable mechanism for publishing structured datasets as interoperable APIs, by leveraging an existing ontology service (OLS) and GitHub's publicly available workflow automation. This approach is applicable to other domain-specific datasets. In parallel with the public OLS-based service, ICTV is using a dedicated access mechanism for data exchange

with NCBI and plans a broader API providing access to a more comprehensive ICTV dataset. These complementary services support specialised or higher-volume exchanges, while the OLS API remains the public, standards-based access layer.

More broadly, this work illustrates how ontology technologies can expose structured datasets as semantically enriched graph representations through interoperable APIs. Traditional database schemas are optimised for storage and transactional operations but often require custom APIs to expose their content programmatically. In contrast, ontology-based representations allow relationships between entities to be explicitly encoded and navigated as a graph, enabling more flexible data integration and discovery. Such representations align with knowledge graph and Linked Open Data (LOD) approaches [53, 54]. More generally, this work demonstrates a reusable pattern for exposing structured, domain-specific datasets as interoperable ontologies and APIs by leveraging established ontology infrastructures, such as the OLS for biomedical ontologies.

Conclusion

This work shows that representing an evolving classification system, such as the ICTV taxonomy, as a unified ontology hosted through a public, scalable, standards-based API, increases its accessibility and addresses key challenges in access, versioning and interoperability. The resulting infrastructure supports backward-compatible resolution of virus taxonomy across historical ICTV releases, enabling historical traceability and

alignment with current classifications through semantic modelling, persistent identifiers and interoperable mappings.

By building on open standards, automated workflows and existing ontology infrastructure services, this approach supports long-term sustainability and scalability while making ICTV taxonomy more readily integrable into computational workflows. It also facilitates synchronisation between authoritative taxonomic references and downstream biological, biomedical and public-health data infrastructures, supporting broader adoption of the latest official ICTV taxonomy as the reference standard and strengthening data consistency, reproducibility and preparedness for emerging viral threats, including through more timely dissemination of high-impact updates such as the pandemic-based designation of the species *Betacoronavirus pandemicum*.

Future developments may support broader interoperability with additional taxonomy providers, sequence repositories and interconnected semantic resources. More broadly, this work provides a reusable model for maintaining, exposing and reusing evolving taxonomic references through sustainable, machine-actionable semantic infrastructure.

Declarations

Not applicable.

Consent for publication

Not applicable.

Funding

This work was supported by the EVORA project (European Union's HORIZON programme grant agreement N° 101131959). The contribution of the University of Alabama at Birmingham (UAB) team to the research reported in this publication was supported by the National Institute of Allergy and Infectious Diseases of the National Institutes of Health under Award Number U24AI162625. The content is solely the responsibility of the authors and does not necessarily represent the official views of the National Institutes of Health. The UAB team assisted with accessing all viral taxonomic data and associated metadata. The UAB team also served as consultants to assist in the understanding of the underlying ICTV data structures and data organization that were used to construct the ontology. In total, the UAB effort comprised approximately 10% of the overall effort.

Acknowledgements

This work is a product of the EVORA project (European Viral Outbreak Response Alliance) and the ICTV’s UAB team.

We gratefully acknowledge Emilia Antonio and Alice Norton from the Pandemic PACT programme for their feedback on the adoption of the ICTV OLS API within Pandemic PACT to align viral pathogen families and scientific names with the latest ICTV taxonomy identifiers.

Disclosure of use of AI-assisted tools including generative AI

- During manuscript preparation and revision in 2024-2026, Philippe Lieutaud, Romain David and R. Curtis Hendrickson used OpenAI ChatGPT, initially with GPT-4o and subsequently with GPT-5-series models, including GPT-5.4, GPT-5.5 and GPT-5.6, at different stages as a writing and editorial aid [55]. The tool was used to suggest rewording and stylistic improvements, identify redundancy, improve the organisation of arguments, check conformity with journal requirements during final review, including reference formatting and the availability of cited links, and suggest improvements to figure layout and presentation. The AI-assisted textual and figure-design suggestions were treated as conceptual proposals and were not incorporated verbatim; no AI-generated images were used. The authors determined and validated all final scientific content, and produced the final figures.

- Philippe Lieutaud also used OpenAI ChatGPT with GPT-5.4 and GPT-5.5 to assist in drafting user and developer documentation published in the ICTV ontology and ICTV Resolver GitHub repositories. He used the same models to assist in porting the ICTV API helper library from Python to JavaScript and PHP, and to check consistency across the three implementations. GPT-5.5 was also used to suggest fixes for Zenodo release synchronisation involving .zenodo.json metadata and new GitHub release events in the ICTV ontology and ICTV-NCBI mapping repositories, and to help design a sharded, failure-resilient strategy for long-running ICTV-NCBI mapping updates. GPT-5.5 also assisted in developing a local script to convert the Word manuscript to LaTeX; Philippe Lieutaud produced and checked the final LaTeX files. The resulting documentation, code

and workflow changes were reviewed and validated by the authors against the intended functionality of the libraries, release processes, mapping workflows and OLS API.

- Separately, on 7 October 2025, James McLaughlin used the GitHub Copilot coding agent (underlying model not recorded) to generate initial GitHub Actions implementations for building and releasing the ICTV ontology and for scheduling updates of the ICTV-NCBI mappings [56]. In the mapping repository, Copilot also drafted associated documentation and an initial modification supporting the incremental reuse of existing mappings. These contributions are preserved in the public development history of the corresponding repositories. The generated code was reviewed and merged by James McLaughlin and subsequently revised and extended by the authors.
- The AI-assisted tools were not used to generate scientific data or independently interpret the results. All generated suggestions and code contributions were critically reviewed and validated by the authors, who take full responsibility for the content of the manuscript and the associated software.

Authors' contributions

Based on Contributor Roles Taxonomy (CRediT) [57]

- Conceptualization: PL
- Project administration: PL, BC

- Resources: CH, EL, PL, BC, JM, HP
- Software: JM, PL
- Methodology: PL, JM, HP, RD, CH
- Writing – original draft: PL, RD
- Writing – review & editing: PL, RD, HP, JM, BC, CH, EL
- Visualization: PL, RD, CH, DMD

Competing interests

The authors declare that they have no competing interests.

Data availability

All ontology artefacts, helper libraries, mapping resources, example notebooks, and continuous-integration workflows are publicly available under open licences. The ICTV MSL41 source data used in this work are available as the ICTVdatabase MSL41.v1 release [22]. The exact EVORA ICTV ontology release used for this manuscript is archived in Zenodo as EVORA-project/ictv-ontology: 2026-Jul-28-74d7801 [58], and its complete version history is available through the corresponding Zenodo concept record [59]. The exact ICTV-NCBI mapping release is archived in Zenodo as EVORA-project/virus-taxonomy-mappings: Virus Taxonomy Mappings Update, release-3 [60], and its complete version history is available through the corresponding Zenodo concept

record [61]. Current development versions and latest releases are available from the ICTV database, ICTV ontology, ICTV-NCBI mapping and unified OLS resources [28], [31], [34], [62].

All resources are reusable, openly licensed and versioned, enabling independent verification and downstream integration.

NCBI Taxonomy taxdump archives used to verify the integration status of ICTV releases are available from the NCBI Taxonomy archive [63].

Availability of supporting source code and requirements

- Project name: ICTV Ontology API and helper libraries
  - Project home page: https://github.com/EVORA-project/ictv-ontology
  - Documentation: https://evora-project.github.io/ictv-ontology/helpers/
  - Operating system(s): Platform independent
  - Programming language: language-agnostic for direct API access; helper libraries are provided in Python 3.8 or later, JavaScript as an ES module, and PHP 7.4 or later.
  - Other requirements: Internet access to the OLS API; depending on the helper library used, the Python requests library, the JavaScript Fetch API

or the PHP cURL extension is required. For PHP, allow_url_fopen must also be enabled when accessing the remote ICTV-NCBI SSSOM mapping.

  - Licences:
    - ontology-generation code, Apache License 2.0;
    - ICTV-derived ontology data, Creative Commons Attribution 4.0 International (CC BY 4.0), with attribution to ICTV;
    - JavaScript, Python and PHP helper libraries, MIT License.
  - Version used for this manuscript: GitHub release 2026-Jul-28-74d7801; Zenodo version DOI 10.5281/zenodo.21506852
  - All versions: 10.5281/zenodo.20610328
  - RRID: SCR_028724
  - bio.tools ID: ictv_ontology_api
- Project name: ICTV-to-NCBITaxon SSSOM mapping
  - Project home page: https://github.com/EVORA-project/virus-taxonomy-mappings
  - Operating system(s): Platform independent
  - Programming language: language-agnostic for SSSOM file utilisation; SSSOM file generation code in Python.
  - Release archived at https://doi.org/10.5281/zenodo.21890424 [60]

    - License: CC0 1.0 Universal
    - RRID: SCR_028725
    - bio.tools ID: ictv_to_ncbitaxon_sssom_mapping
- Project name: ICTV Taxon Resolver
    - Project home page: https://github.com/EVORA-project/ictv-resolver
    - Live instance: https://evora-project.github.io/ictv-resolver
    - Programming languages: HTML, JavaScript and CSS.
    - Other requirements: A modern web browser with JavaScript and internet access to OLS.
    - License: MIT.
    - Version used: v1.0.5.
    - RRID: SCR_028722
    - bio.tools ID: ictv_taxon_resolver
- External service dependency: Ontology Lookup Service (OLS):
    - Project home page: https://www.ebi.ac.uk/ols4/
    - Documentation: https://github.com/EBISPOT/ols4/blob/dev/README.md [64]
    - Source code: https://github.com/EBISPOT/ols4

- Software licence: Apache License 2.0

List of abbreviations

- **API(s):** Application Programming Interface(s)
- **CI/CD:** Continuous Integration and Deployment
- **COL:** Catalogue of Life
- **CURIE:** Compact Uniform Resource Identifier
- **DCTERMS:** Dublin Core Terms namespace
- **ENA:** European Nucleotide Archive
- **ETL process:** Extract, Transform, and Load
- **EVA:** European Virus Archive
- **EVORA:** European Viral Outbreak Response Alliance.
- **FAIR:** Findable, Accessible, Interoperable and Reusable
- **FOAF**: Friend of a Friend ontology
- **GBIF:** Global Biodiversity Information Facility
- **IAO:** Information Artifact Ontology
- **ICTV:** International Committee on Taxonomy of Viruses

- **IRI**: Internationalized Resource Identifier
- **LOD:** Linked Open Data
- **MCP:** Model Context Protocol
- **MSL:** Master Species List
- **NCBI:** National Center for Biotechnology Information
- **OLS:** Ontology Lookup Service
- **OWL:** Web Ontology Language
- **PROV-O**: Provenance Ontology
- **RDFS:** Resource Description Framework Schema
- **REST:** Representational State Transfer
- **SKOS:** Simple Knowledge Organization System
- **SNOMED:** Systematized Nomenclature of Medicine
- **SSSOM:** Simple Standard for Sharing Ontology Mapping
- **TAXRANK:** Taxonomic Rank Vocabulary
- **VMR:** Virus Metadata Resource

References

1. International Committee on Taxonomy of Viruses (ICTV). ICTV website. 2026. https://ictv.global/. Accessed 09 June 2026.

2. International Union of Microbiological Societies (IUMS). IUMS website. 2026. https://www.iums.org/. Accessed 09 June 2026.

3. Zerbini FM, Siddell SG, Mushegian AR, Walker PJ, Lefkowitz EJ, et al. Differentiating between viruses and virus species by writing their names correctly. Arch Virol 2022;167:1231–1234. doi:10.1007/s00705-021-05323-4

4. International Committee on Taxonomy of Viruses (ICTV). ICTV Master Species List (MSL). 2026. https://ictv.global/msl. Accessed 09 June 2026.

5. Simmonds P, Adriaenssens EM, Lefkowitz EJ et al. Changes to virus taxonomy, the international code of virus classification and nomenclature, and the ICTV statutes ratified by the International Committee on Taxonomy of Viruses (2025). *Arch Virol* 2026;171:23. doi:10.1007/s00705-025-06485-1

6. Black EJ, et al. Virus taxonomy: the database of the International Committee on Taxonomy of Viruses. Nucleic Acids Res 2026;54(D1):D776–D789. doi:10.1093/nar/gkaf1159

7. National Center for Biotechnology Information (NCBI). NCBI website. 2026. https://www.ncbi.nlm.nih.gov/. Accessed 09 June 2026.

8. GBIF. Global Biodiversity Information Facility website. 2026. https://www.gbif.org/. Accessed 09 June 2026.

9. Catalogue of Life. Catalogue of Life (COL). 2026. https://www.catalogueoflife.org/. Accessed 09 June 2026.

10. Wikidata. Wikidata. 2026. https://www.wikidata.org/. Accessed 09 June 2026.

11. National Center for Biotechnology Information (NCBI). GenBank. 2026. https://www.ncbi.nlm.nih.gov/genbank/. Accessed 09 June 2026.

12. European Nucleotide Archive (ENA). ENA Browser. 2026. https://www.ebi.ac.uk/ena/browser/home. Accessed 09 June 2026.

13. Boyle B, Hopkins N, Lu Z, et al. The taxonomic name resolution service: an online tool for automated standardization of plant names. BMC Bioinformatics 2013;14:16. doi:10.1186/1471-2105-14-16

14. EVORA. European Viral Outbreak Response Alliance project website. 2026. https://evora-project.eu/. Accessed 09 June 2026.

15. European Commission. European Viral Outbreak Response Alliance (EVORA), grant agreement ID 101131959. CORDIS. 2026. https://doi.org/10.3030/101131959. Accessed 09 June 2026.

16. Malone J, Stevens R, et al. Ten simple rules for selecting a bio-ontology. PLoS Comput Biol 2016;12(2):e1004743. doi:10.1371/journal.pcbi.1004743

17. McLaughlin J, Lagrimas J, Iqbal H, et al. OLS4: a new Ontology Lookup Service for a growing interdisciplinary knowledge ecosystem. Bioinformatics 2025;41(5):btaf279. doi:10.1093/bioinformatics/btaf279

18. EMBL-EBI. Ontology Lookup Service (OLS4). 2026. https://www.ebi.ac.uk/ols4/. Accessed 09 June 2026.

19. McMurry JA, Juty N, Blomberg N, et al. Identifiers for the 21st century: how to design, provision, and reuse persistent identifiers. PLoS Biol 2017;15(6):e2001414. doi:10.1371/journal.pbio.2001414

20. International Committee on Taxonomy of Viruses (ICTV). ICTV Virus Metadata Resource (VMR). 2026. https://ictv.global/vmr. Accessed 09 June 2026.

21. International Committee on Taxonomy of Viruses (ICTV). MSL41 representation of ICTV taxon ICTV20040588. 2026. http://ictv.global/id/MSL41/ICTV20040588. Accessed 02 September 2026.

22. Hendrickson RC, Mims L, Lefkowitz EJ. ICTV-Virus-Knowledgebase/ICTVdatabase: Taxonomy Release: MSL41.v1 20260320 [Dataset]. Zenodo 2026. doi:10.5281/zenodo.19339543

23. Kibbe WA, Arze C, Felix V, et al. Disease Ontology 2015 update: an expanded and updated database of human diseases for linking biomedical knowledge through disease data. Nucleic Acids Res 2015;43(D1):D1071–D1078. doi:10.1093/nar/gku1011

24. Lebo T, Sahoo S, McGuinness D, et al. PROV-O: the PROV ontology. W3C Recommendation 2013. https://www.w3.org/TR/prov-o/. Accessed 12 May 2026.

25. Miles A, Bechhofer S. SKOS simple knowledge organization system reference. W3C Recommendation 2009. https://www.w3.org/TR/skos-reference/. Accessed 12 May 2026.

26. Xu F, Juty N, Goble C, Jupp S, Parkinson H, Courtot M. Features of a FAIR vocabulary. J Biomed Semantics 2023;14(1):6. doi:10.1186/s13326-023-00286-8

27. Smith B, Ashburner M, Rosse C, et al. The OBO Foundry: coordinated evolution of ontologies to support biomedical data integration. Nat Biotechnol 2007;25(11):1251–1255. doi:10.1038/nbt1346

28. Fielding RT, Taylor RN. Principled design of the modern Web architecture. ACM Trans Internet Technol 2002;2(2):115–150. doi:10.1145/514183.514185

29. International Committee on Taxonomy of Viruses (ICTV). ICTV database GitHub repository. 2026. https://github.com/ICTV-Virus-Knowledgebase/ICTVdatabase/. Accessed 09 June 2026.

30. David R, Rybina A, Burel J, et al. “Be sustainable”: EOSC-Life recommendations for implementation of FAIR principles in life science data handling. EMBO J 2023;42:e115008. doi:10.15252/embj.2023115008

31. EVORA Project. EVORA ICTV ontology GitHub repository. 2026. https://github.com/EVORA-project/ictv-ontology. Accessed 09 June 2026.

32. David R, Baumann K, LeFranc Y, et al. Converging on a semantic interoperability framework for the European Data Space for Science, Research and Innovation (EOSC). In: Proceedings of the 2nd Workshop on Ontologies for FAIR and FAIR Ontologies (Onto4FAIR). 2023. doi:10.5281/zenodo.8102786

33. Matentzoglu N, Balhoff J, Bello S, et al. A simple standard for sharing ontological mappings (SSSOM). Database 2022;2022:baac035. doi:10.1093/database/baac035

34. EVORA Project. EVORA virus-taxonomy-mappings GitHub repository. 2026. https://github.com/EVORA-project/virus-taxonomy-mappings. Accessed 09 June 2026.

35. EVORA Project. EVORA ICTV ontology helper and API documentation. 2026. https://evora-project.github.io/ictv-ontology/helpers/. Accessed 09 June 2026.

36. EVORA Project. EVORA ICTV Taxon Resolver live interface. 2026. https://evora-project.github.io/ictv-resolver. Accessed 09 June 2026.

37. EVORA Project. EVORA ICTV Taxon Resolver GitHub repository. 2026. https://github.com/EVORA-project/ictv-resolver. Accessed 09 June 2026.

38. EVORA Project. EVORA Portal catalogue. 2026. https://portal.evora-project.eu/. Accessed 09 June 2026.

39. Pandemic PACT. Programme website. 2026. https://www.pandemicpact.org/. Accessed 09 June 2026.

40. Pandemic PACT. Dashboard visualisations. 2026. https://www.pandemicpact.org/grants/visualise. Accessed 09 June 2026.

41. European Virus Archive (EVA). EVA website. 2026. https://www.european-virus-archive.com/. Accessed 09 June 2026.

42. Romette JL, Prat CM, Gould EA, et al. The European Virus Archive goes global: a growing resource for research. Antiviral Res 2018;158:127–134. doi:10.1016/j.antiviral.2018.07.017

43. Ritsch M, Cassman NA, Saghaei S, Marz M. Navigating the Landscape: A Comprehensive Review of Current Virus Databases. *Viruses* 2023;15(9):1834. doi:10.3390/v15091834

44. Wilkinson MD, Dumontier M, Aalbersberg IJ, et al. The FAIR guiding principles for scientific data management and stewardship. Sci Data 2016;3:160018. doi:10.1038/sdata.2016.18

45. Sansone SA, McQuilton P, Rocca-Serra P, et al. FAIRsharing as a community approach to standards, repositories and policies. Nat Biotechnol 2019;37(4):358–367. doi:10.1038/s41587-019-0080-8

46. David R, Mabile L, Specht A, et al. FAIRness literacy: the Achilles' heel of applying FAIR principles. Data Sci J 2020;19(1):32. doi:10.5334/dsj-2020-032

47. Research Data Alliance COVID-19 Working Group. Recommendations and guidelines on data sharing. 2020. doi:10.15497/rda00052

48. Simmonds P, Adriaenssens EM, Zerbini FM, et al. Four principles to establish a universal virus taxonomy. PLoS Biol 2023;21(2):e3001922. doi:10.1371/journal.pbio.3001922

49. World Health Organization (WHO). Pathogens prioritization: a scientific framework for epidemic and pandemic research preparedness. Meeting report. 2024. https://www.who.int/publications/m/item/pathogens-prioritization-a-scientific-framework-for-epidemic-and-pandemic-research-preparedness. Accessed 09 June 2026.

50. Specker F, Paz A, Crowther TW, Maynard DS. Treemendous: an R package for integrating taxonomic information across backbones. PeerJ 2024;12:e16896. doi:10.7717/peerj.16896

51. Schoch CL, Ciufo S, Domrachev M, et al. NCBI taxonomy: a comprehensive update on curation, resources and tools. Database 2020;2020:baaa062. doi:10.1093/database/baaa062

52. Berners-Lee T. Linked Data. 2006. https://www.w3.org/DesignIssues/LinkedData.html. Accessed 09 June 2026.

53. Hogan A, Blomqvist E, Cochez M, et al. Knowledge graphs. ACM Comput Surv 2021;54(4):1–37. doi:10.1145/3447772

54. Kamdar MR, Musen MA. An empirical meta-analysis of the life sciences linked open data on the web. Sci Data 2021;8:24. doi:10.1038/s41597-021-00797-y

55. OpenAI (2026). ChatGPT (GPT- 4o and GPT- 5-series models, including GPT- 5.4, GPT- 5.5 and GPT- 5.6) [large language models]. Responses to queries made by Philippe Lieutaud, Romain David and R. Curtis Hendrickson during manuscript preparation and revision, June 2024–August 2026. https://chatgpt.com/. Accessed 24 August 2026.

56. GitHub (2025). GitHub Copilot coding agent (underlying model not recorded) [AI-assisted coding agent]. Responses to tasks initiated by James McLaughlin, 7 October 2025. https://github.com/EVORA-project/ictv-ontology/pull/45. Accessed 09 June 2026; https://github.com/EVORA-project/virus-taxonomy-mappings/pull/2. Accessed 09 June 2026.

57. National Information Standards Organization (NISO). Contributor Roles Taxonomy (CRediT). 2026. https://credit.niso.org/. Accessed 09 June 2026.

58. McLaughlin J, Lieutaud P, Hendrickson C. EVORA-project/ictv-ontology: 2026-Jul-28-74d7801 [Software]. Zenodo 2026. doi:10.5281/zenodo.21506852

59. McLaughlin J, Lieutaud P, Hendrickson C. EVORA-project/ictv-ontology [Software]. Zenodo 2026. doi:10.5281/zenodo.20610328

60. McLaughlin J, Lieutaud P. EVORA-project/virus-taxonomy-mappings: Virus Taxonomy Mappings Update. Version release-3 [Software]. Zenodo 2026. doi:10.5281/zenodo.21890424

61. McLaughlin J, Lieutaud P. EVORA-project/virus-taxonomy-mappings [Software]. Zenodo 2026. doi:10.5281/zenodo.20610592

62. EMBL-EBI. Unified ICTV ontology in OLS. 2026. https://www.ebi.ac.uk/ols4/ontologies/ictv. Accessed 09 June 2026.

63. National Center for Biotechnology Information (NCBI). NCBI Taxonomy archive (taxdump archive). 2026. https://ftp.ncbi.nih.gov/pub/taxonomy/taxdump_archive/. Accessed 09 June 2026.

64. EMBL-EBI. OLS4 server documentation. 2026. https://github.com/EBISPOT/ols4/blob/dev/README.md. Accessed 09 June 2026.

Additional files

Additional file 1

File name: Additional file 1_Supplementary material.pdf

File format: .pdf

Title: Supplementary materials—API use, helper libraries, provider landscape and mapping specification

Description: Supplementary Materials S1–S4 provide API usage examples, the helper-library architecture, a comparison of virus taxonomy providers and the ICTV-NCBI mapping specification.

Graphical abstract

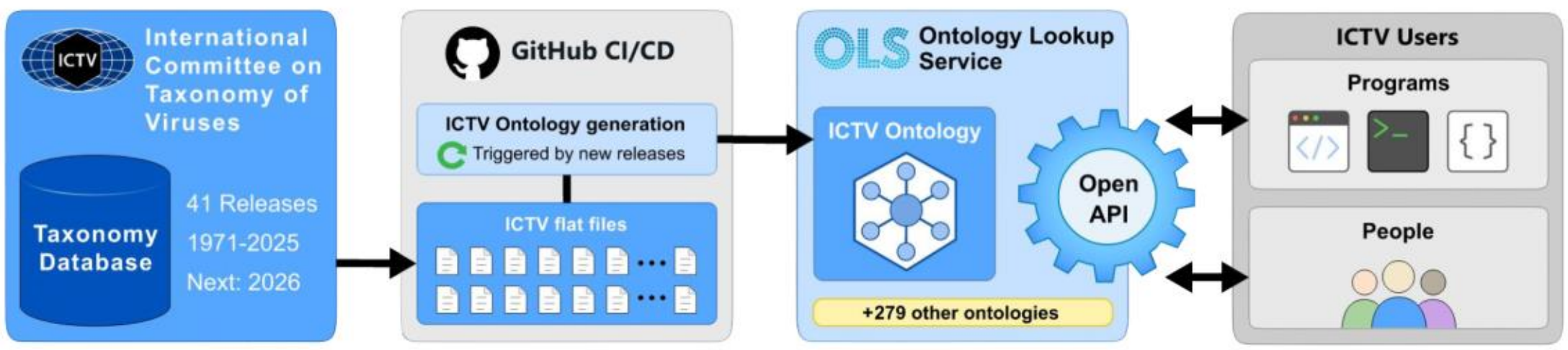

Caption: Unified ICTV ontology generation and public API access. Diagram showing 41 International Committee on Taxonomy of Viruses (ICTV) taxonomy releases transformed through GitHub continuous integration and continuous delivery (CI/CD) into a unified ICTV ontology hosted by the Ontology Lookup Service (OLS). An open application programming interface (API) provides programmatic access for software and browsing access for users.

Alt text: Diagram showing 41 International Committee on Taxonomy of Viruses (ICTV) taxonomy releases transformed through GitHub continuous integration and continuous delivery (CI/CD) into a unified ICTV ontology hosted by the Ontology Lookup Service

(OLS). An open application programming interface (API) provides programmatic access for software and browsing access for users.

Figures

Figure 1

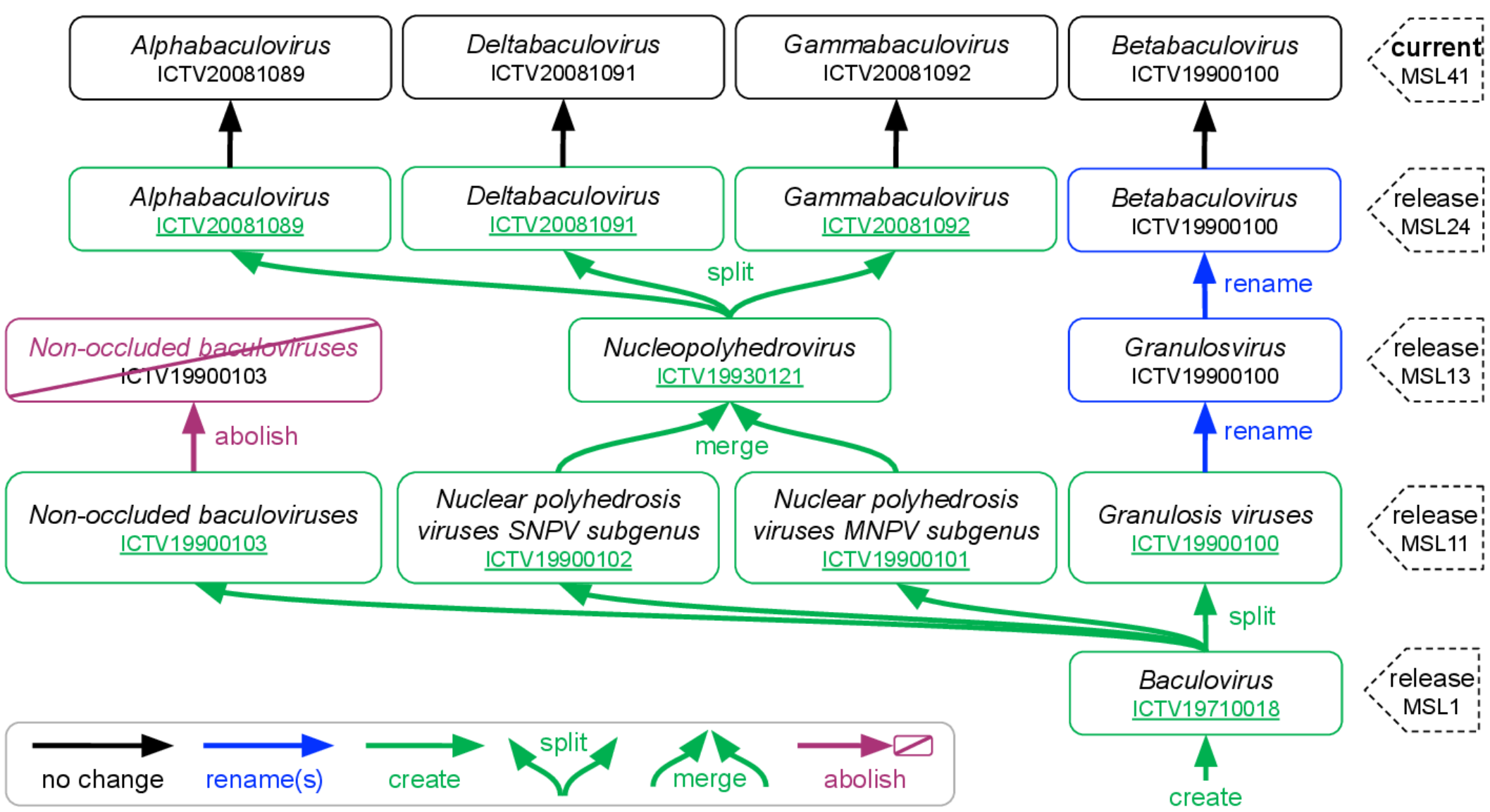

Figure 1. Illustrative taxonomic changes across ICTV releases for a genus-level taxon first recorded in MSL1. A diagram of historical and current genus names originating with the genus *Baculovirus* (ICTV19710018), first recorded in the first ICTV release, and ending with the four currently recognised genera. This example illustrates how the grouping of species into genera, although species are not shown here, can be redefined over time as viruses are better understood and characterised. In this historical trajectory, the original genus was first split into four genera; two of these were then merged; and

the resulting lineage was later split into three genera. This illustrates some of the complexity that can occur in the history of a single taxon, and more broadly how taxonomic changes across different ranks and releases can make the overall taxonomy difficult to trace. It also shows how ICTV IDs are assigned to link historical versions of the same taxon. When a taxon is created, split or merged (green single, diverging or converging arrows, respectively), a new ICTV ID is assigned (underlined and in green). When taxa are renamed, promoted, demoted or moved, the ICTV ID remains the same, linking the different versions.

Alt text: Diagram showing historical and current ICTV genus names and identifiers originating with *Baculovirus* (ICTV19710018) and ending with four currently recognised genera. Green arrows indicate taxon creation, splits and merges across releases.

Figure 2

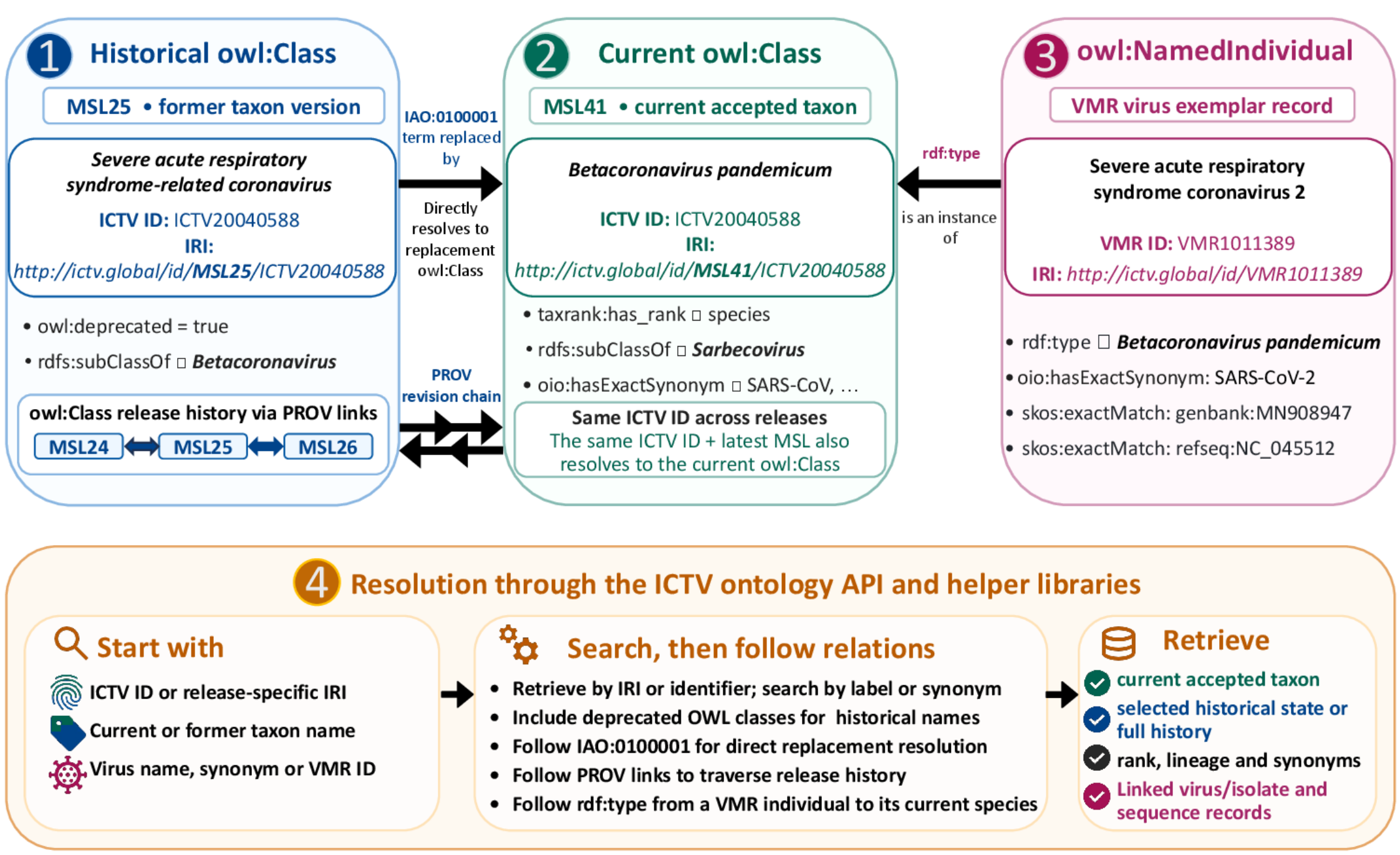

Figure 2. Historical and current taxon resolution using ICTV taxon and VMR virus record information. A former MSL25 taxon version is represented as a release-specific OWL class (i.e. owl:Class) (1), marked as deprecated with “owl:deprecated = true”, and linked through IAO:0100001 to its current MSL41 representation, while PROV relations connect successive historical release states. The current accepted taxon is represented by a distinct OWL class carrying the same stable ICTV identifier (2). In simple taxon rename paths, this identifier can also be combined with the latest MSL release to retrieve the corresponding current OWL class. A VMR virus exemplar record is represented as an owl:NamedIndividual and asserted as an instance of its corresponding species OWL class using rdf:type (3). The resolution workflow (4) shows how ICTV identifiers and release-specific IRIs, current or former taxon names, and virus names, synonyms or VMR identifiers can be used through the ICTV ontology API and helper libraries to retrieve current taxa, historical states, lineage, synonyms and linked sequence records. In the context of this ontology, an OWL class represents an ICTV taxon and should not be confused with the ICTV rank class; rdfs:subClassOf expresses hierarchical relations between these ontology entities. The statement owl:deprecated = true applies to all historical, release-specific OWL classes representing taxa. Deprecated OWL classes linked by either an IAO:0100001 (replaced by) relation or prov:hadRevision link have been updated in a subsequent release. Only OWL classes with owl:deprecated = true and neither an IAO:0100001 relation nor a prov:hadRevision link represent truly abolished taxa.

Alt text: Four-part ontology diagram showing a former MSL25 taxon version represented by a deprecated OWL class and linked directly through IAO:0100001 to its

corresponding current MSL41 OWL class. PROV relations connect the OWL classes in preceding and subsequent releases. The MSL25 and MSL41 classes share the stable identifier ICTV20040588 but have release-specific IRIs. VMR1011389 is a virus exemplar record represented as an OWL named individual. It is linked by rdf:type to the corresponding current species OWL class and by skos:exactMatch cross-references to GenBank and RefSeq records. A lower panel summarises the supported resolution inputs and outputs.

Figure 3

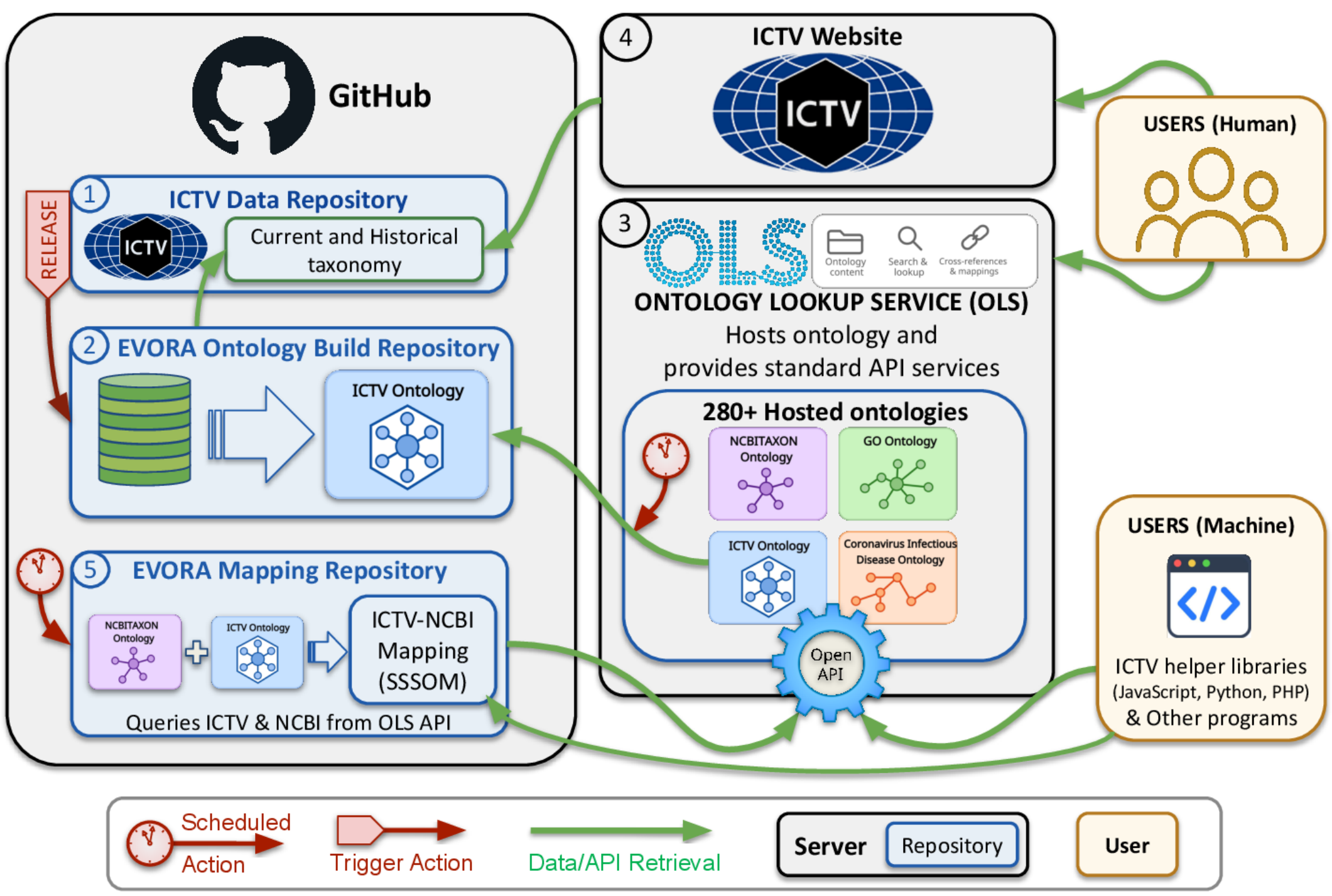

Figure 3. Automated data flows for ICTV ontology generation, publication, ICTV-NCBI mapping and API access. Official ICTV taxonomy data are released through the public ICTV Data repository (1), hosted on GitHub. A new release triggers the EVORA CI/CD workflow hosted in the EVORA Ontology Build repository (2), which retrieves the ICTV

release files, transforms them into OWL ontology artefacts and merges them into a unified ICTV ontology. In this unified ontology, the latest ICTV release represents the current authoritative taxonomy, while entities from previous releases are retained as deprecated historical OWL classes linked through explicit semantic relations. Through scheduled actions, the unified ontology is loaded into the Ontology Lookup Service (OLS) (3), which provides both a browsable human interface and standard API services. The same ICTV source data also feed the ICTV website (4), which provides the official human-facing access point to ICTV taxonomy. In parallel, a separate EVORA Mapping repository (5) runs scheduled workflows that query OLS to use the latest ICTV and NCBITaxon ontologies to generate ICTV–NCBI mappings in SSSOM format; these mappings are stored in the corresponding public repository. Downstream access is supported through direct API queries and language-specific OLS ICTV API helper libraries, enabling external applications to perform historical taxon resolution, cross-resource mapping and to reduce manual curation. Green arrows indicate data/API retrieval actions; dark red arrows indicate workflow trigger actions; dark red clocks indicate scheduled actions. Black boxes represent servers, blue boxes repositories and brown boxes users.

Alt text: Diagram showing the automated data flows for ICTV ontology generation, publication, ICTV–NCBI mapping and API access. Public ICTV taxonomy release data are retrieved by EVORA GitHub workflows, transformed into OWL artefacts, merged into a unified ontology, loaded into OLS for browsing and API access, and used by a separate mapping workflow to generate ICTV-NCBI SSSOM mappings. Symbols

distinguish ICTV, EVORA, OLS and user-facing components, including repositories, servers, scheduled actions, workflow triggers and data/API retrieval flows.

Figure 4

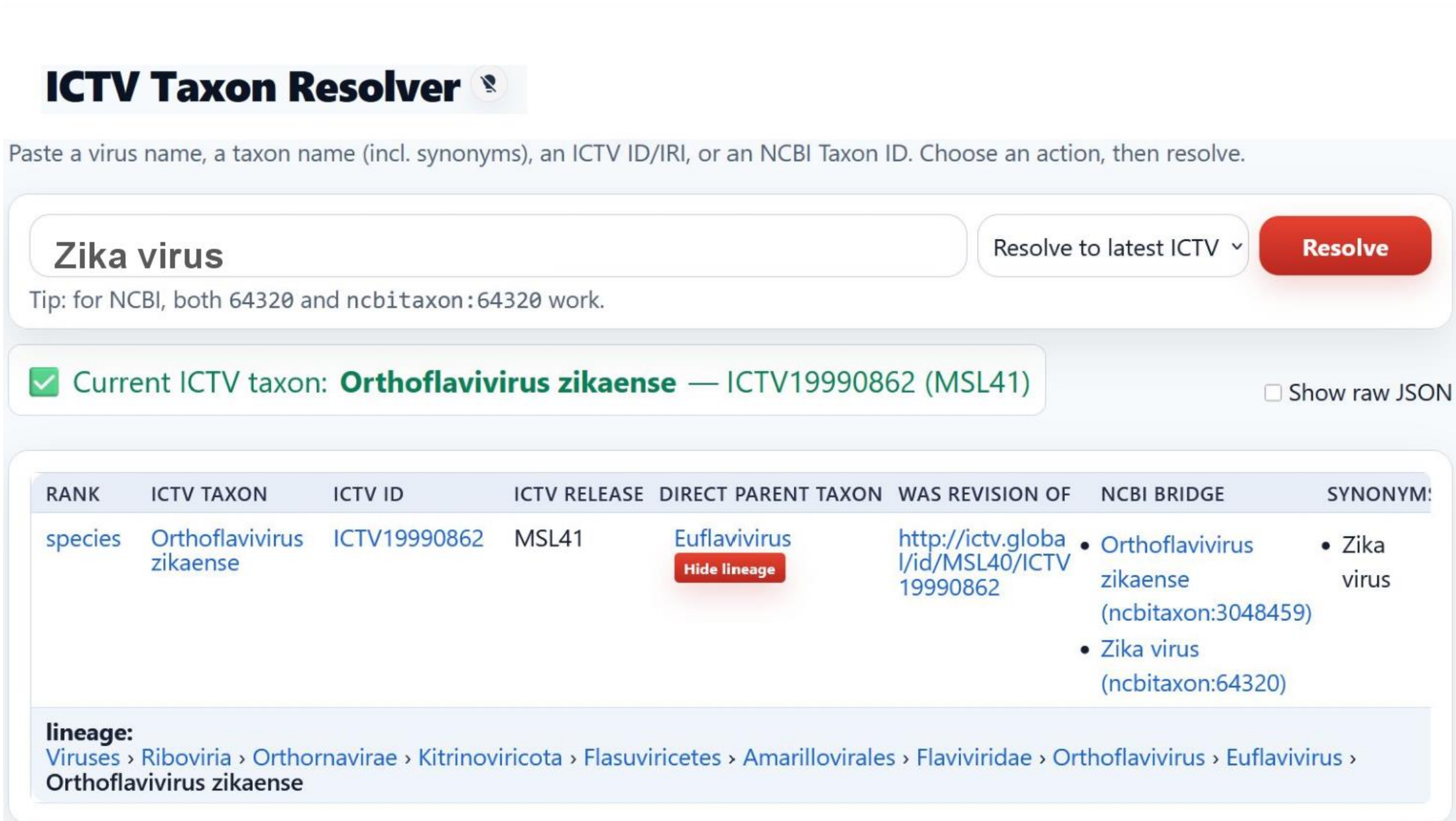

Figure 4. ICTV Taxon Resolver and programmatic taxonomic resolution. The ICTV Taxon Resolver interface illustrates automated resolution of historical or synonymous virus names to the currently accepted ICTV taxon, including lineage reconstruction and cross-references to external identifiers. The resolver is a reference implementation built on the JavaScript helper library that encapsulates Ontology Lookup Service (OLS) API calls. It accepts heterogeneous user inputs, including historical virus names, ICTV identifiers, Internationalized Resource Identifiers (IRIs), synonyms and NCBI Taxon identifiers, and applies a resolution strategy combining label matching, synonym detection, obsolete-term replacement chains and cross-taxonomy mappings. The output

panel displays the currently accepted ICTV taxon together with its hierarchical lineage, rank, revision history and available cross-references to external resources.

Alt text: Screenshot of the ICTV Taxon Resolver web interface showing a historical or synonymous virus taxon query and an output panel with the currently accepted ICTV taxon, lineage, rank, revision history and external cross-references.

Editable review manuscript with page and line numbers
Click here to access/download;Manuscript;GIGA-D-26-00217_manuscript_review_linenumbered.docx

Programmatic access to ICTV virus taxonomy through a public ontology API

Article type: Technical Note

Manuscript ID: GIGA-D-26-00217

Authors: Philippe Lieutaud[1]*, James McLaughlin[2], R. Curtis Hendrickson[3], Romain David[4], Helen Parkinson[2], Elliot J. Lefkowitz[3], Donald M. Dempsey[3], Bruno Coutard[1]

[1] European Viral Outbreak Response Alliance (EVORA); unité des virus émergents - UVE - UMR 190 - Aix Marseille Université, IRD 190, INSERM U1207, France

[2] European Bioinformatics Institute (EMBL-EBI), European Molecular Biology Laboratory, Wellcome Trust Genome Campus, Hinxton, Cambridge CB10 1SD, United Kingdom

[3] International Committee on the Taxonomy of Viruses (ICTV); University of Alabama at Birmingham, Department of Microbiology, Birmingham, Alabama 35294, United States

[4] European Research Infrastructure on Highly Pathogenic Agents (ERINHA AISBL), 98 rue du Trône, B-1050 Bruxelles, Belgium

Corresponding author: Philippe Lieutaud, Philippe.Lieutaud@univ-amu.fr

Author emails: Philippe.Lieutaud@univ-amu.fr; jmcl@ebi.ac.uk; curtish@uab.edu; romain.david@erinha.eu; parkinso@ebi.ac.uk; elliotl@uab.edu; ddempsey@uab.edu; bruno.coutard@univ-amu.fr

Abstract

Background

The International Committee on Taxonomy of Viruses (ICTV) is responsible for developing and maintaining a universal virus taxonomy. As the reference framework for organising the viral world, it is essential for virology and related fields. Despite its widespread use in research and public health, programmatic access to ICTV taxonomy has remained limited, posing challenges for integration, versioning, and interoperability across databases and bioinformatics resources requiring up-to-date virus taxonomy.

Findings

To address this, we developed a public and sustainable solution leveraging ontology-based APIs. All available ICTV Master Species List (MSL) releases, from MSL1 to MSL41, were transformed into a unified, semantically structured ontology comprising more than 195,000 current and historical entities and deployed through the Ontology Lookup Service (OLS). The ontology is automatically rebuilt and republished whenever a new MSL release becomes available. Complementary ICTV-NCBI mappings and helper libraries support integration into downstream systems.

Conclusions

Together, these resources enable, for the first time, public programmatic retrieval of current and historical ICTV taxon names, taxonomic relationships, metadata, and persistent identifiers through stable endpoints, including resolution of former taxonomic terms to their current accepted taxon or taxa and retrieval of taxon histories across

releases. More broadly, this work illustrates a general strategy for transforming structured biological datasets into semantically enriched graph resources exposed through scalable public APIs. These developments enhance interoperability, reduce manual curation, and support FAIR-aligned taxonomic data management in virology and pandemic preparedness.

Keywords

ICTV; Virus taxonomy; Ontology; API; FAIR principles; Interoperability

Key points

- ICTV provides the official taxonomy for classifying viruses and naming virus taxa, but lacks standardised programmatic access.
- Transforming ICTV data into an ontology enables semantic, machine-actionable access across releases via ontology-based APIs.
- ICTV-NCBI mappings support interoperability across bioinformatics resources.
- The integrated ontology-based resource enables programmatic resolution of current and historical viral taxa.
- This approach provides a reusable model for exposing biological datasets through public APIs.

Introduction and landscape

The International Committee on Taxonomy of Viruses (ICTV) [1], established under the Virology Division of the International Union of Microbiological Societies (IUMS) [2], is the authoritative body responsible for the classification of viruses and naming of virus taxa, ensuring a standardised and commonly accepted nomenclature. Virus taxonomy provides a structured framework for classifying viruses based on evolutionary relationships, shared biological properties, and genomic features, such as genetic material, structural features, replication strategies and host range. While everyday virus names function as vernacular labels that can vary across studies and contexts, the ICTV taxon names provide formal, standardised designations that ensure unambiguous communication across the scientific community [3].

The regularly updated ICTV Master Species Lists (MSLs) [4] reflect ongoing advances in virology, including classification of newly discovered viruses and refined taxonomic insights, with one major new release each year, sometimes complemented by minor releases [5]. As virus discovery accelerates, particularly through metagenomics, maintaining consistent and up-to-date taxonomic references in databases and bioinformatics tools relying on ICTV taxa becomes increasingly important.

While the ICTV releases provide authoritative reference data, their distribution format is primarily designed for human consultation and does not readily support programmatic access [6]. As a result, integrating ICTV taxonomy updates into third-party computational systems remains challenging. Broader taxonomic platforms such as NCBI Taxonomy (National Center for Biotechnology Information) [7], GBIF (Global

Biodiversity Information Facility) [8], COL (Catalogue of Life) [9] and Wikidata [10] embed ICTV releases as the authoritative source for virus taxonomy. However, these platforms often incorporate ICTV updates with substantial delays and may lack systematic support for resolving historical taxonomic changes.

Downstream resources can lag by one or more ICTV taxonomic updates, resulting in discrepancies in the classification they expose. Such inconsistencies create significant challenges for users who require the most up-to-date virus classifications, potentially affecting research accuracy and data consistency. For example, outdated species names may persist for some time in major sequence repositories such as GenBank [11] and the European Nucleotide Archive (ENA) [12], leading to further propagation of downstream inconsistencies. Since these repositories rely on the NCBI Taxonomy as their primary taxonomic reference, such delays can be observed by comparing historical taxonomic archives of NCBI and ICTV. A concrete example is ICTV renaming "*Severe acute respiratory syndrome-related coronavirus*" to "*Betacoronavirus pandemicum*" in MSL39. This change did not appear in NCBI Taxonomy for more than a year, and, when it did, was represented by the creation of a new NCBI taxon, rather than a direct renaming of the existing taxa. Obsolete taxon names that continue to be treated as current references complicate reproducibility and hinder interoperability across resources. These problems are inevitable, given that NCBI is not simply embedding ICTV taxonomy, but integrating it within a broader, sequence-linked system whose update process must preserve continuity for existing records and dependent workflows. As such, ICTV remains the authoritative source for virus taxonomy, while NCBI serves as a widely used integration layer. Consequently, taxon abolitions, splits, merges,

moves, and renames may require specific handling rather than simple replacement. Such cases illustrate why programmatic resolution mechanisms are needed [13], along with traceable ICTV identifiers.

The European Viral Outbreak Response Alliance (EVORA) project [14] is a European initiative (2024-2026; grant No. 101131959) [15] that aims to enhance pandemic preparedness and response by strengthening research coordination, regulatory alignment, and data sharing among EU Research Infrastructures. Improving universal adoption of accurate and up-to-date virus taxonomy represents an important component of this mission. Members of EVORA and ICTV collaborated to provide a structured, machine-actionable version of the official ICTV taxonomy using ontology-based APIs, with support for historical name resolution, revision tracking and cross-system identifiers.

Many biological data resources are distributed as relational databases, spreadsheets or tabular datasets that are well suited for storage, but less adapted to programmatic integration across heterogeneous systems. In addition to addressing the immediate need for improved access to ICTV taxonomy, this work illustrates a broader methodological approach for transforming structured database content into semantically enriched knowledge representations through the use of ontologies. Ontologies play a crucial role in the management, integration, and analysis of biological data by providing explicit, human- and machine-readable semantics that support the consistent annotation, sharing and interpretation of complex datasets [16]. Converting ICTV releases into an ontology enables graph-based querying and integration. As graph-based representations naturally capture hierarchical relationships, synonyms, and historical

changes, representing taxonomy as an ontology therefore enables more expressive queries, automated reasoning, and easier integration with other semantic resources.

Across the life sciences, the Ontology Lookup Service (OLS) provides widely used infrastructure for searching, browsing and accessing biological and biomedical ontologies through standard APIs, supporting the reuse of ontology terms and relations in data curation and computational workflows [17, 18].

Methods and strategy

In this paper, "OLS API" refers to the generic programmatic interface provided by the OLS, including its Representational State Transfer (REST) endpoints and controllers. "ICTV ontology API" refers to access to the ICTV ontology through the OLS API, either directly via OLS endpoints or indirectly through ICTV-specific helper libraries built on top of them.

Design requirements and use cases

To support practical integration of ICTV taxonomy into data systems and analytical workflows, several key use cases were defined to address common end-user challenges for automated processing. First, it must provide historical resolution by mapping obsolete names, synonyms, and ICTV identifiers from previous ICTV releases to their corresponding taxa in the current ICTV classification. Second, taxonomic changes must be tracked across releases, including taxa that have been abolished, split, merged, renamed, or reassigned (promoted) within the hierarchy. Third, cross-resource

mapping must be supported, and, specifically, a mapping, with provenance, between NCBI Taxonomy identifiers and ICTV identifiers (historical and current) must be provided. Finally, there must be a stable, public, well-documented API for performing these functions.

Persistent identifiers

Persistent identifiers are key for data integration [19]. The ICTV defines persistent identifiers for all versions of all taxa, as well as links connecting the taxa into hierarchies and documenting changes between releases. These unique identifiers facilitate resolution of outdated taxonomic terms to current ones by allowing taxa to be efficiently tracked across renames, rank changes, merges, splits, and abolitions between releases. To support programmatic resolution and cross-release linkage, and to further support FAIR principles, ICTV publicly documented these existing identifiers and defined Compact Uniform Resource Identifiers (CURIEs) for them. ICTV provides three principal identifiers. In the shorthand used below, the letter prefix indicates the identifier type and # represents the numeric identifier value:

- Taxnode identifier (Taxnode ID; TN#): uniquely identifies a taxon node within the ICTV source database in a specific release.
- ICTV identifier (ICTV ID; ICTV#): identifies the same taxon across releases, including through renames, changes in location within the taxonomic tree (moves), and changes in rank (promotion/demotion). Splits and merges create new ICTV IDs, which are linked back to their antecedent taxa.

- Isolate identifier (Isolate ID; VMR#): defined by the Virus Metadata Resource (VMR) [20], links GenBank sequence accessions for one or more segments to the vernacular name and the corresponding viral species [6].

During ontology generation, TN identifiers are used as ICTV source-table keys to reconstruct parent–child relationships within each release, associate VMR records with taxon nodes and interpret changes between releases. They are not included in the ontology because their role is limited to processing the ICTV source data. Instead, each release-specific taxon state is represented as an OWL class and is assigned a resolvable IRI combining the MSL release and the persistent ICTV ID (for example, http://ictv.global/id/MSL41/ICTV20040588). This design uniquely identifies each release-specific taxon state, preserves direct resolution across simple renames and uses explicit revision and replacement relations when taxonomic events create new ICTV IDs.

This identifier design underpins the modelling of ICTV releases as semantically linked ontology resources. An extremely abridged example of the history of a taxon related to the genus *Baculovirus* in Figure 1 illustrates some of the complexity, and demonstrates how ICTV IDs link historic versions of a taxon, from the first ICTV release to the current MSL41 release [21].

Initial modelling of ICTV releases as ontologies

To meet the design requirements and use cases described above, including historical resolution, cross-release tracking and programmatic querying, each of the 41 (as of 2026) official ICTV taxonomy updates since 1971 was modelled as an independent

OWL2 ontology, representing hierarchies, ranks, synonyms and revision metadata. Taxa were modelled as OWL classes and virus isolates as OWL named individuals. This approach captures taxonomic relationships and their evolution over time, while preserving the structural and semantic fidelity of the original ICTV data in a machine-readable format.

The ontologies were constructed using established semantic standards and interoperable vocabularies commonly used in biomedical ontology ecosystems (e.g. [22], built upon semantic web standards including OWL, RDFS, PROV-O [23] and SKOS semantic relations [24], together with complementary vocabularies such as Dublin Core Terms (DCTERMS), Friend Of A Friend (FOAF), the Taxonomic Rank Vocabulary (TAXRANK) and oboInOwl ontology annotations. This supports compatibility with the broader life-science ontology ecosystem [25] and reuses selected terms and annotations from OBO-associated resources [26]. Where a replacement exists, deprecated historical OWL classes are linked to their replacements using IAO:0100001 (term replaced by), enabling rapid resolution of former terms to their current representations.

The OLS enables direct exposure of ontology data through stable REST API controllers, a web-based user interface [27], and a Model Context Protocol (MCP) server for access by AI agents. OLS provides a ready-to-use infrastructure to host ontologies and deliver programmatic access through standardised endpoints [17]. This approach eliminates the need to develop and maintain custom API infrastructures while ensuring compatibility with the broader ecosystem of life science ontologies. Thus, transforming existing data into a semantically structured graph representation leverages the OLS

infrastructure to efficiently provide standard, public APIs and interoperability across data infrastructures.

The set of release-specific ontologies was published via OLS. The OLS API enables dynamic querying, retrieval of data and linking across ontologies, allowing applications to access up-to-date information directly from ICTV data via the ontology stored in OLS.

Redesign as a unified ontology

However, querying multiple ontologies proved complex for the primary use case of reconciling historical references with the current taxonomy. The system was therefore redesigned to merge all releases into a single, unified ontology (over 195,000 terms in 2026). In this model, the most recent ICTV release represents the authoritative classification, while entities from previous releases are preserved as deprecated historical OWL classes and, where applicable, linked through explicit replacement relations. This design reduces query complexity, while preserving full history, provenance and backward compatibility across ICTV releases. This enables any taxonomic reference appearing in historical datasets or publications to be programmatically reconciled with the current ICTV classification.

The ontology is primarily functional: it was engineered to expose current and historical ICTV taxonomy through OLS and the helper libraries so that taxon identity, lineage, synonyms and revision history can be queried programmatically. It does not import BFO, COB or RO, has not been submitted to the OBO Foundry, and no submission is currently planned.

Figure 2 illustrates how the entities and relations in the unified ontology support taxon resolution across ICTV releases. Release-specific taxon representations, modelled as OWL classes, are connected through PROV revision relations and, when a replacement is defined, through IAO:0100001 to the corresponding OWL class in the latest MSL, which represents the currently accepted ICTV taxon. In simple taxon rename paths, reusing the stable ICTV ID with the latest MSL release also retrieves the current OWL class, whereas IAO:0100001 provides a more direct route to the replacement OWL class and can resolve replacement or merge events without traversing the full release history. VMR virus exemplar records modelled as OWL named individuals are linked by rdf:type to their corresponding species OWL classes in the ontology. Together, these relations allow identifiers and current or former taxon or virus names to be resolved to current taxa, to defined historical states or to complete taxon histories through the OLS API and helper libraries.

Availability and sustainability

Continuous Integration and Deployment (CI/CD) workflows implemented through public GitHub repositories automatically trigger the ontology generation workflow for each release published in the ICTV's official GitHub repository [28]. This automation removes manual intervention and contributes to long-term sustainability of the infrastructure, in line with recommendations for sustainable FAIR implementation in Life Sciences [29]. The unified ontology is immediately available as a release from the EVORA GitHub repository [30], and is automatically deployed to the OLS, typically within 24 hours of an ICTV release, which provides public web browsing and API access. Dissemination and

long-term discoverability are further supported through versioned GitHub and Zenodo releases, as well as registration of the software resources in bio.tools and SciCrunch (see Availability of supporting source code and requirements section).

ICTV-NCBI mapping

To improve interoperability at the broader scale [31], ICTV-NCBI mappings are generated using the OLS API, and stored in SSSOM (Simple Standard for Sharing Ontology Mapping) format, aligning ICTV identifiers with NCBI Taxon identifiers [32]. The code to generate this mapping is maintained in a dedicated GitHub repository [33], where the resulting SSSOM files are then published as releases (see Additional file 1, Supplementary Material S4). While this analysis is also automated using a GitHub CI/CD workflow, it runs on a schedule, decoupled from the release cycles of the two ontologies, allowing independent updates while remaining consistent with the continuously updated ontology infrastructure.

These mappings not only facilitate direct and bidirectional linkage between ICTV and NCBI taxonomies, but also enable alignment across downstream resources that rely on NCBI as a taxonomic backbone, including sequence repositories and bioinformatics platforms. This feature enables broader platforms that rely on the NCBI taxonomy for viral references (e.g., ENA) to stay current with the ICTV taxonomy while continuing to leverage the broader coverage of NCBI taxonomy.

The NCBI integrates the ICTV Taxonomy into its own taxonomy system, which is used to designate species related to the entries in its sequence databases (e.g. GenBank). This integration is supported by an established exchange process between the ICTV

database team and the NCBI team responsible for virus taxonomy updates. This process predates the present work and accommodates NCBI's specific sequence-linked update requirements. The public ICTV ontology API and ICTV-NCBI mappings complement this process by providing a general, community-facing and machine-actionable layer for accessing and reconciling current and historical ICTV taxonomy. Historically, ICTV updates have been incorporated with roughly a one-year delay, resulting in NCBI being one or two releases behind the latest ICTV version (as described above). Observations following MSL41 release suggest a recent effort by NCBI to shorten this delay, with progressive integration starting in May 2026, about two months after the MSL41 release. This included the integration of renamed taxa, while newly created taxa remained absent and abolished taxa had not yet been removed (see Additional file 1, Supplementary Material S3). As part of this work, ICTV has publicly documented several persistent identifiers (described above) that link taxa in successive ICTV Taxonomy versions, which should facilitate rapid integration of new releases. These identifiers are exposed in the ICTV ontology metadata and support the generation of SSSOM ICTV-NCBI mappings. In the released mapping file, lexical ICTV-to-NCBI Taxon correspondences are expressed with skos:exactMatch, enabling alignment between ICTV taxonomy and NCBI Taxonomy identifiers used by downstream sequence resources such as GenBank.

Results

Unified ontology for ICTV taxonomy and OLS deployment

We have produced a unified ontology for the ICTV taxonomy, aggregating all releases into a single resource. The ontology is available for download or via OLS and enables programmatic access to current and historical classifications. Ontology updates are automatically triggered by new ICTV releases and reflected in OLS, typically within 24 hours.

Asynchronously, ICTV-NCBI mappings are generated in SSSOM format, enabling bidirectional resolution between the taxonomies. Mappings are maintained in a dedicated EVORA virus-taxonomy-mappings repository and updated independently [33]. The mapping supports bidirectional many-to-many resolution between ICTV and NCBI taxonomies and facilitates integration with downstream resources. The overall architecture of the generation and publication workflow is shown in **Figure 3**.

To simplify integration into downstream applications, ICTV-specific helper libraries were developed on top of the standard OLS API. These libraries are provided in JavaScript, Python and PHP and encapsulate resolution logic (synonyms, obsolescence, lineage, mappings) while returning normalised data about taxon objects. These objects contain identifiers, labels, synonyms, ranks, lineage, obsolescence metadata and cross-references, enabling application developers to integrate ICTV-aware logic without direct manipulation of raw API calls. The helper libraries prioritise exact identifier and name-based routes before free-text fallback. A bare ICTV ID returns the corresponding OWL

class from the latest matching MSL release by default, while an explicit MSL release retrieves the corresponding historical state. For VMR records represented in the ontology, a VMR identifier resolves through the corresponding OLS individual and its rdf:type relation to the current species OWL class.

The OLS API documentation, ICTV-specific guidance and helper libraries are publicly available [34] (see Additional file 1, Supplementary Material S1). Together, the unified ontology, its OLS deployment, the helper libraries and the complementary SSSOM mappings constitute an integrated ontology-based access resource for automated resolution, cross-taxonomy linkage and reproducible versioning.

Reference application and early adopters

A public demonstration interface, the ICTV Taxon Resolver [35] web application, was developed as a reference implementation using the JavaScript helper library. The interface provides two selectable actions. The default action, ‘Resolve to latest ICTV’, returns the current accepted ICTV taxon when one can be resolved. ‘Show history across MSLs’ starts from the OWL class representing the taxon identified from the query and displays the release-specific OWL classes linked to it across the MSL releases included in the ontology. Consequently, the displayed history may begin after MSL1 or end before MSL41, for example when a taxon was introduced later or subsequently abolished. The source code is available from the EVORA ICTV Resolver GitHub repository [36]. The resolver allows users to submit current and historical ICTV taxon names and virus names, ICTV identifiers, Internationalized Resource Identifiers (IRIs) or NCBI Taxon IDs and receive the corresponding currently accepted ICTV taxon

or taxa, together with their full lineages and revision history. Concise usage guidance is displayed directly beneath the interface title. This tool illustrates practical applicability while serving as a functional community resource. The interface and an example resolution of the former species name *Zika virus* to its post-2022 name and lineage, *Orthoflavivirus zikaense*, are shown in **Figure 4**. For example, in history mode, the relationship between *Alphabaculovirus* and *Nucleopolyhedrovirus* illustrated in Figure 1 can be traced from MSL23 back to MSL13. The resolver is a demonstration and reference implementation built on the JavaScript helper library and does not expose every capability of the OLS-backed ICTV ontology API, for which complete developer documentation is available separately.

Among the first operational deployments using the ICTV ontology API is the EVORA Portal [37], a consolidated catalogue of research infrastructures' resources and services in virology designed to support outbreak response and pandemic preparedness. The utilisation of the ICTV ontology API is integrated through one of the helper libraries directly into the catalogue generation workflow, enabling retrieval of up-to-date taxonomic terms along with associated lineage and synonym information. This ensures consistent use of the latest ICTV taxonomy, even when metadata provided by contributing research infrastructures correspond to earlier ICTV releases. As an added value for users of the research infrastructures, lineage information and historical synonyms retrieved through the ontology API enable enhanced search capabilities. This allows users to find resources using outdated taxa or alternative names, an option that was not available in the former search module.

Early adoption of the ICTV ontology API has also been observed in an external initiative concerned with pandemic preparedness and data integration: the Pandemic PACT programme [38]. This programme is now using ICTV taxonomy via the OLS API to align viral pathogen families and scientific names within its scope with the latest ICTV taxonomy (see Acknowledgements section). The retrieved ICTV metadata will be incorporated into Pandemic PACT dashboard visualisations [39], where they will be integrated with metadata from other ontologies accessed through the OLS, such as the Systematized Nomenclature of Medicine (SNOMED), aligning up-to-date viral pathogen taxonomy with related information, including associated diseases.

Integration of ICTV taxonomy through an API enables timely and automated updates across resources. The European Virus Archive (EVA) [40], a network of distributed virus collections using a single web catalogue for virus and virus-related material ordering and distribution [41], plans to integrate the ICTV ontology into its workflows using OLS API queries to maintain local references to virus taxonomy aligned with the most recent ICTV releases. This will allow EVA datasets to remain synchronised with official taxonomy updates while preserving historical annotations associated with archived materials, which will enhance the interconnectivity of research and data management processes, improving resource discovery and data consistency.

These deployments demonstrate practical applicability across research infrastructures and public health platforms. The system enables automated resolution, cross-taxonomy linkage, reduced manual curation and reproducible versioning.

Discussion

Virus data resources include numerous databases with different scopes, data models, functionalities and degrees of FAIR maturity, as reflected in recent reviews of virus databases [42]. This heterogeneity creates practical challenges for interoperability and synchronisation with authoritative taxonomic references such as ICTV taxonomy. This work demonstrates how relational datasets can be easily transformed and exposed through scalable, public, ontology-based APIs. Standards-based ontology-enabled APIs drastically reduce the work needed to make datasets machine-actionable. Here, we developed an ontology for the widely used and regularly updated ICTV virus taxonomy and exposed it through a public API, providing downstream systems with programmatic access to current and historical taxonomic information and the ability to resolve former terms to the current classification, thereby supporting uses ranging from direct queries to recurring automated updates. The ontology infrastructure acts as a semantic resolution layer that continuously bridges evolving authoritative ICTV releases with downstream computational ecosystems and legacy taxonomic references. Timely updates, in turn, reduce discrepancies that arise when downstream systems rely on outdated or partially synchronised taxonomic references and enable reproducible taxonomic resolution across computational workflows. Research infrastructures, sequence repositories, biodiversity catalogues, and knowledge bases can use automated alignment mechanisms to maintain consistency with official ICTV classifications, reducing the need for extensive manual curation.

Persistent ICTV identifiers and replacement relations provide a structured mechanism to track taxonomic evolution, historically prone to ambiguity and data fragmentation, in a computationally accessible way. Complementary ICTV-NCBI mappings, together with ontology cross-references to associated sequence resources, provide a pragmatic bridge between the authoritative, annually updated ICTV classification and NCBI taxonomy. This improves reproducibility and supports FAIR principles, and their progressive implementation [43, 44, 45]. While this approach substantially improves interoperability and historical resolution, synchronisation across downstream resources still depends on heterogeneous update cycles and integration strategies. In addition, some complex taxonomic transitions, such as splits, merges, or taxon abolitions, may still require complementary expert interpretation in specific downstream analytical contexts.

These capabilities help connect distributed data systems in FAIR-aligned federated environments, where independently maintained resources must remain discoverable, accessible and interoperable, including initiatives such as the European Open Science Cloud (EOSC) and the European Health Data Space (EHDS).

Consistent and machine-actionable taxonomy references support large-scale computational analyses, automated data integration workflows and reproducible comparative studies by reducing ambiguity in taxonomic interpretation. This contributes to improved retrieval and harmonisation of virological data across interoperable research infrastructures and data-sharing environments [46], supporting more reliable data integration, comparative analyses, evidence-based decision making, and more informed experimental design and data interpretation. The infrastructure also

establishes a foundation for future enhancements, including expanded mappings with sequence repositories and the possible development of additional interfaces relying on the public OLS API.

Our approach aligns with the article “Four principles to establish a universal virus taxonomy” [47] that outlines key recommendations to achieve a coherent and comprehensive virus taxonomy. Our work helps make ICTV taxonomy more useful by ensuring that references to the official, evolution-based ICTV taxonomy, can be easily maintained and kept up-to-date, irrespective of the intended use or alternate classification scheme. For example, classifying viruses to support the surveillance and public health response to disease outbreaks (Pathogens prioritization: a scientific framework for epidemic and pandemic research preparedness) [48] results in a classification that is significantly different than that of the ICTV taxonomy. It is however still important to reference the current evolutionary-based classification so that our current knowledge on viruses can be quickly and easily adapted to newly identified or newly emerging ones. The ICTV taxonomy serves as the universal reference for virus classification, while the services we report here enable the critical links needed to connect that taxonomy to alternate schemes.

Adoption of the ICTV ontology API by resources relying on virus taxonomy (EVORA and Pandemic PACT) demonstrates its practical applicability. Ontology-based APIs can facilitate alignment between biological taxonomy resources and clinical terminologies used in public health systems, enabling consistent pathogen identification across research infrastructures, data catalogues, and policy-oriented dashboards. By supporting automated retrieval of up-to-date taxonomic terms, lineage information and

synonym relationships, the API-based approach supports consistent taxonomy usage across distributed data infrastructures and reduces the need for manual synchronisation of taxonomic references similarly to interoperability approaches explored in tools such as Treemendous for reconciling taxonomic backbones in tree species naming [49].

Taxonomic resources and biodiversity platforms such as NCBI Taxonomy and GBIF could use the OLS API to implement more automated synchronisation with ICTV releases. Databases such as ENA and GenBank, which depend on the NCBI taxonomy and its integration of ICTV classifications [50], would in turn benefit from improved data integration and accuracy. In cases where updates of virus taxonomy within NCBI do not immediately reflect the latest ICTV release, these resources could additionally leverage the ICTV ontology API, coupled with the provided SSSOM mappings, to maintain up-to-date virus taxonomy.

Our work builds on established FAIR and semantic standards [25, 51]. The inclusion of detailed metadata and the reuse of established semantic standards are critical for ensuring that the ICTV taxonomy serves as a reliable reference across different platforms and applications. This approach provides a lightweight and sustainable mechanism for publishing structured datasets as interoperable APIs, by leveraging an existing ontology service (OLS) and GitHub’s publicly available workflow automation. This approach is applicable to other domain-specific datasets. In parallel with the public OLS-based service, ICTV is using a dedicated access mechanism for data exchange with NCBI and plans a broader API providing access to a more comprehensive ICTV dataset. These complementary services support specialised or higher-volume exchanges, while the OLS API remains the public, standards-based access layer.

More broadly, this work illustrates how ontology technologies can expose structured datasets as semantically enriched graph representations through interoperable APIs. Traditional database schemas are optimised for storage and transactional operations but often require custom APIs to expose their content programmatically. In contrast, ontology-based representations allow relationships between entities to be explicitly encoded and navigated as a graph, enabling more flexible data integration and discovery. Such representations align with knowledge graph and Linked Open Data (LOD) approaches [52, 53]. More generally, this work demonstrates a reusable pattern for exposing structured, domain-specific datasets as interoperable ontologies and APIs by leveraging established ontology infrastructures, such as the OLS for biomedical ontologies.

Conclusion

This work shows that representing an evolving classification system, such as the ICTV taxonomy, as a unified ontology hosted through a public, scalable, standards-based API, increases its accessibility and addresses key challenges in access, versioning and interoperability. The resulting infrastructure supports backward-compatible resolution of virus taxonomy across historical ICTV releases, enabling historical traceability and alignment with current classifications through semantic modelling, persistent identifiers and interoperable mappings.

By building on open standards, automated workflows and existing ontology infrastructure services, this approach supports long-term sustainability and scalability

while making ICTV taxonomy more readily integrable into computational workflows. It also facilitates synchronisation between authoritative taxonomic references and downstream biological, biomedical and public-health data infrastructures, supporting broader adoption of the latest official ICTV taxonomy as the reference standard and strengthening data consistency, reproducibility and preparedness for emerging viral threats, including through more timely dissemination of high-impact updates such as the pandemic-based designation of the species *Betacoronavirus pandemicum*.

Future developments may support broader interoperability with additional taxonomy providers, sequence repositories and interconnected semantic resources. More broadly, this work provides a reusable model for maintaining, exposing and reusing evolving taxonomic references through sustainable, machine-actionable semantic infrastructure.

Declarations

Ethics approval and consent to participate

Not applicable.

Consent for publication

Not applicable.

Funding

This work was supported by the EVORA project (European Union's HORIZON programme grant agreement N° 101131959). The contribution of the University of Alabama at Birmingham (UAB) team to the research reported in this publication was supported by the National Institute of Allergy and Infectious Diseases of the National Institutes of Health under Award Number U24AI162625. The content is solely the responsibility of the authors and does not necessarily represent the official views of the National Institutes of Health. The UAB team assisted with accessing all viral taxonomic data and associated metadata. The UAB team also served as consultants to assist in the understanding of the underlying ICTV data structures and data organization that were used to construct the ontology. In total, the UAB effort comprised approximately 10% of the overall effort.

Acknowledgements

This work is a product of the EVORA project (European Viral Outbreak Response Alliance) and the ICTV's UAB team.

We gratefully acknowledge Emilia Antonio and Alice Norton from the Pandemic PACT programme for their feedback on the adoption of the ICTV OLS API within Pandemic PACT to align viral pathogen families and scientific names with the latest ICTV taxonomy identifiers.

Authors' contributions

Based on Contributor Roles Taxonomy (CRediT) [54]

- Conceptualization: PL
- Project administration: PL, BC
- Resources: CH, EL, PL, BC, JM, HP
- Software: JM, PL
- Methodology: PL, JM, HP, RD, CH
- Writing – original draft: PL, RD
- Writing – review & editing: PL, RD, HP, JM, BC, CH, EL
- Visualization: PL, RD, CH, DMD

Disclosure of use of AI-assisted tools including generative AI

- During manuscript preparation and revision in 2024-2026, Philippe Lieutaud, Romain David and R. Curtis Hendrickson used OpenAI ChatGPT, initially with GPT- 4o and subsequently with GPT- 5-series models, including GPT- 5.4, GPT- 5.5 and GPT- 5.6, at different stages as a writing and editorial aid [55]. The tool was used to suggest rewording and stylistic improvements, identify redundancy, improve the organisation of arguments, check conformity with journal requirements during final review, including reference formatting and the availability of cited links, and suggest improvements to figure layout and

presentation. The AI-assisted textual and figure-design suggestions were treated as conceptual proposals and were not incorporated verbatim; no AI-generated images were used. The authors determined and validated all final scientific content, and produced the final figures.

- Philippe Lieutaud also used OpenAI ChatGPT with GPT- 5.4 and GPT- 5.5 to assist in drafting user and developer documentation published in the ICTV ontology and ICTV Resolver GitHub repositories. He used the same models to assist in porting the ICTV API helper library from Python to JavaScript and PHP, and to check consistency across the three implementations. GPT- 5.5 was also used to suggest fixes for Zenodo release synchronisation involving .zenodo.json metadata and new GitHub release events in the ICTV ontology and ICTV-NCBI mapping repositories, and to help design a sharded, failure-resilient strategy for long-running ICTV-NCBI mapping updates. GPT- 5.5 also assisted in developing a local script to convert the Word manuscript to LaTeX; Philippe Lieutaud produced and checked the final LaTeX files. The resulting documentation, code and workflow changes were reviewed and validated by the authors against the intended functionality of the libraries, release processes, mapping workflows and OLS API.

- Separately, on 7 October 2025, James McLaughlin used the GitHub Copilot coding agent (underlying model not recorded) to generate initial GitHub Actions implementations for building and releasing the ICTV ontology and for scheduling updates of the ICTV-NCBI mappings [56]. In the mapping repository, Copilot also drafted associated documentation and an initial modification supporting the

incremental reuse of existing mappings. These contributions are preserved in the public development history of the corresponding repositories. The generated code was reviewed and merged by James McLaughlin and subsequently revised and extended by the authors.

- The AI-assisted tools were not used to generate scientific data or independently interpret the results. All generated suggestions and code contributions were critically reviewed and validated by the authors, who take full responsibility for the content of the manuscript and the associated software.

Competing interests

The authors declare that they have no competing interests.

Data availability

All ontology artefacts, helper libraries, mapping resources, example notebooks, and continuous-integration workflows are publicly available under open licences. The ICTV MSL41 source data used in this work are available as the ICTVdatabase MSL41.v1 release [21]. The exact EVORA ICTV ontology release used for this manuscript is archived in Zenodo as EVORA-project/ictv-ontology: 2026-Jul-28-74d7801 doi:10.5281/zenodo.21506852 [57]. The concept DOI 10.5281/zenodo.20610328 resolves to all versions. The exact ICTV-NCBI mapping release is archived in Zenodo as EVORA-project/virus-taxonomy-mappings: Virus Taxonomy Mappings Update, release-3 doi:10.5281/zenodo.21890424 [58]. The complete version history is

accessible through the Zenodo concept DOI doi:10.5281/zenodo.20610592. Current development versions and latest releases are available from the public repositories listed below:

- ICTV database: https://github.com/ICTV-Virus-Knowledgebase/ICTVdatabase/
- ICTV ontology generation code, helper libraries, and generated ontologies (OWL/Turtle files): https://github.com/EVORA-project/ictv-ontology
- Latest ICTV ontology release: https://github.com/EVORA-project/ictv-ontology/releases/latest
- ICTV-NCBI SSSOM mappings: https://github.com/EVORA-project/virus-taxonomy-mappings
- Latest ICTV-NCBI mapping release: https://github.com/EVORA-project/virus-taxonomy-mappings/releases/latest
- Unified ICTV ontology in OLS: https://www.ebi.ac.uk/ols4/ontologies/ictv [59]

All resources are reusable, openly licensed and versioned, enabling independent verification and downstream integration.

NCBI Taxonomy taxdump archives used to verify the integration status of ICTV releases are available from the NCBI taxonomy archive: https://ftp.ncbi.nih.gov/pub/taxonomy/taxdump_archive/ [60]

Availability of supporting source code and requirements

- Project name: ICTV Ontology API and helper libraries
    - Project home page: https://github.com/EVORA-project/ictv-ontology
    - Documentation: https://evora-project.github.io/ictv-ontology/helpers/
    - Operating system(s): Platform independent
    - Programming language: language-agnostic for direct API access; helper libraries are provided in Python 3.8 or later, JavaScript as an ES module, and PHP 7.4 or later.
    - Other requirements: Internet access to the OLS API; depending on the helper library used, the Python requests library, the JavaScript Fetch API or the PHP cURL extension is required. For PHP, allow_url_fopen must also be enabled when accessing the remote ICTV-NCBI SSSOM mapping.
    - Licences:
        - ontology-generation code, Apache License 2.0;
        - ICTV-derived ontology data, Creative Commons Attribution 4.0 International (CC BY 4.0), with attribution to ICTV;
        - JavaScript, Python and PHP helper libraries, MIT License.

    - Version used for this manuscript: GitHub release 2026-Jul-28-74d7801; Zenodo version DOI 10.5281/zenodo.21506852
    - All versions: 10.5281/zenodo.20610328
    - RRID: SCR_028724
    - bio.tools ID: ictv_ontology_api
- Project name: ICTV-to-NCBITaxon SSSOM mapping
    - Project home page: https://github.com/EVORA-project/virus-taxonomy-mappings
    - Operating system(s): Platform independent
    - Programming language: language-agnostic for SSSOM file utilisation; SSSOM file generation code in Python.
    - Release archived at https://doi.org/10.5281/zenodo.21890424 [58]
    - License: CC0 1.0 Universal
    - RRID: SCR_028725
    - bio.tools ID: ictv_to_ncbitaxon_sssom_mapping
- Project name: ICTV Taxon Resolver
    - Project home page: https://github.com/EVORA-project/ictv-resolver
    - Live instance: https://evora-project.github.io/ictv-resolver

  - Programming languages: HTML, JavaScript and CSS.
  - Other requirements: A modern web browser with JavaScript and internet access to OLS.
  - License: MIT.
  - Version used: v1.0.5.
  - RRID: SCR_028722
  - bio.tools ID: ictv_taxon_resolver
- External service dependency: Ontology Lookup Service (OLS):
  - Project home page: https://www.ebi.ac.uk/ols4/
  - Documentation: https://github.com/EBISPOT/ols4/blob/dev/README.md [61]
  - Source code: https://github.com/EBISPOT/ols4
  - Software licence: Apache License 2.0

List of abbreviations

- **API(s):** Application Programming Interface(s)
- **CI/CD:** Continuous Integration and Deployment
- **COL:** Catalogue of Life

- **CURIE:** Compact Uniform Resource Identifier
- **DCTERMS:** Dublin Core Terms namespace
- **ENA:** European Nucleotide Archive
- **ETL process:** Extract, Transform, and Load
- **EVA:** European Virus Archive
- **EVORA:** European Viral Outbreak Response Alliance.
- **FAIR:** Findable, Accessible, Interoperable and Reusable
- **FOAF**: Friend of a Friend ontology
- **GBIF:** Global Biodiversity Information Facility
- **IAO:** Information Artifact Ontology
- **ICTV:** International Committee on Taxonomy of Viruses
- **IRI**: Internationalized Resource Identifier
- **LOD:** Linked Open Data
- **MCP:** Model Context Protocol
- **MSL:** Master Species List
- **NCBI:** National Center for Biotechnology Information
- **OLS:** Ontology Lookup Service

- **OWL:** Web Ontology Language
- **PROV-O**: Provenance Ontology
- **RDFS:** Resource Description Framework Schema
- **REST:** Representational State Transfer
- **SKOS:** Simple Knowledge Organization System
- **SNOMED:** Systematized Nomenclature of Medicine
- **SSSOM:** Simple Standard for Sharing Ontology Mapping
- **TAXRANK:** Taxonomic Rank Vocabulary
- **VMR:** Virus Metadata Resource

References

1. International Committee on Taxonomy of Viruses (ICTV). ICTV website. 2026. https://ictv.global/. Accessed 09 June 2026.
2. International Union of Microbiological Societies (IUMS). IUMS website. 2026. https://www.iums.org/. Accessed 09 June 2026.
3. Zerbini FM, Siddell SG, Mushegian AR, Walker PJ, Lefkowitz EJ, et al. Differentiating between viruses and virus species by writing their names correctly. Arch Virol 2022;167:1231–1234. doi:10.1007/s00705-021-05323-4

4. International Committee on Taxonomy of Viruses (ICTV). ICTV Master Species List (MSL). 2026. https://ictv.global/msl. Accessed 09 June 2026.

5. Simmonds P, Adriaenssens EM, Lefkowitz EJ et al. Changes to virus taxonomy, the international code of virus classification and nomenclature, and the ICTV statutes ratified by the International Committee on Taxonomy of Viruses (2025). *Arch Virol* 2026;171:23. doi:10.1007/s00705-025-06485-1

6. Black EJ, et al. Virus taxonomy: the database of the International Committee on Taxonomy of Viruses. Nucleic Acids Res 2026;54(D1):D776–D789. doi:10.1093/nar/gkaf1159

7. National Center for Biotechnology Information (NCBI). NCBI website. 2026. https://www.ncbi.nlm.nih.gov/. Accessed 09 June 2026.

8. GBIF. Global Biodiversity Information Facility website. 2026. https://www.gbif.org/. Accessed 09 June 2026.

9. Catalogue of Life. Catalogue of Life (COL). 2026. https://www.catalogueoflife.org/. Accessed 09 June 2026.

10. Wikidata. Wikidata. 2026. https://www.wikidata.org/. Accessed 09 June 2026.

11. National Center for Biotechnology Information (NCBI). GenBank. 2026. https://www.ncbi.nlm.nih.gov/genbank/. Accessed 09 June 2026.

12. European Nucleotide Archive (ENA). ENA Browser. 2026. https://www.ebi.ac.uk/ena/browser/home. Accessed 09 June 2026.

13. Boyle B, Hopkins N, Lu Z, et al. The taxonomic name resolution service: an online tool for automated standardization of plant names. BMC Bioinformatics 2013;14:16. doi:10.1186/1471-2105-14-16

14. EVORA. European Viral Outbreak Response Alliance project website. 2026. https://evora-project.eu/. Accessed 09 June 2026.

15. European Commission. European Viral Outbreak Response Alliance (EVORA), grant agreement ID 101131959. CORDIS. 2026. https://doi.org/10.3030/101131959. Accessed 09 June 2026.

16. Malone J, Stevens R, et al. Ten simple rules for selecting a bio-ontology. PLoS Comput Biol 2016;12(2):e1004743. doi:10.1371/journal.pcbi.1004743

17. McLaughlin J, Lagrimas J, Iqbal H, et al. OLS4: a new Ontology Lookup Service for a growing interdisciplinary knowledge ecosystem. Bioinformatics 2025;41(5):btaf279. doi:10.1093/bioinformatics/btaf279

18. EMBL-EBI. Ontology Lookup Service (OLS4). 2026. https://www.ebi.ac.uk/ols4/. Accessed 09 June 2026.

19. McMurry JA, Juty N, Blomberg N, et al. Identifiers for the 21st century: how to design, provision, and reuse persistent identifiers. PLoS Biol 2017;15(6):e2001414. doi:10.1371/journal.pbio.2001414

20. International Committee on Taxonomy of Viruses (ICTV). ICTV Virus Metadata Resource (VMR). 2026. https://ictv.global/vmr. Accessed 09 June 2026.

21. Hendrickson RC, Mims L, Lefkowitz EJ. ICTV-Virus-Knowledgebase/ICTVdatabase: Taxonomy Release: MSL41.v1 20260320 [Dataset]. Zenodo 2026. doi:10.5281/zenodo.19339543

22. Kibbe WA, Arze C, Felix V, et al. Disease Ontology 2015 update: an expanded and updated database of human diseases for linking biomedical knowledge through disease data. Nucleic Acids Res 2015;43(D1):D1071–D1078. doi:10.1093/nar/gku1011

23. Lebo T, Sahoo S, McGuinness D, et al. PROV-O: the PROV ontology. W3C Recommendation 2013. https://www.w3.org/TR/prov-o/. Accessed 12 May 2026.

24. Miles A, Bechhofer S. SKOS simple knowledge organization system reference. W3C Recommendation 2009. https://www.w3.org/TR/skos-reference/. Accessed 12 May 2026.

25. Xu F, Juty N, Goble C, Jupp S, Parkinson H, Courtot M. Features of a FAIR vocabulary. J Biomed Semantics 2023;14(1):6. doi:10.1186/s13326-023-00286-8

26. Smith B, Ashburner M, Rosse C, et al. The OBO Foundry: coordinated evolution of ontologies to support biomedical data integration. Nat Biotechnol 2007;25(11):1251–1255. doi:10.1038/nbt1346

27. Fielding RT, Taylor RN. Principled design of the modern Web architecture. ACM Trans Internet Technol 2002;2(2):115–150. doi:10.1145/514183.514185

28. International Committee on Taxonomy of Viruses (ICTV). ICTV database GitHub repository. 2026. https://github.com/ICTV-Virus-Knowledgebase/ICTVdatabase/. Accessed 09 June 2026.

29. David R, Rybina A, Burel J, et al. "Be sustainable": EOSC-Life recommendations for implementation of FAIR principles in life science data handling. EMBO J 2023;42:e115008. doi:10.15252/embj.2023115008

30. EVORA Project. EVORA ICTV ontology GitHub repository. 2026. https://github.com/EVORA-project/ictv-ontology. Accessed 09 June 2026.

31. David R, Baumann K, LeFranc Y, et al. Converging on a semantic interoperability framework for the European Data Space for Science, Research and Innovation (EOSC). In: Proceedings of the 2nd Workshop on Ontologies for FAIR and FAIR Ontologies (Onto4FAIR). 2023. doi:10.5281/zenodo.8102786

32. Matentzoglu N, Balhoff J, Bello S, et al. A simple standard for sharing ontological mappings (SSSOM). Database 2022;2022:baac035. doi:10.1093/database/baac035

33. EVORA Project. EVORA virus-taxonomy-mappings GitHub repository. 2026. https://github.com/EVORA-project/virus-taxonomy-mappings. Accessed 09 June 2026.

34. EVORA Project. EVORA ICTV ontology helper and API documentation. 2026. https://evora-project.github.io/ictv-ontology/helpers/. Accessed 09 June 2026.

35. EVORA Project. EVORA ICTV Taxon Resolver live interface. 2026. https://evora-project.github.io/ictv-resolver. Accessed 09 June 2026.

36. EVORA Project. EVORA ICTV Taxon Resolver GitHub repository. 2026. https://github.com/EVORA-project/ictv-resolver. Accessed 09 June 2026.

37. EVORA Project. EVORA Portal catalogue. 2026. https://portal.evora-project.eu/. Accessed 09 June 2026.

38. Pandemic PACT. Programme website. 2026. https://www.pandemicpact.org/. Accessed 09 June 2026.

39. Pandemic PACT. Dashboard visualisations. 2026. https://www.pandemicpact.org/grants/visualise. Accessed 09 June 2026.

40. European Virus Archive (EVA). EVA website. 2026. https://www.european-virus-archive.com/. Accessed 09 June 2026.

41. Romette JL, Prat CM, Gould EA, et al. The European Virus Archive goes global: a growing resource for research. Antiviral Res 2018;158:127–134. doi:10.1016/j.antiviral.2018.07.017

42. Ritsch M, Cassman NA, Saghaei S, Marz M. Navigating the Landscape: A Comprehensive Review of Current Virus Databases. *Viruses* 2023;15(9):1834. doi:10.3390/v15091834

43. Wilkinson MD, Dumontier M, Aalbersberg IJ, et al. The FAIR guiding principles for scientific data management and stewardship. Sci Data 2016;3:160018. doi:10.1038/sdata.2016.18

44. Sansone SA, McQuilton P, Rocca-Serra P, et al. FAIRsharing as a community approach to standards, repositories and policies. Nat Biotechnol 2019;37(4):358–367. doi:10.1038/s41587-019-0080-8

45. David R, Mabile L, Specht A, et al. FAIRness literacy: the Achilles' heel of applying FAIR principles. Data Sci J 2020;19(1):32. doi:10.5334/dsj-2020-032

46. Research Data Alliance COVID-19 Working Group. Recommendations and guidelines on data sharing. 2020. doi:10.15497/rda00052

47. Simmonds P, Adriaenssens EM, Zerbini FM, et al. Four principles to establish a universal virus taxonomy. PLoS Biol 2023;21(2):e3001922. doi:10.1371/journal.pbio.3001922

48. World Health Organization (WHO). Pathogens prioritization: a scientific framework for epidemic and pandemic research preparedness. Meeting report. 2024. https://www.who.int/publications/m/item/pathogens-prioritization-a-scientific-framework-for-epidemic-and-pandemic-research-preparedness. Accessed 09 June 2026.

49. Specker F, Paz A, Crowther TW, Maynard DS. Treemendous: an R package for integrating taxonomic information across backbones. PeerJ 2024;12:e16896. doi:10.7717/peerj.16896

50. Schoch CL, Ciufo S, Domrachev M, et al. NCBI taxonomy: a comprehensive update on curation, resources and tools. Database 2020;2020:baaa062. doi:10.1093/database/baaa062

51. Berners-Lee T. Linked Data. 2006. https://www.w3.org/DesignIssues/LinkedData.html. Accessed 09 June 2026.

52. Hogan A, Blomqvist E, Cochez M, et al. Knowledge graphs. ACM Comput Surv 2021;54(4):1–37. doi:10.1145/3447772

53. Kamdar MR, Musen MA. An empirical meta-analysis of the life sciences linked open data on the web. Sci Data 2021;8:24. doi:10.1038/s41597-021-00797-y

54. National Information Standards Organization (NISO). Contributor Roles Taxonomy (CRediT). 2026. https://credit.niso.org/. Accessed 09 June 2026.

55. OpenAI (2026). ChatGPT (GPT-4o and GPT-5-series models, including GPT-5.4, GPT-5.5 and GPT-5.6) [large language models]. Responses to queries made by Philippe Lieutaud, Romain David and R. Curtis Hendrickson during manuscript preparation and revision, June 2024–August 2026. https://chatgpt.com/. Accessed 24 August 2026.

56. GitHub (2025). GitHub Copilot coding agent (underlying model not recorded) [AI-assisted coding agent]. Responses to tasks initiated by James McLaughlin, 7 October 2025. https://github.com/EVORA-project/ictv-ontology/pull/45. Accessed 09 June 2026; https://github.com/EVORA-project/virus-taxonomy-mappings/pull/2. Accessed 09 June 2026.

57. McLaughlin J, Lieutaud P, Hendrickson C. EVORA-project/ictv-ontology: 2026-Jul-28-74d7801 [Software]. Zenodo 2026. doi:10.5281/zenodo.21506852

58. McLaughlin J, Lieutaud P. EVORA-project/virus-taxonomy-mappings: Virus Taxonomy Mappings Update. Version release-3 [Software]. Zenodo 2026. doi:10.5281/zenodo.21890424

59. EMBL-EBI. Unified ICTV ontology in OLS. 2026. https://www.ebi.ac.uk/ols4/ontologies/ictv. Accessed 09 June 2026.

60. National Center for Biotechnology Information (NCBI). NCBI Taxonomy archive (taxdump archive). 2026. https://ftp.ncbi.nih.gov/pub/taxonomy/taxdump_archive/. Accessed 09 June 2026.

61. EMBL-EBI. OLS4 server documentation. 2026. https://github.com/EBISPOT/ols4/blob/dev/README.md. Accessed 09 June 2026.

Additional files

Additional file 1

File name: Additional file 1_Supplementary material.pdf

File format: .pdf

Title: Supplementary materials—API use, helper libraries, provider landscape and mapping specification

Description: Supplementary Materials S1–S4 provide API usage examples, the helper-library architecture, a comparison of virus taxonomy providers and the ICTV-NCBI mapping specification.

Graphical abstract

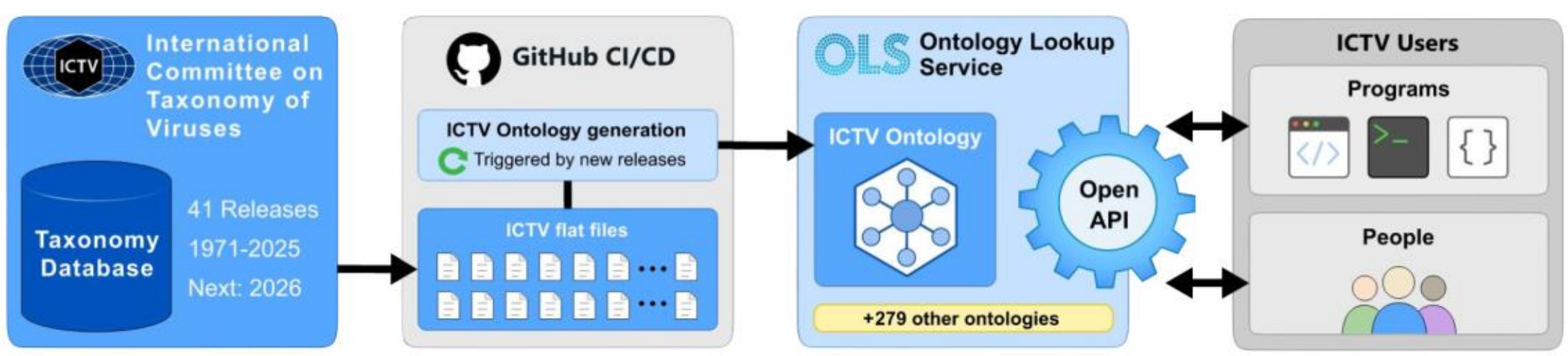

Caption: Unified ICTV ontology generation and public API access. Diagram showing 41 International Committee on Taxonomy of Viruses (ICTV) taxonomy releases transformed through GitHub continuous integration and continuous delivery (CI/CD) into a unified ICTV ontology hosted by the Ontology Lookup Service (OLS). An open application programming interface (API) provides programmatic access for software and browsing access for users.

Alt text: Diagram showing 41 International Committee on Taxonomy of Viruses (ICTV) taxonomy releases transformed through GitHub continuous integration and continuous delivery (CI/CD) into a unified ICTV ontology hosted by the Ontology Lookup Service (OLS). An open application programming interface (API) provides programmatic access for software and browsing access for users.

Figures

Figure 1

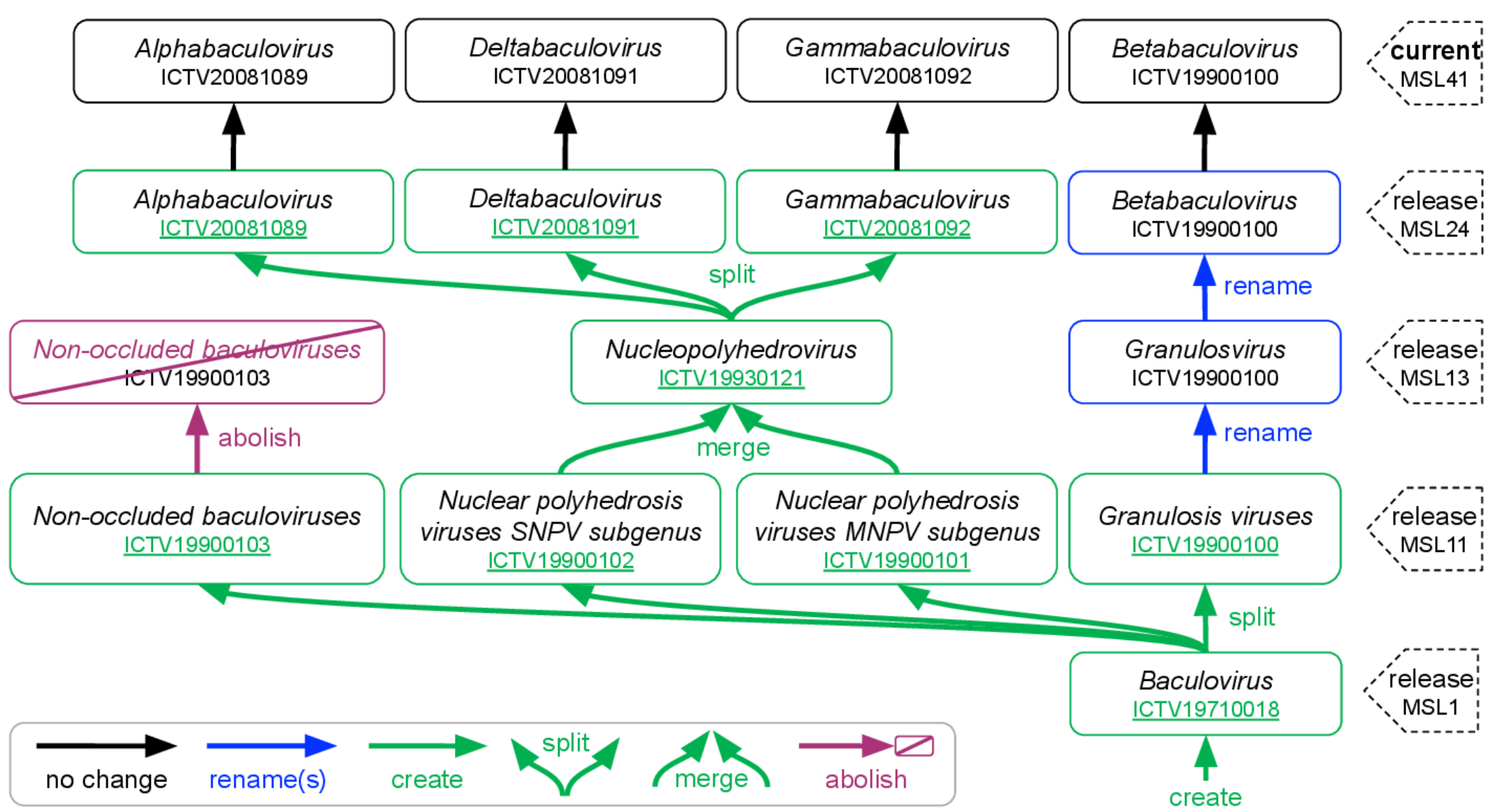

Figure 1. Illustrative taxonomic changes across ICTV releases for a genus-level taxon first recorded in MSL1. A diagram of historical and current genus names originating with the genus *Baculovirus* (ICTV19710018), first recorded in the first ICTV release, and ending with the four currently recognised genera. This example illustrates how the

grouping of species into genera, although species are not shown here, can be redefined over time as viruses are better understood and characterised. In this historical trajectory, the original genus was first split into four genera; two of these were then merged; and the resulting lineage was later split into three genera. This illustrates some of the complexity that can occur in the history of a single taxon, and more broadly how taxonomic changes across different ranks and releases can make the overall taxonomy difficult to trace. It also shows how ICTV IDs are assigned to link historical versions of the same taxon. When a taxon is created, split or merged (green single, diverging or converging arrows, respectively), a new ICTV ID is assigned (underlined and in green). When taxa are renamed, promoted, demoted or moved, the ICTV ID remains the same, linking the different versions.

Alt text: Diagram showing historical and current ICTV genus names and identifiers originating with *Baculovirus* (ICTV19710018) and ending with four currently recognised genera. Green arrows indicate taxon creation, splits and merges across releases.

Figure 2

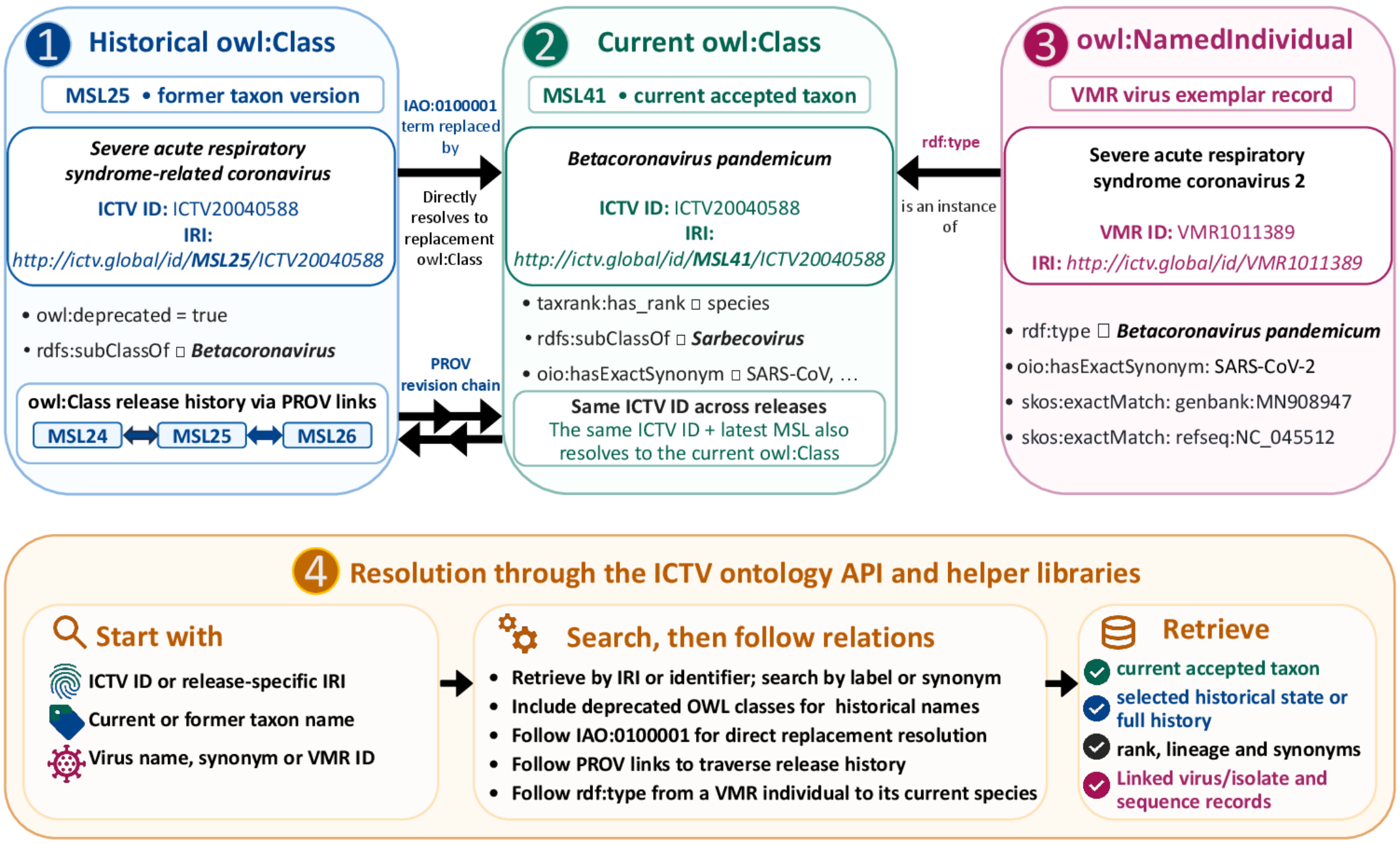

Figure 2. Historical and current taxon resolution using ICTV taxon and VMR virus record information. A former MSL25 taxon version is represented as a release-specific OWL class (i.e. owl:Class) (1), marked as deprecated with "owl:deprecated = true", and linked through IAO:0100001 to its current MSL41 representation, while PROV relations connect successive historical release states. The current accepted taxon is represented by a distinct OWL class carrying the same stable ICTV identifier (2). In simple taxon rename paths, this identifier can also be combined with the latest MSL release to retrieve the corresponding current OWL class. A VMR virus exemplar record is represented as an owl:NamedIndividual and asserted as an instance of its corresponding species OWL class using rdf:type (3). The resolution workflow (4) shows how ICTV identifiers and release-specific IRIs, current or former taxon names, and virus

names, synonyms or VMR identifiers can be used through the ICTV ontology API and helper libraries to retrieve current taxa, historical states, lineage, synonyms and linked sequence records. In the context of this ontology, an OWL class represents an ICTV taxon and should not be confused with the ICTV rank class; rdfs:subClassOf expresses hierarchical relations between these ontology entities. The statement owl:deprecated = true applies to all historical, release-specific OWL classes representing taxa. Deprecated OWL classes linked by either an IAO:0100001 (replaced by) relation or prov:hadRevision link have been updated in a subsequent release. Only OWL classes with owl:deprecated = true and neither an IAO:0100001 relation nor a prov:hadRevision link represent truly abolished taxa.

Alt text: Four-part ontology diagram showing a former MSL25 taxon version represented by a deprecated OWL class and linked directly through IAO:0100001 to its corresponding current MSL41 OWL class. PROV relations connect the OWL classes in preceding and subsequent releases. The MSL25 and MSL41 classes share the stable identifier ICTV20040588 but have release-specific IRIs. VMR1011389 is a virus exemplar record represented as an OWL named individual. It is linked by rdf:type to the corresponding current species OWL class and by skos:exactMatch cross-references to GenBank and RefSeq records. A lower panel summarises the supported resolution inputs and outputs.

Figure 3

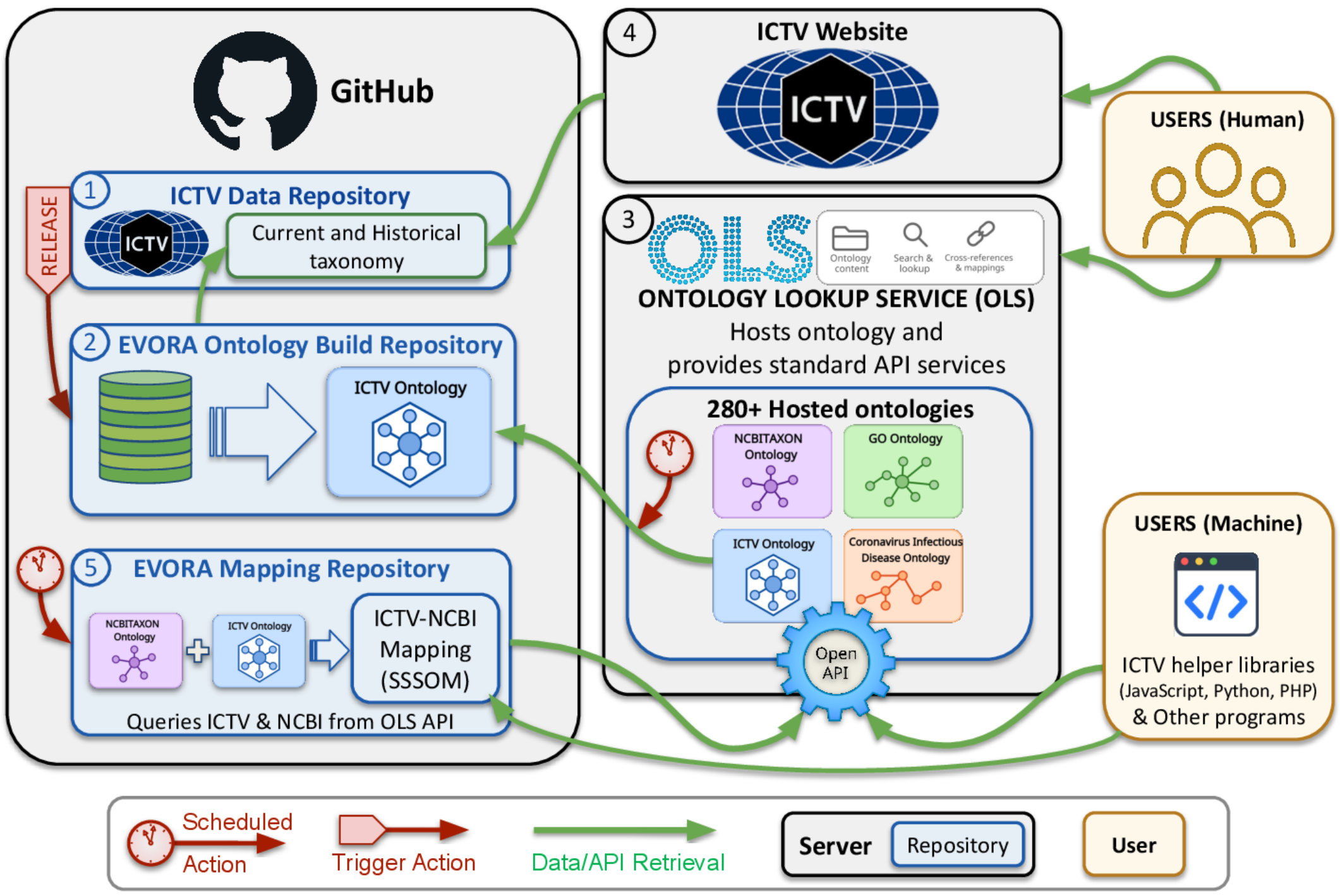

Figure 3. Automated data flows for ICTV ontology generation, publication, ICTV-NCBI mapping and API access. Official ICTV taxonomy data are released through the public ICTV Data repository (1), hosted on GitHub. A new release triggers the EVORA CI/CD workflow hosted in the EVORA Ontology Build repository (2), which retrieves the ICTV release files, transforms them into OWL ontology artefacts and merges them into a unified ICTV ontology. In this unified ontology, the latest ICTV release represents the current authoritative taxonomy, while entities from previous releases are retained as deprecated historical OWL classes linked through explicit semantic relations. Through scheduled actions, the unified ontology is loaded into the Ontology Lookup Service (OLS) (3), which provides both a browsable human interface and standard API services. The same ICTV source data also feed the ICTV website (4), which provides the official

human-facing access point to ICTV taxonomy. In parallel, a separate EVORA Mapping repository (5) runs scheduled workflows that query OLS to use the latest ICTV and NCBITaxon ontologies to generate ICTV–NCBI mappings in SSSOM format; these mappings are stored in the corresponding public repository. Downstream access is supported through direct API queries and language-specific OLS ICTV API helper libraries, enabling external applications to perform historical taxon resolution, cross-resource mapping and to reduce manual curation. Green arrows indicate data/API retrieval actions; dark red arrows indicate workflow trigger actions; dark red clocks indicate scheduled actions. Black boxes represent servers, blue boxes repositories and brown boxes users.

Alt text: Diagram showing the automated data flows for ICTV ontology generation, publication, ICTV–NCBI mapping and API access. Public ICTV taxonomy release data are retrieved by EVORA GitHub workflows, transformed into OWL artefacts, merged into a unified ontology, loaded into OLS for browsing and API access, and used by a separate mapping workflow to generate ICTV-NCBI SSSOM mappings. Symbols distinguish ICTV, EVORA, OLS and user-facing components, including repositories, servers, scheduled actions, workflow triggers and data/API retrieval flows.

Figure 4

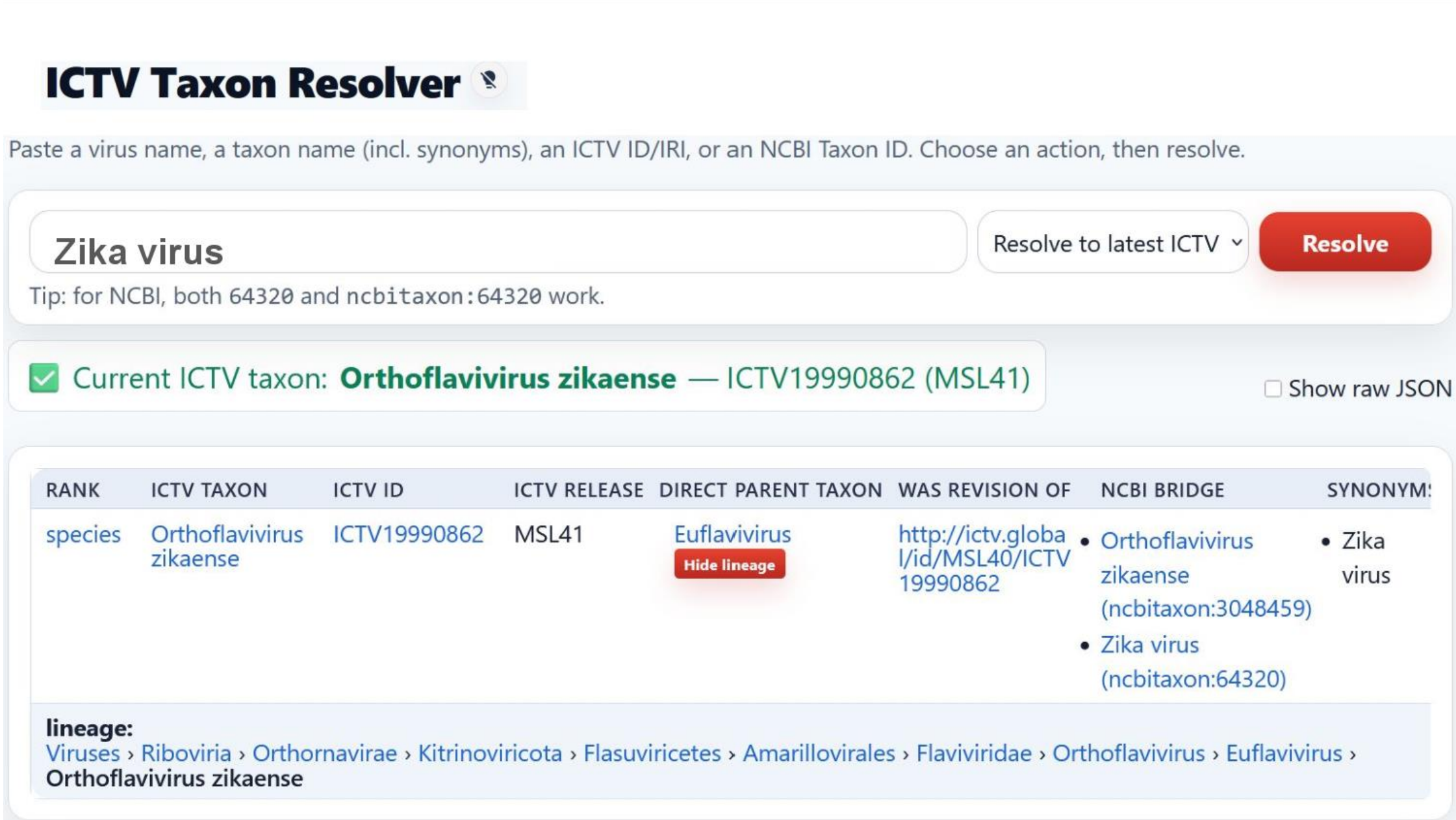

Figure 4. ICTV Taxon Resolver and programmatic taxonomic resolution. The ICTV Taxon Resolver interface illustrates automated resolution of historical or synonymous virus names to the currently accepted ICTV taxon, including lineage reconstruction and cross-references to external identifiers. The resolver is a reference implementation built on the JavaScript helper library that encapsulates Ontology Lookup Service (OLS) API calls. It accepts heterogeneous user inputs, including historical virus names, ICTV identifiers, Internationalized Resource Identifiers (IRIs), synonyms and NCBI Taxon identifiers, and applies a resolution strategy combining label matching, synonym detection, obsolete-term replacement chains and cross-taxonomy mappings. The output panel displays the currently accepted ICTV taxon together with its hierarchical lineage, rank, revision history and available cross-references to external resources.

Alt text: Screenshot of the ICTV Taxon Resolver web interface showing a historical or synonymous virus taxon query and an output panel with the currently accepted ICTV taxon, lineage, rank, revision history and external cross-references.

Clean compiled manuscript without line numbers Click here to access/download;Manuscript;main_review.pdf

GigaScience, 2026, 1–15

doi: xx.xxxx/xxxx
Manuscript in Preparation
Technical Note

TECHNICAL NOTE

Programmatic access to ICTV virus taxonomy through a public ontology API

Philippe Lieutaud[1,*], James McLaughlin[2,*], R. Curtis Hendrickson[3,*], Romain David[4,*], Helen Parkinson[2,*], Elliot J. Lefkowitz[3,*], Donald M. Dempsey[3,*] and Bruno Coutard[1,*]

[1]European Viral Outbreak Response Alliance (EVORA); unité des virus émergents - UVE - UMR 190 - Aix Marseille Université, IRD 190, INSERM U1207, France and [2]European Bioinformatics Institute (EMBL-EBI), European Molecular Biology Laboratory, Wellcome Trust Genome Campus, Hinxton, Cambridge CB10 1SD, United Kingdom and [3]International Committee on the Taxonomy of Viruses (ICTV); University of Alabama at Birmingham, Department of Microbiology, Birmingham, Alabama 35294, United States and [4]European Research Infrastructure on Highly Pathogenic Agents (ERINHA AISBL), 98 rue du Trône, B-1050 Bruxelles, Belgium

*Corresponding author: Philippe.Lieutaud@univ-amu.fr. Author emails: Philippe.Lieutaud@univ-amu.fr; jmcl@ebi.ac.uk; curtish@uab.edu; romain.david@erinha.eu; parkinso@ebi.ac.uk; elliotl@uab.edu; ddempsey@uab.edu; bruno.coutard@univ-amu.fr.

Abstract

Background
The International Committee on Taxonomy of Viruses (ICTV) is responsible for developing and maintaining a universal virus taxonomy. As the reference framework for organising the viral world, it is essential for virology and related fields. Despite its widespread use in research and public health, programmatic access to ICTV taxonomy has remained limited, posing challenges for integration, versioning, and interoperability across databases and bioinformatics resources requiring up-to-date virus taxonomy.

Findings
To address this, we developed a public and sustainable solution leveraging ontology-based APIs. All available ICTV Master Species List (MSL) releases, from MSL1 to MSL41, were transformed into a unified, semantically structured ontology comprising more than 195,000 current and historical entities and deployed through the Ontology Lookup Service (OLS). The ontology is automatically rebuilt and republished whenever a new MSL release becomes available. Complementary ICTV-NCBI mappings and helper libraries support integration into downstream systems.

Conclusions
Together, these resources enable, for the first time, public programmatic retrieval of current and historical ICTV taxon names, taxonomic relationships, metadata, and persistent identifiers through stable endpoints, including resolution of former taxonomic terms to their current accepted taxon or taxa and retrieval of taxon histories across releases. More broadly, this work illustrates a general strategy for transforming structured biological datasets into semantically enriched graph resources exposed through scalable public APIs. These developments enhance interoperability, reduce manual curation, and support FAIR-aligned taxonomic data management in virology and pandemic preparedness.

Key words: ICTV; Virus taxonomy; Ontology; API; FAIR principles; Interoperability

Compiled on: August 25, 2026.
Draft manuscript prepared by the author.

Key Points

- ICTV provides the official taxonomy for classifying viruses and naming virus taxa, but lacks standardised programmatic access.
- Transforming ICTV data into an ontology enables semantic, machine-actionable access across releases via ontology-based APIs.
- ICTV-NCBI mappings support interoperability across bioinformatics resources.
- The integrated ontology-based resource enables programmatic resolution of current and historical viral taxa.
- This approach provides a reusable model for exposing biological datasets through public APIs.

Introduction and landscape

The International Committee on Taxonomy of Viruses (ICTV) [1], established under the Virology Division of the International Union of Microbiological Societies (IUMS) [2], is the authoritative body responsible for the classification of viruses and naming of virus taxa, ensuring a standardised and commonly accepted nomenclature. Virus taxonomy provides a structured framework for classifying viruses based on evolutionary relationships, shared biological properties, and genomic features, such as genetic material, structural features, replication strategies and host range. While everyday virus names function as vernacular labels that can vary across studies and contexts, the ICTV taxon names provide formal, standardised designations that ensure unambiguous communication across the scientific community [3].

The regularly updated ICTV Master Species Lists (MSLs) [4] reflect ongoing advances in virology, including classification of newly discovered viruses and refined taxonomic insights, with one major new release each year, sometimes complemented by minor releases [5]. As virus discovery accelerates, particularly through metagenomics, maintaining consistent and up-to-date taxonomic references in databases and bioinformatics tools relying on ICTV taxa becomes increasingly important.

While the ICTV releases provide authoritative reference data, their distribution format is primarily designed for human consultation and does not readily support programmatic access [6]. As a result, integrating ICTV taxonomy updates into third-party computational systems remains challenging. Broader taxonomic platforms such as NCBI Taxonomy (National Center for Biotechnology Information) [7], GBIF (Global Biodiversity Information Facility) [8], COL (Catalogue of Life) [9] and Wikidata [10] embed ICTV releases as the authoritative source for virus taxonomy. However, these platforms often incorporate ICTV updates with substantial delays and may lack systematic support for resolving historical taxonomic changes.

Downstream resources can lag by one or more ICTV taxonomic updates, resulting in discrepancies in the classification they expose. Such inconsistencies create significant challenges for users who require the most up-to-date virus classifications, potentially affecting research accuracy and data consistency. For example, outdated species names may persist for some time in major sequence repositories such as GenBank [11] and the European Nucleotide Archive (ENA) [12], leading to further propagation of downstream inconsistencies. Since these repositories rely on the NCBI Taxonomy as their primary taxonomic reference, such delays can be observed by comparing historical taxonomic archives of NCBI and ICTV. A concrete example is ICTV renaming "*Severe acute respiratory syndrome-related coronavirus*" to "*Betacoronavirus pandemicum*" in MSL39. This change did not appear in NCBI Taxonomy for more than a year, and, when it did, was represented by the creation of a new NCBI taxon, rather than a direct renaming of the existing taxa. Obsolete taxon names that continue to be treated as current references complicate reproducibility and hinder interoperability across resources. These problems are inevitable, given that NCBI is not simply embedding ICTV taxonomy, but integrating it within a broader, sequence-linked system whose update process must preserve continuity for existing records and dependent workflows. As such, ICTV remains the authoritative source for virus taxonomy, while NCBI serves as a widely used integration layer. Consequently, taxon abolitions, splits, merges, moves, and renames may require specific handling rather than simple replacement. Such cases illustrate why programmatic resolution mechanisms are needed [13], along with traceable ICTV identifiers.

The European Viral Outbreak Response Alliance (EVORA) project [14] is a European initiative (2024-2026; grant No. 101131959) [15] that aims to enhance pandemic preparedness and response by strengthening research coordination, regulatory alignment, and data sharing among EU Research Infrastructures. Improving universal adoption of accurate and up-to-date virus taxonomy represents an important component of this mission. Members of EVORA and ICTV collaborated to provide a structured, machine-actionable version of the official ICTV taxonomy using ontology-based APIs, with support for historical name resolution, revision tracking and cross-system identifiers.

Many biological data resources are distributed as relational databases, spreadsheets or tabular datasets that are well suited for storage, but less adapted to programmatic integration across heterogeneous systems. In addition to addressing the immediate need for improved access to ICTV taxonomy, this work illustrates a broader methodological approach for transforming structured database content into semantically enriched knowledge representations through the use of ontologies. Ontologies play a crucial role in the management, integration, and analysis of biological data by providing explicit, human- and machine-readable semantics that support the consistent annotation, sharing and interpretation of complex datasets [16]. Converting ICTV releases into an ontology enables graph-based querying and integration. As graph-based representations naturally capture hierarchical relationships, synonyms, and historical changes, representing taxonomy as an ontology therefore enables more expressive queries, automated reasoning, and easier integration with other semantic resources.

Across the life sciences, the Ontology Lookup Service (OLS) provides widely used infrastructure for searching, browsing and accessing biological and biomedical ontologies through standard APIs, supporting the reuse of ontology terms and relations in data curation and computational workflows [17, 18].

Methods and strategy

In this paper, "OLS API" refers to the generic programmatic interface provided by the OLS, including its Representational State Transfer (REST) endpoints and controllers. "ICTV ontology API" refers to access to the ICTV ontology through the OLS API, either directly via OLS endpoints or indirectly through ICTV-specific helper libraries built on top of them.

Design requirements and use cases

To support practical integration of ICTV taxonomy into data systems and analytical workflows, several key use cases were defined

to address common end-user challenges for automated processing. First, it must provide historical resolution by mapping obsolete names, synonyms, and ICTV identifiers from previous ICTV releases to their corresponding taxa in the current ICTV classification. Second, taxonomic changes must be tracked across releases, including taxa that have been abolished, split, merged, renamed, or reassigned (promoted) within the hierarchy. Third, cross-resource mapping must be supported, and, specifically, a mapping, with provenance, between NCBI Taxonomy identifiers and ICTV identifiers (historical and current) must be provided. Finally, there must be a stable, public, well-documented API for performing these functions.

Persistent identifiers

Persistent identifiers are key for data integration [19]. The ICTV defines persistent identifiers for all versions of all taxa, as well as links connecting the taxa into hierarchies and documenting changes between releases. These unique identifiers facilitate resolution of outdated taxonomic terms to current ones by allowing taxa to be efficiently tracked across renames, rank changes, merges, splits, and abolitions between releases. To support programmatic resolution and cross-release linkage, and to further support FAIR principles, ICTV publicly documented these existing identifiers and defined Compact Uniform Resource Identifiers (CURIEs) for them. ICTV provides three principal identifiers. In the shorthand used below, the letter prefix indicates the identifier type and # represents the numeric identifier value:

- Taxnode identifier (Taxnode ID; TN#): uniquely identifies a taxon node within the ICTV source database in a specific release.
- ICTV identifier (ICTV ID; ICTV#): identifies the same taxon across releases, including through renames, changes in location within the taxonomic tree (moves), and changes in rank (promotion/demotion). Splits and merges create new ICTV IDs, which are linked back to their antecedent taxa.
- Isolate identifier (Isolate ID; VMR#): defined by the Virus Metadata Resource (VMR) [20], links GenBank sequence accessions for one or more segments to the vernacular name and the corresponding viral species [6].

During ontology generation, TN identifiers are used as ICTV source-table keys to reconstruct parent–child relationships within each release, associate VMR records with taxon nodes and interpret changes between releases. They are not included in the ontology because their role is limited to processing the ICTV source data. Instead, each release-specific taxon state is represented as an OWL class and is assigned a resolvable IRI combining the MSL release and the persistent ICTV ID (for example, `http://ictv.global/id/MSL41/ICTV20040588`). This design uniquely identifies each release-specific taxon state, preserves direct resolution across simple renames and uses explicit revision and replacement relations when taxonomic events create new ICTV IDs.

This identifier design underpins the modelling of ICTV releases as semantically linked ontology resources. An extremely abridged example of the history of a taxon related to the genus *Baculovirus* in Figure 1 illustrates some of the complexity, and demonstrates how ICTV IDs link historic versions of a taxon, from the first ICTV release to the current MSL41 release [21].

Initial modelling of ICTV releases as ontologies

To meet the design requirements and use cases described above, including historical resolution, cross-release tracking and programmatic querying, each of the 41 (as of 2026) official ICTV taxonomy updates since 1971 was modelled as an independent OWL2 ontology, representing hierarchies, ranks, synonyms and revision metadata. Taxa were modelled as OWL classes and virus isolates as OWL named individuals. This approach captures taxonomic relationships and their evolution over time, while preserving the structural and semantic fidelity of the original ICTV data in a machine-readable format.

The ontologies were constructed using established semantic standards and interoperable vocabularies commonly used in biomedical ontology ecosystems (e.g. [22], built upon semantic web standards including OWL, RDFS, PROV-O [23] and SKOS semantic relations [24], together with complementary vocabularies such as Dublin Core Terms (DCTERMS), Friend Of A Friend (FOAF), the Taxonomic Rank Vocabulary (TAXRANK) and oboInOwl ontology annotations. This supports compatibility with the broader life-science ontology ecosystem [25] and reuses selected terms and annotations from OBO-associated resources [26]. Where a replacement exists, deprecated historical OWL classes are linked to their replacements using IAO:0100001 (term replaced by), enabling rapid resolution of former terms to their current representations.

The OLS enables direct exposure of ontology data through stable REST API controllers, a web-based user interface [27], and a Model Context Protocol (MCP) server for access by AI agents. OLS provides a ready-to-use infrastructure to host ontologies and deliver programmatic access through standardised endpoints [17]. This approach eliminates the need to develop and maintain custom API infrastructures while ensuring compatibility with the broader ecosystem of life science ontologies. Thus, transforming existing data into a semantically structured graph representation leverages the OLS infrastructure to efficiently provide standard, public APIs and interoperability across data infrastructures.

The set of release-specific ontologies was published via OLS. The OLS API enables dynamic querying, retrieval of data and linking across ontologies, allowing applications to access up-to-date information directly from ICTV data via the ontology stored in OLS.

Redesign as a unified ontology

However, querying multiple ontologies proved complex for the primary use case of reconciling historical references with the current taxonomy. The system was therefore redesigned to merge all releases into a single, unified ontology (over 195,000 terms in 2026). In this model, the most recent ICTV release represents the authoritative classification, while entities from previous releases are preserved as deprecated historical OWL classes and, where applicable, linked through explicit replacement relations. This design reduces query complexity, while preserving full history, provenance and backward compatibility across ICTV releases. This enables any taxonomic reference appearing in historical datasets or publications to be programmatically reconciled with the current ICTV classification.

The ontology is primarily functional: it was engineered to expose current and historical ICTV taxonomy through OLS and the helper libraries so that taxon identity, lineage, synonyms and revision history can be queried programmatically. It does not import BFO, COB or RO, has not been submitted to the OBO Foundry, and no submission is currently planned.

Figure 2 illustrates how the entities and relations in the unified ontology support taxon resolution across ICTV releases. Release-specific taxon representations, modelled as OWL classes, are connected through PROV revision relations and, when a replacement is defined, through IAO:0100001 to the corresponding OWL class in the latest MSL, which represents the currently accepted ICTV taxon. In simple taxon rename paths, reusing the stable ICTV ID with the latest MSL release also retrieves the current OWL class, whereas IAO:0100001 provides a more direct route to the replacement OWL class and can resolve replacement or merge events without traversing the full release history. VMR virus exemplar records modelled as OWL named individuals are linked by rdf:type to their corresponding species OWL classes in the ontology. Together,

these relations allow identifiers and current or former taxon or virus names to be resolved to current taxa, to defined historical states or to complete taxon histories through the OLS API and helper libraries.

Availability and sustainability

Continuous Integration and Deployment (CI/CD) workflows implemented through public GitHub repositories automatically trigger the ontology generation workflow for each release published in the ICTV's official GitHub repository [28]. This automation removes manual intervention and contributes to long-term sustainability of the infrastructure, in line with recommendations for sustainable FAIR implementation in Life Sciences [29]. The unified ontology is immediately available as a release from the EVORA GitHub repository [30], and is automatically deployed to the OLS, typically within 24 hours of an ICTV release, which provides public web browsing and API access. Dissemination and long-term discoverability are further supported through versioned GitHub and Zenodo releases, as well as registration of the software resources in bio.tools and SciCrunch (see Availability of supporting source code and requirements section).

ICTV-NCBI mapping

To improve interoperability at the broader scale [31], ICTV-NCBI mappings are generated using the OLS API, and stored in SSSOM (Simple Standard for Sharing Ontology Mapping) format, aligning ICTV identifiers with NCBI Taxon identifiers [32]. The code to generate this mapping is maintained in a dedicated GitHub repository [33], where the resulting SSSOM files are then published as releases (see Additional file 1, Supplementary Material S4). While this analysis is also automated using a GitHub CI/CD workflow, it runs on a schedule, decoupled from the release cycles of the two ontologies, allowing independent updates while remaining consistent with the continuously updated ontology infrastructure.

These mappings not only facilitate direct and bidirectional linkage between ICTV and NCBI taxonomies, but also enable alignment across downstream resources that rely on NCBI as a taxonomic backbone, including sequence repositories and bioinformatics platforms. This feature enables broader platforms that rely on the NCBI taxonomy for viral references (e.g., ENA) to stay current with the ICTV taxonomy while continuing to leverage the broader coverage of NCBI taxonomy.

The NCBI integrates the ICTV Taxonomy into its own taxonomy system, which is used to designate species related to the entries in its sequence databases (e.g. GenBank). This integration is supported by an established exchange process between the ICTV database team and the NCBI team responsible for virus taxonomy updates. This process predates the present work and accommodates NCBI's specific sequence-linked update requirements. The public ICTV ontology API and ICTV-NCBI mappings complement this process by providing a general, community-facing and machine-actionable layer for accessing and reconciling current and historical ICTV taxonomy. Historically, ICTV updates have been incorporated with roughly a one-year delay, resulting in NCBI being one or two releases behind the latest ICTV version (as described above). Observations following MSL41 release suggest a recent effort by NCBI to shorten this delay, with progressive integration starting in May 2026, about two months after the MSL41 release. This included the integration of renamed taxa, while newly created taxa remained absent and abolished taxa had not yet been removed (see Additional file 1, Supplementary Material S3). As part of this work, ICTV has publicly documented several persistent identifiers (described above) that link taxa in successive ICTV Taxonomy versions, which should facilitate rapid integration of new releases. These identifiers are exposed in the ICTV ontology metadata and support the generation of SSSOM ICTV-NCBI mappings. In the released mapping file, lexical ICTV-to-NCBI Taxon correspondences are expressed with skos:exactMatch, enabling alignment between ICTV taxonomy and NCBI Taxonomy identifiers used by downstream sequence resources such as GenBank.

Results

Unified ontology for ICTV taxonomy and OLS deployment

We have produced a unified ontology for the ICTV taxonomy, aggregating all releases into a single resource. The ontology is available for download or via OLS and enables programmatic access to current and historical classifications. Ontology updates are automatically triggered by new ICTV releases and reflected in OLS, typically within 24 hours.

Asynchronously, ICTV-NCBI mappings are generated in SSSOM format, enabling bidirectional resolution between the taxonomies. Mappings are maintained in a dedicated EVORA virus-taxonomy-mappings repository and updated independently [33]. The mapping supports bidirectional many-to-many resolution between ICTV and NCBI taxonomies and facilitates integration with downstream resources. The overall architecture of the generation and publication workflow is shown in **Figure 3**.

To simplify integration into downstream applications, ICTV-specific helper libraries were developed on top of the standard OLS API. These libraries are provided in JavaScript, Python and PHP and encapsulate resolution logic (synonyms, obsolescence, lineage, mappings) while returning normalised data about taxon objects. These objects contain identifiers, labels, synonyms, ranks, lineage, obsolescence metadata and cross-references, enabling application developers to integrate ICTV-aware logic without direct manipulation of raw API calls. The helper libraries prioritise exact identifier and name-based routes before free-text fallback. A bare ICTV ID returns the corresponding OWL class from the latest matching MSL release by default, while an explicit MSL release retrieves the corresponding historical state. For VMR records represented in the ontology, a VMR identifier resolves through the corresponding OLS individual and its rdf:type relation to the current species OWL class.

The OLS API documentation, ICTV-specific guidance and helper libraries are publicly available [34] (see Additional file 1, Supplementary Material S1). Together, the unified ontology, its OLS deployment, the helper libraries and the complementary SSSOM mappings constitute an integrated ontology-based access resource for automated resolution, cross-taxonomy linkage and reproducible versioning.

Reference application and early adopters

A public demonstration interface, the ICTV Taxon Resolver [35] web application, was developed as a reference implementation using the JavaScript helper library. The interface provides two selectable actions. The default action, 'Resolve to latest ICTV', returns the current accepted ICTV taxon when one can be resolved. 'Show history across MSLs' starts from the OWL class representing the taxon identified from the query and displays the release-specific OWL classes linked to it across the MSL releases included in the ontology. Consequently, the displayed history may begin after MSL1 or end before MSL41, for example when a taxon was introduced later or subsequently abolished. The source code is available from the EVORA ICTV Resolver GitHub repository [36]. The resolver allows users to submit current and historical ICTV taxon names and virus names, ICTV identifiers, Internationalized Resource Identifiers (IRIs) or NCBI Taxon IDs and receive the cor-

responding currently accepted ICTV taxon or taxa, together with their full lineages and revision history. Concise usage guidance is displayed directly beneath the interface title. This tool illustrates practical applicability while serving as a functional community resource. The interface and an example resolution of the former species name *Zika virus* to its post-2022 name and lineage, *Orthoflavivirus zikaense*, are shown in **Figure 4**. For example, in history mode, the relationship between *Alphabaculovirus* and *Nucleopolyhedrovirus* illustrated in Figure 1 can be traced from MSL23 back to MSL13. The resolver is a demonstration and reference implementation built on the JavaScript helper library and does not expose every capability of the OLS-backed ICTV ontology API, for which complete developer documentation is available separately.

Among the first operational deployments using the ICTV ontology API is the EVORA Portal [37], a consolidated catalogue of research infrastructures' resources and services in virology designed to support outbreak response and pandemic preparedness. The utilisation of the ICTV ontology API is integrated through one of the helper libraries directly into the catalogue generation workflow, enabling retrieval of up-to-date taxonomic terms along with associated lineage and synonym information. This ensures consistent use of the latest ICTV taxonomy, even when metadata provided by contributing research infrastructures correspond to earlier ICTV releases. As an added value for users of the research infrastructures, lineage information and historical synonyms retrieved through the ontology API enable enhanced search capabilities. This allows users to find resources using outdated taxa or alternative names, an option that was not available in the former search module.

Early adoption of the ICTV ontology API has also been observed in an external initiative concerned with pandemic preparedness and data integration: the Pandemic PACT programme [38]. This programme is now using ICTV taxonomy via the OLS API to align viral pathogen families and scientific names within its scope with the latest ICTV taxonomy (see Acknowledgements section). The retrieved ICTV metadata will be incorporated into Pandemic PACT dashboard visualisations [39], where they will be integrated with metadata from other ontologies accessed through the OLS, such as the Systematized Nomenclature of Medicine (SNOMED), aligning up-to-date viral pathogen taxonomy with related information, including associated diseases.

Integration of ICTV taxonomy through an API enables timely and automated updates across resources. The European Virus Archive (EVA) [40], a network of distributed virus collections using a single web catalogue for virus and virus-related material ordering and distribution [41], plans to integrate the ICTV ontology into its workflows using OLS API queries to maintain local references to virus taxonomy aligned with the most recent ICTV releases. This will allow EVA datasets to remain synchronised with official taxonomy updates while preserving historical annotations associated with archived materials, which will enhance the interconnectivity of research and data management processes, improving resource discovery and data consistency.

These deployments demonstrate practical applicability across research infrastructures and public health platforms. The system enables automated resolution, cross-taxonomy linkage, reduced manual curation and reproducible versioning.

Discussion

Virus data resources include numerous databases with different scopes, data models, functionalities and degrees of FAIR maturity, as reflected in recent reviews of virus databases [42]. This heterogeneity creates practical challenges for interoperability and synchronisation with authoritative taxonomic references such as ICTV taxonomy. This work demonstrates how relational datasets can be easily transformed and exposed through scalable, public, ontology-based APIs. Standards-based ontology-enabled APIs drastically reduce the work needed to make datasets machine-actionable. Here, we developed an ontology for the widely used and regularly updated ICTV virus taxonomy and exposed it through a public API, providing downstream systems with programmatic access to current and historical taxonomic information and the ability to resolve former terms to the current classification, thereby supporting uses ranging from direct queries to recurring automated updates. The ontology infrastructure acts as a semantic resolution layer that continuously bridges evolving authoritative ICTV releases with downstream computational ecosystems and legacy taxonomic references. Timely updates, in turn, reduce discrepancies that arise when downstream systems rely on outdated or partially synchronised taxonomic references and enable reproducible taxonomic resolution across computational workflows. Research infrastructures, sequence repositories, biodiversity catalogues, and knowledge bases can use automated alignment mechanisms to maintain consistency with official ICTV classifications, reducing the need for extensive manual curation.

Persistent ICTV identifiers and replacement relations provide a structured mechanism to track taxonomic evolution, historically prone to ambiguity and data fragmentation, in a computationally accessible way. Complementary ICTV-NCBI mappings, together with ontology cross-references to associated sequence resources, provide a pragmatic bridge between the authoritative, annually updated ICTV classification and NCBI taxonomy. This improves reproducibility and supports FAIR principles, and their progressive implementation [43, 44, 45]. While this approach substantially improves interoperability and historical resolution, synchronisation across downstream resources still depends on heterogeneous update cycles and integration strategies. In addition, some complex taxonomic transitions, such as splits, merges, or taxon abolitions, may still require complementary expert interpretation in specific downstream analytical contexts.

These capabilities help connect distributed data systems in FAIR-aligned federated environments, where independently maintained resources must remain discoverable, accessible and interoperable, including initiatives such as the European Open Science Cloud (EOSC) and the European Health Data Space (EHDS).

Consistent and machine-actionable taxonomy references support large-scale computational analyses, automated data integration workflows and reproducible comparative studies by reducing ambiguity in taxonomic interpretation. This contributes to improved retrieval and harmonisation of virological data across interoperable research infrastructures and data-sharing environments [46], supporting more reliable data integration, comparative analyses, evidence-based decision making, and more informed experimental design and data interpretation. The infrastructure also establishes a foundation for future enhancements, including expanded mappings with sequence repositories and the possible development of additional interfaces relying on the public OLS API.

Our approach aligns with the article "Four principles to establish a universal virus taxonomy" [47] that outlines key recommendations to achieve a coherent and comprehensive virus taxonomy. Our work helps make ICTV taxonomy more useful by ensuring that references to the official, evolution-based ICTV taxonomy, can be easily maintained and kept up-to-date, irrespective of the intended use or alternate classification scheme. For example, classifying viruses to support the surveillance and public health response to disease outbreaks (Pathogens prioritization: a scientific framework for epidemic and pandemic research preparedness) [48] results in a classification that is significantly different than that of the ICTV taxonomy. It is however still important to reference the current evolutionary-based classification so that our current knowledge on viruses can be quickly and easily adapted to newly identified or newly emerging ones. The ICTV taxonomy serves as the universal reference for virus classification, while the services we report here enable the critical links needed

to connect that taxonomy to alternate schemes.

Adoption of the ICTV ontology API by resources relying on virus taxonomy (EVORA and Pandemic PACT) demonstrates its practical applicability. Ontology-based APIs can facilitate alignment between biological taxonomy resources and clinical terminologies used in public health systems, enabling consistent pathogen identification across research infrastructures, data catalogues, and policy-oriented dashboards. By supporting automated retrieval of up-to-date taxonomic terms, lineage information and synonym relationships, the API-based approach supports consistent taxonomy usage across distributed data infrastructures and reduces the need for manual synchronisation of taxonomic references similarly to interoperability approaches explored in tools such as Treemendous for reconciling taxonomic backbones in tree species naming [49].

Taxonomic resources and biodiversity platforms such as NCBI Taxonomy and GBIF could use the OLS API to implement more automated synchronisation with ICTV releases. Databases such as ENA and GenBank, which depend on the NCBI taxonomy and its integration of ICTV classifications [50], would in turn benefit from improved data integration and accuracy. In cases where updates of virus taxonomy within NCBI do not immediately reflect the latest ICTV release, these resources could additionally leverage the ICTV ontology API, coupled with the provided SSSOM mappings, to maintain up-to-date virus taxonomy.

Our work builds on established FAIR and semantic standards [25, 51]. The inclusion of detailed metadata and the reuse of established semantic standards are critical for ensuring that the ICTV taxonomy serves as a reliable reference across different platforms and applications. This approach provides a lightweight and sustainable mechanism for publishing structured datasets as interoperable APIs, by leveraging an existing ontology service (OLS) and GitHub's publicly available workflow automation. This approach is applicable to other domain-specific datasets. In parallel with the public OLS-based service, ICTV is using a dedicated access mechanism for data exchange with NCBI and plans a broader API providing access to a more comprehensive ICTV dataset. These complementary services support specialised or higher-volume exchanges, while the OLS API remains the public, standards-based access layer.

More broadly, this work illustrates how ontology technologies can expose structured datasets as semantically enriched graph representations through interoperable APIs. Traditional database schemas are optimised for storage and transactional operations but often require custom APIs to expose their content programmatically. In contrast, ontology-based representations allow relationships between entities to be explicitly encoded and navigated as a graph, enabling more flexible data integration and discovery. Such representations align with knowledge graph and Linked Open Data (LOD) approaches [52, 53]. More generally, this work demonstrates a reusable pattern for exposing structured, domain-specific datasets as interoperable ontologies and APIs by leveraging established ontology infrastructures, such as the OLS for biomedical ontologies.

Conclusion

This work shows that representing an evolving classification system, such as the ICTV taxonomy, as a unified ontology hosted through a public, scalable, standards-based API, increases its accessibility and addresses key challenges in access, versioning and interoperability. The resulting infrastructure supports backward-compatible resolution of virus taxonomy across historical ICTV releases, enabling historical traceability and alignment with current classifications through semantic modelling, persistent identifiers and interoperable mappings.

By building on open standards, automated workflows and existing ontology infrastructure services, this approach supports long-term sustainability and scalability while making ICTV taxonomy more readily integrable into computational workflows. It also facilitates synchronisation between authoritative taxonomic references and downstream biological, biomedical and public-health data infrastructures, supporting broader adoption of the latest official ICTV taxonomy as the reference standard and strengthening data consistency, reproducibility and preparedness for emerging viral threats, including through more timely dissemination of high-impact updates such as the pandemic-based designation of the species *Betacoronavirus pandemicum*.

Future developments may support broader interoperability with additional taxonomy providers, sequence repositories and interconnected semantic resources. More broadly, this work provides a reusable model for maintaining, exposing and reusing evolving taxonomic references through sustainable, machine-actionable semantic infrastructure.

Declarations

Ethics approval and consent to participate

Not applicable.

Consent for publication

Not applicable.

Funding

This work was supported by the EVORA project (European Union's HORIZON programme grant agreement N° 101131959). The contribution of the University of Alabama at Birmingham (UAB) team to the research reported in this publication was supported by the National Institute of Allergy and Infectious Diseases of the National Institutes of Health under Award Number U24AI162625. The content is solely the responsibility of the authors and does not necessarily represent the official views of the National Institutes of Health. The UAB team assisted with accessing all viral taxonomic data and associated metadata. The UAB team also served as consultants to assist in the understanding of the underlying ICTV data structures and data organization that were used to construct the ontology. In total, the UAB effort comprised approximately 10% of the overall effort.

Acknowledgements

This work is a product of the EVORA project (European Viral Outbreak Response Alliance) and the ICTV's UAB team.

We gratefully acknowledge Emilia Antonio and Alice Norton from the Pandemic PACT programme for their feedback on the adoption of the ICTV OLS API within Pandemic PACT to align viral pathogen families and scientific names with the latest ICTV taxonomy identifiers.

Authors' contributions

Based on Contributor Roles Taxonomy (CRediT) [54]

- Conceptualization: PL
- Project administration: PL, BC
- Resources: CH, EL, PL, BC, JM, HP
- Software: JM, PL
- Methodology: PL, JM, HP, RD, CH

- Writing – original draft: PL, RD
- Writing – review & editing: PL, RD, HP, JM, BC, CH, EL
- Visualization: PL, RD, CH, DMD

Disclosure of use of AI-assisted tools including generative AI

- During manuscript preparation and revision in 2024-2026, Philippe Lieutaud, Romain David and R. Curtis Hendrickson used OpenAI ChatGPT, initially with GPT-4o and subsequently with GPT-5-series models, including GPT-5.4, GPT-5.5 and GPT-5.6, at different stages as a writing and editorial aid [55]. The tool was used to suggest rewording and stylistic improvements, identify redundancy, improve the organisation of arguments, check conformity with journal requirements during final review, including reference formatting and the availability of cited links, and suggest improvements to figure layout and presentation. The AI-assisted textual and figure-design suggestions were treated as conceptual proposals and were not incorporated verbatim; no AI-generated images were used. The authors determined and validated all final scientific content, and produced the final figures.
- Philippe Lieutaud also used OpenAI ChatGPT with GPT-5.4 and GPT-5.5 to assist in drafting user and developer documentation published in the ICTV ontology and ICTV Resolver GitHub repositories. He used the same models to assist in porting the ICTV API helper library from Python to JavaScript and PHP, and to check consistency across the three implementations. GPT-5.5 was also used to suggest fixes for Zenodo release synchronisation involving .zenodo.json metadata and new GitHub release events in the ICTV ontology and ICTV-NCBI mapping repositories, and to help design a sharded, failure-resilient strategy for long-running ICTV-NCBI mapping updates. GPT-5.5 also assisted in developing a local script to convert the Word manuscript to LaTeX; Philippe Lieutaud produced and checked the final LaTeX files. The resulting documentation, code and workflow changes were reviewed and validated by the authors against the intended functionality of the libraries, release processes, mapping workflows and OLS API.
- Separately, on 7 October 2025, James McLaughlin used the GitHub Copilot coding agent (underlying model not recorded) to generate initial GitHub Actions implementations for building and releasing the ICTV ontology and for scheduling updates of the ICTV-NCBI mappings [56]. In the mapping repository, Copilot also drafted associated documentation and an initial modification supporting the incremental reuse of existing mappings. These contributions are preserved in the public development history of the corresponding repositories. The generated code was reviewed and merged by James McLaughlin and subsequently revised and extended by the authors.
- The AI-assisted tools were not used to generate scientific data or independently interpret the results. All generated suggestions and code contributions were critically reviewed and validated by the authors, who take full responsibility for the content of the manuscript and the associated software.

Competing interests

The authors declare that they have no competing interests.

Data availability

All ontology artefacts, helper libraries, mapping resources, example notebooks, and continuous-integration workflows are publicly available under open licences. The ICTV MSL41 source data used in this work are available as the ICTVdatabase MSL41.v1 release [21]. The exact EVORA ICTV ontology release used for this manuscript is archived in Zenodo as EVORA-project/ictv-ontology: 2026-Jul-28-74d7801 doi:10.5281/zenodo.21506852 [57]. The concept DOI 10.5281/zenodo.20610328 resolves to all versions. The exact ICTV-NCBI mapping release is archived in Zenodo as EVORA-project/virus-taxonomy-mappings: Virus Taxonomy Mappings Update, release-3 doi:10.5281/zenodo.21890424 [58]. The complete version history is accessible through the Zenodo concept DOI doi:10.5281/zenodo.20610592. Current development versions and latest releases are available from the public repositories listed below:

- ICTV database: https://github.com/ICTV-Virus-Knowledgebase/ICTVdatabase/
- ICTV ontology generation code, helper libraries, and generated ontologies (OWL/Turtle files): https://github.com/EVORA-project/ictv-ontology
- Latest ICTV ontology release: https://github.com/EVORA-project/ictv-ontology/releases/latest
- ICTV-NCBI SSSOM mappings: https://github.com/EVORA-project/virus-taxonomy-mappings
- Latest ICTV-NCBI mapping release: https://github.com/EVORA-project/virus-taxonomy-mappings/releases/latest
- Unified ICTV ontology in OLS: https://www.ebi.ac.uk/ols4/ontologies/ictv [59]

All resources are reusable, openly licensed and versioned, enabling independent verification and downstream integration.

NCBI Taxonomy taxdump archives used to verify the integration status of ICTV releases are available from the NCBI taxonomy archive: https://ftp.ncbi.nih.gov/pub/taxonomy/taxdump_archive/ [60]

Availability of supporting source code and requirements

- Project name: ICTV Ontology API and helper libraries
  - Project home page: https://github.com/EVORA-project/ictv-ontology
  - Documentation: https://evora-project.github.io/ictv-ontology/helpers/
  - Operating system(s): Platform independent
  - Programming language: language-agnostic for direct API access; helper libraries are provided in Python 3.8 or later, JavaScript as an ES module, and PHP 7.4 or later.
  - Other requirements: Internet access to the OLS API; depending on the helper library used, the Python requests library, the JavaScript Fetch API or the PHP cURL extension is required. For PHP, allow_url_fopen must also be enabled when accessing the remote ICTV-NCBI SSSOM mapping.
  - Licences:
    * ontology-generation code, Apache License 2.0;
    * ICTV-derived ontology data, Creative Commons Attribution 4.0 International (CC BY 4.0), with attribution to ICTV;
    * JavaScript, Python and PHP helper libraries, MIT License.
  - Version used for this manuscript: GitHub release 2026-Jul-28-74d7801; Zenodo version DOI 10.5281/zenodo.21506852
  - All versions: 10.5281/zenodo.20610328
  - RRID: SCR_028724
  - bio.tools ID: ictv_ontology_api
- Project name: ICTV-to-NCBITaxon SSSOM mapping
  - Project home page: https://github.com/EVORA-project/v

irus-taxonomy-mappings

- Operating system(s): Platform independent
- Programming language: language-agnostic for SSSOM file utilisation; SSSOM file generation code in Python.
- Release archived at https://doi.org/10.5281/zenodo.21890424 [58]
- License: CC0 1.0 Universal
- RRID: SCR_028725
- bio.tools ID: ictv_to_ncbitaxon_sssom_mapping

- Project name: ICTV Taxon Resolver
  - Project home page: https://github.com/EVORA-project/ictv-resolver
  - Live instance: https://evora-project.github.io/ictv-resolver
  - Programming languages: HTML, JavaScript and CSS.
  - Other requirements: A modern web browser with JavaScript and internet access to OLS.
  - License: MIT.
  - Version used: v1.0.5.
  - RRID: SCR_028722
  - bio.tools ID: ictv_taxon_resolver

- External service dependency: Ontology Lookup Service (OLS):
  - Project home page: https://www.ebi.ac.uk/ols4/
  - Documentation: https://github.com/EBISPOT/ols4/blob/dev/README.md [61]
  - Source code: https://github.com/EBISPOT/ols4
  - Software licence: Apache License 2.0

List of abbreviations

- **API(s):** Application Programming Interface(s)
- **CI/CD:** Continuous Integration and Deployment
- **COL:** Catalogue of Life
- **CURIE:** Compact Uniform Resource Identifier
- **DCTERMS:** Dublin Core Terms namespace
- **ENA:** European Nucleotide Archive
- **ETL process:** Extract, Transform, and Load
- **EVA:** European Virus Archive
- **EVORA:** European Viral Outbreak Response Alliance.
- **FAIR:** Findable, Accessible, Interoperable and Reusable
- **FOAF**: Friend of a Friend ontology
- **GBIF:** Global Biodiversity Information Facility
- **IAO:** Information Artifact Ontology
- **ICTV:** International Committee on Taxonomy of Viruses
- **IRI**: Internationalized Resource Identifier
- **LOD:** Linked Open Data
- **MCP:** Model Context Protocol
- **MSL:** Master Species List
- **NCBI:** National Center for Biotechnology Information
- **OLS:** Ontology Lookup Service
- **OWL:** Web Ontology Language
- **PROV-O**: Provenance Ontology
- **RDFS:** Resource Description Framework Schema
- **REST:** Representational State Transfer
- **SKOS:** Simple Knowledge Organization System
- **SNOMED:** Systematized Nomenclature of Medicine
- **SSSOM:** Simple Standard for Sharing Ontology Mapping
- **TAXRANK:** Taxonomic Rank Vocabulary
- **VMR:** Virus Metadata Resource

References

1. International Committee on Taxonomy of Viruses (ICTV). ICTV website. 2026. https://ictv.global/. Accessed 09 June 2026.
2. International Union of Microbiological Societies (IUMS). IUMS website. 2026. https://www.iums.org/. Accessed 09 June 2026.
3. Zerbini FM, Siddell SG, Mushegian AR, Walker PJ, Lefkowitz EJ, et al. Differentiating between viruses and virus species by writing their names correctly. Arch Virol 2022;167:1231–1234. doi:10.1007/s00705-021-05323-4
4. International Committee on Taxonomy of Viruses (ICTV). ICTV Master Species List (MSL). 2026. https://ictv.global/msl. Accessed 09 June 2026.
5. Simmonds P, Adriaenssens EM, Lefkowitz EJ et al. Changes to virus taxonomy, the international code of virus classification and nomenclature, and the ICTV statutes ratified by the International Committee on Taxonomy of Viruses (2025). *Arch Virol* 2026;171:23. doi:10.1007/s00705-025-06485-1
6. Black EJ, et al. Virus taxonomy: the database of the International Committee on Taxonomy of Viruses. Nucleic Acids Res 2026;54(D1):D776–D789. doi:10.1093/nar/gkaf1159
7. National Center for Biotechnology Information (NCBI). NCBI website. 2026. https://www.ncbi.nlm.nih.gov/. Accessed 09 June 2026.
8. GBIF. Global Biodiversity Information Facility website. 2026. https://www.gbif.org/. Accessed 09 June 2026.
9. Catalogue of Life. Catalogue of Life (COL). 2026. https://www.catalogueoflife.org/. Accessed 09 June 2026.
10. Wikidata. Wikidata. 2026. https://www.wikidata.org/. Accessed 09 June 2026.
11. National Center for Biotechnology Information (NCBI). GenBank. 2026. https://www.ncbi.nlm.nih.gov/genbank/. Accessed 09 June 2026.
12. European Nucleotide Archive (ENA). ENA Browser. 2026. https://www.ebi.ac.uk/ena/browser/home. Accessed 09 June 2026.
13. Boyle B, Hopkins N, Lu Z, et al. The taxonomic name resolution service: an online tool for automated standardization of plant names. BMC Bioinformatics 2013;14:16. doi:10.1186/1471-2105-14-16
14. EVORA. European Viral Outbreak Response Alliance project website. 2026. https://evora-project.eu/. Accessed 09 June 2026.
15. European Commission. European Viral Outbreak Response Alliance (EVORA), grant agreement ID 101131959. CORDIS. 2026. https://doi.org/10.3030/101131959. Accessed 09 June 2026.
16. Malone J, Stevens R, et al. Ten simple rules for selecting a bio-ontology. PLoS Comput Biol 2016;12(2):e1004743. doi:10.1371/journal.pcbi.1004743
17. McLaughlin J, Lagrimas J, Iqbal H, et al. OLS4: a new Ontology Lookup Service for a growing interdisciplinary knowledge ecosystem. Bioinformatics 2025;41(5):btaf279. doi:10.1093/bioinformatics/btaf279
18. EMBL-EBI. Ontology Lookup Service (OLS4). 2026. https://www.ebi.ac.uk/ols4/. Accessed 09 June 2026.
19. McMurry JA, Juty N, Blomberg N, et al. Identifiers for the 21st century: how to design, provision, and reuse persistent identifiers. PLoS Biol 2017;15(6):e2001414. doi:10.1371/journal.pbio.2001414
20. International Committee on Taxonomy of Viruses (ICTV). ICTV Virus Metadata Resource (VMR). 2026. https://ictv.global/vmr. Accessed 09 June 2026.
21. Hendrickson RC, Mims L, Lefkowitz EJ. ICTV-Virus-Knowledgebase/ICTVdatabase: Taxonomy Release: MSL41.v1 20260320 [Dataset]. Zenodo 2026. doi:10.5281/zenodo.19339543

22. Kibbe WA, Arze C, Felix V, et al. Disease Ontology 2015 update: an expanded and updated database of human diseases for linking biomedical knowledge through disease data. Nucleic Acids Res 2015;43(D1):D1071–D1078. doi:10.1093/nar/gku1011
23. Lebo T, Sahoo S, McGuinness D, et al. PROV-O: the PROV ontology. W3C Recommendation 2013. https://www.w3.org/TR/prov-o/. Accessed 12 May 2026.
24. Miles A, Bechhofer S. SKOS simple knowledge organization system reference. W3C Recommendation 2009. https://www.w3.org/TR/skos-reference/. Accessed 12 May 2026.
25. Xu F, Juty N, Goble C, Jupp S, Parkinson H, Courtot M. Features of a FAIR vocabulary. J Biomed Semantics 2023;14(1):6. doi:10.1186/s13326-023-00286-8
26. Smith B, Ashburner M, Rosse C, et al. The OBO Foundry: coordinated evolution of ontologies to support biomedical data integration. Nat Biotechnol 2007;25(11):1251–1255. doi:10.1038/nbt1346
27. Fielding RT, Taylor RN. Principled design of the modern Web architecture. ACM Trans Internet Technol 2002;2(2):115–150. doi:10.1145/514183.514185
28. International Committee on Taxonomy of Viruses (ICTV). ICTV database GitHub repository. 2026. https://github.com/ICTV-Virus-Knowledgebase/ICTVdatabase/. Accessed 09 June 2026.
29. David R, Rybina A, Burel J, et al. "Be sustainable": EOSC-Life recommendations for implementation of FAIR principles in life science data handling. EMBO J 2023;42:e115008. doi:10.15252/embj.2023115008
30. EVORA Project. EVORA ICTV ontology GitHub repository. 2026. https://github.com/EVORA-project/ictv-ontology. Accessed 09 June 2026.
31. David R, Baumann K, LeFranc Y, et al. Converging on a semantic interoperability framework for the European Data Space for Science, Research and Innovation (EOSC). In: Proceedings of the 2nd Workshop on Ontologies for FAIR and FAIR Ontologies (Onto4FAIR). 2023. doi:10.5281/zenodo.8102786
32. Matentzoglu N, Balhoff J, Bello S, et al. A simple standard for sharing ontological mappings (SSSOM). Database 2022;2022:baac035. doi:10.1093/database/baac035
33. EVORA Project. EVORA virus-taxonomy-mappings GitHub repository. 2026. https://github.com/EVORA-project/virus-taxonomy-mappings. Accessed 09 June 2026.
34. EVORA Project. EVORA ICTV ontology helper and API documentation. 2026. https://evora-project.github.io/ictv-ontology/helpers/. Accessed 09 June 2026.
35. EVORA Project. EVORA ICTV Taxon Resolver live interface. 2026. https://evora-project.github.io/ictv-resolver. Accessed 09 June 2026.
36. EVORA Project. EVORA ICTV Taxon Resolver GitHub repository. 2026. https://github.com/EVORA-project/ictv-resolver. Accessed 09 June 2026.
37. EVORA Project. EVORA Portal catalogue. 2026. https://portal.evora-project.eu/. Accessed 09 June 2026.
38. Pandemic PACT. Programme website. 2026. https://www.pandemicpact.org/. Accessed 09 June 2026.
39. Pandemic PACT. Dashboard visualisations. 2026. https://www.pandemicpact.org/grants/visualise. Accessed 09 June 2026.
40. European Virus Archive (EVA). EVA website. 2026. https://www.european-virus-archive.com/. Accessed 09 June 2026.
41. Romette JL, Prat CM, Gould EA, et al. The European Virus Archive goes global: a growing resource for research. Antiviral Res 2018;158:127–134. doi:10.1016/j.antiviral.2018.07.017
42. Ritsch M, Cassman NA, Saghaei S, Marz M. Navigating the Landscape: A Comprehensive Review of Current Virus Databases. *Viruses* 2023;15(9):1834. doi:10.3390/v15091834
43. Wilkinson MD, Dumontier M, Aalbersberg IJ, et al. The FAIR guiding principles for scientific data management and stewardship. Sci Data 2016;3:160018. doi:10.1038/sdata.2016.18
44. Sansone SA, McQuilton P, Rocca-Serra P, et al. FAIRsharing as a community approach to standards, repositories and policies. Nat Biotechnol 2019;37(4):358–367. doi:10.1038/s41587-019-0080-8
45. David R, Mabile L, Specht A, et al. FAIRness literacy: the Achilles' heel of applying FAIR principles. Data Sci J 2020;19(1):32. doi:10.5334/dsj-2020-032
46. Research Data Alliance COVID-19 Working Group. Recommendations and guidelines on data sharing. 2020. doi:10.15497/rda00052
47. Simmonds P, Adriaenssens EM, Zerbini FM, et al. Four principles to establish a universal virus taxonomy. PLoS Biol 2023;21(2):e3001922. doi:10.1371/journal.pbio.3001922
48. World Health Organization (WHO). Pathogens prioritization: a scientific framework for epidemic and pandemic research preparedness. Meeting report. 2024. https://www.who.int/publications/m/item/pathogens-prioritization-a-scientific-framework-for-epidemic-and-pandemic-research-preparedness. Accessed 09 June 2026.
49. Specker F, Paz A, Crowther TW, Maynard DS. Treemendous: an R package for integrating taxonomic information across backbones. PeerJ 2024;12:e16896. doi:10.7717/peerj.16896
50. Schoch CL, Ciufo S, Domrachev M, et al. NCBI taxonomy: a comprehensive update on curation, resources and tools. Database 2020;2020:baaa062. doi:10.1093/database/baaa062
51. Berners-Lee T. Linked Data. 2006. https://www.w3.org/DesignIssues/LinkedData.html. Accessed 09 June 2026.
52. Hogan A, Blomqvist E, Cochez M, et al. Knowledge graphs. ACM Comput Surv 2021;54(4):1–37. doi:10.1145/3447772
53. Kamdar MR, Musen MA. An empirical meta-analysis of the life sciences linked open data on the web. Sci Data 2021;8:24. doi:10.1038/s41597-021-00797-y
54. National Information Standards Organization (NISO). Contributor Roles Taxonomy (CRediT). 2026. https://credit.niso.org/. Accessed 09 June 2026.
55. OpenAI (2026). ChatGPT (GPT-4o and GPT-5-series models, including GPT-5.4, GPT-5.5 and GPT-5.6) [large language models]. Responses to queries made by Philippe Lieutaud, Romain David and R. Curtis Hendrickson during manuscript preparation and revision, June 2024–August 2026. https://chatgpt.com/. Accessed 24 August 2026.
56. GitHub (2025). GitHub Copilot coding agent (underlying model not recorded) [AI-assisted coding agent]. Responses to tasks initiated by James McLaughlin, 7 October 2025. https://github.com/EVORA-project/ictv-ontology/pull/45. Accessed 09 June 2026; https://github.com/EVORA-project/virus-taxonomy-mappings/pull/2. Accessed 09 June 2026.
57. McLaughlin J, Lieutaud P, Hendrickson C. EVORA-project/ictv-ontology: 2026-Jul-28-74d7801 [Software]. Zenodo 2026. doi:10.5281/zenodo.21506852
58. McLaughlin J, Lieutaud P. EVORA-project/virus-taxonomy-mappings: Virus Taxonomy Mappings Update. Version release-3 [Software]. Zenodo 2026. doi:10.5281/zenodo.21890424
59. EMBL-EBI. Unified ICTV ontology in OLS. 2026. https://www.ebi.ac.uk/ols4/ontologies/ictv. Accessed 09 June 2026.
60. National Center for Biotechnology Information (NCBI). NCBI Taxonomy archive (taxdump archive). 2026. https://ftp.ncbi.nih.gov/pub/taxonomy/taxdump_archive/. Accessed 09 June 2026.
61. EMBL-EBI. OLS4 server documentation. 2026. https://github.com/EBISPOT/ols4/blob/dev/README.md. Accessed 09 June 2026.

Additional files

Additional file 1

File name: Additional_file_1_Supplementary_material.pdf

File format: .pdf

Title: Supplementary materials—API use, helper libraries, provider landscape and mapping specification

Description: Supplementary Materials S1–S4 provide API usage examples, the helper-library architecture, a comparison of virus taxonomy providers and the ICTV-NCBI mapping specification.

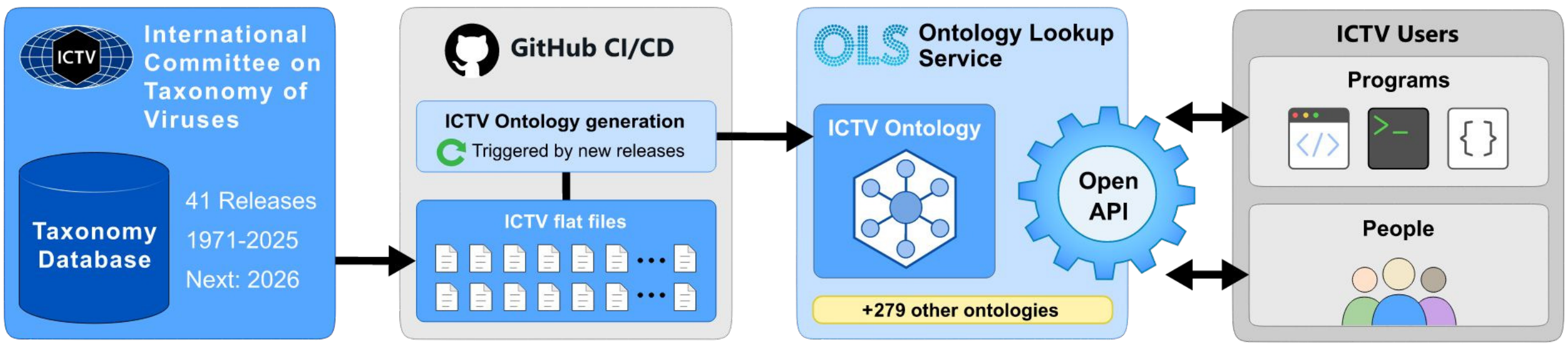

Graphical abstract. Unified ICTV ontology generation and public API access. Diagram showing 41 International Committee on Taxonomy of Viruses (ICTV) taxonomy releases transformed through GitHub continuous integration and continuous delivery (CI/CD) into a unified ICTV ontology hosted by the Ontology Lookup Service (OLS). An open application programming interface (API) provides programmatic access for software and browsing access for users.

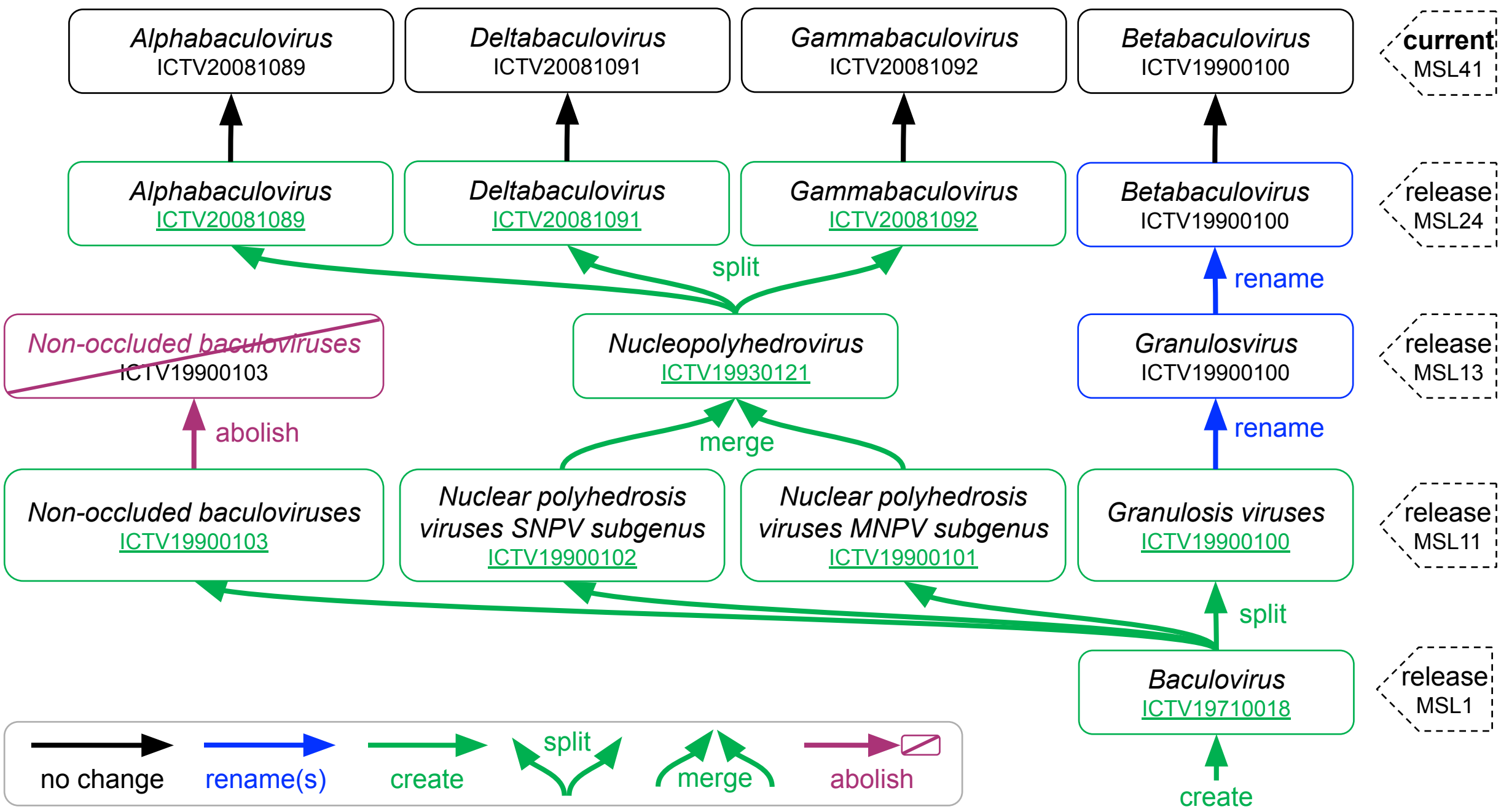

Figure 1. Illustrative taxonomic changes across ICTV releases for a genus-level taxon first recorded in MSL1. A diagram of historical and current genus names originating with the genus *Baculovirus* (ICTV19710018), first recorded in the first ICTV release, and ending with the four currently recognised genera. This example illustrates how the grouping of species into genera, although species are not shown here, can be redefined over time as viruses are better understood and characterised. In this historical trajectory, the original genus was first split into four genera; two of these were then merged; and the resulting lineage was later split into three genera. This illustrates some of the complexity that can occur in the history of a single taxon, and more broadly how taxonomic changes across different ranks and releases can make the overall taxonomy difficult to trace. It also shows how ICTV IDs are assigned to link historical versions of the same taxon. When a taxon is created, split or merged (green single, diverging or converging arrows, respectively), a new ICTV ID is assigned (underlined and in green). When taxa are renamed, promoted, demoted or moved, the ICTV ID remains the same, linking the different versions.

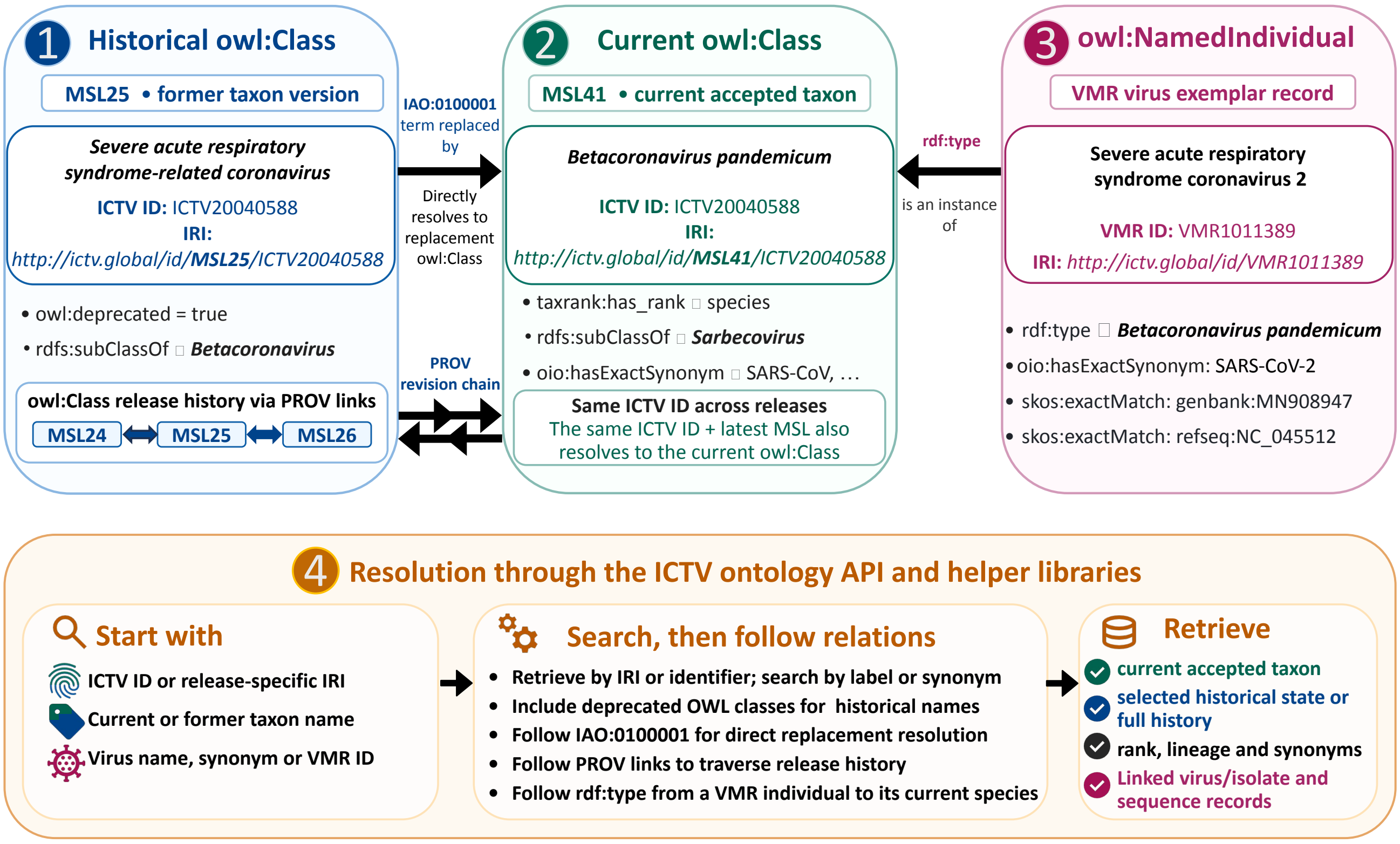

Figure 2. Historical and current taxon resolution using ICTV taxon and VMR virus record information. A former MSL25 taxon version is represented as a release-specific OWL class (i.e. owl:Class) (1), marked as deprecated with "owl:deprecated = true", and linked through IAO:0100001 to its current MSL41 representation, while PROV relations connect successive historical release states. The current accepted taxon is represented by a distinct OWL class carrying the same stable ICTV identifier (2). In simple taxon rename paths, this identifier can also be combined with the latest MSL release to retrieve the corresponding current OWL class. A VMR virus exemplar record is represented as an owl:NamedIndividual and asserted as an instance of its corresponding species OWL class using rdf:type (3). The resolution workflow (4) shows how ICTV identifiers and release-specific IRIs, current or former taxon names, and virus names, synonyms or VMR identifiers can be used through the ICTV ontology API and helper libraries to retrieve current taxa, historical states, lineage, synonyms and linked sequence records. In the context of this ontology, an OWL class represents an ICTV taxon and should not be confused with the ICTV rank class; rdfs:subClassOf expresses hierarchical relations between these ontology entities. The statement owl:deprecated = true applies to all historical, release-specific OWL classes representing taxa. Deprecated OWL classes linked by either an IAO:0100001 (replaced by) relation or prov:hadRevision link have been updated in a subsequent release. Only OWL classes with owl:deprecated = true and neither an IAO:0100001 relation nor a prov:hadRevision link represent truly abolished taxa.

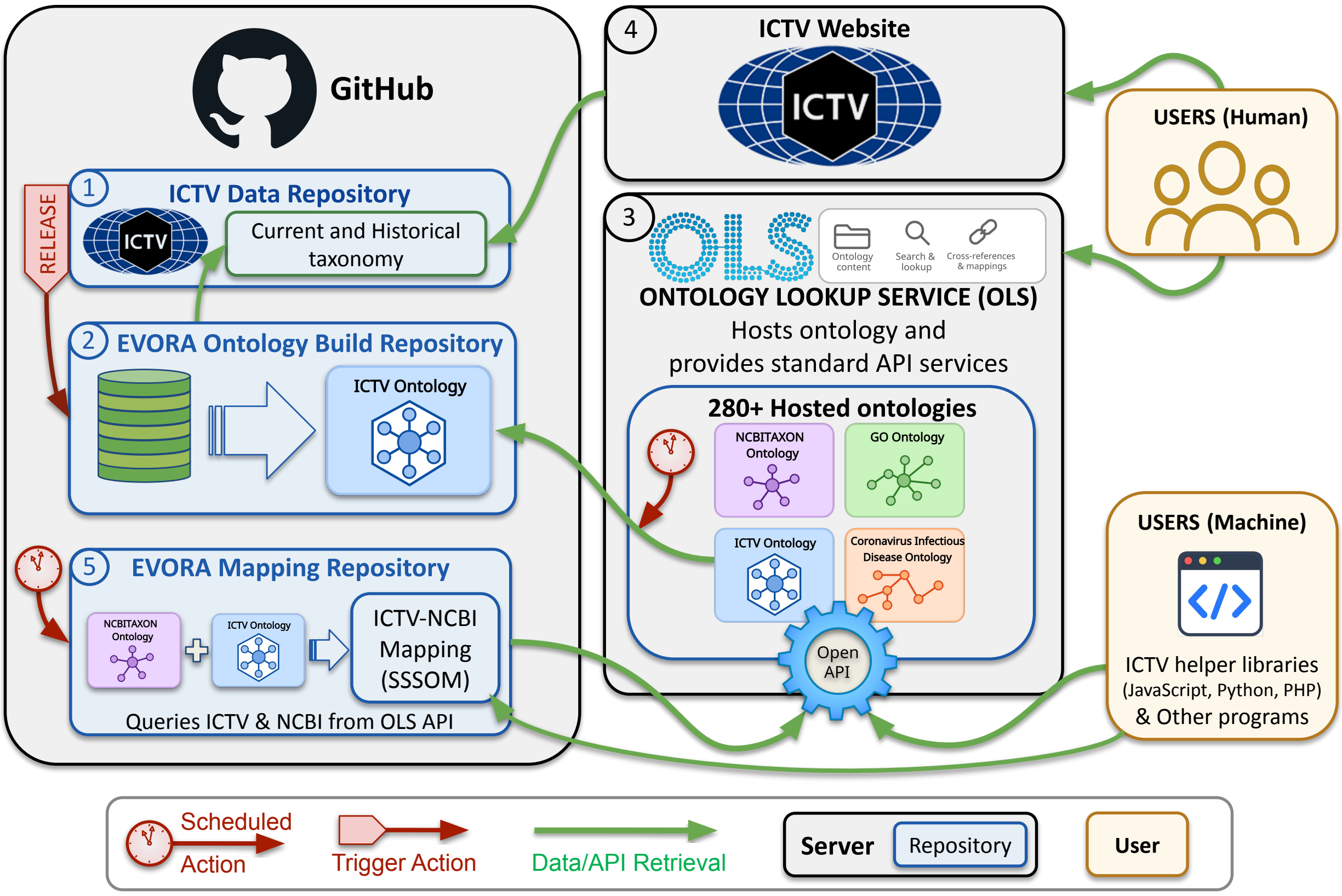

Figure 3. Automated data flows for ICTV ontology generation, publication, ICTV-NCBI mapping and API access. Official ICTV taxonomy data are released through the public ICTV Data repository (1), hosted on GitHub. A new release triggers the EVORA CI/CD workflow hosted in the EVORA Ontology Build repository (2), which retrieves the ICTV release files, transforms them into OWL ontology artefacts and merges them into a unified ICTV ontology. In this unified ontology, the latest ICTV release represents the current authoritative taxonomy, while entities from previous releases are retained as deprecated historical OWL classes linked through explicit semantic relations. Through scheduled actions, the unified ontology is loaded into the Ontology Lookup Service (OLS) (3), which provides both a browsable human interface and standard API services. The same ICTV source data also feed the ICTV website (4), which provides the official human-facing access point to ICTV taxonomy. In parallel, a separate EVORA Mapping repository (5) runs scheduled workflows that query OLS to use the latest ICTV and NCBITaxon ontologies to generate ICTV–NCBI mappings in SSSOM format; these mappings are stored in the corresponding public repository. Downstream access is supported through direct API queries and language-specific OLS ICTV API helper libraries, enabling external applications to perform historical taxon resolution, cross-resource mapping and to reduce manual curation. Green arrows indicate data/API retrieval actions; dark red arrows indicate workflow trigger actions; dark red clocks indicate scheduled actions. Black boxes represent servers, blue boxes repositories and brown boxes users.

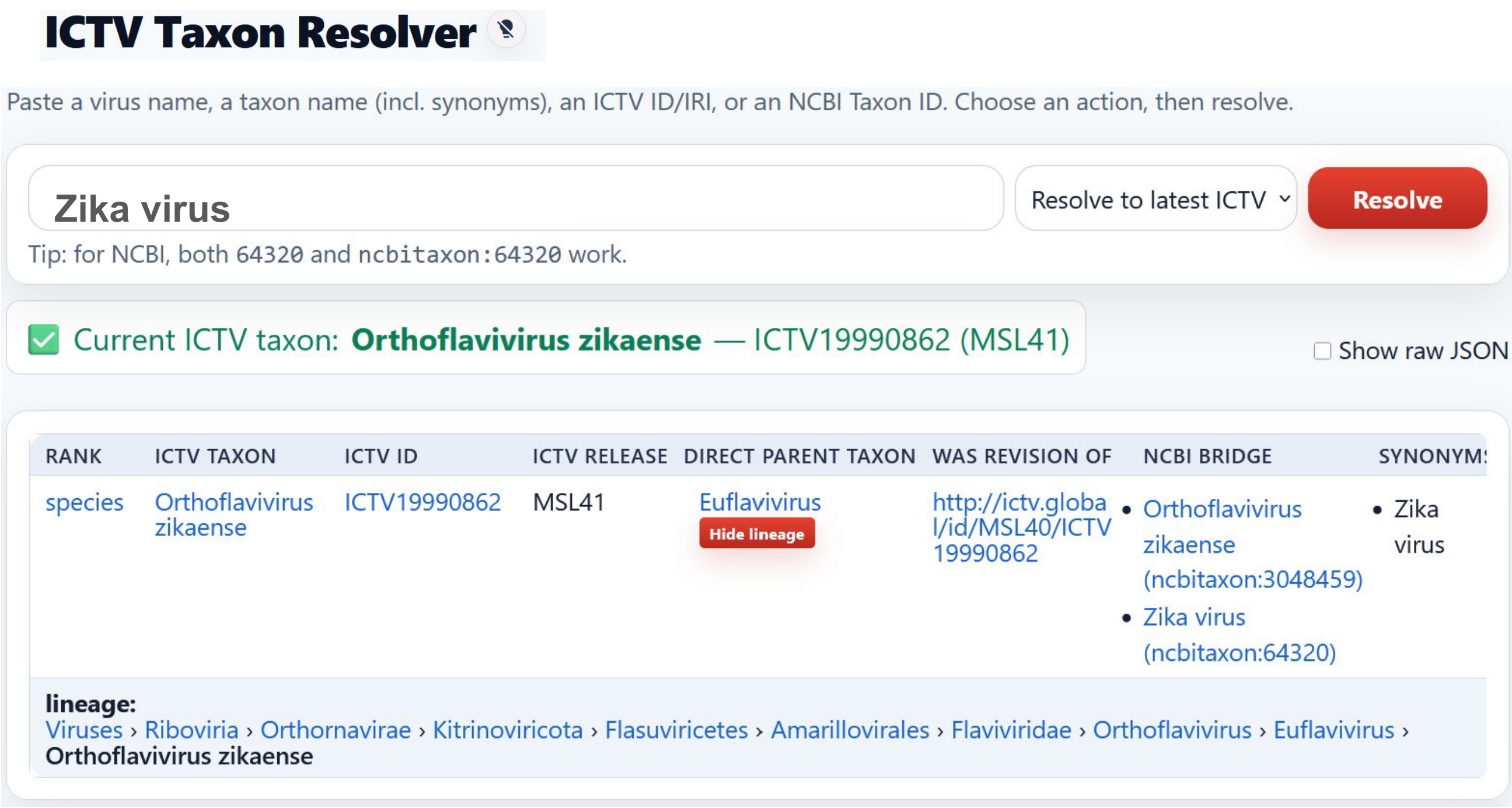

Figure 4. ICTV Taxon Resolver and programmatic taxonomic resolution. The ICTV Taxon Resolver interface illustrates automated resolution of historical or synonymous virus names to the currently accepted ICTV taxon, including lineage reconstruction and cross-references to external identifiers. The resolver is a reference implementation built on the JavaScript helper library that encapsulates Ontology Lookup Service (OLS) API calls. It accepts heterogeneous user inputs, including historical virus names, ICTV identifiers, Internationalized Resource Identifiers (IRIs), synonyms and NCBI Taxon identifiers, and applies a resolution strategy combining label matching, synonym detection, obsolete-term replacement chains and cross-taxonomy mappings. The output panel displays the currently accepted ICTV taxon together with its hierarchical lineage, rank, revision history and available cross-references to external resources.

Point-by-point response to reviewers Click here to access/download;Manuscript;point_by_point_response_to_revie

POINT-BY-POINT RESPONSE TO REVIEWERS

Manuscript: Programmatic access to ICTV virus taxonomy through a public ontology API

Article type: Technical Note

Dear Editor and Reviewers,

We thank the Editor and Reviewers for their careful and constructive assessment. We revised the manuscript and supporting resources accordingly. The comments concerned clarification, documentation and presentation; no new scientific data or analyses were required. The responses below address the reviewer comments point by point, and all substantive revisions are described below and incorporated in the revised manuscript.

Summary of principal revisions

- Clarified complete MSL1–MSL41 coverage, the public release-specific IRI design and the source-only role of TN identifiers.
- Added Figure 2 and accompanying text explaining historical and current taxon resolution, PROV and replacement relations, and VMR isolates as OWL NamedIndividuals linked to species OWL classes.
- Clarified the complementary roles of ICTV, NCBI Taxonomy, the public ontology API and the ICTV–NCBI mappings; named and archived the mapping resource.
- Expanded direct API, helper-library and Taxon Resolver documentation, including worked examples in Supplementary Material S1 and the Figure 4 result screenshot.
- Applied terminology, accessibility and presentation revisions, including context-sensitive use of 'virus taxonomy', qualified FAIR language and the colour-blind-safe Figure 1 palette.

Location note. Section locations are provided below and correspond to the clean, line-numbered revised manuscript.

Response to Reviewer 1

We thank the reviewer for the detailed comments and respond point by point below.

R1.1

Comment

> Have NCBI been included in conversations about these developments? It seems strange that representatives from EMBL-EBI are authors but there is no inclusion of partners from NCBI when NCBITaxon is a key resource for many platforms, application ontologies, and tools. Could the authors mention engagement efforts with NCBI so we

> understand whether the ICTV IDs and synonymy will be adopted? I think this is important as well as the authors pointedly describe the challenges of updating taxonomy at NCBI, but don't note that major consumers/providers of data are public health agencies. Every change in NCBITaxon breaks critical workflows in systems used in routine public health operations. NCBI is caught between a rock and a hard place every time ICTV makes changes - wanting to respect expert recommendations while managing pushback from the community e.g. there were/are many challenges of adopting the change of 'Severe acute respiratory syndrome-related coronavirus' to 'Betacoronavirus pandemicum' including adapting communication strategies with the public and Ministries of Health. Understanding communication/collaborations with NCBI would be helpful.

Response. We thank the reviewer for this important point. We agree that the respective roles of ICTV and NCBI Taxonomy, as well as the existing interactions between them, required clearer explanation.

ICTV is responsible for establishing the internationally agreed taxonomy and nomenclature of viruses. NCBI Taxonomy has a different operational role, linking taxonomic identifiers to a very large number of sequence records and downstream resources. Consequently, the incorporation of ICTV changes into NCBI cannot always be implemented as a simple replacement of a name or taxon. Abolitions, splits, merges, moves and other changes must be handled in a way that preserves continuity for existing records and dependent workflows.

NCBI staff were not directly involved in developing the public ICTV ontology API described in this manuscript, and we do not claim that NCBI has committed to adopting it. The API is publicly available and could be used by NCBI or other downstream systems, but its main purpose is to provide a general, community-facing and machine-actionable access layer for current and historical ICTV taxonomy.

The ICTV database team, represented among the authors of this manuscript, has an established working relationship with the NCBI team responsible for incorporating virus taxonomy updates. This relationship and its associated processes predate the present work. The ontology API and ICTV-NCBI mappings are intended to complement these existing interactions, not to replace or disrupt them.

In parallel, ICTV is using a dedicated access mechanism for its data exchange with NCBI and plans a broader API providing access to a more comprehensive ICTV dataset. These services complement the public OLS-based API and support more specialised or higher-volume exchanges.

We made targeted revisions to the Introduction, the ICTV-NCBI mapping section and the Discussion to clarify these points.

Location in revised manuscript. Introduction; Results: ICTV–NCBI mapping; Discussion

R1.2

Comment

> The taxonomy change modelling in Figure 1 was very helpful. It helped me understand the ICTV hierarchy and use of ICTV identifiers. Would it be possible to provide figures demonstrating the use of isolate and TN identifiers? It is not clear to me how those will be used.

Response. We thank the reviewer for this helpful suggestion. We clarified that TN identifiers are used as ICTV source-table keys during ontology generation. They are used to reconstruct parent-child relationships, associate VMR records with taxon nodes and interpret changes between releases, but are not embedded in the generated ontology. Public release-specific taxon states are instead represented as OWL classes with resolvable IRIs combining the MSL release and the persistent ICTV ID. We also added Figure 2 and accompanying text to show how historical and current taxon representations, replacement and revision relations, and VMR virus exemplar records are modelled as OWL named individuals and support taxon resolution.

Location in revised manuscript. Methods: Persistent identifiers and unified ontology; Figure 2; Additional file 1, Supplementary Material S1

R1.3

Comment

> Figure 1 was very clear in how changes occur in the hierarchy in different ICTV releases. However, when I went to look in the ICTV Taxon Resolver, I wouldn't have understood the changes as clearly without that figure. For example, when I looked up Alphabaculovirus, it doesn't show ever having any relation to Nucleopolyhedrovirus like it does in the figure. How far back does the Taxon Resolver go? EBI-OLS suggests that the ontology was built on MSL41, so previous changes may not be modelled. Could the authors provide a little more information about this? And is there a user guide available that could provide some guidance on using the interface? I couldn't find one on the website or in the GitHub documentation, and I think it could be very helpful for those familiarizing themselves with the ICTV hierarchy and the tool.

Response. We thank the reviewer for highlighting this point. We clarified that the unified ontology contains every available release from MSL1 to MSL41. The Resolver description now states that "Show history across MSLs" starts from the OWL class representing the taxon identified from the query and displays the release-specific OWL classes linked to it across the MSL releases included in the ontology. A history may therefore begin after MSL1 or end before MSL41 if a taxon was introduced later or subsequently abolished. We also state that the relationship between Alphabaculovirus and Nucleopolyhedrovirus illustrated in Figure 1 can be traced in history mode from MSL23 back to MSL13. The goal of the default Resolver mode, "Resolve to latest ICTV", is to efficiently link to the latest version of a term. The alternative "Show history across MSLs" mode displays the linked release-specific OWL classes described above, while full histories are also available on the ICTV website.

Location in revised manuscript. Abstract—Findings; Results: ICTV Taxon Resolver

R1.4

Comment

> Is there a user guide available that could provide some guidance on using the interface? I couldn't find one on the website or in the GitHub documentation, and I think it could be very helpful for those familiarizing themselves with the ICTV hierarchy and the tool.

Response. We thank the reviewer for this useful suggestion. Concise usage guidance is displayed directly beneath the ICTV Taxon Resolver interface title, and the revised Results section draws attention to it. We also added a direct citation to the ICTV-specific API and helper-library documentation and expanded Supplementary Material S1 with direct OLS requests and a working JavaScript example.

Location in revised manuscript. Results: ICTV Taxon Resolver and developer documentation; Additional file 1, Supplementary Material S1

R1.5

Comment

> The authors reference the Disease Ontology and using semantic practices prescribed by the OBO Foundry. Does the ICTV ontology use BFO/COB and RO as building blocks? It was unclear as I looked in the documentation. It also does not appear that the ICTV ontology has been submitted to the OBO Foundry, can the authors describe whether this is planned?

Response. We thank the reviewer for raising this point. We clarified the ontology's scope: it reuses selected ontology terms and annotations but does not import BFO, COB or RO. It has not been submitted to the OBO Foundry, and no submission is currently planned.

Location in revised manuscript. Methods: Ontology modelling and unified-ontology redesign

Response to Reviewer 2

We thank the reviewer for the detailed comments and respond point by point below.

R2.1

Comment

> Title: consider including OLS.

Response. We considered this suggestion carefully. OLS was included in earlier versions of the title. We ultimately replaced “OLS API” with “public ontology API” to make the title immediately understandable to readers who may not know OLS and to avoid adding another acronym to the title. ICTV and API were retained because they are central to the paper and are commonly understood across the intended audience. To make the role of OLS clearer, we added a sentence at the end of the

Introduction describing its broader role in making biological and biomedical ontologies searchable, browsable and programmatically accessible through standard APIs. OLS is also identified explicitly in the Abstract and described throughout the manuscript. We therefore retained the current title.

Location in revised manuscript. Title; Abstract; Introduction

R2.2

Comment

> Abstract, line 27: Replace 'framework' with 'ontology' or similar.

Response. We thank the reviewer for prompting this clarification. We replaced 'framework' in the Abstract with a precise description of the relevant components: 'Complementary ICTV–NCBI mappings and helper libraries support integration into downstream systems.' In other project-specific passages, we likewise used the component names or pointed to the previously described approach. We retained 'framework' only where it has a generic meaning, such as 'taxonomic framework', or occurs in the title of a cited source.

Location in revised manuscript. Abstract—Findings; Key Points; Results; Figure 3; Discussion; Conclusions

R2.3

Comment

> Introduction, line 105: clarify 'to make meaning human and machine readable', e.g. as 'through explicit, human- and machine-readable semantics'.

Response. We agree and revised the sentence to state that ontologies provide explicit, human- and machine-readable semantics that support the consistent annotation, sharing and interpretation of complex datasets.

Location in revised manuscript. Introduction

R2.4

Comment

> Throughout the manuscript: consider replacing 'viral taxonomy' with 'virus taxonomy' and other instances.

Response. We thank the reviewer for this terminology suggestion. We reviewed the occurrences contextually and now use 'virus taxonomy' when referring to the ICTV taxonomic system, which includes taxa at all ranks. We retained scientifically appropriate uses of 'viral' where it modifies another noun, for example 'viral taxa'.

Location in revised manuscript. Abstract; main manuscript; Additional file 1

R2.5

Comment

> Results, line 244: 'Mappings are maintained in a dedicated repository and updated independently.' Which GitHub repository?

Response. We thank the reviewer for identifying this omission. The revised Results section now names and cites the dedicated EVORA virus-taxonomy-mappings repository, and the Data availability statement cites the exact mapping release archived in Zenodo together with its concept DOI for all versions.

Location in revised manuscript. Results: ICTV–NCBI mapping; Data availability; References

R2.6

Comment

> Results, line 284: remove second 'pathogens'.

Response. Thank you for noting this duplication. The second occurrence of 'pathogens' was removed.

Location in revised manuscript. Results: Reference application and early adopters

R2.7

Comment

> Please include links to documentation and/or tutorials for app developers apart from the supplementary material, and a plan for the dissemination of the resources.

Response. We thank the reviewer for this helpful suggestion. The revised Results section now cites the public ICTV-specific developer documentation and helper libraries, and Supplementary Material S1 provides detailed API and helper-library examples. The Availability and sustainability section now describes dissemination and discoverability through OLS, versioned GitHub and Zenodo releases, and registration of the software resources in bio.tools and SciCrunch.

Location in revised manuscript. Results: API/helper-library documentation; Methods: Availability and sustainability; Availability of supporting source code and requirements; Additional file 1, Supplementary Material S1

R2.8

Comment

> Discussion, line 310: Temper 'much easier to access' and focus on the improved technical capability.

Response. We agree and replaced the subjective phrase 'much easier to access' with a specific description of the improved technical capability: downstream systems now have programmatic access to current and historical taxonomic information and can resolve former terms to the current classification, supporting uses from direct queries to recurring automated updates.

Location in revised manuscript. Discussion

R2.9

Comment

> Throughout the manuscript: the wording 'alignment with FAIR principles' or 'FAIR-aligned' is sufficient; FAIR-compliant is not necessary as there is not a defined compliance standard.

Response. We agree. We revised the manuscript to use 'FAIR-aligned' or similarly qualified wording rather than claiming full FAIR compliance.

Location in revised manuscript. Discussion; Conclusions; Additional file 1

R2.10

Comment

> Figure 1: consider changing the red color to color-blind-friendly magenta.

Response. We thank the reviewer for this accessibility suggestion. The red encoding in Figure 1 was replaced with the colour-blind-safe dark magenta #AA3377 (RGB 170, 51, 119) from Paul Tol's bright qualitative colour scheme.

Location in revised manuscript. Figure 1

R2.11

Comment

> Supplementary Material S1: please add a concrete example and screenshot of result in tutorial style.

Response. Supplementary Material S1 now provides a worked example for resolving a former name using direct OLS API requests, identifies the replacement relation followed to the current OWL class, and gives the equivalent JavaScript helper-library example. The corresponding completed Resolver example is shown in the new Figure 4, providing a visual result without duplicating the same material in the supplement.

Location in revised manuscript. Results: ICTV Taxon Resolver; Figure 4; Additional file 1, Supplementary Material S1

Closing statement

We thank the Editor and Reviewers again for their constructive assessment. We believe that the revisions improve the clarity, reproducibility and usability of the manuscript and its associated resources.

Additional file 1: supplementary material

Click here to access/download
Supplementary Material
Additional_file_1_Supplementary_material.pdf

Revised submission of manuscript GIGA-D-26-00217: "Programmatic access to ICTV virus taxonomy through a public ontology API"

Dear Editors,

Please find attached the revised version of our manuscript entitled "Programmatic access to ICTV virus taxonomy through a public ontology API", submitted for further consideration as a Technical Note in *GigaScience* (manuscript GIGA-D-26-00217). We have addressed all the reviewers' comments and hope that we have responded to them adequately.

Thank you for your time and consideration. We look forward to your feedback.

Sincerely,

Philippe Lieutaud
Chief Technical Officer, European Virus Archive
WP7 Leader, European Viral Outbreak Response Alliance
Aix-Marseille Université
Email: Philippe.Lieutaud@univ-amu.fr
