# Supplementary material for: Programmatic access to ICTV virus taxonomy through a public ontology API
Source: Gigascience. 2026 Sep 4;15:giag089. doi: 10.1093/gigascience/giag089 (PMC13615544; doi:10.1093/gigascience/giag089)
Supplement: Supplementary file 4 [file giag089_reviewer_1_report_original_submission.pdf]

Reviewer Report

Title: Programmatic access to ICTV virus taxonomy through a public ontology API

Version: Original Submission **Date:** 7/9/2026

Reviewer name: Reviewer 1

Reviewer Comments to Author:

The manuscript entitled "Programmatic access to ICTV virus taxonomy through a public ontology API" describes the development of an ontology for the ICTV virus taxonomy hierarchy (genera as classes, species as individuals; identifiers for taxa across hierarchy changes and for tracking taxa changes within ICTV releases, identifiers for isolates that link to Genbank) based on DCTERMS, OIO (oboInOWL), OMO (OBO Metadata Ontology), IAO and NCBITaxon. The authors also developed mappings between ICTV taxonomy and NCBITaxon, and an API for updating resources reliant on NCBITaxon. The authors also created a tool called ICTV Taxon Resolver that provides mappings between deprecated taxon rankings and new taxon relationships to better enable tracking of changes over time. The authors describe the challenge of updating NCBITaxon, including delays in taxa ranking updates and the creation of new classes/taxids rather than synonymizing new names with existing (old) nomenclature. The ICTV ontology was integrated into EBI-OLS (ontology look-up service) to enable easy searching and discovery for public users, and is also integrated into the EVORA Portal which hosts virology resources to support pandemic preparedness as well as the Pandemic PACT program (included in their dashboard).
The manuscript describes the benefits of ontologies (data integration, enhanced interoperability, standardization empowering data reuse, etc), and seeing more ontologies in action is very exciting - I am a big champion of ontologies and use EBI-OLS nearly every day. The ecosystem of resources offered by ICTV is also wonderful to see. Figure 1 is very useful as it shows how changes can occur in the taxonomy and how they could be tracked using ICTV identifiers and the ontology. I looked up this example in the ICTV Taxon Resolver to see how it would be visualized. The architecture and ecosystem presented in Figure 2 is very helpful, as is the Zika synonymy representation in the ICTV Taxon Resolver in Figure 3. Code for tools is made publicly available in multiple GitHub repositories and documentation for the API is also published. Overall, this is a well written paper and a valuable project. I enjoyed reading it.
I have a few questions and suggestions for the authors.
1. Have NCBI been included in conversations about these developments? It seems strange that representatives from EBML-EBI are authors but there is no inclusion of partners from NCBI when NCBITaxon is a key resource for many platforms, application ontologies, and tools. Could the authors mention engagement efforts with NCBI so we understand whether the ICTV IDs and synonymy will be adopted? I think this is important as well as the authors pointedly describe the challenges of updating taxonomy at NCBI, but don't note that major consumers/providers of data are public health agencies. Every change in NCBITaxon breaks critical workflows in systems used in routine public health operations. NCBI is caught between a rock and a hard place every time ICTV makes changes - wanting to respect expert recommendations while managing pushback from the community e.g. there were/are many challenges of adopting the change of "Severe acute respiratory syndrome-related coronavirus" to

"Betacoronavirus pandemicum" including adapting communication strategies with the public and Ministries of Health. Understanding communication/collaborations with NCBI would be helpful.
2. The taxonomy change modelling in Figure 1 was very helpful. It helped me understand the ICTV hierarchy and use of ICTV identifiers. Would it be possible to provide figures demonstrating the use of isolate and TN identifiers? It is not clear to me how those will be used.
3. Figure 1 was very clear in how changes occur in the hierarchy in different ICTV releases. However, when I went to look in the ICTV Taxon Resolver, I wouldn't have understood the changes as clearly without that figure. For example, when I looked up Alphabaculovirus, it doesn't show ever having any relation to Nucleopolyhedrovirus like it does in the figure. How far back does the Taxon Resolver go? EBI-OLS suggests that the ontology was built on MLS41, so previous changes may not be modelled. Could the authors provide a little more information about this? And is there a user guide available that could provide some guidance on using the interface? I couldn't find one on the website or in the GitHub documentation, and I think it could be very helpful for those familiarizing themselves with the ICTV hierarchy and the tool.
4. The authors reference the Disease Ontology and using semantic practices prescribed by the OBO Foundry. Does the ICTV ontology use BFO/COB and RO as building blocks? It was unclear as I looked in the documentation. It also does not appear that the ICTV ontology has been submitted to the OBO Foundry, can the authors describe whether this is planned?
Taken together, I think this collection of resources will be a valuable contribution to the community. If the authors can address my questions/suggestions, I recommend publication.
Questions for Reviewers:
1) Are the methods appropriate to the aims of the study, are they well described, and are necessary controls included?
Yes
2) Are the conclusions adequately supported by the data shown?
Yes
3) Please indicate the quality of language in the manuscript. Does it require a heavy editing for language and clarity?
Language is acceptable.
4) Are you able to assess all statistics in the manuscript, including the appropriateness of statistical tests used?
Not applicable.

Methods

Are the methods appropriate to the aims of the study, are they well described, and are necessary controls included? Choose an item.

Conclusions

Are the conclusions adequately supported by the data shown? Choose an item.

Reporting Standards

Does the manuscript adhere to the journal's guidelines on [minimum standards of reporting?]() Choose an item.

Choose an item.

Statistics

Are you able to assess all statistics in the manuscript, including the appropriateness of statistical tests used? Choose an item.

Quality of Written English

Please indicate the quality of language in the manuscript: Choose an item.

Declaration of Competing Interests

Please complete a declaration of competing interests, considering the following questions:

- Have you in the past five years received reimbursements, fees, funding, or salary from an organisation that may in any way gain or lose financially from the publication of this manuscript, either now or in the future?
- Do you hold any stocks or shares in an organisation that may in any way gain or lose financially from the publication of this manuscript, either now or in the future?
- Do you hold or are you currently applying for any patents relating to the content of the manuscript?
- Have you received reimbursements, fees, funding, or salary from an organization that holds or has applied for patents relating to the content of the manuscript?
- Do you have any other financial competing interests?
- Do you have any non-financial competing interests in relation to this paper?

If you can answer no to all of the above, write 'I declare that I have no competing interests' below. If your reply is yes to any, please give details below.

I declare that I have no competing interests.

I agree to the open peer review policy of the journal. I understand that my name will be included on my report to the authors and, if the manuscript is accepted for publication, my named report including any attachments I upload will be posted on the website along with the authors' responses. I agree for my report to be made available under an Open Access Creative Commons CC-BY license (http://creativecommons.org/licenses/by/4.0/). I understand that any comments which I do not wish to be included in my named report can be included as confidential comments to the editors, which will not be published.

I agree to the open peer review policy of the journal

To further support our reviewers, we have joined with Publons, where you can gain additional credit to further highlight your hard work (see: https://publons.com/journal/530/gigascience). On publication of this paper, your review will be automatically added to Publons, you can then choose whether or not to claim your Publons credit. I understand this statement.

Yes Choose an item.
