# Supplementary material for: Programmatic access to ICTV virus taxonomy through a public ontology API
Source: Gigascience. 2026 Sep 4;15:giag089. doi: 10.1093/gigascience/giag089 (PMC13615544; doi:10.1093/gigascience/giag089)
Supplement: Supplementary file 6 [file giag089_reviewer_2_report_original_submission.pdf]

Reviewer Report

Title: Programmatic access to ICTV virus taxonomy through a public ontology API

Version: Original Submission **Date:** 7/20/2026

Reviewer name: Reviewer 2

Reviewer Comments to Author:

Summary
This manuscript presents a timely resource that provides the ICTV Master Species List (MSL) releases as a unified, machine-accessible representation served through the Ontology Lookup Service (OLS). The OLS ICTV resource enables public programmatic access to current and historical ICTV taxonomy, provides ICTV-NCBI mappings and integration libraries, and aims to improve interoperability, versioning, and FAIR-aligned management of virus taxonomy data. The work demonstrates a generalizable approach for exposing structured biological datasets as semantically enriched resources through scalable APIs. The manuscript is in general well-written, clear and with all relevant resources linked.
Scope
The manuscript is well suited to GigaScience, as it describes a reusable bioinformatics infrastructure and community resource that promotes open access, interoperability, and FAIR data principles. The work describes a sustainable, publicly accessible ontology and corresponding API tool for virus taxonomy, making it highly relevant to the journal's readers and the scientific community interested in biological databases, ontologies, computational infrastructure, and data integration.
My recommendation is a revision with minor changes as listed below.
Title
- consider including OLS
Abstract
- L27 Replace "framework" with "ontology" or similar
Introduction
- L105 clarify "to make meaning human and machine readable" e.g. as "through explicit, human- and machine-readable semantics"
- Throughout the manuscript: consider replacing "viral taxonomy" with "virus taxonomy" and other instances
Results
-L244: "Mappings are maintained in a dedicated repository and updated independently." -- which Github repo?
-L284: remove second "pathogens"
- please include links to documentation and/or tutorials for app developers apart from the supplementary material, and a plan for the dissemination of the resources.
Discussion
- L310: Temper "much easier to access" and focus on the improved technical capability
- Throughout the manuscript: the wording "alignment with FAIR principles" or "FAIR-aligned" is

sufficient, FAIR-compliant is not necessary as there is not a defined compliance standard.
Figure 1
- consider changing the red color to color-blind-friendly magenta
SMS1
- please add a concrete example and screenshot of result in tutorial style

Methods

Are the methods appropriate to the aims of the study, are they well described, and are necessary controls included? Choose an item.

Conclusions

Are the conclusions adequately supported by the data shown? Choose an item.

Reporting Standards

Does the manuscript adhere to the journal's guidelines on [minimum standards of reporting?]() Choose an item.

Choose an item.

Statistics

Are you able to assess all statistics in the manuscript, including the appropriateness of statistical tests used? Choose an item.

Quality of Written English

Please indicate the quality of language in the manuscript: Choose an item.

Declaration of Competing Interests

Please complete a declaration of competing interests, considering the following questions:

- Have you in the past five years received reimbursements, fees, funding, or salary from an organisation that may in any way gain or lose financially from the publication of this manuscript, either now or in the future?
- Do you hold any stocks or shares in an organisation that may in any way gain or lose financially from the publication of this manuscript, either now or in the future?
- Do you hold or are you currently applying for any patents relating to the content of the manuscript?
- Have you received reimbursements, fees, funding, or salary from an organization that holds or has applied for patents relating to the content of the manuscript?

- Do you have any other financial competing interests?
- Do you have any non-financial competing interests in relation to this paper?

If you can answer no to all of the above, write 'I declare that I have no competing interests' below. If your reply is yes to any, please give details below.

I declare that I have no competing interests

I agree to the open peer review policy of the journal. I understand that my name will be included on my report to the authors and, if the manuscript is accepted for publication, my named report including any attachments I upload will be posted on the website along with the authors' responses. I agree for my report to be made available under an Open Access Creative Commons CC-BY license (http://creativecommons.org/licenses/by/4.0/). I understand that any comments which I do not wish to be included in my named report can be included as confidential comments to the editors, which will not be published.

I agree to the open peer review policy of the journal

To further support our reviewers, we have joined with Publons, where you can gain additional credit to further highlight your hard work (see: https://publons.com/journal/530/gigascience). On publication of this paper, your review will be automatically added to Publons, you can then choose whether or not to claim your Publons credit. I understand this statement.

Yes Choose an item.
